# Visible light mediated iron-catalyzed addition of oxamic acids to imines†

**DOI:** 10.1039/d4ra02258k

**Published:** 2024-04-18

**Authors:** Margaux Badufle, Frédéric Robert, Yannick Landais

**Affiliations:** a Univ. Bordeaux, CNRS, Bordeaux INP, ISM, UMR 5255 F-33400 Talence France yannick.landais@u-bordeaux.fr

## Abstract

Oxamic acids where shown to add to imines, providing a broad range of α-aminoacid amides in generally good yields. The process is efficient on pre-formed imines but may also be conducted using a 3-component strategy by simply mixing aldehydes, amines and oxamic acids in the presence of ferrocene, acting both as a photocatalyst under visible light and as a Lewis acid. The reaction proceeds through the addition onto the imine of a carbamoyl radical intermediate generated through a charge transfer from the carboxylate ligand to a Fe(iii) species (LMCT).

## Abstract 2

Oxamic acids where shown to add to imines, providing a broad range of α-aminoacid amides in generally good yields.

## Graphical abstract

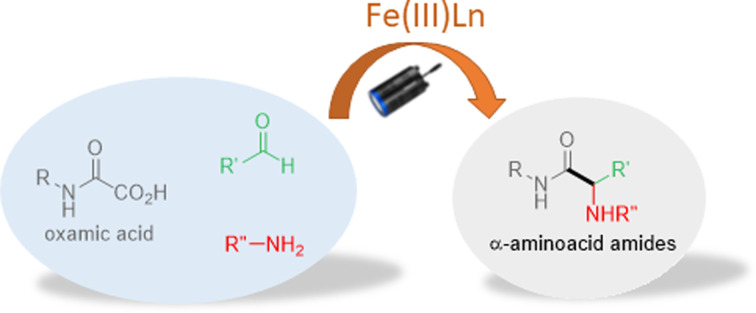

Carbamoyl radicals add efficiently to a wide range of unsaturated systems, including alkenes,^1^ alkynes,^2^ arenes^3^ and heteroarenes (Minisci reaction),^4^ allowing the straightforward incorporation of the amide functional group onto carbon skeletons (Fig. 1A). The generation and use of this class of radicals has recently undergone a major boom.^5^ Carbamoyl radicals can be generated from a fairly wide variety of precursors, including formamides, oxamic acids and their corresponding potassium salts or activated esters, 4-substituted-1,4-dihydropyridines (DHPs) esters and carbamoyl chlorides.^5^ Photo-induced and photocatalyzed processes have profoundly modified recently the way to generate carbamoyl radicals and extended their scope of application.^5*b*,*c*^ Amongst the above precursors, formamides appear as relatively ideal and their conversion into carbamoyl radicals has been carried out through hydrogen-atom transfer process using either Fenton-type methods^6^ or direct or indirect HAT using TBADT or alkoxy radicals.^7^ Although quite efficient, this approach suffers from the potential concurrent C–H abstraction at CHO and α to nitrogen. In turn, carbamoyl chlorides have the disadvantage of being accessible using particularly toxic phosgene derivatives,^8^ while DHP ester derivatives suffer from their low attractiveness in terms of atom-economy.^9^ In contrast, oxamic acids are readily available by combination of amines with oxalic acid derivatives^5*b*^ and only produce CO_2_ as by-product upon oxidation, explaining their attractiveness as precursors of carbamoyl radicals.^1*k*,3,4*c*,5*b*^

**Fig. 1:**
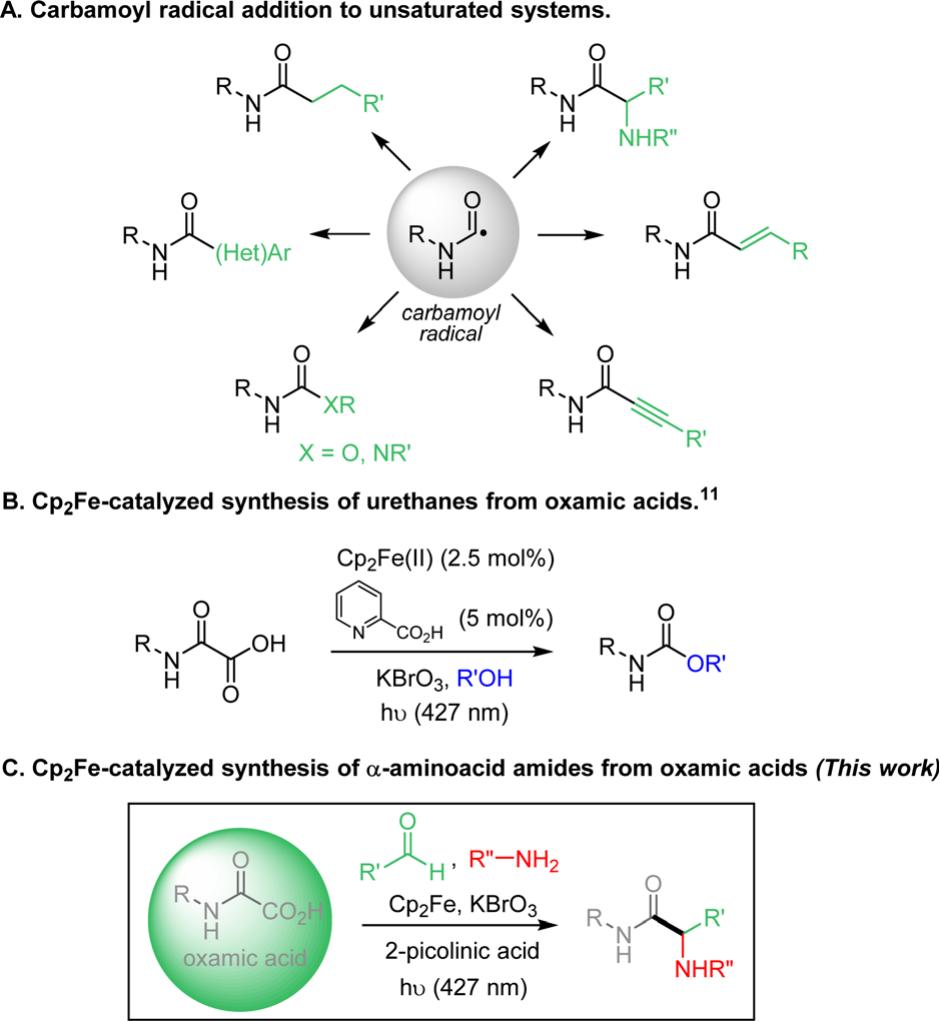
Generation of carbamoyl radicals from oxamic acids and their additions to unsaturated systems.

In this context, we recently reported several methods to generate carbamoyl radicals from simple oxamic acids, using photoredox conditions (including visible and NIR light) and electrochemistry.^4*b*,10^ More recently, our laboratory developed a new oxamic acid decarboxylation procedure using Cp_2_Fe as a catalyst and KBrO_3_ as an oxidant, which relied on a photocatalyzed LMCT process (Fig. 1B).^11^ This modified procedure has the advantage to be practically simple, does not require strict anhydrous and deaerated conditions and uses cheap iron catalysts and oxidant. We report here an application of this visible-light mediated LMCT process to the addition of oxamic acids onto imines (Fig. 1C). The addition of the carbamoyl motif to imines has been very little developed to date,^12,13^ even though it gives access to the α-aminoacid amide fragment, the polypeptides basic unit. This iron-based procedure enables the carbamoylation of imines in generally good yields under mild conditions. A three-component process, through the simple mixing of oxamic acids, anilines and aldehydes under standard catalytic conditions was also developed, which showed broad scope and robustness.

Reaction conditions were first optimized with the preparation of α-aminoacid amide 3a, as summarized in Table 1, using pre-formed imine 2a and oxamic acid 1a as models. Cp_2_Fe (2.5 mol%) was used as the iron catalyst in the presence of 2-picolinic acid as the ligand in DCE. Without any acid or oxidant additives, the coupling led to only 2% of the desired amide 3a (Table 1, entry 1). Acids such as trifluoroacetic acid and BF_3_–Et_2_O were then added to activate the imine function, which led to improved yields of 3a as shown in entries 2 and 3, in good agreement with studies of Jacobi von Wangelin *et al.*^12*b*^ This result thus shows that the catalytic cycle is operative in the absence of a terminal oxidant (*vide infra*). However, all our efforts to further improve the conversion under these “acidic conditions” met with failure. We then turned our attention to the use of KBrO_3_, which proved to be the best terminal oxidant in our previous studies.^11^ This modification led to much improved yield (entry 4). Solvents were also varied, indicating that DCE is superior (entries 5 and 6). Various amount of KBrO_3_ was then tested, showing that optimal yield could be reached using only 0.5 eq. of oxidant (entries 8 and 9). Varying the amount of oxamic acid (entry 10) or imine (11) slightly improved the yield. However, we also observed that under these conditions, the excess of 1a or 2a made the final purification of 3a more tedious. The amount of ligand was also varied (entries 12 and 13) as well as that of the iron catalyst (entry 14), which did not modify the yield to a large extent. Control experiments were performed, showing that, Cp_2_Fe and light were both essential for the process to occur (entries 15 and 16), while the absence of ligand had a minor effect on the conversion (entry 17). Finally, the process was repeated using air and O_2_ atmosphere as oxidants (entries 18 and 19). Air led to a moderate yield, while pure oxygen provided 3a in trace amount.

**Table 1:** Addition of oxamic acid 1a to imine 2a

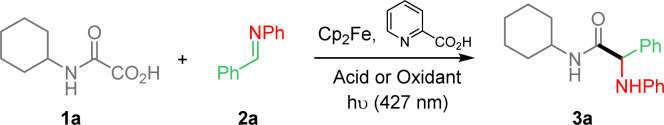							
Entry^a^	Oxamic acid (eq.)	Imine (eq.)	Acid or oxidant (eq.)	Fe cat. (mol%)	Ligand (mol%)	Solvent	Yield^b^ (%)
1	1.0	1.0	—	2.5	5.0	DCE	2
2	1.0	1.0	TFA (1.0)	2.5	5.0	DCE	48
3	1.0	1.0	BF_3_–Et_2_O (1.0)	2.5	5.0	DCE	20
4	1.0	1.0	KBrO_3_ (1.0)	2.5	5.0	DCE	71
5	1.0	1.0	KBrO_3_ (1.0)	2.5	5.0	MeCN	45
6	1.0	1.0	KBrO_3_ (1.0)	2.5	5.0	PhCl	25
7	1.0	1.0	KBrO_3_ (0.5)	2.5	5.0	DCE	70
8	1.0	1.0	KBrO_3_ (0.2)	2.5	5.0	DCE	60
9	1.0	1.0	KBrO_3_ (2.0)	2.5	5.0	DCE	71
10	1.3	1.0	KBrO_3_ (0.5)	2.5	5.0	DCE	77
11	1.0	1.3	KBrO_3_ (0.5)	2.5	5.0	DCE	75
12	1.3	1.0	KBrO_3_ (0.5)	2.5	2.5	DCE	59
13	1.3	1.0	KBrO_3_ (0.5)	2.5	7.5	DCE	76
14	1.3	1.0	KBrO_3_ (0.5)	5.0	10	DCE	70
15^c^	1.3	1.0	KBrO_3_ (0.5)	2.5	5.0	DCE	NA
16^d^	1.3	1.0	KBrO_3_ (0.5)	—	5.0	DCE	NA
17^e^	1.3	1.0	KBrO_3_ (0.5)	2.5	—	DCE	69
18^f^	1.3	1.0	Air	2.5	5.0	DCE	45
19^f^	1.3	1.0	O_2_	2.5	5.0	DCE	5

a Unless otherwise mentioned, all reactions were performed with Cp_2_Fe (2.5 mol%) and ligand (5 mol%) in the indicated solvent (0.1 M), in a sealed tube.

b Isolated yields of 3a.

c Absence of blue LED.

d Absence of Cp_2_Fe.

e Absence of ligand.

f Yields of 3a determined by ^1^H NMR with 1,3,5-trimethylbenzene as an external standard.

From these results (Table 1, entry 7), the substrate scope was extended, varying the nature of oxamic acids 1 using pre-formed imines 2a (Scheme 1). The mild reaction conditions allowed the formation of various α-aminoacid amides 3a–r in moderate to high yields. Reaction conditions are compatible with the presence on the oxamic acid structure of electron-rich arenes such as thiophene (3f) or alkoxyarenes as in 3m–o, and electron-poor arenes (3p–r). Substrates having benzylic hydrogens as in 3d–e, 3k or 3l led to high isolated yields, suggesting that competitive 1,5-HAT from a putative aminyl radical does not operate. *Ortho*-substituted aryloxamic acids were converted into amides 3k and 3o in satisfying yields. Oxamic acids issued from α-aminoacid ^4*b*^ led to the desired product (3g) in moderate yield as a mixture of two diastereomers in a 1 : 1 ratio. Surprisingly, secondary oxamic acid provided the desired product 3h albeit in modest yield, while the same precursor was shown to fail to deliver the desired isocyanate under similar conditions.^11^*N*-Acetylphenylhydrazone, known for its high reactivity toward C-centered radicals also failed to react under our conditions (ESI†).^14^ The scope of the methodology was further extended varying the nature of all partners as summarized in Scheme 2.

**Scheme 1:**
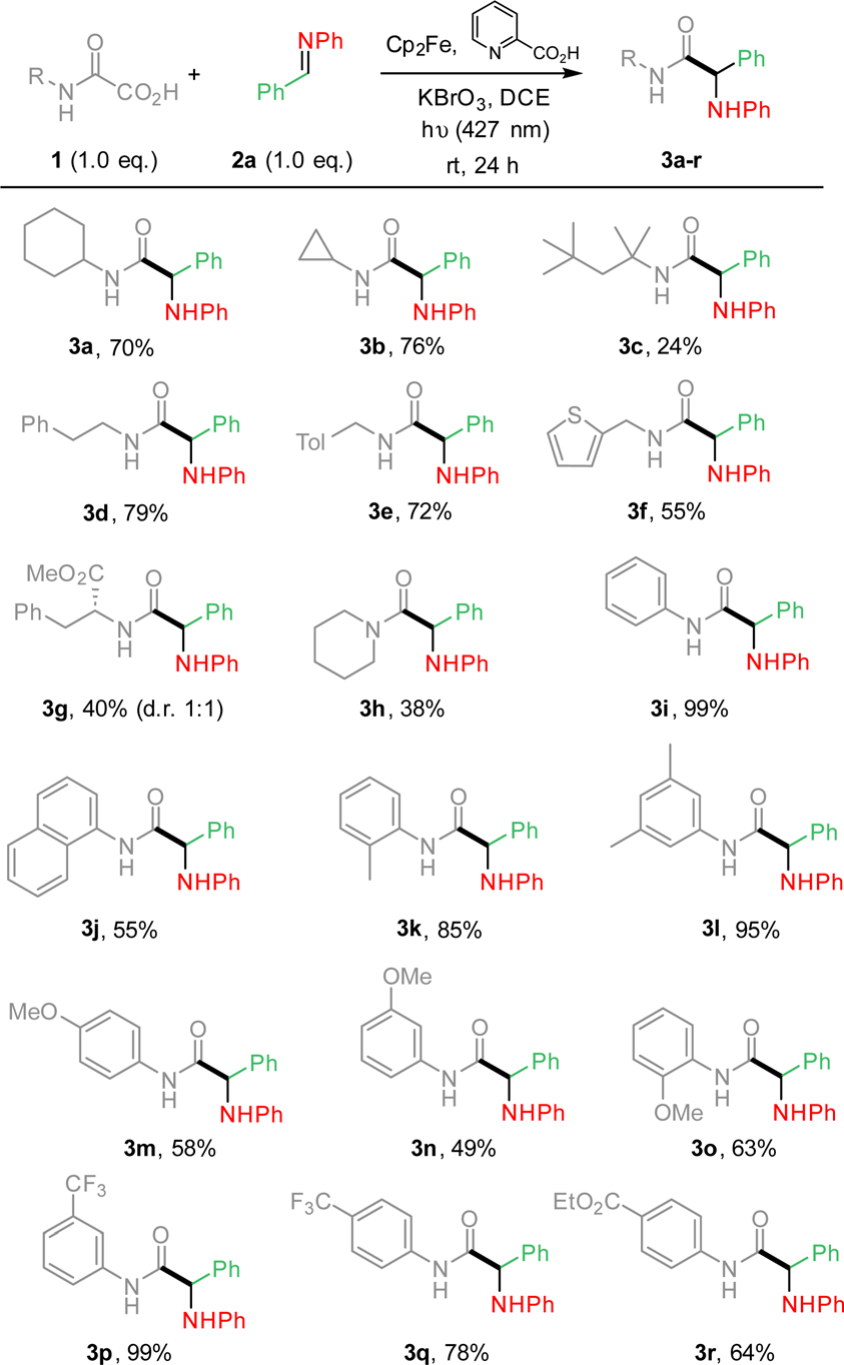
Photocatalyzed Cp_2_Fe-mediated addition of oxamic acids onto imines. Oxamic acid scope.

**Scheme 2:**
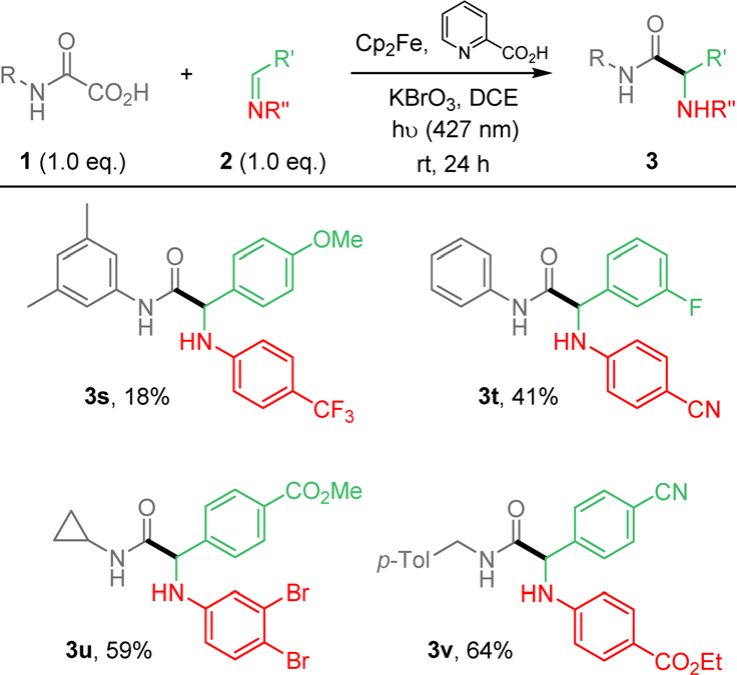
Photocatalyzed Cp_2_Fe-mediated addition of oxamic acids onto imines. 2-component strategy.

We then decided to implement the methodology by developing a more practical 3-component approach to extend the scope of application and potentially enable future automation of this reaction. Optimization of the process was carried out, using oxamic acid 1a, benzaldehyde 4a and aniline 5a to afford α-aminoacid amide 3a (ESI†). The best yield was obtained using a 1.0 : 1.3 : 1.3 ratio of 1a/4a/5a (Table S1, ESI†). However, due to purification issues, conditions using 1.0/1.0/1.0 ratio were finally retained and applied to the 3-component process as summarized in Scheme 3. Compounds 3 were thus generally accessible in moderate to good yields. The 3-component approach compares well with the 2-component version in terms of yields (Scheme 2*vs.*3). As above, reaction conditions are compatible with various substituents and functional groups on arene moieties, including free OH, halogens, esters, nitriles and fluorine-containing substituents. 3y having an amino substituent on the aldehyde fragment was observed in ^1^H NMR but could not be isolated pure. Finally aliphatic aldehydes and aliphatic or benzylamines as well as sulfonylamines (not shown) were tested and did not provide the desired addition products (ESI†).

**Scheme 3:**
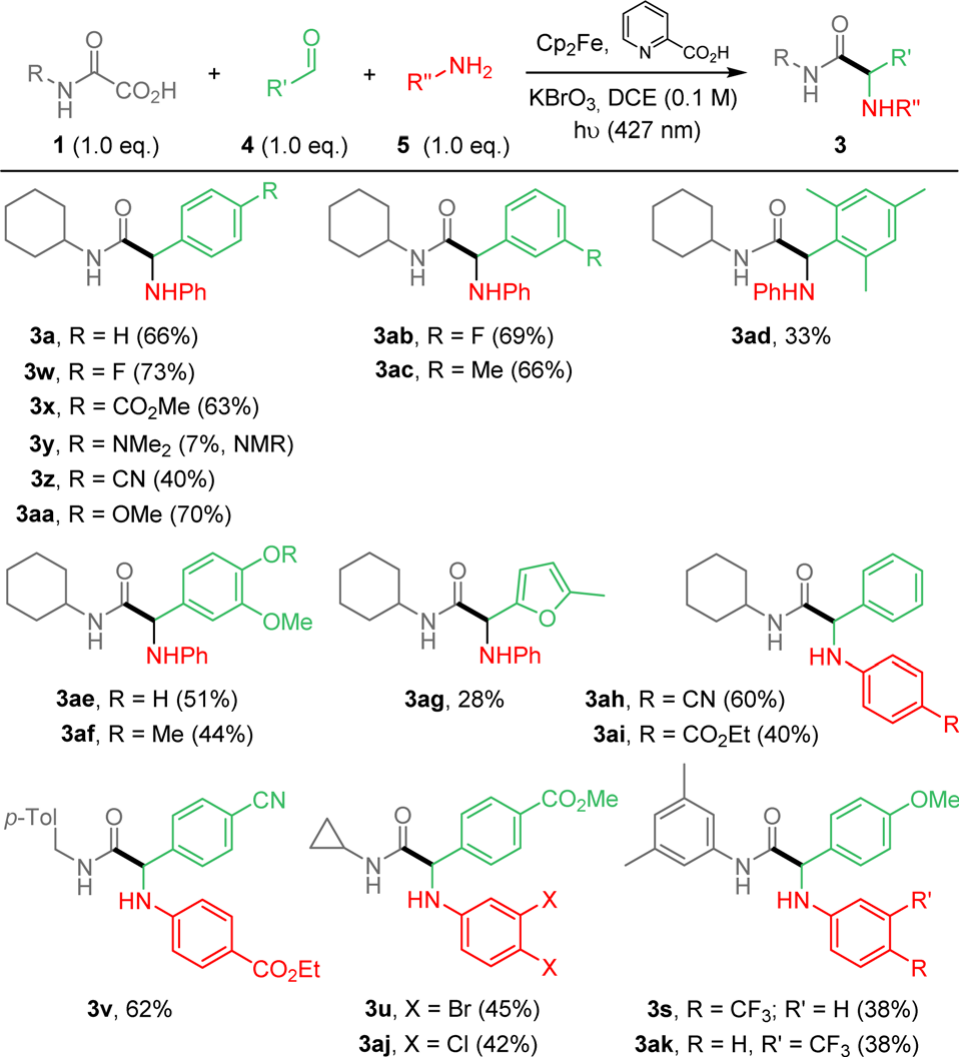
Photocatalyzed Cp_2_Fe-mediated addition of oxamic acids onto imines. 3-component strategy.

Several control experiments were finally carried out to get mechanistic insights (ESI†). For instance, reaction between 1a and 2a under standard conditions, but in the presence of TEMPO, did not provide 3a, suggesting that the process follows a radical pathway. Finally, addition of 1,1-diphenylethylene to 1a and 2a under conditions above, led to a mixture in which the product resulting from the carbamoyl addition onto the olefin was isolated in 33% yield, indicating that a carbamoyl radical was generated during the process. On the basis of the above results and previous reports,^11,12*b*^ a tentative mechanism is finally proposed in Fig. 2. Cp_2_Fe and 2-picolinic acid likely provides mixed Cp_2_Fe–picolinate complexes, which upon oxidation with KBrO_3_ generate the catalytically active species, *i.e.* Fe(iii)Ln.^15^ The latter then combines with oxamic acid 1 to afford the iron–carboxylate I, which suffers a photoactivated Ligand to Metal Charge Transfer (LMCT) leading to carboxyl radical II and Fe(ii)Ln.^16–18^ Decarboxylation of II then forms the carbamoyl radical III, which can add to the imine IV, activated by a Brønsted (TFA, picolinic acid, oxamic acid) or a Lewis acid (BF_3_ or Fe(iii)Ln) to provide the cation-radical V. The latter may finally be reduced by Fe(ii)Ln (path a), to give upon protonation the final α-aminoacid amide 3, regenerating Fe(iii)Ln.^19^ Oxidation potentials of +0.44 V (*vs.* SCE)^20^ and in the range +0.6 to +0.9 V (*vs.* SCE)^21^ in CH_3_CN respectively for Cp_2_Fe/Cp_2_Fe^+^ and ArNH_2_^+^˙/ArNH_2_ indicate that V may effectively be reduced to provide 3 and regenerate Fe(iii)Ln. However the very close potential values may also explain the recourse to an external source of oxidant to maintain the catalytic cycle. It is worth mentioning that additional experiments using Cp_2_Fe^+^ as a catalyst for the reaction between 1a and 2a, in the absence of KBrO_3_, led to 3a in 52% yield, further supporting the catalytic cycle proposed in Fig. 2. In the absence of a Brønsted acid, Fe(iii)Ln and Fe(ii)Ln species likely play a dual role in the catalytic cycle, the former as an oxidant of the oxamate I and both as Lewis acids, to activate the imine partner,^14^ which does not react in the absence of acid activation (*vide supra*).^12^ The need for 0.5 eq. of KBrO_3_ to reoxidize Fe(ii)Ln into Fe(iii)Ln may also be required as to maintain sufficient Fe(iii) in the catalytic cycle (path b), as Fe(iii) is known to bind strongly to nitrogen,^14^ preventing the restoration of the catalytic cycle. Further studies are ongoing in our laboratory to clarify this point.

**Fig. 2:**
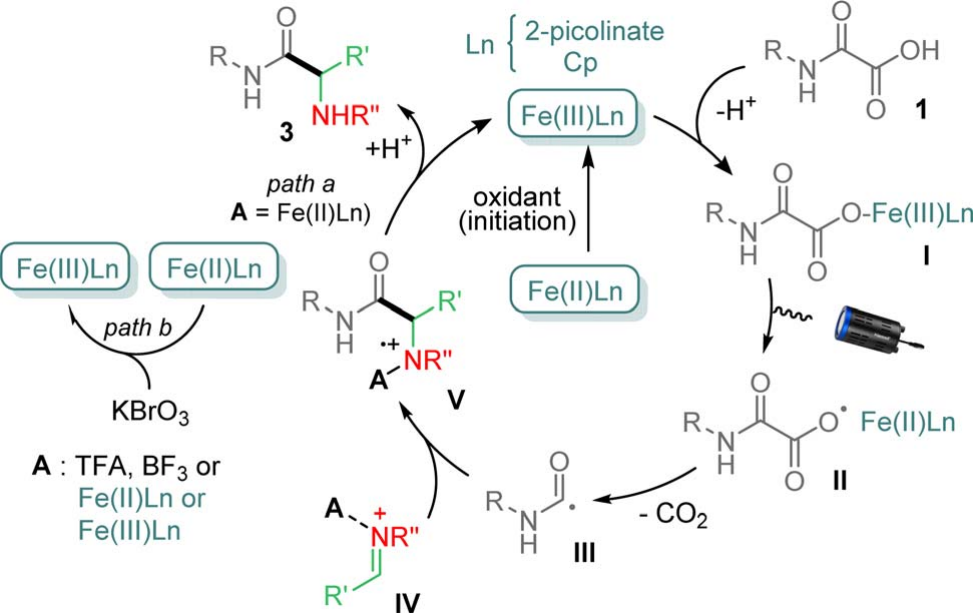
Mechanism of the photocatalyzed Cp_2_Fe-mediated addition of oxamic acids onto imines.

In summary, we reported a straightforward photoactivated ferrocene-mediated addition of oxamic acids onto imines, which provides a broad range of α-aminoacid amides in good yields. The process may be carried out on pre-formed imines or by simply mixing aldehydes, amines and oxamic acids, in the presence of the iron-complex catalyst. The reaction proceeds through the formation of a nucleophilic carbamoyl radical species generated through a LMCT from an oxamate–Fe(iii) intermediate. Iron complexes are believed to play a dual role, both as oxidant of the oxamic acid and as Lewis acid to activate the imine partner. The methodology, which uses readily available starting materials, catalyst and oxidant proceeds under mild conditions and should thus find useful for synthetic applications.

## Conflicts of interest

There are no conflicts to declare.

## Supplementary Material

RA-014-D4RA02258K-s001
