# Supplementary material for: Visible light mediated iron-catalyzed addition of oxamic acids to imines
Source: RSC Adv. 2024 Apr 18;14(18):12528–32. doi: 10.1039/d4ra02258k (PMC11024671; doi:10.1039/d4ra02258k)
Supplement: RA-014-D4RA02258K-s001 [file RA-014-D4RA02258K-s001.pdf]

Electronic Supplementary Material (ESI) for RSC Advances.
This journal is © The Royal Society of Chemistry 2024

SUPPORTING INFORMATION

Visible light Mediated Iron-Catalyzed Addition of Oxamic Acids to Imines

Margaux Badufle,[a] Frédéric Robert,[a] and Yannick Landais*[a]

[a] *Univ. Bordeaux, CNRS, Bordeaux INP, ISM, UMR 5255, F-33400 Talence, France.*

e-mail : yannick.landais@u-bordeaux.fr

Table of content

1. GENERAL INFORMATION 1
2. SYNTHESIS OF OXAMIC ACIDS AS STARTING MATERIALS 1
3. SYNTHESIS OF IMINES AS STARTING MATERIALS 3
4. MECHANISTIC STUDIES 5
5. OPTIMIZATION OF THE THREE-COMPONENT REACTION 7
6. VISIBLE LIGHT MEDIATED IRON-CATALYZED SYNTHESIS OF A-AMINO ACID AMIDES 7
7. UNSUCCESSFUL EXAMPLES AND RATIONALIZATION OF SOME LOW YIELDS 8
8. CHARACTERIZATION OF PRODUCTS 8
9. REFERENCES 24
10. NMR SPECTRA 24

1. General information

Used reagents were purchased from Sigma-Aldrich, TCI, Fluorochem, BDLPharm. Solvents, aldehydes and amines were freshly distillated on CaH_2 or recrystallized in appropriate solvent.

NMR analysis were performed using Bruker NEO 300 equipped with a 5 mm BBFO probe (with z-gradients) which tunes to nuclei in the range between ^{109}Ag and ^{31}P (^{1}H NMR: 300 MHz, ^{19}F NMR: 272 MHz, ^{13}C NMR: 75MHz). All NMR spectra were phased and baseline-corrected. Chemical shifts were referenced to the TMS signal. Unless otherwise indicated, the corresponding chemical shifts (δ) and coupling constants (J) are expressed in ppm and Hz. For the multiplicity: broad singlet = bs, singlet = s, doublet = d, triplet = t, quartet = q, doublet of doublets = dd, and multiplets = m.

FT-IR analysis were performed using a Perkin- Elmer Spectrum 100 using film technique on a KBr disc.

High-resolution mass spectra (HRMS) analysis were obtained thanks to a QExactive^{TM} benchtop Orbitrap mass spectrometer coupled to a Vanquish UHPLC system (Thermo Scientific, San Jose, USA) using electrospray ionization mode. The instrument was equipped with an ESI source and spectra were recorded either in the negative or positive mode. Full MS scans were acquired over the range 50-1300 m/z. Analyses were processed by direct infusion (FIA). Samples were introduced by injection through a 20 μL sample loop from the LC pump. XCalibur software version 4.1 was used for data acquisition and FreeStyle software version 1.5 for processing (Thermo Scientific, San Jose, USA).

Melting point were measured using Stuart melting point apparatus SMP10.

Thin-layer chromatography (TLC) was performed using silica gel 60 F254 pre-coated plates (Merck) and revealed with UV light (254 nm) or Hanessian's stain.

Silica (0.043-0.063 mm) was used for flash column chromatography on silica gel, with Cyclohexane:ethyl acetate (Chex:EtOAc) solvent system as eluent.

2. Synthesis of oxamic acids as starting materials

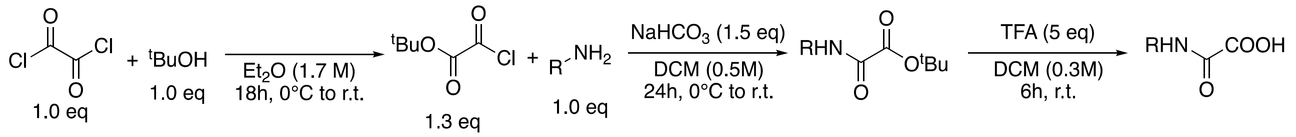

All oxamic acids were synthesized in three steps, following an adapted previous reported procedure[1]

PROCEDURE A:

A solution of ^{t}BuOH (1.0 eq.) in Et_2O (1.7M) was added dropwise into a 0°C solution of oxalyl chloride (1.0 eq.) in Et_2O (2.5M). The mixture was warmed up to r.t. and stirred at r.t. overnight. Excess of solvent and oxalyl chloride were removed to get *tert*-butyl oxalyl chloride, which was used as obtained in the next reaction.

A solution of *tert*-butyl oxalyl chloride (1.3 eq.) in DCM (1M) was added dropwise to a 0°C solution of corresponding amine (1.0 eq.) and $NaHCO_3$ (1.5 eq) in DCM (1M). The mixture was stirred at r.t. overnight, then dissolved in EtOAc and water. Layers were separated and the aqueous one was extracted with EtOAc. Gathered organic layers were washed with brine, dried over Na_2SO_4 and evaporated to dryness to get crude material, which was used as obtained in next reaction.

Oxamate (1.0 eq.), DCM (0.3M) and TFA (5.0 eq.) were stirred at r.t. until completion of the reaction monitored by TLC (Chex/EtOAc 90:10). Solvent and excess of acid were removed by evaporation and the crude mixture was purified by reprecipitation from DCM/PE 1:10 to afford pure compound.

Following oxamic acids were synthesized following procedure A and analytical data were identical to those previously reported[2,3,4,5,6,7,8,9,10,11]. New oxamic acids are described below.

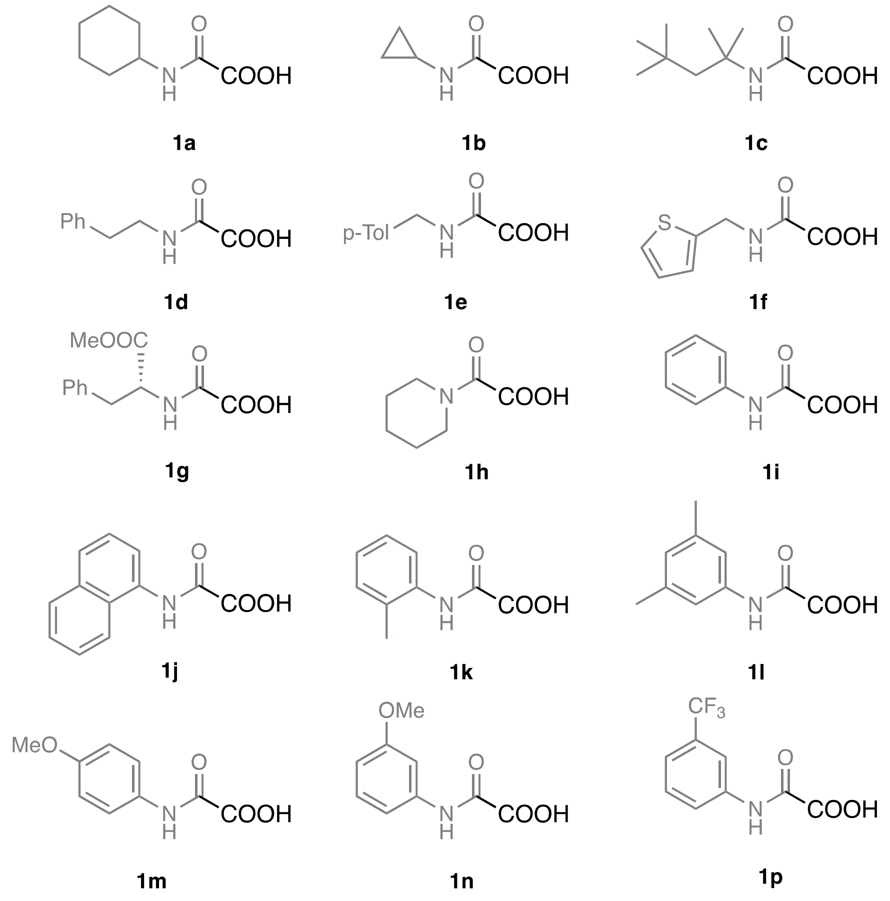

1a[2], **1b**[3], **1c**[4], **1d**[5], **1e**[6], **1f**[7], **1g**[8], **1h**[3], **1i**[9], **1j**[10], **1k**[9], **1l**[11], **1m**[9], **1n**[9], **1p**[5]

Figure 1: Previously described oxamic acids

***N*-(2-methoxyphenyl)oxamic acid**

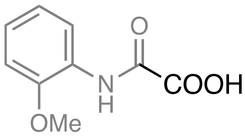

1o

Following general procedure A, **1o** was obtained from *tert*-butyl (2-methoxyphenyl)oxamate (4.0 g, 15.9 mmol, 1.0 eq.) as a fine white crystalline powder (2.57 g, 83%).

^{1}H NMR ($CDCl_3$, 300 MHz) : δ_H 9.62 (1H, br s), 8.32-8.29 (1H, dd, *J* = 8.0 Hz, *J* = 1.6 Hz), 7.21-7.16 (1H, ddd, *J* = 8.1 Hz, *J* = 7.6 Hz, *J* = 1.6 Hz), 7.04-7.98 (1H, ddd, *J* = 7.9 Hz, *J*= 7.6 Hz, *J* = 1.1 Hz), 6.98-6.92 (1H, dd, *J* = 8.2 Hz, *J* = 1.3 Hz), 3.93 (3H, s).

^{13}C NMR ($CDCL_3$, 75 MHz) : δ_C 160.2, 154.8, 149.0, 126.4, 125.3, 121.2, 120.1, 110.5, 56.0.

FTIR (film, KBr): ν (cm^{-1}) = 3384, 3240, 1688, 1598, 1547, 1487, 1465, 1435, 1356, 1333, 1291, 1252, 1197, 1177, 1161, 1115, 1042, 1027, 782, 761, 734, 698.

HRMS (TOF/ESI$^-$) Calculated for $C_9H_8O_4N$ [M-H]$^-$ 194.04588 found 194.04548.

m.p. 151°C (DCM/PE 1:10)

N-(4-trifluoromethylphenyl)oxamic acid

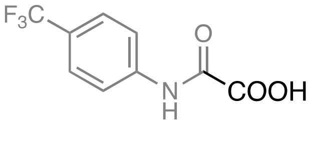

1q

Following general procedure A, **1q** was obtained from *tert*-butyl (4-trifluoromethylphenyl)oxamate (4.0 g, 13.8 mmol, 1.0 eq.) as a fine white crystalline powder (2.98 g, 92%).

^{1}H NMR ($CDCl_3$, 300 MHz) : δ_H 9.11 (1H, br s), 7.79-7.77 (2H, m), 7.69-7.67 (2H, m).

^{19}F NMR (Acetone d$_6$, 282 MHz) : δ_F -62.7

^{13}C NMR (Acetone d$_6$, 75 MHz) : δ_C 206.6, 161.5 (d, J_{C-F} = 2.8 Hz), 156.9 (q, J_{C-F} = 5.9 Hz), 141.8 (d, J_{C-F} = 7.6 Hz), 127.0 (q, J_{C-F} = 4.0 Hz), 126.9 (q, J_{C-F}= 32 Hz), 126.2 (q, J_{C-F} = 272 Hz), 121.1 (d, J_{C-F} = 5.9 Hz).

FTIR (film, KBr): ν (cm^{-1}) = 3307, 1757, 1687, 1619, 1605, 1551, 1415, 1333, 1312, 1254, 1210, 1167, 1126, 111, 1070, 1018, 934, 841.

HRMS (TOF/ESI$^-$) Calculated for $C_9H_5O_3NF_3$ [M-H]$^-$ 232.02270, found 232.02227.

m.p. 181°C (DCM/PE 1:10)

N-(4-ethoxycarbonylphenyl)oxamic acid

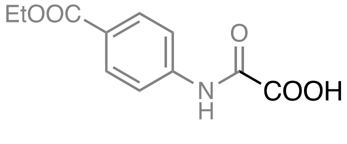

1r

Following general procedure A, **1r** was obtained from *tert*-butyl (4-ethoxycarbonylphenyl)oxamate (5.0 g, 16.3 mmol, 1.0 eq.) as a fine white crystalline powder (3.56 g, 92%).

^{1}H NMR (Acetone d$_6$, 300 MHz) : δ_H 10.22 (1H, br s), 8.05-7.97 (4H, m), 5.55 (1H, br s), 4.37 (2H, q, *J*= 7.1 Hz), 1.38-1.33 (3H, t, *J*= 7.1 Hz).

^{13}C NMR ($CDCL_3$, 75 MHz) : δ_C 206.3, 166.2, 161.6, 156.9, 142.5, 131.2, 127.6, 120.5, 120.4, 61.4, 14.6.

FTIR (film, KBr): ν (cm^{-1}) = 3499, 3434, 3318, 17171696, 1605, 1542, 1284, 1174, 1112, 1018, 863, 769, 747, 726.

HRMS (TOF/ESI$^-$) Calculated for $C_{11}H_{10}O_5N$ [M-H]$^-$ 236.05645 found 236.05610.

m.p. 182°C (DCM/PE 1:10)

3. Synthesis of imines as starting materials

PROCEDURE B:

All imines were synthesized according to a previously reported procedure[12].

Aniline (1 eq.) and aldehyde (1 eq.) were refluxed into ethanol (0.2M) overnight. Mixture was cooled down to r.t. The obtained precipitate was filtered and washed with EtOAc and the crude mixture was recrystallized from appropriate solvent to get pure awaited compound.

N-[(4-methoxyphenyl)methylene]-4-(trifluoromethyl)benzenamine

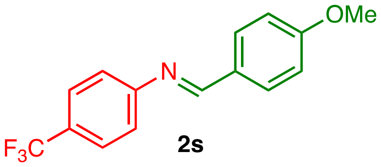

2s

Following the general procedure B, **2a** was obtained from anisaldehyde (845 mg, 6.2 mmol, 1.0 eq.) and (4-trifluoromethyl)aniline (1.000 g, 6.2 mmol, 1.0 eq.) as a pale yellow powder (1.43, 5.14 mmol, 83%), after recrystallisation from diisopropylether.

^{1}H NMR ($CDCl_3$, 300 MHz) : δ_H 8.35 (1H, s), 7.88-7.85 (2H, m), 7.65-7.62 (2H, m), 7.25-7.22 (2H, m), 7.02-6.99 (2H, m), 3.89 (3H, s). Analytical data were identical with those previously reported[12].

N-[(3-fluoro)phenylmethylene]-4-(cyano)benzenamine

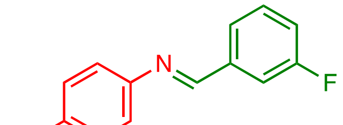

2t

Following the general procedure B, **2t** was obtained from 3-fluorobenzaldehyde (1.000 g, 8.1 mmol, 1.0 eq.) and 4-aminobenzonitrile (952 mg, 8.1 mmol, 1.0 eq.) as a pale yellow powder (0.89 g, 4.05 mmol, 50%) after recrystallization from EtOAc then diisopropylether.

Analytical data were identical with those previously reported[13].

Methyl 4-[[(3,4-dibromophenyl)imino]methyl]benzoate

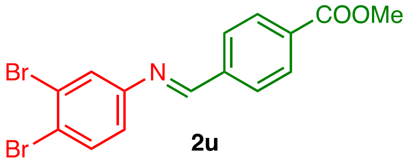

2u

Following the general procedure B, **2u** was obtained from 3,4-dibromoaniline (1.53 g, 6.1 mmol, 1.0 eq.) and methyl 4-formylbenzoate (1.00 g, 6.1 mmol, 1.0 eq.) as a pale yellow crystal (1.89 g, 4.8 mmol, 79%, 91% pure) after recrystallization from EtOAc then diisopropylether.

^{1}H NMR ($CDCl_3$, 300 MHz) : δ_H 8.47 (1H, s), 8.16-8.13 (2H, m), 7.98-7.94 (2H, m), 7.65-7.62 (1H, d, *J* = 8.5 Hz), 7.50-7.49 (d, *J* = 2.4 Hz), 7.07-7.04 (1H, dd, *J* = 8.4 Hz, *J* = 2.4 Hz).

^{13}C NMR ($CDCL_3$, 75 MHz) : δ_C 166.4, 160.3, 151.5, 139.3, 134.0, 132.9, 130.1, 128.9, 125.8, 125.2, 122.1, 121.6, 52.4.

FTIR (film, KBr): ν (cm^{-1}) = 2994, 2949, 2886, 1720, 1627, 1568, 1547, 1454, 1435, 1412, 1358, 1278, 1229, 1187, 1107, 1010, 899, 867, 855, 813, 767, 697.

HRMS (TOF/ESI) Calculated for $C_{15}H_{12}O_2NBr_2$ $[M+H]^+$ 395.92293 found 395.92166.

m.p. 127°C (diisopropylether).

Ethyl 4-[[(4-cyanophenyl)methylene]amino]benzoate

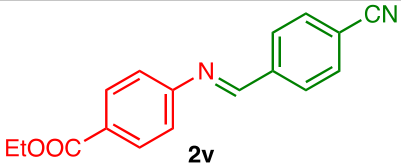

2v

2v was obtained from fusion of 4-cyanobenzaldehyde (794 mg, 6.1 mmol, 1.0 eq.) and benzocaine (1.000 g, 6.1 mmol, 1.0 eq.) and after a recrystallisation from Et_2O then diisopropylether as a pale yellow crystal (1.45g, 5.2 mmol, 86%, 94% pure).

^{1}H NMR ($CDCl_3$, 300 MHz) : δ_H 8.47 (1H, s), 8.16-8.13 (2H, m), 7.98-7.94 (2H, m), 7.65-7.62 (1H, m), 7.50-7.49 (1H, m), 7.07-7.04 (1H, m), 3.96 (3H, s).

Analytical data were identical with those previously reported[13].

^{1}H NMR ($CDCl_3$, 300 MHz) : δ_H 8.47 (1H, s), 8.10-8.06 (2H m), 8.03-8.00 (2H, m), 7.78-7.75 (2H, m), 7.23-7.20 (2H, m), 4.41-4.34 (2H, q, *J* = 7.1 Hz), 1.42-1.37 (3H, t, *J* = 7.1 Hz).

^{13}C NMR ($CDCL_3$, 75 MHz) : δ_C 166.2, 159.4, 155.1, 139.6, 132.6, 130.9, 129.3, 128.6, 120.6, 118.4, 115.0, 61.1, 14.4.

FTIR (film, KBr): ν (cm^{-1}) = 3053, 2992, 2909, 2224, 1703, 1599, 1284, 1199, 1170, 1126, 1106, 1025, 989, 896, 839, 774, 701, 557.

HRMS (TOF/ESI) Calculated for $C_{17}H_{15}O_2N_2$ $[M+H]^+$ 279.11280 found 279.11188.

m.p. 162°C (diisopropylether).

4. Mechanistic studies

a) Radical trapping experiment using TEMPO

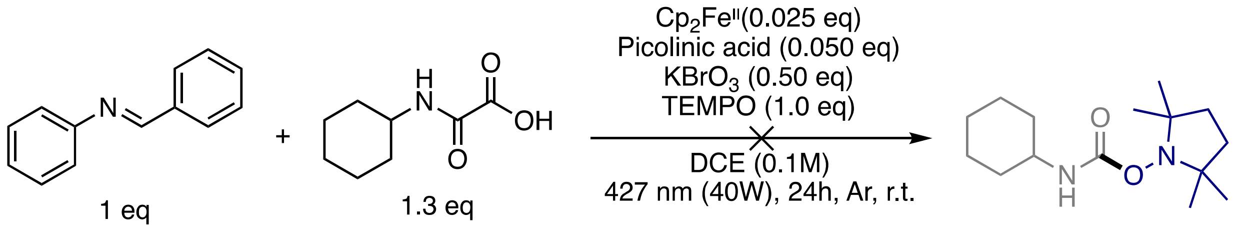

Following optimized conditions, reaction was performed adding TEMPO (1.0 eq.) as a radical trap. To a dried sealed Schlenk tube under Argon were charged oxamic acid (1 eq.), ferrocene (0.025 eq.), imine (1 eq.), picolinic acid (0.050 eq.), $KBrO_3$ (0.50 eq.), TEMPO (1.0 eq.). The tube was evacuated and refilled with Argon 3 times. Then distillated dried-solvent (0.1 M) was added. The mixture was purged with Argon. The tube was sealed and the mixture was irradiated with a Kessil lamp (40W, 427 nm, 5 cm away from the light source, cooled to r.t. with a fan) over 24h. Solvent was evaporated to dryness to get a crude mixture. A sample was analyzed by GC-MS and crude NMR was obtained using mesitylene as an external standard.

Only traces of **3a** were detected by GC-MS (5% NMR yield) suggesting that TEMPO interfered in the radical reaction process. Moreover, several by-products from isocyanate and carbamoyl radical were observed by GC-MS.

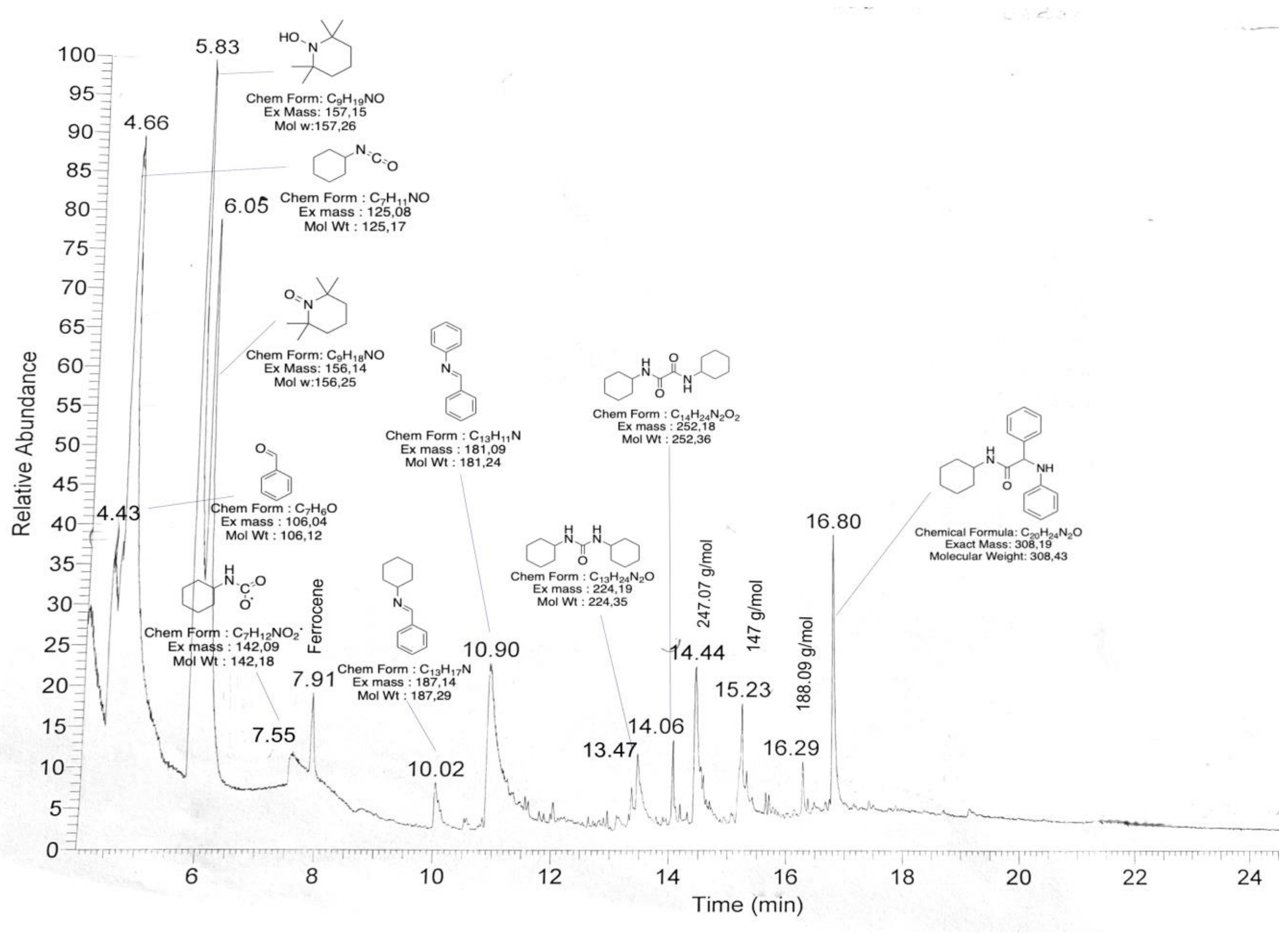

Figure 2: GC-MS of the reference reaction with TEMPO

b) Radical trapping experiment using 1,1-diphenylethylene

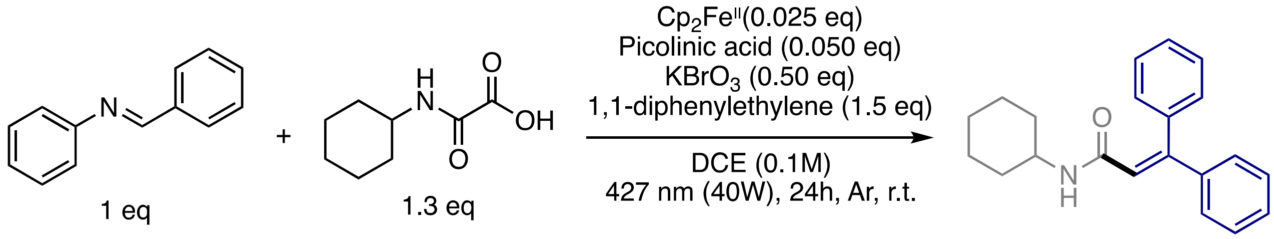

Following optimized conditions, reaction was performed adding 1,1-diphenylethylene (1.5 eq.) as a radical trap. To a dried sealed Schlenk tube under Argon were charged oxamic acid (1 eq.), ferrocene (0.025 eq.), imine (1 eq.), picolinic acid (0.050 eq.), $KBrO_3$ (0.50 eq.), 1,1-diphenylethylene (1.5 eq.). The tube was evacuated and refilled with Argon 3 times. Then distillated dried-solvent (0.1 M) was added. The mixture was purged with Argon. The tube was sealed and the mixture irradiated with a Kessil lamp (40W, 427 nm, 5 cm away from the light source, cooled to r.t. with a fan) over 24h. The solvent was evaporated to dryness to get the crude product. A sample was analyzed by GC-MS and the crude was purified by flash column chromatography on silica gel (3 cm x 10 cm, SiO_2 x 100: 35g) using Chex:EtOAc as a solvent system for elution.

Awaited *N*-(cyclohexyl)-2-diphenyl-2-ethenamine was isolated with a 33% yield and compound **3a** was isolated with a 29% yield.

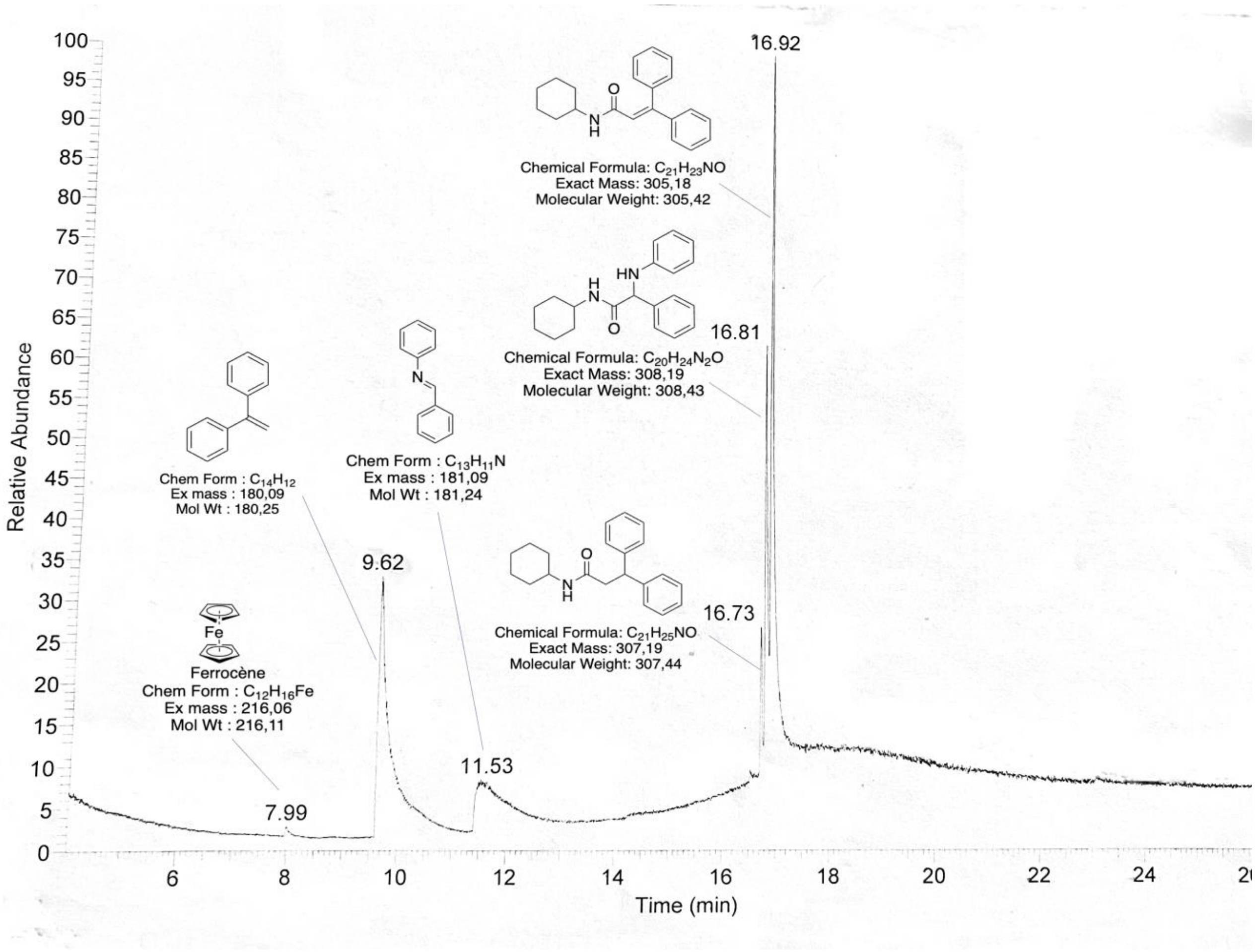

Figure 3: GC-MS of the reference reaction with 1,1-diphenylethylene

5. Optimization of the three-component reaction

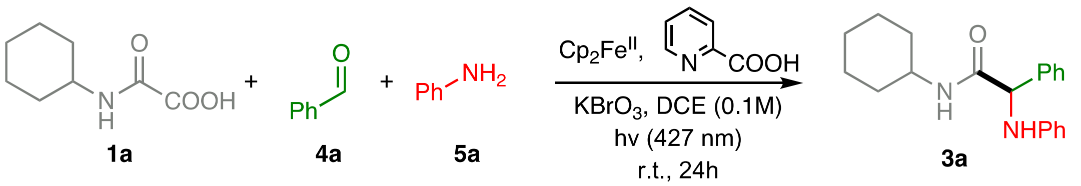

Oxamic acid (eq.)	Aldehyde (eq.)	Amine (eq.)	Oxidant (eq.)	Fe cat. (eq.)	Ligand (eq.)	Solvent (M)	Isolated yield
1.3	1.0	1.0	$KBrO_3$ (0.50)	0.025	0.050	DCE (0.1)	66%
1.0	1.0	1.0	$KBrO_3$ (0.50)	0.025	0.050	DCE (0.1)	66%
1.0	1.1	1.1	$KBrO_3$ (0.50)	0.025	0.050	DCE (0.1)	66%
1.0	1.2	1.2	$KBrO_3$ (0.50)	0.025	0.050	DCE (0.1)	69%
1.0	1.3	1.3	$KBrO_3$ (0.50)	0.025	0.050	DCE (0.1)	78%

6. Visible light mediated iron-catalyzed synthesis of α-amino acid amides

Procedure C: two components reaction

To a dried sealed Schlenk tube under Argon were charged oxamic acid (1 eq.), ferrocene (0.025 eq.), imine (1 eq.), picolinic acid (0.050 eq.) and $KBrO_3$ (0.50 eq.). The tube was evacuated and refilled with Argon 3 times. Then distillated dried-solvent (0.1 M) was added. The mixture was purged with Argon. The tube was sealed and the mixture irradiated with a Kessil lamp (40W, 427 nm, 5 cm away from the light source, cooled to r.t. with a fan) over 24h. Solvent was evaporated to dryness to get a crude mixture that was purified by flash column chromatography on silica gel (3 cm x 10 cm, SiO_2 x 100: 35g) using Chex:EtOAc as a solvent system for elution. If the compound was not pure enough, it was sonicated in pentane, filtered and dried. Then recrystallized from toluene, filtered and dried. Traces of toluene were removed by redissolving the compound twice in MeOH then $CHCl_3$.

Procedure D: three components reaction

To a dried sealed Schlenk tube under Argon were charged oxamic acid (1 eq.), ferrocene (0.025 eq.), picolinic acid (0.050 eq.), $KBrO_3$ (0.50 eq.) and aldehyde (1.0 eq.) and/or amine (1.0 eq.) if they were solids. The tube was evacuated and refilled with Argon 3 times. Then distillated dried-solvent (0.1 M) was added. The mixture was purged with Argon. Aldehyde (1.0 eq.) and amine (1.0 eq.) were finally added at this point if they were liquids. The tube was sealed and the mixture irradiated with a Kessil lamp (40W, 427 nm, 5 cm away from the light source, cooled to r.t. with a fan) over 24h. Solvent was evaporated to dryness to get a crude mixture that was purified by flash column chromatography on silica gel (3 cm x 10 cm, SiO_2 x 100: 35g) using Chex:EtOAc as a solvent system for elution. If the compound was not pure enough, it was sonicated in pentane, filtered and dried. Then recrystallized from toluene, filtered and dried. Traces of toluene were removed by redissolving the compound twice in MeOH then $CHCl_3$.

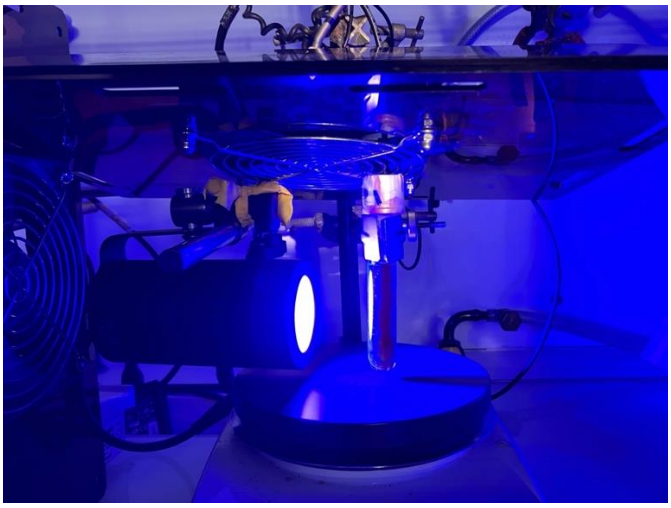

Figure 4: Set up used for the photocatalysis

7. Unsuccessful examples and rationalization of some low yields

Aliphatic aldehydes, aliphatic amines, benzylamine and benzenesulfonamide were found not to react properly in the reaction conditions as they lead to the formation of unstable imines at room temperature, which could undergo further condensations[14].

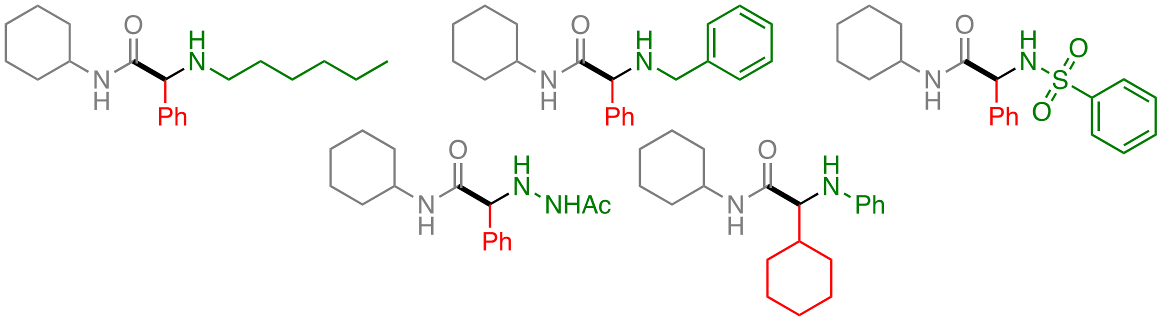

Figure 5: Unsuccessful examples

Lower yields were observed in several cases. For instance, compound **3y** bearing a *N,N*-dimethylamino substituent on the aromatic is prone to oxidation[15] and was only detected by ^{1}H NMR and not isolated. Low yield for **3s**, **3ag**, **3ak** highlight some complex electronic effects of diphenylimine substituents, which could influence the reaction at different steps : imine formation, imine reactivity toward the carbamoyl radical, and the stability of corresponding aminyl radical V[13,16,17]. Notably, the *in-situ* generation of corresponding imine **2ag** is not easy under the reaction conditions, with a 49% yield reported in the literature after 7h at reflux in toluene[18].

8. Characterization of products

N-(Cyclohexyl)-α-(phenylamino)benzeneacetamide

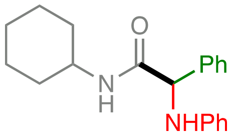

3a

Following the general procedure C, **3a** was obtained from *N*-(cyclohexyl)oxamic acid (100 mg, 0.58 mmol, 1.0 eq.) and diphenylimine (100 mg, 0.58 mmol, 1.0 eq.) as a white solid (127 mg, 0.41 mmol, 70%) after purification by flash column chromatography on silica gel (Chex/EtOAc 95:5) (Rf = 0.16, Chex/EtOAc 90:10).

Following the general procedure D, **3a** was obtained from *N*-(cyclohexyl)oxamic acid (100 mg, 0.58 mmol, 1.0 eq.), benzaldehyde (62 mg, 0.58 mmol, 1.0 eq.) and aniline (54 mg,

0.58 mmol, 1.0 eq.) as a white solid (119 mg, 0.39 mmol, 66%) after purification by flash column chromatography on silica gel (Chex/EtOAc 95:5) (Rf = 0.16, Chex/EtOAc 90:10).

^{1}H NMR ($CDCl_3$, 300 MHz) : δ_H 7.43-7.35 (5H, m), 7.22-7.16 (2H, m), 6.83-6.78 (1H, m), 6.65-6.62 (2H, m), 6.58 (1H, br s), 4.70 (1H, s), 4.47 (1H, br s), 3.87-3.75 (1H, m), 1.91-1.77 (2H, m), 1.68-1.57 (3H, m), 1.38-126 (2H, m), 1.18-1.04 (3H, m).

^{13}C NMR ($CDCl_3$, 75 MHz) : δ_C 170.1, 146.9, 139.2, 129.4, 129.3, 128.6, 127.5, 119.2, 114.0, 64,5, 48.2, 33.1, 32.9, 25.6, 24.9, 24.8.

FTIR (film, KBr): ν (cm^{-1}) = 3303, 3055, 2931, 2854, 1647, 1602, 1504, 1452, 1430, 1350, 1316, 1261, 749, 693.

HRMS (TOF/ESI$^-$) Calculated for $C_{20}H_{23}ON_2$ $[M-H]^-$ 307.18159, found 307.18143.

m.p. 174-176°C (toluene).

***N*-(Cyclopropyl)-α-(phenylamino)benzeneacetamide**

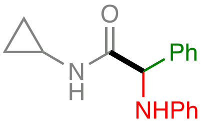

3b

Following the general procedure C, **3b** was obtained from *N*-(cyclopropyl)oxamic acid (71 mg, 0.55 mmol, 1.0 eq.) and diphenylimine (100 mg, 0.55 mmol, 1.0 eq.) as a white solid (112 mg, 0.42 mmol, 72%) after purification by flash column chromatography on silica gel (Chex/EtOAc 85:15) (Rf = 0.15, Chex/EtOAc 80:20).

^{1}H NMR ($CDCl_3$, 300 MHz) : δ_H 7.43-7.33 (5H, m), 7.21-7.16 (2H, m), 6.85 (1H, br s), 6.83-6.78 (1H, m), 6.63-6.60 (2H, m), 4.72 (1H, s), 4.58 (1H, br s), 2.76-2.68 (1H, m), 0.77-0.71 (2H, m), 0.46-0.42 (2H, m).

^{13}C NMR ($CDCl_3$, 75 MHz) : δ_C 172.7, 146.7, 138.8, 129.4, 129.2, 128.6, 127.4, 119.1, 113.8, 64.0, 22.7, 6.7, 6.4.

FTIR (film, KBr): ν (cm^{-1}) = 3293, 3052, 3031, 2924, 2854, 1654, 1602, 1542, 1504, 1454, 1428, 1361, 1316, 1265, 1181, 1028, 750, 733, 694.

HRMS (TOF/ESI$^-$) Calculated for $C_{17}H_{17}ON_2$ $[M-H]^-$ 265.13464, found 265.13443.

m.p. 136°C (toluene).

***N*-(1,1,3,3-Tetramethylbutyl)-α-(phenylamino)benzeneacetamide**

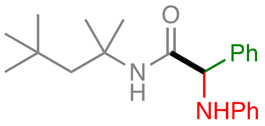

3c

Following the general procedure C, **3c** was obtained from *N*-(1,1,3,3-tetramethylbutyl)oxamic acid (111 mg, 0.55 mmol, 1.0 eq.) and diphenylimine (100 mg, 0.55 mmol, 1.0 eq.) as a white solid (112 mg, 0.42 mmol, 72%) after purification by flash column chromatography on silica gel (Chex/EtOAc 98:2) (Rf = 0.22, Chex/EtOAc 95:5).

^{1}H NMR ($CDCl_3$, 300 MHz) : δ_H 7.45-7.33 (5H, m), 7.20-7.14 (dd, *J* = 8.5 Hz, *J* = 7.3 Hz), 6.81-6.75 (1H, tt, *J* = 7.3 Hz, *J* = 1.1 Hz), 6.64-6.61 (2H, dd, *J* = 8.6 Hz, *J* = 1.1 Hz), 6.47 (1H, br s), 4.69 (1H, br s), 4.62 (1H, s), 1.63-1.60 (2H, d, *J* = 9.7 Hz), 1.40-1.39 (6H, d, *J* = 3.5 Hz), 0.87 (9H, s).

^{13}C NMR ($CDCl_3$, 75 MHz) : δ_C 169.8, 146.9, 139.4, 129.3,128.5, 127.4, 119.0, 114.0, 64.7, 55.4, 53.2, 31.6, 31.5, 28.8, 28.2.

FTIR (film, KBr): ν (cm^{-1}) = 3344, 3054, 3031, 2954, 2925, 2855, 1661, 1603, 1505, 1482, 1454, 1365, 1315, 1260, 1226, 749, 694.

HRMS (TOF/ESI$^-$) Calculated for $C_{22}H_{29}ON_2$ $[M-H]^-$ 337.22654, found 337. 22861

m.p. 67°C (toluene)

N-(2-Phenethyl)-α-(phenylamino)benzeneacetamide

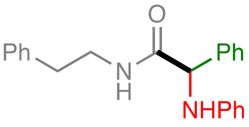

3d

Following the general procedure C, **3d** was obtained from *N*-(2-phenethyl)oxamic acid (107 mg, 0.55 mmol, 1.0 eq.) and diphenylimine (100 mg, 0.55 mmol, 1.0 eq.) as a brown oil (145 mg, 0.44 mmol, 79%) after purification by flash column chromatography on silica gel (Chex/EtOAc 95:5 to 90:10) (Rf = 0.08, Chex/EtOAc 90:10).

^{1}H NMR ($CDCl_3$, 300 MHz) : δ_H 7.36 (5H, s), 7.24-7.19 (5H, m), 7.02-7.19 (2H, m), 6.86-6.81 (1H, m), 6.76 (1H, br s), 6.64-6.62 (2H, m), 4.73 (1H, s), 4.57 (1H, br s), 3.68-3.43 (2H, m), 2.86-2.65 (2H, m).

^{13}C NMR ($CDCl_3$, 75 MHz) : δ_C 171.1, 146.6, 138.8, 138.6, 129.3, 129.2, 128.8, 128.6, 128.5, 127.4, 126.4, 119.0, 113.8, 63.9, 40.6, 35.6.

FTIR (film, KBr): ν (cm^{-1}) = 3316, 3060, 3028, 2925, 2854, 1657, 1602, 1503, 1454, 1431, 1315, 1264, 750, 696.

HRMS (TOF/ESI-) Calculated for $C_{22}H_{21}ON_2$ $[M-H]^-$ 329.16594, found 329.16592.

N-(4-Methylbenzyl)-α-(phenylamino)benzeneacetamide

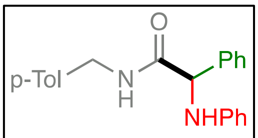

3e

Following the general procedure C, **3e** was obtained from *N*-(4-methylbenzyl)oxamic acid (107 mg, 0.55 mmol, 1.0 eq.) and diphenylimine (100 mg, 0.55 mmol, 1.0 eq.) as a white solid (131 mg, 0.40 mmol, 72%) after purification by flash column chromatography on silica gel (Chex/EtOAc 95:5) (Rf = 0.12, Chex/EtOAc 90:10).

^{1}H NMR ($CDCl_3$, 300 MHz) : δ_H 7.49-7.46 (2H, m), 7.44-7.39 (3H, m), 7.24-7.22 (2H, m), 7.12-7.04 (4H, m), 7.00 (1H, br s), 6.86-6.82 (1H, m), 6.68-6.65 (2H, m), 4.83 (1H, s), 4.60 (1H, br s), 4.57-4.50 (1H, dd, *J* = 14.8 Hz, *J* = 5.5 Hz), 4.39-4.33 (1H, dd, *J* = 14.8 Hz, *J* = 5.5 Hz), 2.34 (3H, s).

^{13}C NMR ($CDCl_3$, 75 MHz) : δ_C 171.1, 146.6, 138.9, 137.2, 134.9, 129.4, 129.3, 129.3, 128.6, 127.6, 127.4, 119.2, 113.9, 64.2, 43.3, 21.1.

FTIR (film, KBr): ν (cm^{-1}) = 3307, 3052, 3028, 2924, 2854, 1654, 1602, 1504, 1454, 1429, 1316, 1264, 1180, 1028, 805, 749, 694.

HRMS (TOF/ESI$^-$) Calculated for $C_{22}H_{21}ON_2$ $[M-H]^-$ 329.16594, found 329.16599.

m.p. 128°C (toluene).

N-(2-Methylthienyl)-α-(phenylamino)benzeneacetamide

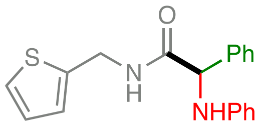

3f

Following the general procedure C, **3f** was obtained from *N*-(2-methylthienyl)oxamic acid (102 mg, 0.55 mmol, 1.0 eq.) and diphenylimine (100 mg, 0.55 mmol, 1.0 eq.) as a white solid (98 mg, 0.30 mmol, 55%) after purification by flash column chromatography on silica gel (Chex/EtOAc 95:5) (Rf = 0.06, Chex/EtOAc 95:5).

^{1}H NMR ($CDCl_3$, 300 MHz) : δ_H 7.46-7.36 (5H, m), 7.22-7.17 (4H, m), 6.92-6.89-6.80 (3H, m), 6.65-6.62 (2H, m), 4.80 (1H, s), 4.74-4.49 (3H, m).

^{13}C NMR ($CDCl_3$, 75 MHz) : δ_C 171.1, 146.6, 140.7, 138.7, 129.4, 129.3, 128.7, 127.5, 126.9, 125.9, 125.2, 119.2, 113.9, 64.2, 38.4.

FTIR (film, KBr): ν (cm^{-1}) = 3374, 3308, 3055, 2924, 2854, 1657, 1602, 1504, 1454, 1428, 1316, 1265, 1225, 750, 694.

HRMS (TOF/ESI$^-$) Calculated for $C_{19}H_{17}ON_2S$ $[M-H]^-$ 321.10676, found 321.10660.

m.p. 136-138°C (toluene).

_N_-((_S_)-Phenylalaninyl)-α-(phenylamino)benzeneacetamide

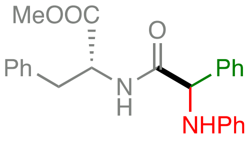

3g

Following the general procedure C, **3g** was obtained from _N_-((_S_)-phenylalaninyl)oxamic acid (139 mg, 0.55 mmol, 1.0 eq.) and diphenylimine (100 mg, 0.55 mmol, 1.0 eq.) as a yellow solid (88 mg, 0.23 mmol, 40%) after purification by flash column chromatography on silica gel (Chex/EtOAc 90:10) (Rf = 0.08, Chex/EtOAc 90:10).

^{1}H NMR ($CDCl_3$, 300 MHz) : δ_H 7.31-7.26 (5H, m), 7.21-7.16 (4H, m), 7.08-7.05 (3H, m), 6.83-6.78 (1H, m), 6.64-6.62 (2H, m), 4.92-4.85 (1H, m), 4.70 (1H, s), 4.41 (1H, br s), 3.64 (3H, s), 3.29-3.22 (1H, m), 3.04-2.97 (1H, m).

^{13}C NMR ($CDCl_3$, 75 MHz) : δ_C 171.5, 171.2, 146.7, 138.4, 136.1, 129.4, 129.3, 129.3, 128.8, 128.7, 127.4, 127.2, 119.4, 114.3, 64.6, 53.2, 52.4, 37.7.

FTIR (film, KBr): ν (cm^{-1}) = 3369, 3059, 3030, 2924, 2853, 1742, 1663, 1602, 1503, 1454, 1435, 1362, 1315, 1261, 1214, 1179, 750, 697.

HRMS (TOF/ESI$^-$) Calculated for $C_{24}H_{23}O_3N_2$ $[M-H]^-$ 387.17142, found 387.17108.

m.p. 137°C (toluene).

_N_-(1-Piperidinyl)-α-(phenylamino)benzeneacetamide

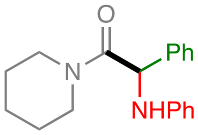

3h

Following the general procedure C, **3h** was obtained from _N_-(1-piperidinyl)oxamic acid (87 mg, 0.55 mmol, 1.0 eq.) and diphenylimine (100 mg, 0.55 mmol, 1.0 eq.) as a beige solid (62 mg, 0.21 mmol, 38%) after purification by flash column chromatography on silica gel (Chex/EtOAc 95:5) (Rf = 0.09, Chex/EtOAc 95:5).

^{1}H NMR ($CDCl_3$, 300 MHz) : δ_H 7.48-7.44 (2H, m), 7.36-7.32 (2H, m), 7.28-7.26 (1H, m, 7.15-7.09 (2H, m), 6.69-6.64 (3H, m), 3.68 (1H, m), 3.49-3.46 (3H, m), 1.57-1.40 (5H, m), 1.07-1.02 (1H, m).

^{13}C NMR ($CDCl_3$, 75 MHz) : δ_C 169.0, 146.6, 139.0, 129.3, 129.0, 128.0, 127.8, 117.7, 113.6, 58.0, 46.6, 43.8, 25.9, 25.5, 24.5.

FTIR (film, KBr): ν (cm^{-1}) = 3380, 3025, 2925, 2854, 1639, 1602, 1581, 1499, 1455, 1424, 1316, 1256, 1178, 1130, 1119, 1013, 748, 710, 693.

HRMS (TOF/ESI$^+$) Calculated for $C_{19}H_{23}ON_2$ $[M+H]^+$ 295.18049 found 295.18015

m.p. 165°C (toluene).

_N_-(Phenyl)-α-(phenylamino)benzeneacetamide

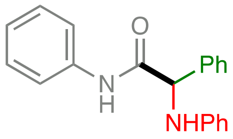

3i

Following the general procedure C, **3i** was obtained from _N_-(phenyl)oxamic acid (91 mg, 0.55 mmol, 1.0 eq.) and diphenylimine (100 mg, 0.55 mmol, 1.0 eq.) as a beige solid (166 mg, 0.55 mmol, 99%) after purification by flash column chromatography on silica gel (Chex/EtOAc 95:5) (Rf = 0.25, Chex/EtOAc 90:10).

^{1}H NMR ($CDCl_3$, 300 MHz) : δ_H 8.78 (1H, br s), 7.55-7.49 (4H, m),7.44-7.37 (3H, m), 7.33-7.28 (2H, m), 7.26-7.21 (2H, m), 7.15-7.09 (1H, m), 6.90-6.85 (1H, m), 6.75-6.72 (2H, m), 4.85 (1H, s), 4.51 (1H, br s).

^{13}C NMR ($CDCl_3$, 75 MHz) : δ_C 169.7, 146.6, 138.4, 137.4, 129.6, 129.5, 129.1, 128.9, 127.5, 124.7, 120.1, 119.9, 114.3, 65.5.

FTIR (film, KBr): ν (cm^{-1}) = 3321, 3204, 3141, 3055, 2882, 1669, 1601, 1516, 1505, 1496, 1443, 1315, 1265, 1246, 1177, 1157, 1128, 1078, 1030, 748, 693, 500.

HRMS (TOF/ESI$^-$) Calculated for $C_{20}H_{17}ON_2$ $[M-H]^-$ 301.13464, found 301.13437

m.p. 136-139°C (toluene)

N-(1-Naphtalenyl)-α-(phenylamino)benzeneacetamide

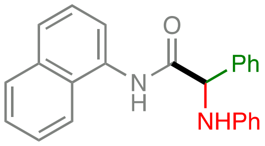

3j

Following the general procedure C, **3j** was obtained from *N*-(1-naphtalenyl)oxamic acid (125 mg, 0.55 mmol, 1.0 eq.) and diphenylimine (100 mg, 0.55 mmol, 1.0 eq.) as a beige solid (166 mg, 0.55 mmol, 99%) after purification by flash column chromatography on silica gel (Chex/EtOAc 92:8) (Rf = 0.15, Chex/EtOAc 90:10).

^{1}H NMR ($CDCl_3$, 300 MHz) : δ_H 9.27 (1H, s), 8.02-7.99 (1H, m), 7.85-7.82 (1H, m), 7.69-7.66 (1H, m), 7.62-7.58 (2H, m), 7.48-7.32 (7H, m), 7.27-7.24 (2H, m), 6.93-6.88 (1H, s), 6.85-6.81 (2H, m), 5.0 (1H, s), 4.69 (1H, br s).

^{13}C NMR ($CDCl_3$, 75 MHz) : δ_C 170.0, 146.5, 138.7, 134.1, 131.8, 129.7, 129.6, 129.0, 128.8, 127.7, 127.6, 127.0, 126.5, 126.1, 125.8, 120.3, 120.2, 120.1, 114.4, 65.4.

FTIR (film, KBr): ν (cm^{-1}) = 3299, 3052, 1662, 1601, 1503, 1454, 1431, 1399, 1347, 1315, 1266, 1180, 792, 770, 750, 694.

HRMS (TOF/ESI$^-$) Calculated for $C_{24}H_{19}ON_2$ [M-H]$^-$ 351.15029, found 351.15015.

m.p. 173-175°C (toluene).

N-(2-Methylphenyl)-α-(phenylamino)benzeneacetamide

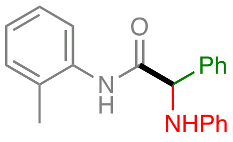

3k

Following the general procedure C, **3k** was obtained from *N*-(2-methylphenyl)oxamic acid (99 mg, 0.55 mmol, 1.0 eq.) and diphenylimine (100 mg, 0.55 mmol, 1.0 eq.) as a beige solid (149 mg, 0.47 mmol, 85%) after purification by flash column chromatography on silica gel (Chex/EtOAc 95:5) (Rf = 0.18, Chex/EtOAc 90:10).

^{1}H NMR ($CDCl_3$, 300 MHz) : δ_H 8.72 (1H, s), 7.93-7.90 (1H, m), 7.57-7.55 (2H, m), 7.44-7.42 (3H, m), 7.28-7.19 (3H, m), 7.16-7.14 (1H, m), 7.10-7.05 (1H, m), 6.91-6.86 (1H, m), 6.78-6.75 (2H, m), 4.91 (1H, s), 4.68 (1H, br s), 2.03 (3H, s).

^{13}C NMR ($CDCl_3$, 75 MHz) : δ_C 169.4, 146.5, 138.6, 135.2, 130.5, 129.5, 129.4, 128.9, 127.5, 126.8, 125.2, 122.4, 119.7, 114.2, 65.1, 17.3.

FTIR (film, KBr): ν (cm^{-1}) = 3329, 3053, 3030, 2923, 1667, 1603, 1588, 1503, 1455, 1430, 1314, 1260, 1180, 751, 694.

HRMS (TOF/ESI$^-$) Calculated for $C_{21}H_{19}ON_2$ [M-H]$^-$ 315.15029, found 315.15005.

m.p. 92-94°C (toluene).

N-(3,5-Dimethylphenyl)-α-(phenylamino)benzeneacetamide

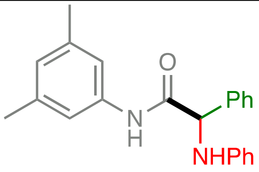

3l

Following the general procedure C, **3l** was obtained from *N*-(3,5-dimethylphenyl)oxamic acid (107 mg, 0.55 mmol, 1.0 eq.) and diphenylimine (100 mg, 0.55 mmol, 1.0 eq.) as a beige solid (174 mg, 0.53 mmol, 95%) after purification by flash column chromatography on silica gel (Chex/EtOAc 95:5) (Rf = 0.22, Chex/EtOAc 90:10).

^{1}H NMR ($CDCl_3$, 300 MHz) : δ_H 8.66 (1H, s), 7.52-7.49 (2H, m), 7.43-7.37 (3H, m), 7.26-7.21 (2H, m), 7.19 (2H, s), 6.90-6.85 (1H, m), 6.77 (1H, s), 6.74-6.71 (2H, m), 4.83 (1H, s), 4.50 (1H, br s), 2.28 (6H, s).

^{13}C NMR ($CDCl_3$, 75 MHz) : δ_C 169.5, 146.6, 138.8, 138.6, 137.2, 129.5, 129.4, 128.8, 127.4, 126.4, 119.8, 117.6, 114.2, 65.5, 21.3.

FTIR (film, KBr): ν (cm^{-1}) = 3315, 3052, 3030, 2919, 2858, 1664, 1602, 1538, 1496, 11454, 1427, 1317, 1265, 1215, 1181, 844, 750, 692, 508.

HRMS (TOF/ESI$^-$) Calculated for $C_{22}H_{21}ON_2$ [M-H]$^-$ 329.16594, found 329.16589.

m.p. 116-118°C (toluene).

N-(4-Dimethoxyphenyl)-α-(phenylamino)benzeneacetamide

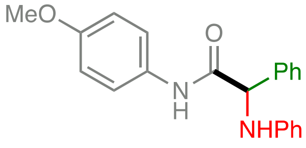

3m

Following the general procedure C, **3m** was obtained from *N*-(4-dimethoxyphenyl)oxamic acid (108 mg, 0.55 mmol, 1.0 eq.) and diphenylimine (100 mg, 0.55 mmol, 1.0 eq.) as a beige solid (106 mg, 0.32 mmol, 58%) after purification by flash column chromatography on silica gel (Chex/EtOAc 95:5) (Rf = 0.09, Chex/EtOAc 90:10).

^{1}H NMR ($CDCl_3$, 300 MHz) : δ_H 8.67 (1H, br s), 7.51-7.48 (2H, m), 7.43-7.36 (5H, m), 7.26-7.20 (2H, m), 6.89-6.81 (3H, m), 6.74-6.71 (2H, m), 4.84 (1H, s), 3.77 (3H, s).

^{13}C NMR ($CDCl_3$, 75 MHz) : δ_C 169.5, 156.8, 146.6, 138.7, 130.5, 129.6, 129.4, 128.9, 127.5, 121.9, 119.8, 114.2, 114.2, 65.2, 55.6.

FTIR (film, KBr): ν (cm^{-1}) = 3316, 3054, 2956, 2933, 2836, 1662, 1602, 1511, 1464, 1454, 1441, 1413, 1314, 1301, 1245, 1174, 1032, 828, 797, 751, 895, 514.

HRMS (TOF/ESI$^-$) Calculated for $C_{21}H_{19}O_2N_2$ [M-H]$^-$ 331.14410 found 331.14523.

m.p. 149°C (toluene).

N-(3-Dimethoxyphenyl)-α-(phenylamino)benzeneacetamide

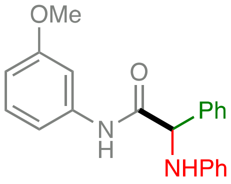

3n

Following the general procedure C, **3n** was obtained from *N*-(3-dimethoxyphenyl)oxamic acid (108 mg, 0.55 mmol, 1.0 eq.) and diphenylimine (100 mg, 0.55 mmol, 1.0 eq.) as a beige solid (90 mg, 0.27 mmol, 49%) after purification by flash column chromatography on silica gel (Chex/EtOAc 95:5) (Rf = 0.15, Chex/EtOAc 90:10).

^{1}H NMR ($CDCl_3$, 300 MHz) : δ_H 8.77 (1H, br s), 7.51-7.48 (2H, m), 7.44-7.34 (4H, m), 7.26-7.16 (3H, m), 6.99-6.96 (1H, m), 6.90-6.85 (1H, m), 6.74-6.71 (2H, m), 6.69-6.66 (1H, m), 4.83 (1H, s), 4.50 (1H, br s), 3.78 (3H, s).

^{13}C NMR ($CDCl_3$, 75 MHz) : δ_C 169.7, 160.3, 146.5, 138.5, 129.8, 129.6, 129.5, 129.0, 127.5, 120.0, 114.3, 112.1, 110.8, 105.6, 65.6, 55.4.

FTIR (film, KBr): ν (cm^{-1}) = 3323, 3054, 2933, 2836, 1667, 1603, 1527, 1496, 1454, 1427, 1314, 1288, 1264, 1217, 1179, 1156, 1043, 858, 774, 751, 732, 692.

HRMS (TOF/ESI$^-$) Calculated for $C_{21}H_{19}O_2N_2$ [M-H]$^-$ 331.14520, found 331.14496.

m.p. 127-129 °C (toluene).

N-(2-Dimethoxyphenyl)-α-(phenylamino)benzeneacetamide

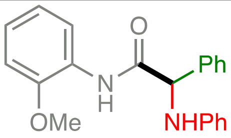

3o

Following the general procedure C, **3o** was obtained from *N*-(2-dimethoxyphenyl)oxamic acid (108 mg, 0.55 mmol, 1.0 eq.) and diphenylimine (100 mg, 0.55 mmol, 1.0 eq.) as a beige solid (116 mg, 0.35 mmol, 63%) after purification by flash column chromatography on silica gel (Chex/EtOAc 95:5) (Rf = 0.24, Chex/EtOAc 90:10).

^{1}H NMR ($CDCl_3$, 300 MHz) : δ_H 9.13 (1H, br s), 8.41-8.37 (1H, m), 7.57-7.53 (2H, m), 7.42-7.39 (3H, m), 7.24-7.19 (2H, m), 7.06-7.03 (1H, m), 7.00-6.97 (1H, m), 6.86-6.83 (2H, m), 6.75-6.72 (2H, m), 4.91 (1H, s), 4.70 (1H, br s), 3.73 (3H, s).

^{13}C NMR ($CDCl_3$, 75 MHz) : δ_C 169.3, 148.5, 146.7, 138.8, 129.4, 129.4, 128.8, 127.6, 127.3, 124.3, 121.2, 120.0, 119.3, 114.2, 110.4, 65.7, 56.0.

FTIR (film, KBr): ν (cm^{-1}) = 3350, 3054, 3031, 2937, 2839, 1677, 1601, 1523, 1505, 1462, 1433, 1314, 1290, 1253, 1218, 1176, 1117, 1047, 1027, 750, 695.

HRMS (TOF/ESI$^-$) Calculated for $C_{21}H_{19}O_2N_2$ [M-H]$^-$ 331.14520, found 331.14504.

m.p. 154°C (toluene).

***N*-(3-Trifluoromethylphenyl)-α-(phenylamino)benzeneacetamide**

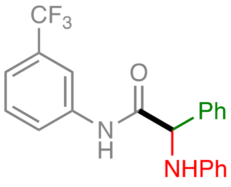

3p

Following the general procedure C, **3p** was obtained from *N*-(3-trifluoromethylphenyl)oxamic acid (129 mg, 0.55 mmol, 1.0 eq.) and diphenylimine (100 mg, 0.55 mmol, 1.0 eq.) as an orange oil (203 mg, 0.55 mmol, 99%) after purification by flash column chromatography on silica gel (Chex/EtOAc 95:5) (Rf = 0.24, Chex/EtOAc 90:10).

^{1}H NMR ($CDCl_3$, 300 MHz) : δ_H 8.99 (1H, br s), 7.86 (1H, s), 7.75-7.72 (1H, m), 7.51-7.48 (2H, m), 7.45-7.35 (5H, m), 7.28-7.23 (2H, m), 6.92-6.88 (1H, m), 6.75-6.73 (2H, m), 4.86 (1H, s), 4.50 (1H, br s).

^{19}F NMR ($CDCl_3$, 272 MHz): δ_F -62.7.

^{13}C NMR ($CDCl_3$, 75 MHz) : δ_C 170.1, 146.4, 138.1, 137.8, 131.7 (q, $J_{C\text{-}F}$ = 33 Hz), 129.7, 129.6, 129.5, 127.4, 123.8 (q, $J_{C\text{-}F}$ = 272 Hz), 123.0, 121.2 (q, $J_{C\text{-}F}$ = 3.5 Hz), 116.6 (q, $J_{C\text{-}F}$ = 3.8 Hz), 114.3, 65.6.

FTIR (film, KBr): ν (cm^{-1}) = 3314, 3162, 3091, 3055, 2925, 1672, 1603, 1531, 1499, 1448, 1429, 1333, 1286, 1265, 1243, 1168, 1127, 1099, 1071, 918, 893, 794, 752, 738, 696.

HRMS (TOF/ESI$^-$) Calculated for $C_{21}H_{26}ON_2F_3$ $[M\text{-}H]^-$ 369.12202, found 369.12164.

***N*-(4-Trifluoromethylphenyl)-α-(phenylamino)benzeneacetamide**

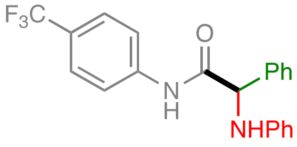

3q

Following the general procedure C, **3q** was obtained from *N*-(4-trifluoromethylphenyl)oxamic acid (129 mg, 0.55 mmol, 1.0 eq.) and diphenylimine (100 mg, 0.55 mmol, 1.0 eq.) as a white powder (160 mg, 0.43 mmol, 78%) after purification by flash column chromatography on silica gel (Chex/EtOAc 95:5) (Rf = 0.24, Chex/EtOAc 90:10).

^{1}H NMR ($CDCl_3$, 300 MHz) : δ_H 9.01 (1H, br s), 7.68-7.65 (2H, m), 7.56-7.54 (2H, m), 7.51-7.48 (2H, m), 7.42-7.40 (3H, m), 7.28-7.23 (2H, m), 6.93-6.88 (1H, m), 6.75-6.72 (2H, m), 4.86 (1H, s), 4.49 (1H, br s).

^{19}F NMR ($CDCl_3$, 272 MHz): δ_F -62.1.

^{13}C NMR ($CDCl_3$, 75 MHz) : δ_C 170.3, 146.4, 140.4 (d, $J_{C\text{-}F}$ = 1.4 Hz), 138.1, 129.7, 129.6, 129.2, 127.4, 126.5 (q, $J_{C\text{-}F}$ = 33 Hz), 126.4 (q, $J_{C\text{-}F}$ = 3.7 Hz), 124.1 (q, $J_{C\text{-}F}$ = 272 Hz), 120.3, 119.7, 114.3, 65.7.

FTIR (film, KBr): ν (cm^{-1}) = 3312, 3056, 3033, 2883, 1913, 1675, 1604, 1526, 1504, 1455, 1410, 1324, 1260, 1166, 1119, 1068, 1018, 840, 752, 738, 695, 507.

HRMS (TOF/ESI$^-$) Calculated for $C_{21}H_{16}ON_2F_3$ $[M\text{-}H]^-$ 369.12202, found 369.12177.

m.p. 161°C (toluene).

Ethyl 4-[[2-phenyl-2-(phenylamino)acetyl]amino]benzoate

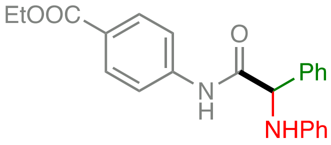

3r

Following the general procedure C, **3r** was obtained from 1-ethyl 4-[(carboxycarbonyl)amino]benzoate (131 mg, 0.55 mmol, 1.0 eq.) and diphenylimine (100 mg, 0.55 mmol, 1.0 eq.) as a white foam (133 mg, 0.35 mmol, 64%) after purification by flash column chromatography on silica gel (Chex/EtOAc 90:10) (Rf = 0.13, Chex/EtOAc 90:10).

^{1}H NMR ($CDCl_3$, 300 MHz) : δ_H 9.00 (1H, br s), 8.00-7.97 (2H, m), 7.63-7.60 (2H, m), 7.48-7.47 (2H, m), 7.47-7.38 (3H, m), 7.26-7.21 (2H, m), 6.90-6.85 (1H, m), 6.74-6.71 (2H, m), 4.86 (1H, s), 4.51 (1H, br s), 4-38-4.31 (2H, q, *J* = 7.5 Hz), 1.40-1.36 (3H, t, *J* = 7.5 Hz).

^{13}C NMR ($CDCl_3$, 75 MHz) : δ_C 170.2, 166.2, 146.4, 141.4, 138.2, 130.8, 129.7, 129.5, 129.1, 127.5, 126.4, 120.2, 119.2, 114.3, 65.6, 61.0, 14.4.

FTIR (film, KBr): ν (cm^{-1}) = 3321, 3055, 2983, 2903, 1697, 1602, 1519, 1492, 1454, 1430, 1408, 1368, 1311, 1280, 1174, 1108, 1020, 857, 770, 751, 736, 694.
HRMS (TOF/ESI$^-$) Calculated for $C_{23}H_{21}O_3N_2$ [M-H]$^-$ 373.15577, found 373.15549.
m.p. 173-175°C (toluene).

***N*-(3,5-dimethylphenyl)-α-(4-trifluoromethylphenylamino)-4-methoxyphenylacetamide**

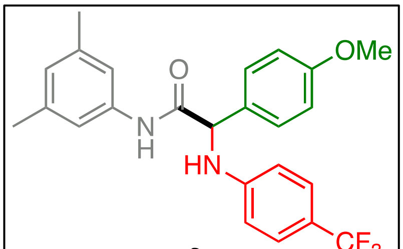

3s

Following the general procedure C, **3s** was obtained from *N*-(3,5-dimethylphenyl)oxamic acid (100 mg, 0.52 mmol, 1.0 eq.) and **2s** (152 mg, 0.52 mmol, 1.0 eq.) as a white solid (30 mg, 0.07 mmol, 18%) after purification by flash column chromatography on silica gel (Chex/EtOAc 95:5) (Rf = 0.15, Chex/EtOAc 90:10).
Following the general procedure D, **3s** was obtained from *N*-(3,5-dimethylphenyl)oxamic acid (100 mg, 0.52 mmol, 1.0 eq.), anisaldehyde (71 mg, 0.52 mmol, 1.0 eq.) and (4-trifluoromethyl)aniline (83 mg, 0.52 mmol, 1.0 eq.) as a white solid (85 mg, 0.20 mmol, 38%) after purification by flash column chromatography on silica gel (Chex/EtOAc 90:10) (Rf = 0.19, Chex/EtOAc 85:15).
^{1}H NMR (CDCl$_3$, 300 MHz) : δ_H 8.19 (1H, s), 7.42-7.39 (4H, m), 7.12 (2H, s), 6.94-6.89 (2H, m), 6.77 (1H, s), 6.68-6.65 (2H, m), 5.10 (1H, br s), 4.84 (1H, s), 3.79 (3H, s), 2.26 (6H, s).
^{19}F NMR (CDCl$_3$, 272 MHz): δ_F -61.3.
^{13}C NMR (CDCl$_3$, 75 MHz) : δ_C 169.2, 160.2, 149.0, 139.0, 137.0, 130.0, 128.6, 126.9 (q, J_{C-F} = 3.8 Hz), 126.8, 120.9 (q, J_{C-F} = 33 Hz), 120.4 (J_{C-F} = 271 Hz), 117.9, 115.0, 113.4, 63.4, 55.5, 21.4.
FTIR (film, KBr): ν (cm^{-1}) = 3296, 3009, 2957, 2920, 2855, 1661, 1616, 1532, 1513, 1464, 1443, 1323, 1255, 1185, 1162, 1112, 1066, 1034, 827.
HRMS (TOF/ESI$^+$) Calculated for $C_{24}H_{24}O_2N_2F_3$ [M+H]$^+$ 429.17844 found 429.17905.
m.p. 114-116°C.

***N*-(phenyl)-α-(4-cyanophenylamino)-3-fluorophenylacetamide**

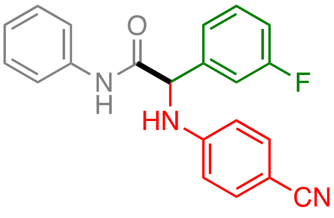

3t

Following the general procedure C, **3t** was obtained from *N*-(phenyl)oxamic acid (90 mg, 0.54 mmol, 1.0 eq.) and *N*-[(3-fluoro)phenylmethylene]-4-(cyano)benzenamine (120 mg, 0.54 mmol, 1.0 eq.) as a white solid (78 mg, 0.23 mmol, 41%) after purification by flash column chromatography on silica gel (Chex/EtOAc 87:13) (Rf = 0.18, Chex/EtOAc 80:20).
Following the general procedure D, **3a** was obtained from *N*-(phenyl)oxamic (90 mg, 0.54 mmol, 1.0 eq.), 3-fluorobenzaldehyde (68 mg, 0.54 mmol, 1.0 eq.) and (4-cyano)aniline (64 mg, 0.54 mmol, 1.0 eq.) as a white solid (50 mg, 0.15 mmol, 27%) after purification by flash column chromatography on silica gel (Chex/EtOAc 90:10 to 80:20) (Rf = 0.10, Chex/EtOAc 85:15).
^{1}H NMR (CDCl$_3$, 300 MHz) : δ_H 8.07 (1H, m), 7.47-7.22 (9H, m), 7.16-7.11 (1H, m), 7.09-7.04 (1H, m), 6.60-6.57 (2H, m), 5.62 (1H, br s), 4.98 (1H, s).
^{19}F NMR (CDCl$_3$, 272 MHz): δ_F -110.4.
^{13}C NMR (CDCl$_3$, 75 MHz) : δ_C 167.1, 163.4 (d, J_{C-F} = 249 Hz), 149.3, 140.1 (d, J_{C-F} = 6.8 Hz), 136.9, 133.9, 131.4 (d, J_{C-F} = 8.3 Hz), 125.4, 123.0 (d, J_{C-F} = 3.1 Hz), 120.3, 120.2, 116.4 (d, J_{C-F} = 21 Hz), 114.3 (d, J_{C-F} = 22 Hz), 113.6, 100.7, 62.1 (d, J_{C-F} = 1.7 Hz).

FTIR (film, KBr): ν (cm^{-1}) = 3329, 3143, 3061, 2216, 1672, 1606, 1520, 1499, 1487, 1445, 1420, 1333, 1317, 1254, 1175, 1148, 825, 754, 738, 690.
HRMS (TOF/ESI^{+}) Calculated for $C_{21}H_{17}ON_3F$ $[M+H]^+$ 346.13502 found 346.13528.
m.p. 166-168°C (toluene).

Methyl-4-[2-(cyclopropylamino)-1-(3,4-bromophenylamino)]-2-oxoethyl]benzoate

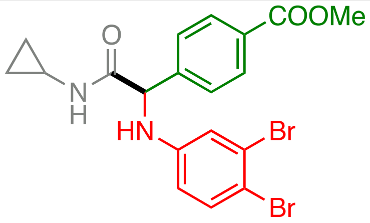

Following the general procedure C, **3u** was obtained from *N*-(cyclopropyl)oxamic acid (75 mg, 0.58 mmol, 1.0 eq.) and methyl **2u** (229 mg, 0.58 mmol, 1.0 eq.) as a white solid (164 mg, 0.23 mmol, 58%) after purification by flash column chromatography on silica gel (Chex/EtOAc 80:20) (Rf = 0.09, Chex/EtOAc 80:20).
Following the general procedure D, **3u** was obtained from (cyclopropyl)oxamic acid (75 mg, 0.58 mmol, 1.0 eq.), methyl 4-formylbenzoate (95 mg, 0.58 mmol, 1.0 eq.) and (3,4-dibromo)aniline (146 mg, 0.58 mmol, 1.0 eq.) as a white solid (127 mg, 0.34 mmol, 45%) after purification by flash column chromatography on silica gel (Chex/EtOAc 90:10 to 80:20) (Rf = 0.09, Chex/EtOAc 80:20).
^{1}H NMR ($CDCl_3$, 300 MHz) : δ_H 8.04-8.02 (2H, m), 7.49-7.46 (2H, m), 7.31-7.26 (1H, m), 6.83-6.82 (1H, m), 6.39-6.36 (1H, m), 6.30 (1H, br s), 4.73 (1H, s), 3.91 (3H, s), 2.70-2.64 (1H, m), 0.77-0.74 (2H, m), 0.43-0.42 (2H, m).
^{13}C NMR ($CDCl_3$, 75 MHz) : δ_C 170.8, 166.5, 145.7, 142.7, 133.9, 130.8, 130.8, 127.4, 125.3, 118.9, 114.8, 113.4, 62.7, 52.4, 23.1, 6.9, 6.7.
FTIR (film, KBr): ν (cm^{-1}) = 3336, 3058, 3009, 2952, 2924, 2851, 1718, 1660, 1591, 1488, 1466, 1436, 1311, 1284, 1192, 1113, 1020, 738, 705.
HRMS (TOF/ESI^{+}) Calculated for $C_{19}H_{19}O_3N_2Br_2$ $[M+H]^+$ 480.97569 found 480.97620.
m.p. 163°C (toluene).

Ethyl 4-[[2-(4-methylbenzylamino)-1-(4-cyanophenyl)-2-oxoethyl]amino]benzoate

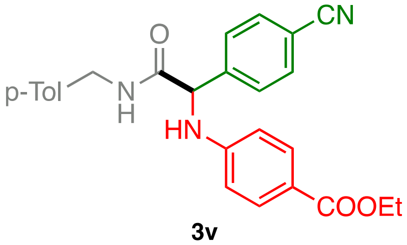

Following the general procedure C, **3v** was obtained from *N*-(4-methylbenzyl)oxamic acid (100 mg, 0.52 mmol, 1.0 eq.) and ethyl 4-[[(4-cyanophenyl)methylene]amino]benzoate (144 mg, 0.52 mmol, 1.0 eq.) as a white solid (141 mg, 0.33 mmol, 64%) after purification by flash column chromatography on silica gel (Chex/EtOAc 90:10 to 80:20) (Rf = 0.26, Chex/EtOAc 80:20).
Following the general procedure D, **3v** was obtained from **2v** (100 mg, 0.52 mmol, 1.0 eq.), 4-cyanobenzaldehyde (68 mg, 0.52 mmol, 1.0 eq.) and benzocaine (86 mg, 0.52 mmol, 1.0 eq.) as a white solid (137 mg, 0.32 mmol, 62%) after purification by flash column chromatography on silica gel (Chex/EtOAc 95:5 to 80:20) (Rf = 0.26, Chex/EtOAc 80:20).
^{1}H NMR ($CDCl_3$, 300 MHz) : δ_H 7.83-7.80 (2H, m), 7.67-7.64 (2H, m), 7.58-7.56 (2H, m), 7.10-7.07 (2H, m), 7.01-6.98 (2H, m), 6.53-6.49 (2H, m), 6.47 (1H, s), 5.44-5.42 (1H, d, *J* = 4.0 Hz), 4.96-4.95 (1H, d, *J* = 3.2 Hz), 4.48-4.30 (2H, m), 4.30-4.23 (2H, q, *J* =7.1Hz), 2.31 (3H, s), 1.35-1.31 (3H, t, *J* = 7.1 Hz).
^{13}C NMR ($CDCl_3$, 75 MHz) : δ_C 169.0, 166.7, 149.4, 143.8, 137.8, 134.3, 133.2, 131.6, 129.6, 128.1, 127.7, 120.8, 118.4, 112.8, 62.1, 60.6, 43.9, 21.2, 14.5

FTIR (film, KBr): ν (cm^{-1}) = 3333, 3056, 2982, 2925, 2873, 2231, 1671, 1605, 1524, 1472, 1418, 1367, 1334, 1313, 1280, 1175, 1108, 1021, 840, 806, 771, 736, 700.
HRMS (TOF/ESI^{+}) Calculated for $C_{26}H_{26}O_3N_3$ [M+H]$^{+}$ 428.19687 found 428.19745.
m.p. 197°C (toluene).

N-(cyclohexyl)-α-(phenylamino)-4-fluorobenzeneacetamide

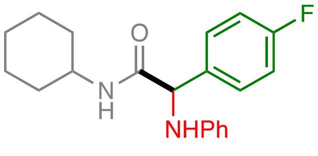

3w

Following the general procedure D, **3w** was obtained from *N*-(cyclohexyl)oxamic acid (100 mg, 0.58 mmol, 1.0 eq.), 4-fluorobenzaldehyde (73 mg, 0.58 mmol, 1.0 eq.) and aniline (54 mg, 0.58 mmol, 1.0 eq.) as a white solid (139 mg, 0.42 mmol, 73%) after purification by flash column chromatography on silica gel (Chex/EtOAc 95:5) (Rf = 0.16, Chex/EtOAc 90:10).
^{1}H NMR (CDCl$_3$, 300 MHz) : δ$_H$ 7.43-7.38 (2H, m), 7.22-7.17 (2H m), 7.09-7.03 (2H, m), 6.84-6.79 (1H, m), 6.64-6.61 (2H, m), 6.58 (1H, br s), 4.69 (1H, s), 4.46 (1H, br s), 3.85-3.75 (1H, m), 1.90-1.77 (2H, m), 1.70-1.59 (3H, m), 1.37-1.29 (2H, m), 1.17-1.00 (3H, m).
^{19}F NMR (CDCl$_3$, 272 MHz): δ$_F$ -113.3.
^{13}C NMR (CDCl$_3$, 75 MHz) : δ$_C$ 169.9, 162.8 (d, J_{C-F} = 248 Hz), 146.6, 135.0 (d, J_{C-F} = 3.3 Hz), 129.4, 129.2 (d, J_{C-F} = 8.3 Hz), 119.4, 116.2 (d, J_{C-F} = 21.6 Hz), 114.0, 63.7, 48.3, 33.1, 32.9, 25.5, 24.9, 24.7.
FTIR (film, KBr): ν (cm^{-1}) = 3306, 3054, 2930, 2885, 1658, 1652, 1648, 1604, 1506, 1451, 1433, 1316, 1263, 1227, 1157, 1094, 841, 804, 750, 692.
HRMS (TOF/ESI+) Calculated for $C_{20}H_{24}ON_2F$ [M+H]$^{+}$ 327.18672 found 327.18644.
HRMS (TOF/ESI+) Calculated for $C_{20}H_{23}ON_2FNa$ [M+Na]$^{+}$ 349.16866 found 349.16840.
m.p. 112-114°C (toluene).

Methyl-4-[2-(cyclohexylamino)-1-(phenylamino)]-2-oxoethyl]benzoate

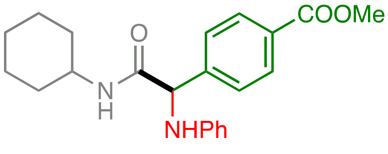

3x

Following the general procedure D, **3x** was obtained from *N*-(cyclohexyl)oxamic acid (100 mg, 0.58 mmol, 1.0 eq.), methyl 4-formylbenzoate (96 mg, 0.58 mmol, 1.0 eq.) and aniline (54 mg, 0.58 mmol, 1.0 eq.) as a white powder (134 mg, 0.37 mmol, 63%) after purification by flash column chromatography on silica gel (Chex/EtOAc 90:10) (Rf = 0.08, Chex/EtOAc 80:20).
^{1}H NMR (CDCl$_3$, 300 MHz) : δ$_H$ 8.05-8.02 (2H, m), 7.53-7.50 (2H, m), 7.18-7.15 (2H, m), 6.82-6.77 (1H, m), 6.63-6.60 (2H, m), 6.54-6.52 (1H, m), 4.77 (1H, s), 4.59 (1H, s), 3.91 (3H, s), 3.84-3.73 (1H, m), 1.89-1.85 (2H, m), 1.69-1.55 (3H, m), 1.40-1.24 (2H, m), 1.20-0.96 (3H, m).
^{13}C NMR (CDCl$_3$, 75 MHz) : δ$_C$ 169.4, 167.7, 146.5, 144.1, 130.6, 130.4, 129.5, 127.5, 119.4, 114.0, 64.0, 52.3, 48.4, 33.0, 32.8, 25.5, 24.8, 24.7.
FTIR (film, KBr): ν (cm^{-1}) = 3327, 3053, 2928, 2854, 1723, 1650, 1603, 1505, 1450, 1435, 1349, 1314, 1283, 1112, 748, 693.
HRMS (TOF/ESI^{+}) Calculated for $C_{22}H_{27}O_3N_2$ [M+H]$^{+}$ 367.20162 found 367.20143.
HRMS (TOF/ESI^{+}) Calculated for $C_{22}H_{26}O_3N_2Na$ [M+Na]$^{+}$ 389.18356 found 389.18324.
m.p. 177°C (toluene).

N-(cyclohexyl)-α-(phenylamino)-4-dimethylaminobenzeneacetamide

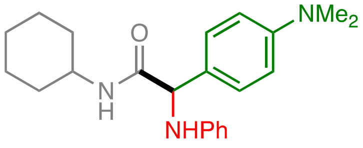

3y

Following the general procedure D, **3y** was obtained from *N*-(cyclohexyl)oxamic acid (100 mg, 0.58 mmol, 1.0 eq.), 4-(dimethylamino)benzaldehyde (87 mg, 0.58 mmol, 1.0 eq.) and aniline (54 mg, 0.58 mmol, 1.0 eq.). Crude NMR (7%) was obtained adding mesitylene as an external standard (42.6 mg, 0.61 eq.).

_N_-(cyclohexyl)-α-(phenylamino)-4-cyanpobenzeneacetamide

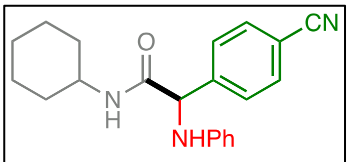

3z

Following the general procedure D, **3z** was obtained from *N*-(cyclohexyl)oxamic acid (100 mg, 0.58 mmol, 1.0 eq.), 4-cyanobenzaldehyde (77 mg, 0.58 mmol, 1.0 eq.) and aniline (54 mg, 0.58 mmol, 1.0 eq.) as a white powder (127 mg, 0.38 mmol, 65%) after purification by flash column chromatography on silica gel (Chex/EtOAc 93:7) (Rf = 0.06, Chex/EtOAc 90:10).

^{1}H NMR ($CDCl_3$, 300 MHz) : δ_H 7.67-7.64 (2H, m), 7.58-7.55 (2H, m), 7.21-7.16 (2H, m), 6.84-6.79 (1H, m), 6.61-6.59 (2H, m), 6.53-6.50 (1H, m), 4.78 (1H, s), 4.57 (1H, br s), 1.90-1.85 (2H m), 1.70-1.58 (3H, m), 1.40-1.25 (2H, m), 1.21-0.96 (3H, m).

^{13}C NMR ($CDCl_3$, 75 MHz) : δ_C 168.9, 146.1, 144.4, 133.0, 129.5, 128.2, 119.7, 118.5, 114.0, 112.5, 63.8, 48.6, 33.1, 32.8, 25.5, 24.8, 24.7.

FTIR (film, KBr): ν (cm^{-1}) = 3327, 3054, 2930, 2854, 2230, 1650, 1603, 1504, 1451, 1433, 1350, 1317, 1261, 750, 692.

HRMS (TOF/ESI$^+$) Calculated for $C_{21}H_{24}ON_3$ $[M+H]^+$ 334.19139 found 334.19111.

HRMS (TOF/ESI$^+$) Calculated for $C_{21}H_{23}ON_3Na$ $[M+Na]^+$ 356.17333 found 356.17308.

m.p. 174-176°C.

_N_-(cyclohexyl)-α-(phenylamino)-4-methoxybenzeneacetamide

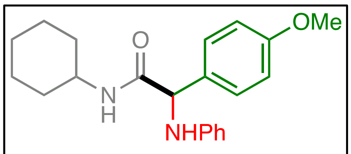

3aa

Following the general procedure D, **3aa** was obtained from *N*-(cyclohexyl) oxamic acid (100 mg, 0.58 mmol, 1.0 eq.), anisaldehyde (80 mg, 0.58 mmol, 1.0 eq.) and aniline (54 mg, 0.58 mmol, 1.0 eq.) as a white powder (138 mg, 41 mmol, 70%) after purification by flash column chromatography on silica gel (Chex/EtOAc 90:10) (Rf = 0.09, Chex/EtOAc 90:10).

^{1}H NMR ($CDCl_3$, 300 MHz) : δ_H 7.35-7.32 (2H, m), 7.21-7.16 (2H, m), 6.91-6.88 (2H, m), 6.82-6.77 (1H, m), 6.64-6.61 (3H, m), 4.65 (1H, s), 4.42 (1H, br s), 3.80 (4H, s), 1.91-1.77 (2H, m), 1.70-1.65 (3H, m), 1.37-1.29 (2H, m), 1.17-1.00 (3H, m).

^{13}C NMR ($CDCl_3$, 75 MHz) : δ_C 170.4, 159.8, 146.9, 131.2, 129.4, 128.7, 119.2, 114.6, 114.0, 63.9, 55.4, 48.2, 33.1, 32.9, 25.5, 24.9, 24.8 .

FTIR (film, KBr): ν (cm^{-1}) = 3304, 3052, 3001, 2927, 2853, 1648, 1604, 1510, 1464, 1451, 1315, 1305, 1250, 1178, 1033, 749, 692.

HRMS (TOF/ESI$^+$) Calculated for $C_{21}H_{27}O_2N_2$ $[M+H]^+$ 331.20671 found 339.20655.

HRMS (TOF/ESI$^+$) Calculated for $C_{21}H_{26}O_2N_2Na$ $[M+Na]^+$ 361.18865 found 361.18834.

m.p. 162°C (toluene).

_N_-(cyclohexyl)-α-(phenylamino)-4-fluorobenzeneacetamide

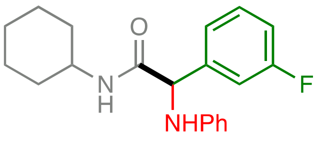

3ab

Following the general procedure D, **3ab** was obtained from *N*-(cyclohexyl)oxamic acid (100 mg, 0.58 mmol, 1.0 eq.), 3-fluorobenzaldehyde (73 mg, 0.58 mmol, 1.0 eq.) and aniline (54 mg, 0.58 mmol, 1.0 eq.) as a white powder (130 mg, 39 mmol, 68%) after purification by flash column chromatography on silica gel (Chex/EtOAc 93:3) (Rf = 0.16, Chex/EtOAc 90:10).

^{1}H NMR ($CDCl_3$, 300 MHz) : δ_H 7.36-7.31 (1H, m), 7.24-7.13 (4H, m), 7.06-7.03 (1H, m), 6.84-6.78 (1H, m), 6.64-6.60 (2H, m), 6.56-6.53 (1H, m), 4.71 (1H, s), 4.53 (1H, br s), 3.86-3.73 (1H, m), 1.92-1.77 (2H, m), 1.70-1.57 (3H, m), 1.42-1.26 (2H, m), 1.21-0.96 (3H, m).

^{19}F NMR ($CDCl_3$, 272 MHz): δ_F -111.5.

^{13}C NMR ($CDCl_3$, 75 MHz) : δ_C 169.5, 163.2 (d, $J_{C\text{-}F}$ = 248 Hz), 146.5, 141.6 (d, $J_{C\text{-}F}$ = 8 Hz), 130.9 (d, $J_{C\text{-}F}$ = 8 Hz), 129.4, 123.2, 119.3, 115.6 (d, $J_{C\text{-}F}$ = 21 Hz), 114.4 (d, $J_{C\text{-}F}$ = 22 Hz), 114.0, 63.8, 48.4, 33.0, 32.8, 25.5, 24.9, 24.7.

FTIR (film, KBr): ν (cm^{-1}) = 3304, 3055, 2926, 2854, 1652,1646, 1603, 1505, 1488, 1449, 1350, 1317, 1253, 1153, 781, 748, 691, 1650, 1603, 1505, 1450, 1435, 1349, 1314, 1283, 1112, 748, 693.

HRMS (TOF/ESI$^+$) Calculated for $C_{20}H_{24}ON_2F$ $[M+H]^+$ 327.18672 found 327.18642.

HRMS (TOF/ESI$^+$) Calculated for $C_{20}H_{23}ON_2FNa$ $[M+Na]^+$ 349.16866 found 349.16840.

m.p. 187°C (toluene).

N-(cyclohexyl)-α-(phenylamino)-3-methylbenzeneacetamide

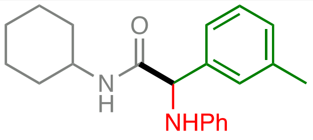

3ac

Following the general procedure D, **3ac** was obtained from *N*-(cyclohexyl)oxamic acid (100 mg, 0.58 mmol, 1.0 eq.), 3-methylbenzaldehyde (70 mg, 0.58 mmol, 1.0 eq.) and aniline (54 mg, 0.58 mmol, 1.0 eq.) as a white powder (125 mg, 38 mmol, 66%) after purification by flash column chromatography on silica gel (Chex/EtOAc 95:5) (Rf = 0.18, Chex/EtOAc 90:10).

^{1}H NMR ($CDCl_3$, 300 MHz) : δ_H 7.29-7.13 (6H, m), 8.83-6.78 (1H, m), 6.65-6.62 (3H, m), 4.66 (1H, s), 4.46 1H, br s), 3.86-3.76 (1H, m, CH_{Cy}), 2.35 (3H, s), 1.91-1.78 (2H, m), 1.66-1.58 (3H, m), 1.38-1.30 (2H, m), 1.18-1.06 (3H, m).

^{13}C NMR ($CDCl_3$, 75 MHz) : δ_C 170.2, 147.0, 139.1, 139.0, 129.4, 129.16, 128.3, 124.4, 119.2, 114.0, 64.6, 48.2, 33.1, 32.9, 25.6, 24.9, 24.8, 21.2

FTIR (film, KBr): ν (cm^{-1}) = 3306, 3051, 3024, 2926, 2854, 1652, 1646, 1603, 1549, 1505, 1464, 1451, 1434, 1317, 1254, 778, 748, 692.

HRMS (TOF/ESI$^+$) Calculated for $C_{21}H_{27}ON_2$ $[M+H]^+$ 323.21179 found 323.21158.

HRMS (TOF/ESI$^+$) Calculated for $C_{21}H_{26}ON_2Na$ $[M+Na]^+$ 345.19373 found 345.19353.

m.p. 134-136°C (toluene).

N-(cyclohexyl)-α-(phenylamino)-2,4,6-trimethylbenzeneacetamide

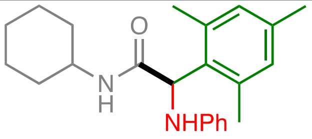

3ad

Following the general procedure D, **3ad** was obtained from *N*-(cyclohexyl)oxamic acid (100 mg, 0.58 mmol, 1.0 eq.), 2,4,6-trimethylbenzaldehyde (87 mg, 0.58 mmol, 1.0 eq.) and aniline (54 mg, 0.58 mmol, 1.0 eq.) as a colorless oil (67 mg, 21 mmol, 36%) after

purification by flash column chromatography on silica gel (Chex/EtOAc 97:3) (Rf = 0.23, Chex/EtOAc 90:10).

^{1}H NMR ($CDCl_3$, 300 MHz) : δ_H 7.19-7.14 (2H, m), 6.87 (2H, s), 6.79-6.74 (1H, m), 6.62-6.59 (2H, m), 6.35-6.32 (1H, m), 5.13 (1H, s), 3.91-3.81 (1H, m), 2.35 (6H, s), 2.26 (3H, s), 1.88-1.85 (2H, m), 1.65-1.59 (3H, m), 1.39-1.27 (2H, m), 1.14-1.07 (3H, m).

^{13}C NMR ($CDCl_3$, 75 MHz) : δ_C 170.7, 147.9, 137.8, 137.1, 133.1, 130.5, 129.4, 118.8, 113.8, 59.7, 48.3, 33.0, 32.8, 25.6, 24.9, 24.8, 21.0, 20.9.

FTIR (film, KBr): ν (cm^{-1}) = 3369, 2923, 2852, 1656, 1604, 1515, 1505, 1464, 1447, 1316, 749.

HRMS (TOF/ESI$^-$) Calculated for $C_{23}H_{29}ON_2$ $[M-H]^-$ 349.22854 found 349.22814.

_N_-(cyclohexyl)-3-methoxy-α-(phenylamino)phenolacetamide

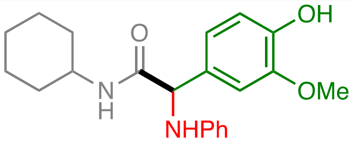

3ae

Following the general procedure D, **3ae** was obtained from *N*-(cyclohexyl)oxamic acid (100 mg, 0.58 mmol, 1.0 eq.), vaniline (89 mg, 0.58 mmol, 1.0 eq.) and aniline (54 mg, 0.58 mmol, 1.0 eq.) as a white powder (106 mg, 29 mmol, 51%) after purification by flash column chromatography on silica gel (Chex/EtOAc 80:20) (Rf = 0.06, Chex/EtOAc 75:25).

^{1}H NMR ($CDCl_3$, 300 MHz) : δ_H 7.21-7.16 (2H, m), 6.90 (3H, m), 6.82-6.77 (1H, m), 6.64-6.57 (3H, m), 5.89-5.88 (1H, br s), 4.61 (1H, s), 4.56 (1H, br s), 3.85-3.79 (4H, s), 1.90-1.76 (2H, m), 1.70-1.57 (3H, m), 1.38-1.25 (2H, m), 1.21-0.97 (3H, m).

^{13}C NMR ($CDCl_3$, 75 MHz) : δ_C 170.5, 147.2, 147.0, 146.0, 131.0, 129.4, 120.2, 119.2, 115.0, 114.0, 110.1, 64.3, 56.0, 48.2, 33.2, 32.9, 25.5, 24.9, 24.7.

FTIR (film, KBr): ν (cm^{-1}) = 3339, 3053, 2930, 2854, 1645, 1603, 1514, 1506, 1464, 1451, 1432, 1373, 1350, 1314, 1267, 1233, 1182, 1155, 1128, 1032, 809, 751, 737, 894.

HRMS (TOF/ESI$^-$) Calculated for $C_{21}H_{25}O_3N_2$ $[M-H]^-$ 353.18707, found 353.18698.

m.p. 136-138°C (toluene).

_N_-(Cyclohexyl)-α-(phenylamino)-3,4-dimethoxybenzeneacetamide

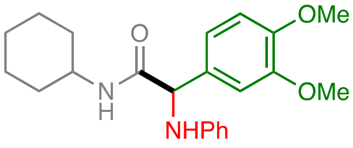

3af

Following the general procedure D, **3af** was obtained from *N*-(cyclohexyl)oxamic acid (100 mg, 0.58 mmol, 1.0 eq.), 3,4-dimethoxybenzaldehyde (97 mg, 0.58 mmol, 1.0 eq.) and aniline (54 mg, 0.58 mmol, 1.0 eq.) as a colorless oil (95 mg, 26 mmol, 44%) after purification by flash column chromatography on silica gel (Chex/EtOAc 85:15) (Rf = 0.16, Chex/EtOAc 80:20).

^{1}H NMR ($CDCl_3$, 300 MHz) : δ_H 7.21-7.16 (2H, m), 6.99-6.93 (2H, m), 6.87-6.80 (2H, m), 6.65-6.63 (2H, m), 6.57-6.54 (1H, m), 6.64 (1H, s), 4.60 (1H, br s), 1.91-1.77 (2H, m), 1.69-1.57 (3H, m), 1.38-1.29 (2H, m), 1.17-1.04 (3H, m).

^{13}C NMR ($CDCl_3$, 75 MHz) : δ_C 170.2, 149.6, 149.3, 146.8, 131.7, 119.8, 119.3, 114.1, 111.6, 110.5, 64.2, 56.1, 56.0, 48.2, 33.2, 32.9, 25.5, 24.9, 24.7.

FTIR (film, KBr): ν (cm^{-1}) = 3353, 3053, 2925, 2853, 1652, 1646, 1604, 1513, 1506, 1464, 1453, 1317, 1259, 1235, 1144, 1028, 750, 693.

HRMS (TOF/ESI$^+$) Calculated for $C_{22}H_{29}O_3N_2$ $[M+H]^+$ 369.21727 found 369.21718.

HRMS (TOF/ESI$^+$) Calculated for $C_{22}H_{28}O_3N_2Na$ $[M+Na]^+$ 391,19921 found 391.19897.

m.p. 112-114°C.

***N*-(Cyclohexyl)-α-(phenylamino)[5-methyl-2-furyl]acetamide**

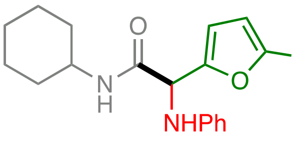

3ag

Following the general procedure D, **3ag** was obtained from *N*-(cyclohexyl)oxamic acid (100 mg, 0.58 mmol, 1.0 eq.), 5-methyl-2-furaldehyde (64 mg, 0.58 mmol, 1.0 eq.) and aniline (54 mg, 0.58 mmol, 1.0 eq.) as an orange oil (52 mg, 16 mmol, 28%) after purification by flash column chromatography on silica gel (Chex/EtOAc 95:5 to 90:10) (Rf = 0.16, Chex/EtOAc 90:10).

^{1}H NMR ($CDCl_3$, 300 MHz) : δ_H 7.25-7.20 (2H, m), 6.86-6.83 (1H, m), 6.69-6.65 (2H, m), 6.26-6.25 (1H, d, *J* = 3.1 Hz), 5.96-5.95 (1H, dd, *J* = 3.1 Hz, *J* = 1.2 Hz), 4.80 (1H, s), 4.60 (1H, br s), 3.90-3.78 (1H, m), 2.31 (3H, s), 1.94-1.81 (2H, m), 1.74-1.58 (3H, m), 1.41-1.28 (2H, m), 1.23-1.06 (3H, m).

^{13}C NMR ($CDCl_3$, 75 MHz) : δ_C 168.2, 152.4, 148.9, 146.6, 129.3, 119.2, 113.8, 109.2, 106.7, 58.2, 48.2, 32.8, 32.7, 25.4, 24.7, 24.6, 13.6.

FTIR (film, KBr): ν (cm^{-1}) = 3314, 2930, 2854, 1652, 1603, 1504, 1450, 1317, 1254, 1221, 1023, 750, 693.

HRMS (TOF/ESI$^+$) Calculated for $[M+H]^+$ 313.19105 found 313.19049. $C_{19}H_{25}O_2N_2$

***N*-(Cyclohexyl)-α-(4-cyanophenylamino)phenylacetamide**

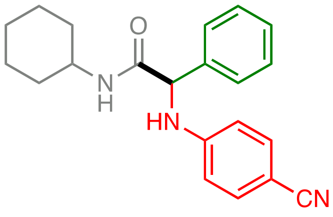

3ah

Following the general procedure D, **3ah** was obtained from *N*-(cyclohexyl)oxamic acid (100 mg, 0.58 mmol, 1.0 eq.), benzaldehyde (62 mg, 0.58 mmol, 1.0 eq.) and 4-cyanoaniline (69 mg, 0.58 mmol, 1.0 eq.) as a white powder (77 mg, 23 mmol, 40%) after purification by flash column chromatography on silica gel (Chex/EtOAc 87:13) (Rf = 0.10, Chex/EtOAc 85:15).

^{1}H NMR ($CDCl_3$, 300 MHz) : δ_H 7.41-7.34 (7H, m), 6.56-6.53 (2H, m), 5.82-5.79 (1H, m), 5.64 (1H, br s), 4.77 (1H, s), 3.79-3.69 (1H, m), 1.90-1.86 (1H, m), 1.70-1.53 (4H, m), 1.36-1.25 (3H, m), 1.19-1.07 (2H, m).

^{13}C NMR ($CDCl_3$, 75 MHz) : δ_C 168.7, 149.6, 138.2, 133.6, 129.5, 128.9, 127.0, 120.1, 113.4, 100.1, 62.0, 48.7, 32.8, 32.6, 25.3, 24.6, 24.5.

FTIR (film, KBr): ν (cm^{-1}) = 3324, 3063, 2932, 2855, 2215, 1671, 1606, 1521, 1452, 1335, 1173, 827, 732, 697.

HRMS (TOF/ESI$^+$) Calculated for $C_{21}H_{24}ON_3$ $[M+H]^+$ 334.19139 found 334.19128.

m.p. 192-194°C (toluene).

Ethyl 4-[[2-((cyclohexyl)amino)-1-(phenyl)-2-oxoethyl]amino]benzoate

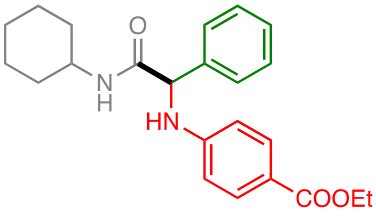

3ai

Following the general procedure D, **3ai** was obtained from *N*-(cyclohexyl)oxamic acid (100 mg, 0.58 mmol, 1.0 eq.), benzaldehyde (62 mg, 0.58 mmol, 1.0 eq.) and benzocaine (97 mg, 0.58 mmol, 1.0 eq.) as a white powder (89 mg, 23 mmol, 40%) after purification by flash column chromatography on silica gel (Chex/EtOAc 90:10) (Rf = 0.24, Chex/EtOAc 80:20).

^{1}H NMR (CDCl$_3$, 300 MHz) : δ$_H$ 7.79-7.76 (2H, m), 7.38-7.28 (5H, m), 6.51-6.48 (2H, m), 6.11-6.08 (1H, m), 5.10 (1H, br s), 4.74 (1H, s), 4.26-4.19 (2H, q, *J* = 7.1 Hz), 3.75-3.66 (1H, m), 1.86-1.80 (1H, m), 1.69-1.48 (4H, m), 1.30-1.21 (5H, t + m, *J* = 7.1 Hz), 1.09-0.85 (3H, m).
^{13}C NMR (CDCl$_3$, 75 MHz) : δ$_C$ 169.3, 166.8, 150.3, 138.6, 131.5, 129.4, 128.8, 127.3, 120.5, 112.8, 62.8, 60.4, 48.6, 33.0, 32.7, 25.5, 24.8, 24.7, 14.5.
FTIR (film, KBr): ν (cm^{-1}) = 3333, 3063, 3033, 2981, 2933, 2856 1689, 1681, 1668, 1661, 1652, 1606, 1524, 1495, 1477, 1453, 1417, 1392, 1367, 1335, 1313, 1279, 1175, 1107, 1024, 840, 771, 784, 699.
HRMS (TOF/ESI$^+$) Calculated for $C_{23}H_{29}O_3N_2$ [M+H]$^+$ 381.21727 found 381.21710.
m.p. 117-120°C (toluene)

Methyl-4-[2-(cyclopropylamino)-1-(3,4-dichlorophenylamino)]-2-oxoethyl]benzoate

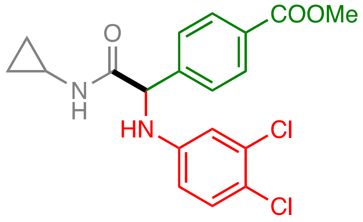

3aj

Following the general procedure D, **3aj** was obtained from *N*-(cyclopropyl)oxamic acid (75 mg, 0.58 mmol, 1.0 eq.), methyl 4-formylbenzoate (95 mg, 0.58 mmol, 1.0 eq.) and 3,4-dichloroaniline (94 mg, 0.58 mmol, 1.0 eq.) as a white powder (95 mg, 24 mmol, 42%) after purification by flash column chromatography on silica gel (Chex/EtOAc 80:20) (Rf = 0.09, Chex/EtOAc 80:20).
^{1}H NMR (CDCl$_3$, 300 MHz) : δ$_H$ 8.06-8.03 (2H, m), 7.50-7.47 (2H, m), 7.17-7.14 (1H, m), 6.65-6.64 (1H, s), 6.44-6.40 (1H, m), 6.22 (1H, br s), 4.74 (1H, s), 3.91 (3H, s), 2.71-2.65 (1H, m), 0.78-0.74 (2H, m), 0.43-0.42 (2H, m).
^{13}C NMR (CDCl$_3$, 75 MHz) : δ$_C$ 170.7, 166.5, 144.5, 142.4, 133.1, 130.9, 130.9, 130.8, 127.6, 122.9, 116.3, 114.5, 63.1, 52.5, 23.1, 6.9, 6.7.
FTIR (film, KBr): ν (cm^{-1}) = 3339, 3063, 3010, 2953, 2924, 2852, 1718, 1660, 1598, 1493, 1477, 1436, 1313, 1285, 1192, 1132, 1115, 1020, 738, 703.
HRMS (TOF/ESI$^+$) Calculated for $C_{19}H_{19}O_3N_2Cl_2$ [M+H]$^+$ 393.07672 found 393.07709.
m.p. 172°C (toluene).

***N*-(3,5-dimethylphenyl)-α-(3-trifluorobenzenamine)-4-methoxybenzeneacetamide**

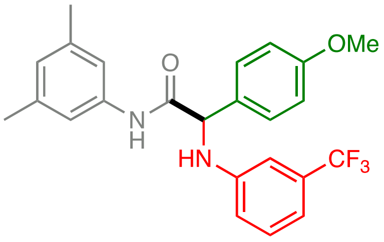

3ak

Following the general procedure D, **3ak** was obtained from *N*-(3,5-dimethylphenyl)oxamic acid (100 mg, 0.52 mmol, 1.0 eq.), anisaldehyde (71 mg, 0.52 mmol, 1.0 eq.) and 3-trifluoromethylaniline (83 mg, 0.52 mmol, 1.0 eq.) as a white powder (85 mg, 20 mmol, 38%) after purification by flash column chromatography on silica gel (Chex/EtOAc 95:5 to 90:10) (Rf = 0.37, Chex/EtOAc 80:20).
^{1}H NMR (CDCl$_3$, 300 MHz) : δ$_H$ 8.18 (1H, s), 7.42-7.39 (2H, m), 7.31-7.25 (1H, m), 7.12 (2H, s), 7.06-7.03 (1H, m), 6.94-6.90 (3H, m), 6.81-6.78 (1H, m), 6.76 (1H, s), 4.91 (1H, br s), 4.81-4.80 (1H, s), 3.80 (3H, s), 2.27 (6H, s).
^{19}F NMR (CDCl$_3$, 272 MHz): δ$_F$ -62.9.
^{13}C NMR (CDCl$_3$, 75 MHz) : δ$_C$ 169.2, 160.2, 146.8, 139.0, 137.0, 131.7 (q, J_{C-F} = 32 Hz), 130.1, 128.7, 126.7, 124.0 (q, J_{C-F} = 232 Hz), 117.9, 116.6, 115.9 (J_{C-F} = 3.6 Hz), 115.0, 111.0 (q, J_{C-F} = 3.6 Hz), 63.9, 56.5, 21.4.

FTIR (film, KBr): ν (cm^{-1}) = 3319, 3013, 2957, 2922, 2840, 1660, 1615, 1512, 1441, 1342, 1306, 1252, 1178, 1165, 1124, 1070, 1034, 835, 784, 697.
HRMS (TOF/ESI$^+$) Calculated for $C_{24}H_{24}O_2N_2F_3$ $[M+H]^+$ 429.17844 found 429.17877.
m.p. 117-120°C (toluene).

9. References

1 G. Kurtay, J. Lusseau, F. Robert and Y. Landais, *Synlett*, 2023, **35**, 342-346.

2 C. Ma, Y. Tian, J. Wang, X. He, Y. Jiang and B. Yu, *Org. Lett.*, 2022, **24**, 8265–8270.

3 M. Jouffroy and J. Kong, *Chem. Eur. J.*, 2019, **25**, 2217–2221.

4 K. Zhou, D. Wang, G. Ju, Z. Deng, P. Huang, Z. Huang, B. Li and Y. Zhao, *Org. Lett.*, 2022, **24**, 5568–5572.

5 S. Choi, A. B. Beeler, A. Pradhan, E. B. Watkins, J. M. Rimoldi, B. Tekwani and M. A. Avery, *J. Comb. Chem.*, 2007, **9**, 292–300.

6 Y. Yu, J. A. Deck, L. A. Hunsaker, L. M. Deck, R. E. Royer, E. Goldberg and D. L. Vander Jagt, *Biochemical Pharmacology*, 2001, **62**, 81–89.

7 G. G. Pawar, F. Robert, E. Grau, H. Cramail and Y. Landais, *Chem. Commun.*, 2018, **54**, 9337–9340.

8 I. M. Ogbu, J. Lusseau, G. Kurtay, F. Robert and Y. Landais, *Chem. Commun.*, 2020, **56**, 12226–12229.

9 J. Liu, B. Zhang, J. Hu, Z. Qiu, X. Chen, X. Tian, Q. Wang, G. Zheng and M. Yuan, *Eur. J. Org. Chem.*, 2023, **26**, e202201378.

10 S. V. Kumar and D. Ma, *J. Org. Chem.*, 2018, **83**, 2706–2713.

11 R. B. Strand, T. Helgerud, T. Solvang, C. A. Sperger and A. Fiksdahl, *Tetrahedron: Asymmetry*, 2011, **22**, 1994–2006.

12 S. Gambaro, C. Talotta, P. Della Sala, A. Soriente, M. De Rosa, C. Gaeta and P. Neri, *J. Am. Chem. Soc.*, 2020, **142**, 14914–14923.

13 L. Wang, C. Cao and C. Cao, *Magn. Reson. Chem.*, 2015, **53**, 520–525.

14 L. Cardinale, M.-O. W. S. Schmotz, M. O. Konev and A. Jacobi Von Wangelin, *Org. Lett.*, 2022, **24**, 506–510.

15 A. K. V. Mruthunjaya and A. A. J. Torriero, *Molecules*, 2023, **28**, 471.

16 H. Neuvonen, K. Neuvonen and F. Fülöp, *J. Org. Chem.*, 2006, **71**, 3141–3148.

17 Z. Li and J.-P. Cheng, *J. Org. Chem.*, 2003, **68**, 7350–7360.

18 I. Sasaki, T. Ikeda, T. Amou, J. Taguchi, H. Ito and T. Ishiyama, *Synlett*, 2016, **27**, 1582–1586.

10. NMR Spectra

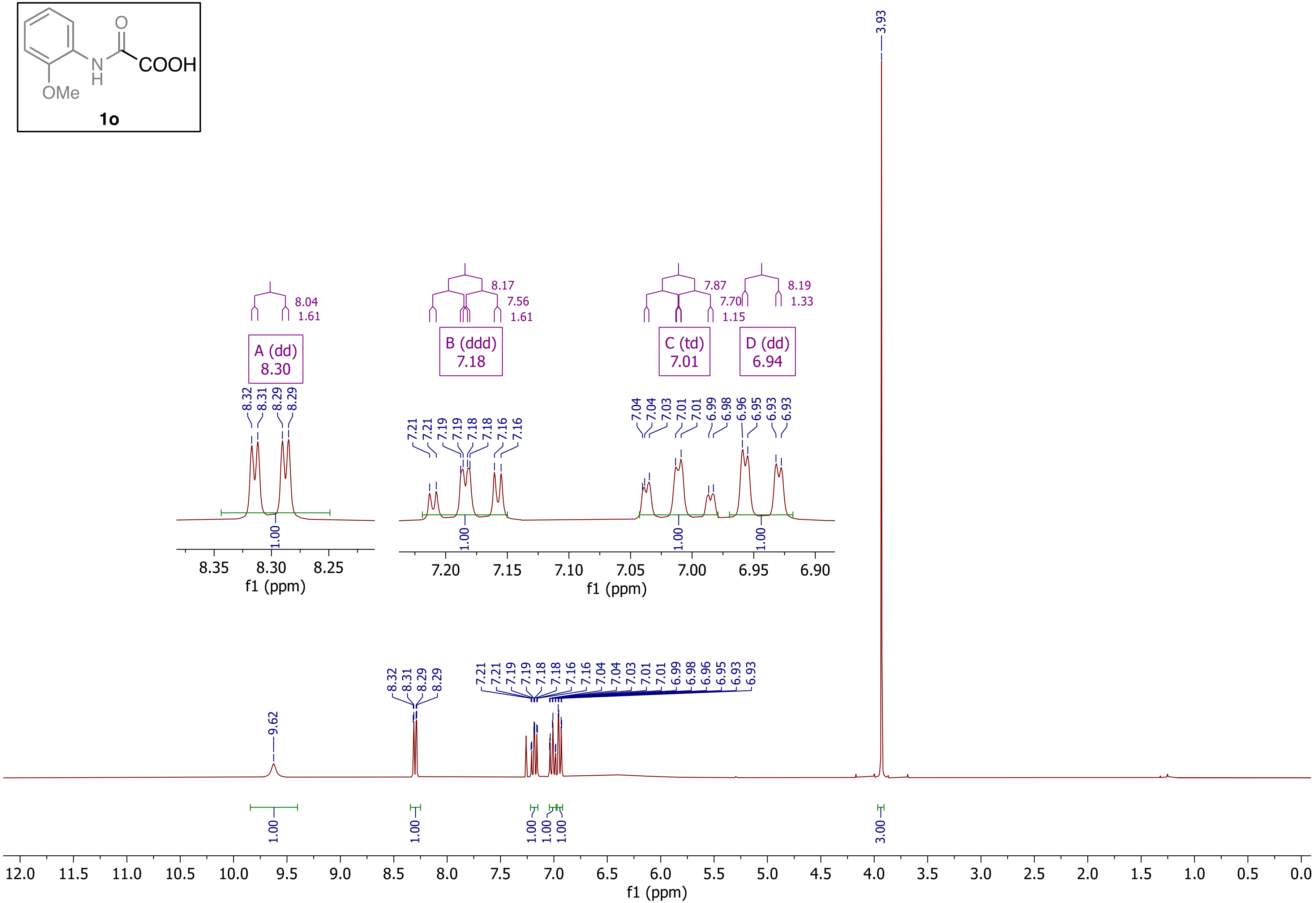
O
N
H
COOH
OMe
1o
A (dd)
8.30
8.04
1.61
B (ddd)
7.18
8.17
7.56
1.61
C (td)
7.01
7.87
7.70
1.15
D (dd)
6.94
8.19
1.33
3.93
9.62
8.32
8.31
8.29
8.29
7.21
7.21
7.19
7.19
7.18
7.18
7.16
7.16
7.04
7.04
7.03
7.01
7.01
6.99
6.98
6.96
6.95
6.93
6.93
1.00
1.00
1.00
1.00
1.00
3.00
f1 (ppm)

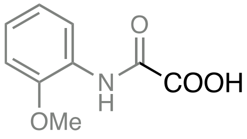
O
N
H
COOH
OMe

1o

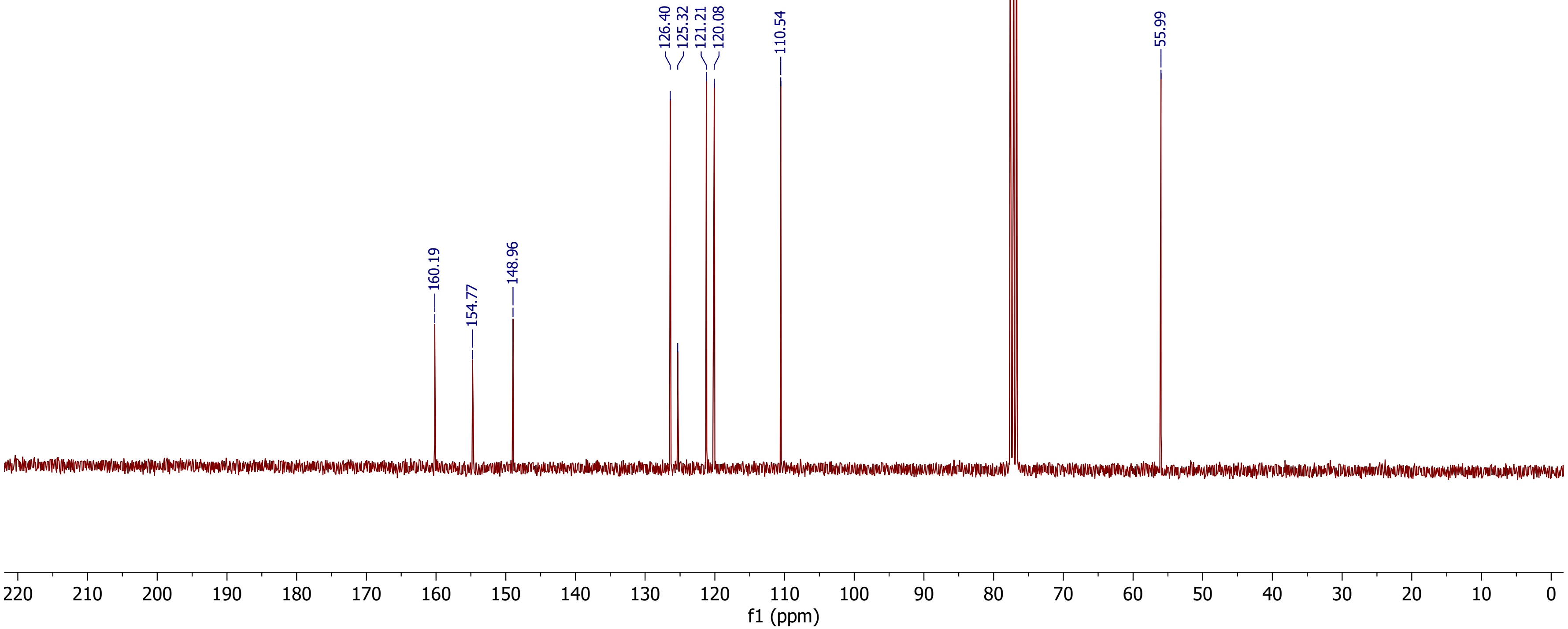
160.19
154.77
148.96
126.40
125.32
121.21
120.08
110.54
55.99
220 210 200 190 180 170 160 150 140 130 120 110 100 90 80 70 60 50 40 30 20 10 0
f1 (ppm)

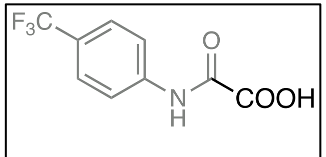
F3C
O
N
H
COOH

1q

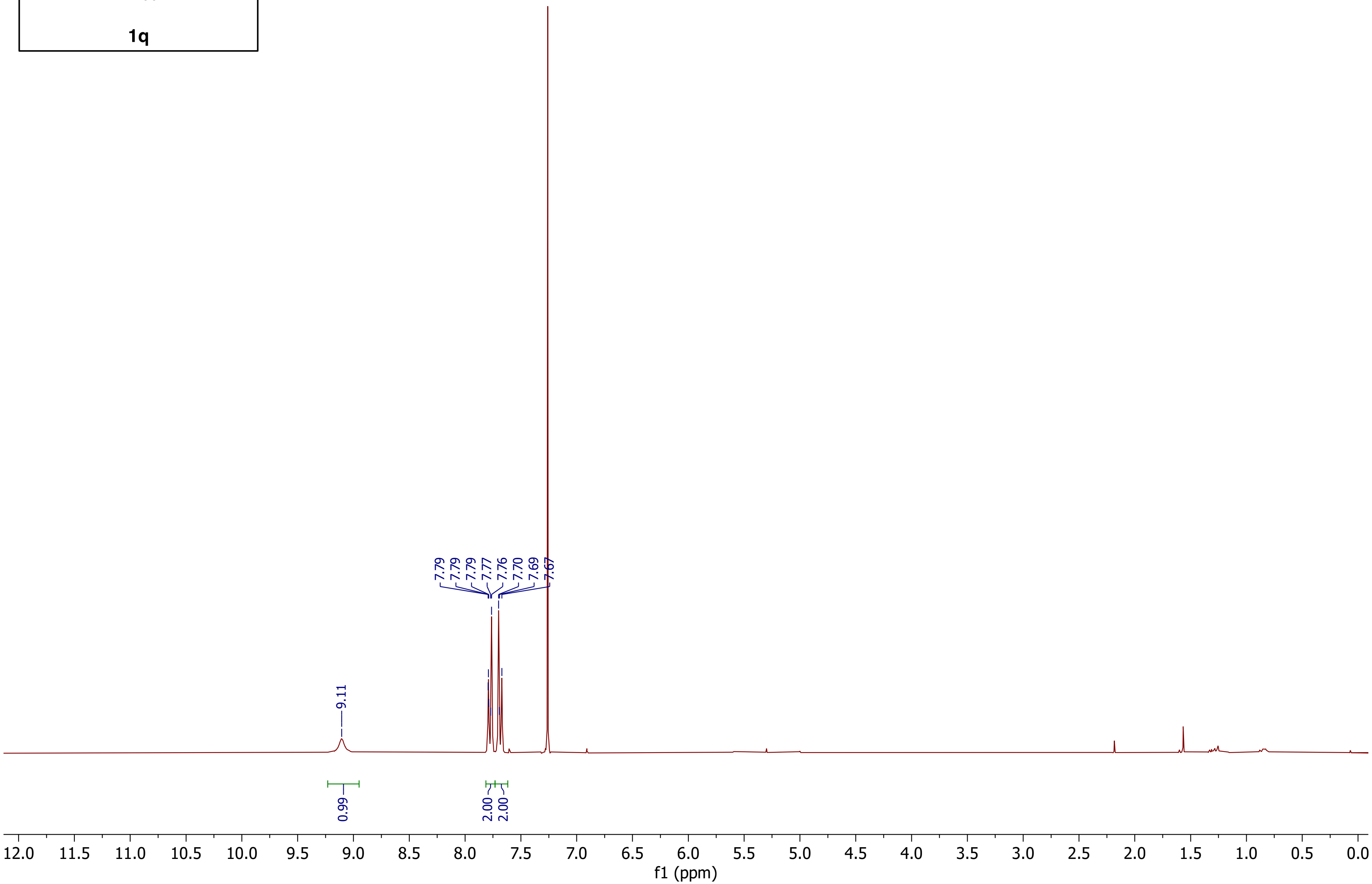
7.79
7.79
7.79
7.77
7.76
7.70
7.69
7.67
9.11
0.99
2.00
2.00
12.0 11.5 11.0 10.5 10.0 9.5 9.0 8.5 8.0 7.5 7.0 6.5 6.0 5.5 5.0 4.5 4.0 3.5 3.0 2.5 2.0 1.5 1.0 0.5 0.0
f1 (ppm)

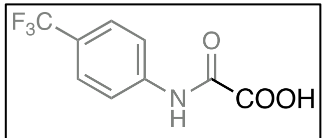
F3C
O
N
H
COOH

1q

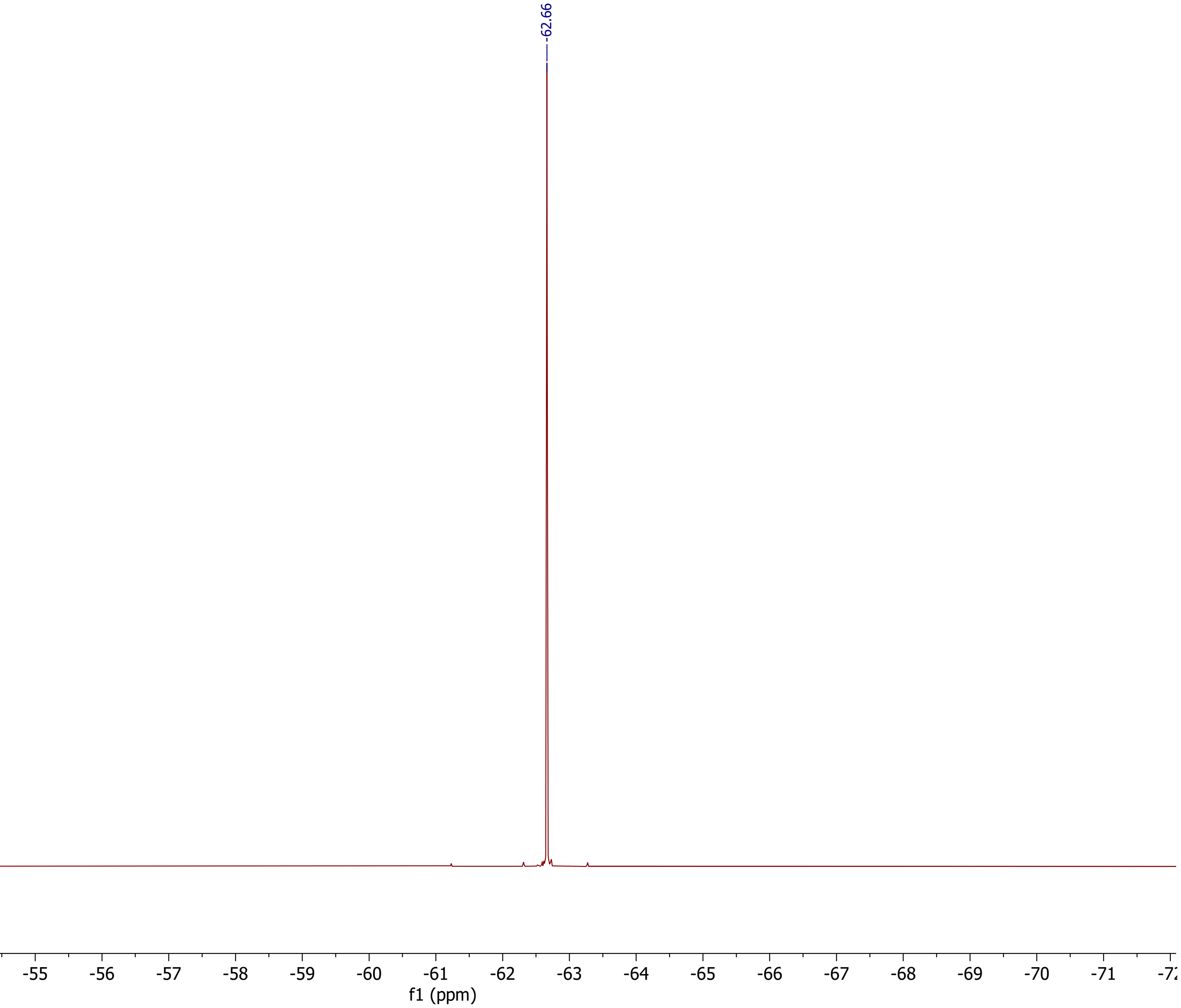
-62.66
-51
-52
-53
-54
-55
-56
-57
-58
-59
-60
-61
-62
-63
-64
-65
-66
-67
-68
-69
-70
-71
f1 (ppm)

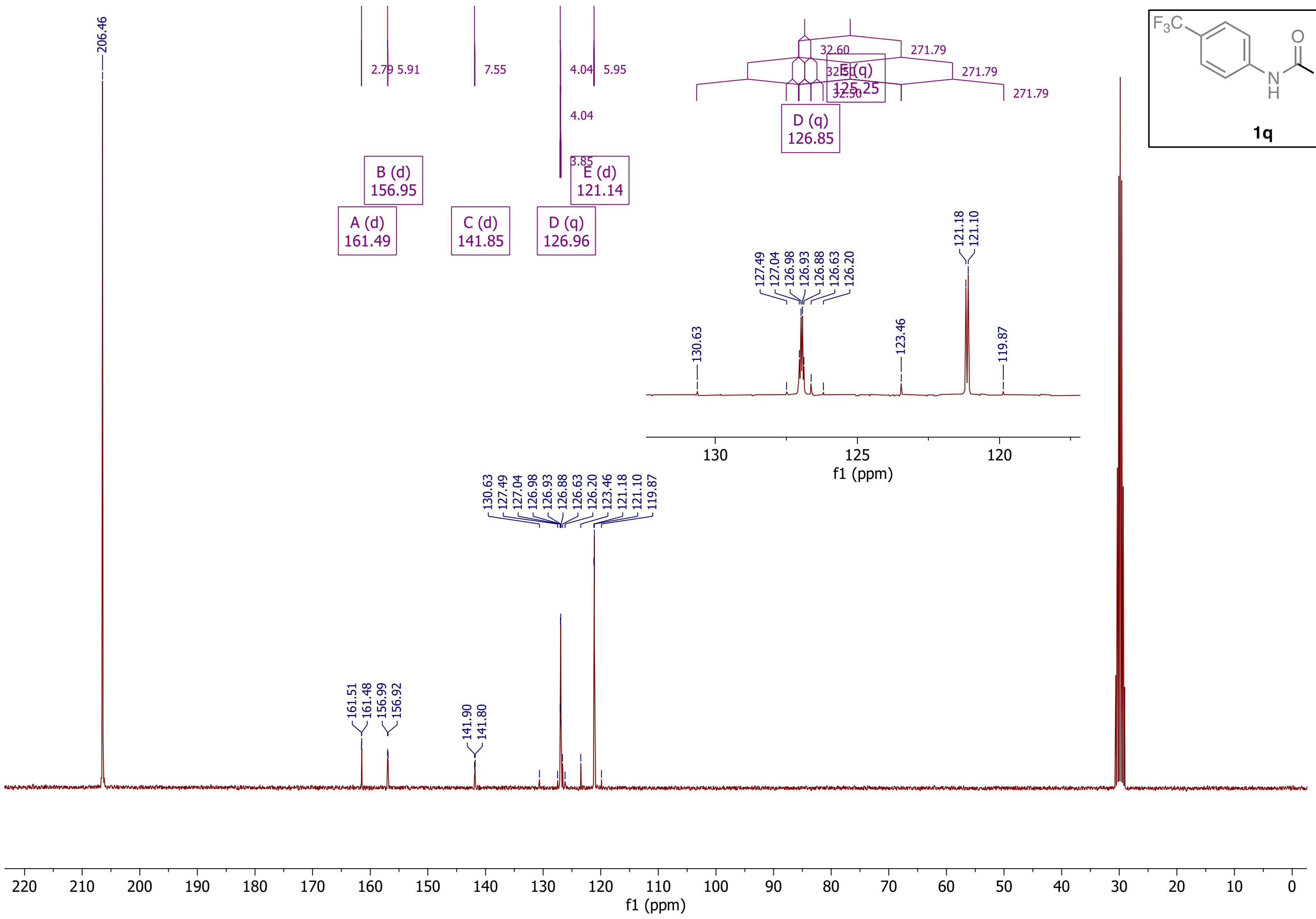
206.46
2.79 5.91
7.55
4.04 5.95
4.04
3.85
A (d) 161.49
B (d) 156.95
C (d) 141.85
D (q) 126.96
E (d) 121.14
32.60
271.79
E (q) 125.25
32.50
D (q) 126.85
127.49
127.04
126.98
126.93
126.88
126.63
126.20
121.18
121.10
130.63
123.46
119.87
130
125
120
f1 (ppm)
130.63
127.49
127.04
126.98
126.93
126.88
126.63
126.20
123.46
121.18
121.10
119.87
161.51
161.48
156.99
156.92
141.90
141.80
220
210
200
190
180
170
160
150
140
130
120
110
100
90
80
70
60
50
40
30
20
10
0
f1 (ppm)
F3C
O
N
H
COOH

1q

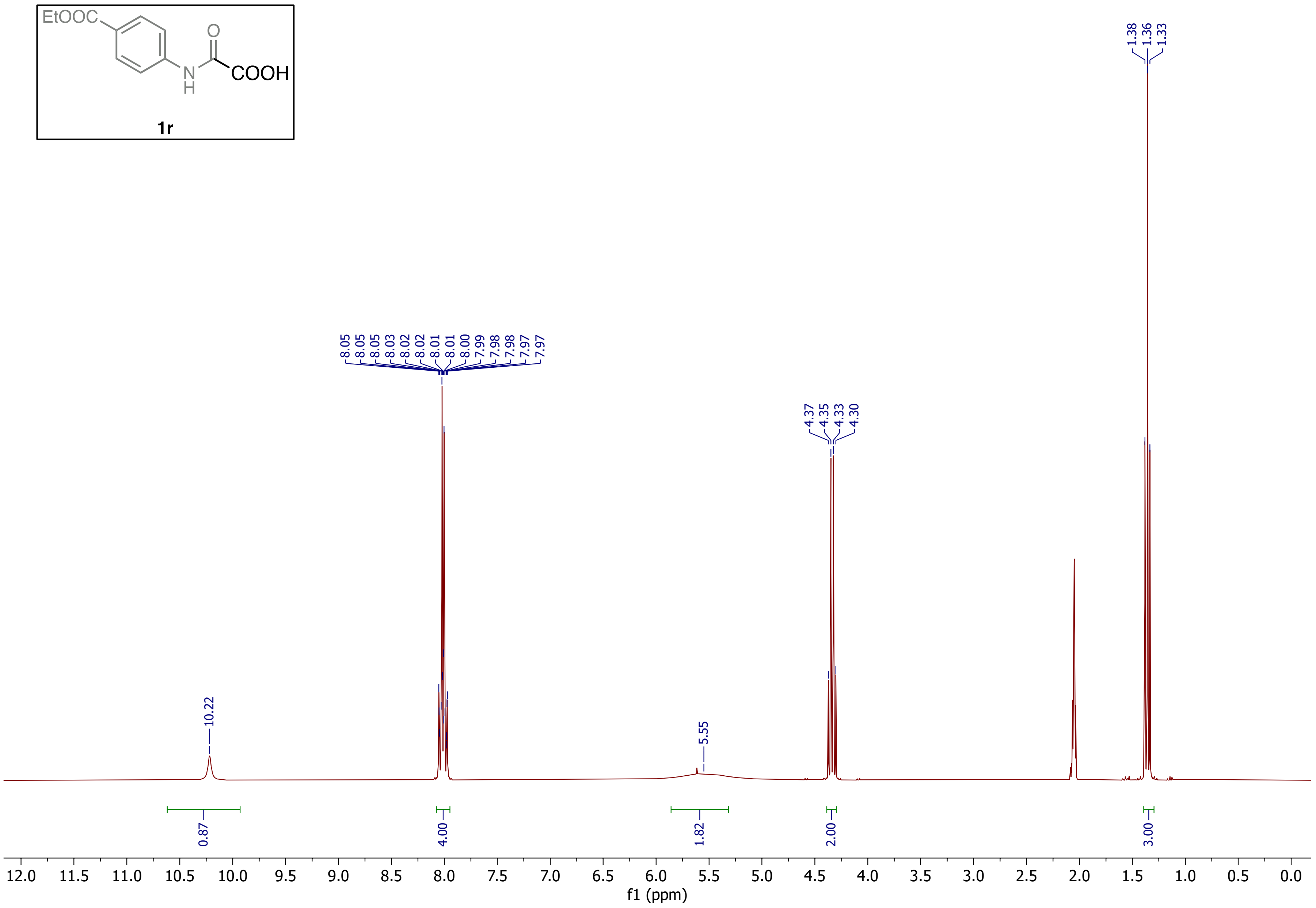

EtOOC
O
N
H
COOH
1r
8.05
8.05
8.05
8.03
8.02
8.02
8.01
8.01
8.00
7.99
7.98
7.98
7.97
7.97
4.37
4.35
4.33
4.30
1.38
1.36
1.33
10.22
5.55
0.87
4.00
1.82
2.00
3.00
12.0 11.5 11.0 10.5 10.0 9.5 9.0 8.5 8.0 7.5 7.0 6.5 6.0 5.5 5.0 4.5 4.0 3.5 3.0 2.5 2.0 1.5 1.0 0.5 0.0
f1 (ppm)

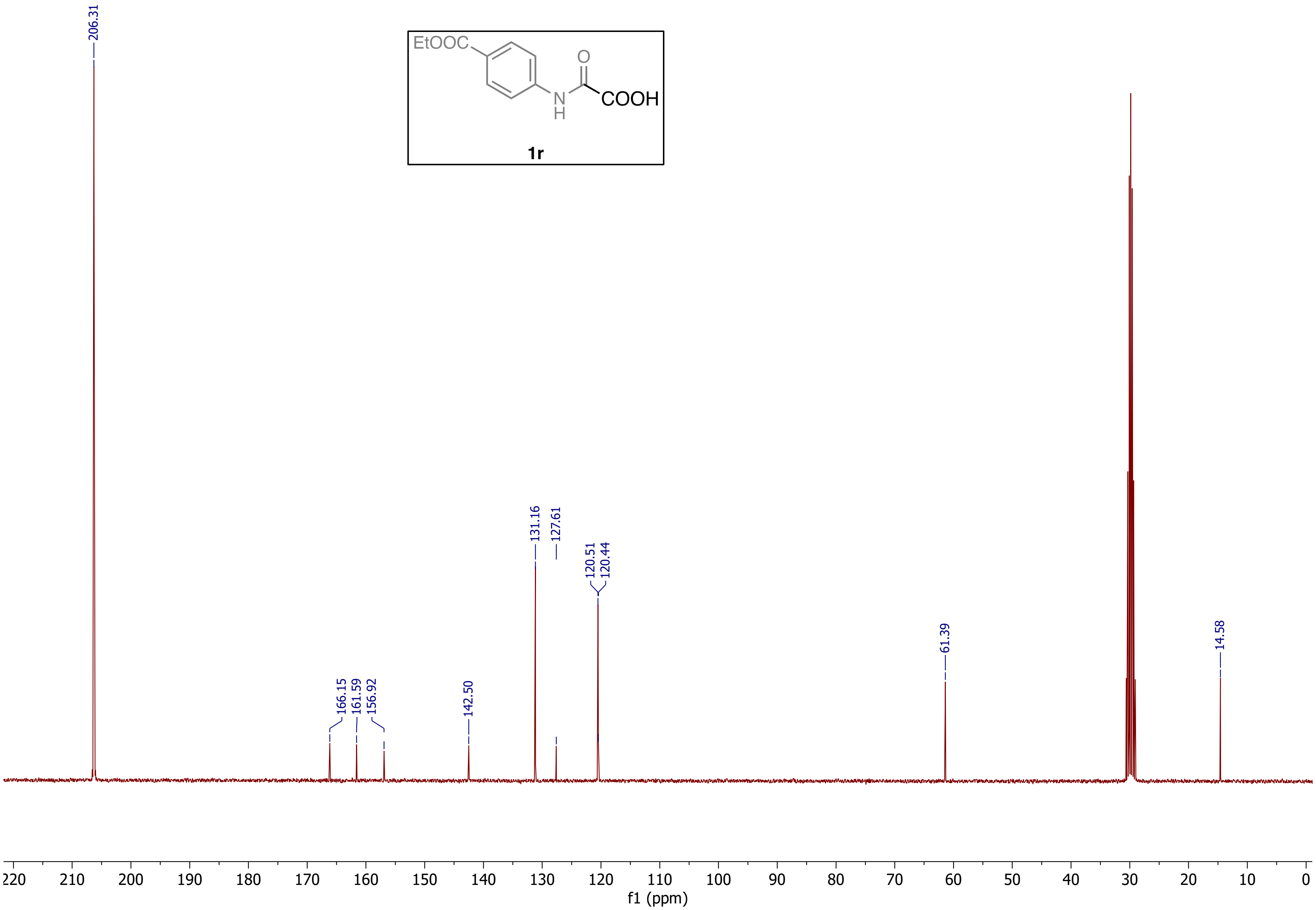

206.31
EtOOC
O
N
H
COOH
1r
166.15
161.59
156.92
142.50
131.16
127.61
120.51
120.44
61.39
14.58
220 210 200 190 180 170 160 150 140 130 120 110 100 90 80 70 60 50 40 30 20 10 0
f1 (ppm)

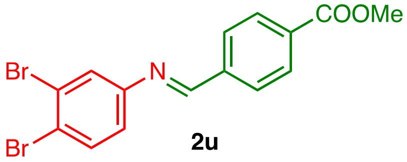
COOMe
Br
Br
N

2u

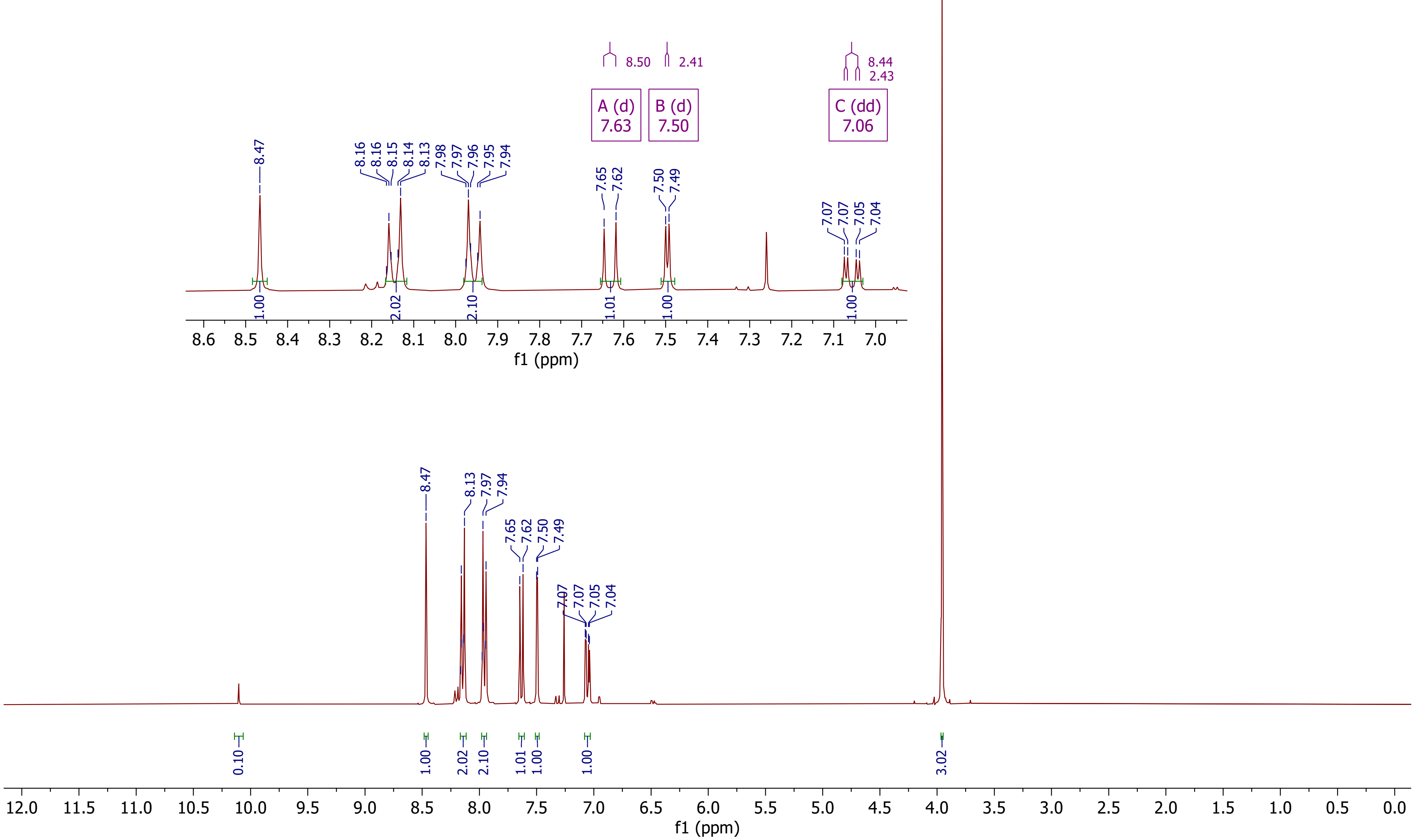
8.50
2.41
8.44
2.43
A (d)
7.63
B (d)
7.50
C (dd)
7.06
8.47
8.16
8.16
8.15
8.14
8.13
7.98
7.97
7.96
7.95
7.94
7.65
7.62
7.50
7.49
7.07
7.07
7.05
7.04
1.00
2.02
2.10
1.01
1.00
1.00
8.6 8.5 8.4 8.3 8.2 8.1 8.0 7.9 7.8 7.7 7.6 7.5 7.4 7.3 7.2 7.1 7.0
f1 (ppm)
3.96
8.47
8.13
7.97
7.94
7.65
7.62
7.50
7.49
7.07
7.07
7.05
7.04
0.10
1.00
2.02
2.10
1.01
1.00
1.00
3.02
12.0 11.5 11.0 10.5 10.0 9.5 9.0 8.5 8.0 7.5 7.0 6.5 6.0 5.5 5.0 4.5 4.0 3.5 3.0 2.5 2.0 1.5 1.0 0.5 0.0
f1 (ppm)

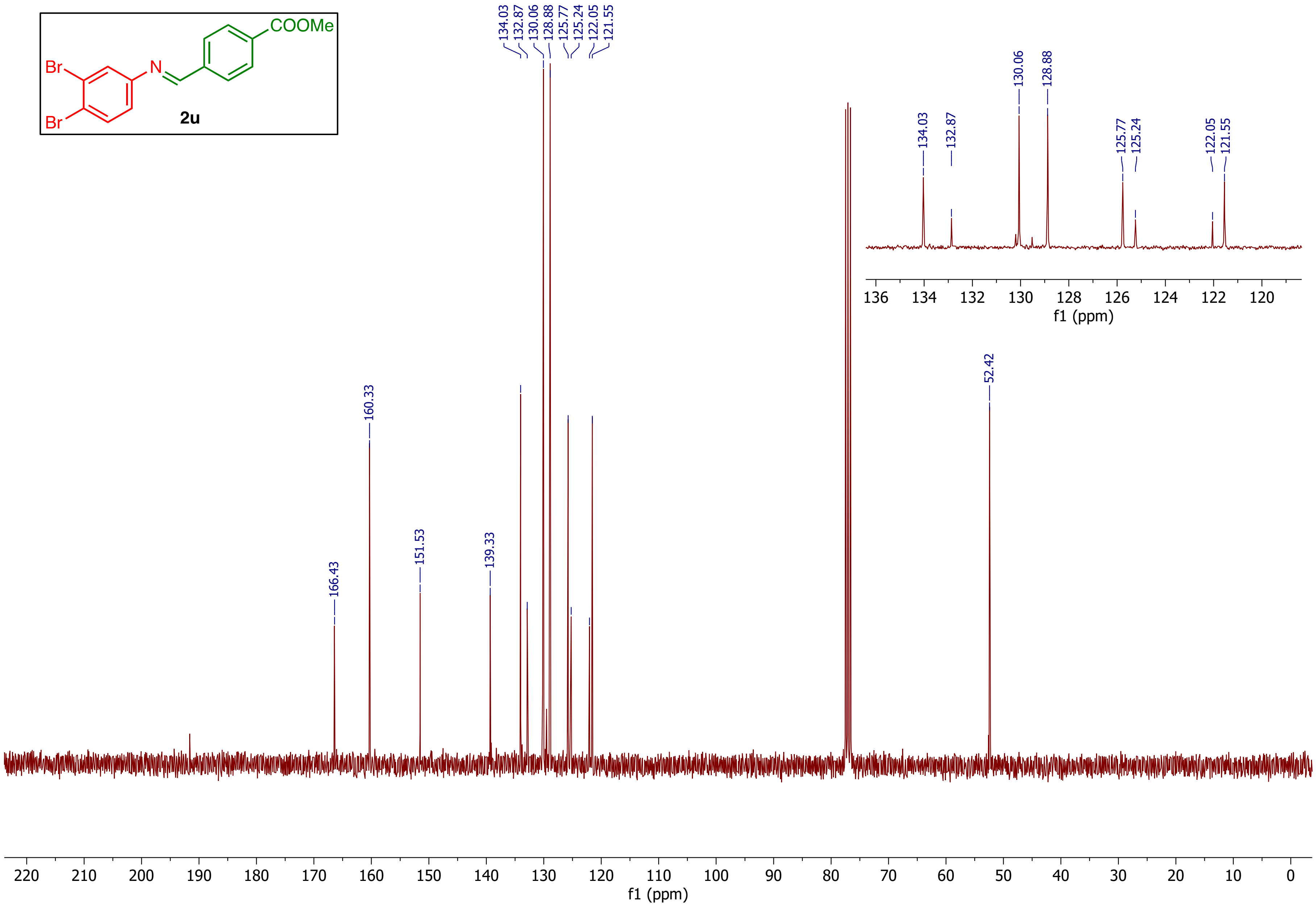

COOMe
Br
Br
N
2u
134.03
132.87
130.06
128.88
125.77
125.24
122.05
121.55
166.43
160.33
151.53
139.33
52.42
136 134 132 130 128 126 124 122 120
f1 (ppm)
220 210 200 190 180 170 160 150 140 130 120 110 100 90 80 70 60 50 40 30 20 10 0
f1 (ppm)

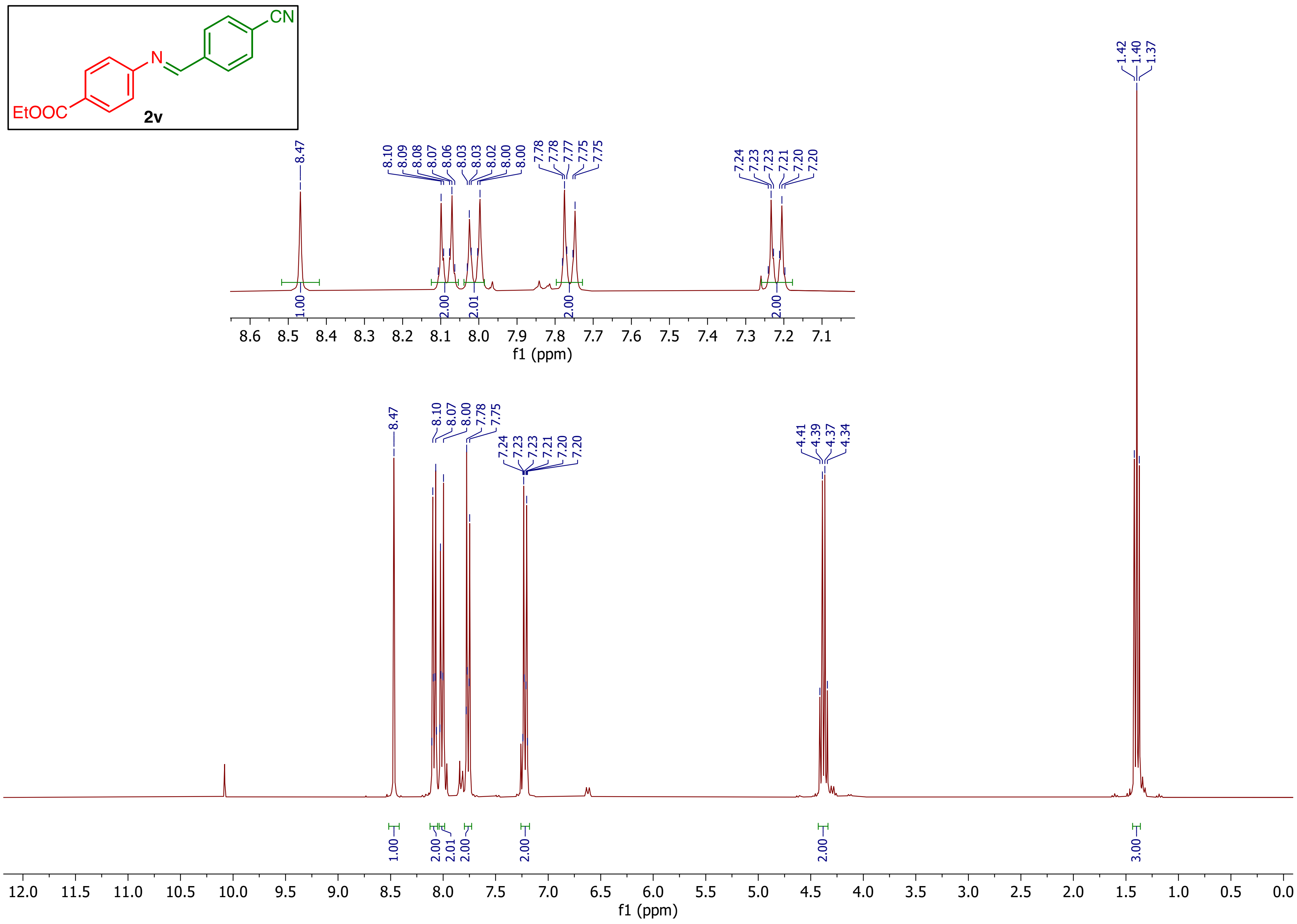
EtOOC
N
CN
2v
f1 (ppm)

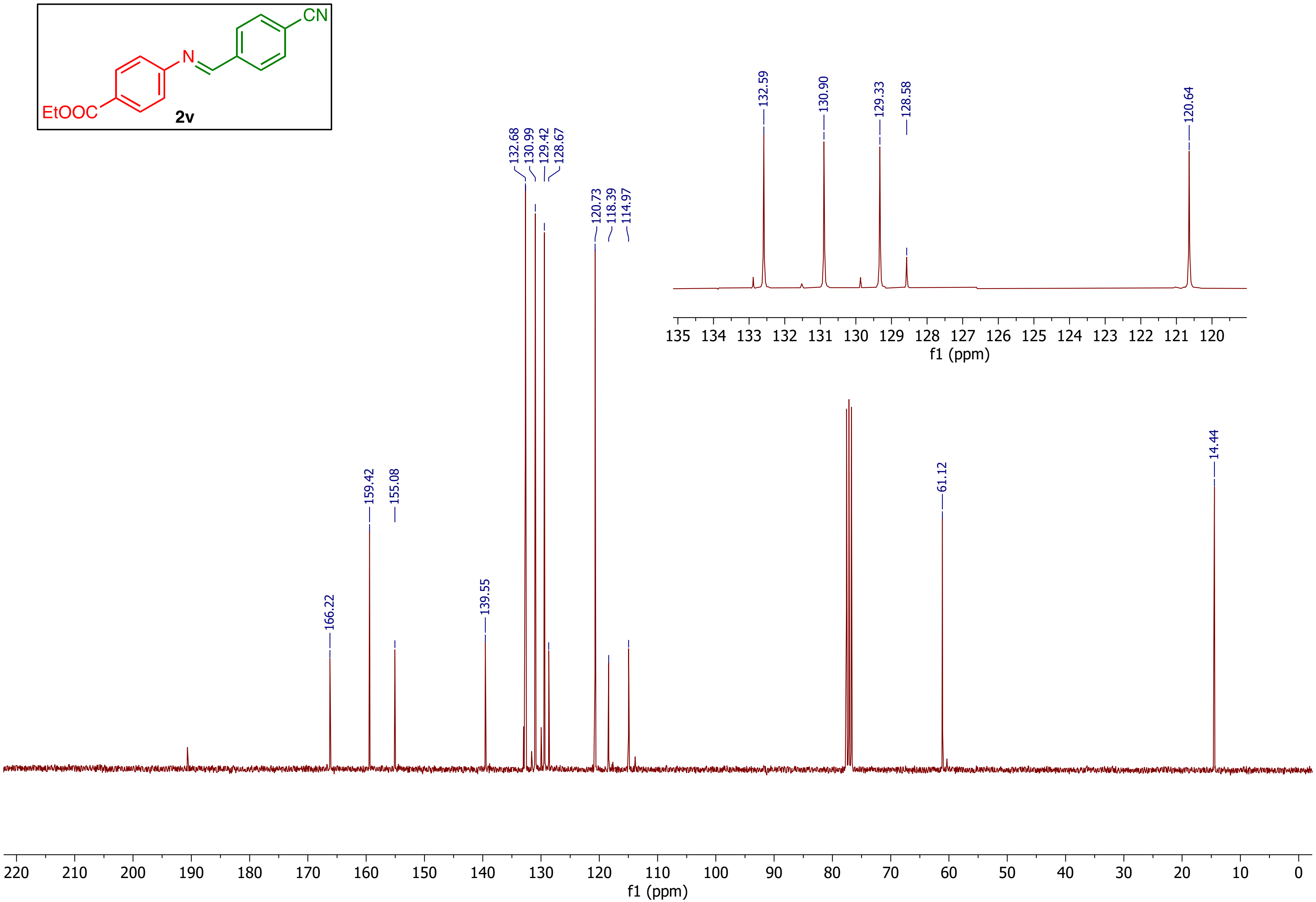

CN
N
EtOOC
2v
132.68
130.99
129.42
128.67
120.73
118.39
114.97
159.42
155.08
166.22
139.55
61.12
14.44
132.59
130.90
129.33
128.58
120.64
135 134 133 132 131 130 129 128 127 126 125 124 123 122 121 120
f1 (ppm)
220 210 200 190 180 170 160 150 140 130 120 110 100 90 80 70 60 50 40 30 20 10 0
f1 (ppm)

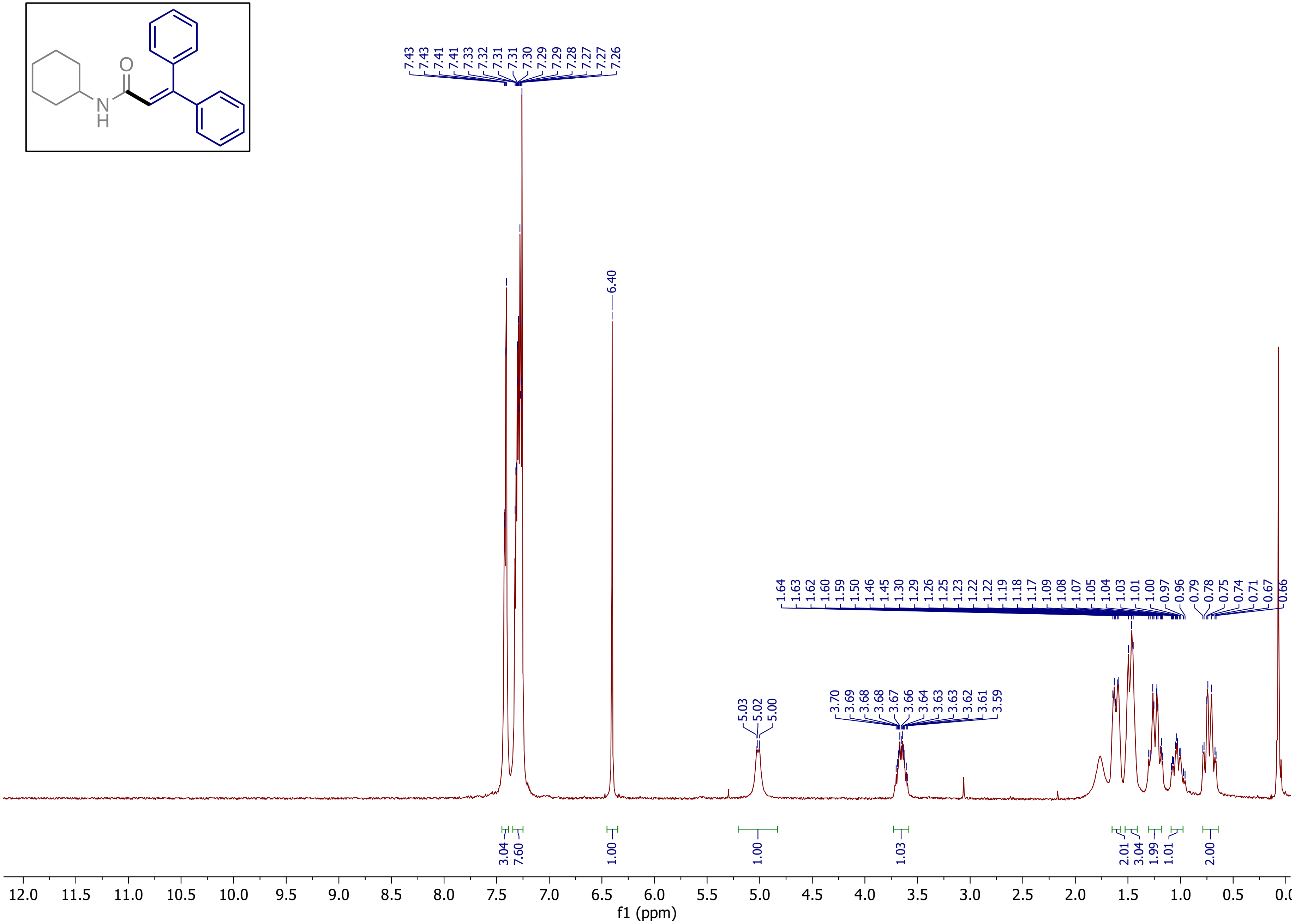
O
N
H
7.43
7.43
7.41
7.41
7.33
7.32
7.31
7.31
7.30
7.29
7.29
7.28
7.27
7.27
7.26
6.40
5.03
5.02
5.00
3.70
3.69
3.68
3.68
3.67
3.66
3.64
3.63
3.63
3.62
3.61
3.59
1.64
1.63
1.62
1.60
1.59
1.50
1.46
1.45
1.30
1.29
1.26
1.25
1.23
1.22
1.22
1.19
1.18
1.17
1.09
1.08
1.07
1.05
1.04
1.03
1.01
1.00
0.97
0.96
0.79
0.78
0.75
0.74
0.71
0.67
0.66
3.04
7.60
1.00
1.00
1.03
2.01
3.04
1.99
1.01
2.00
12.0
11.5
11.0
10.5
10.0
9.5
9.0
8.5
8.0
7.5
7.0
6.5
6.0
5.5
5.0
4.5
4.0
3.5
3.0
2.5
2.0
1.5
1.0
0.5
f1 (ppm)

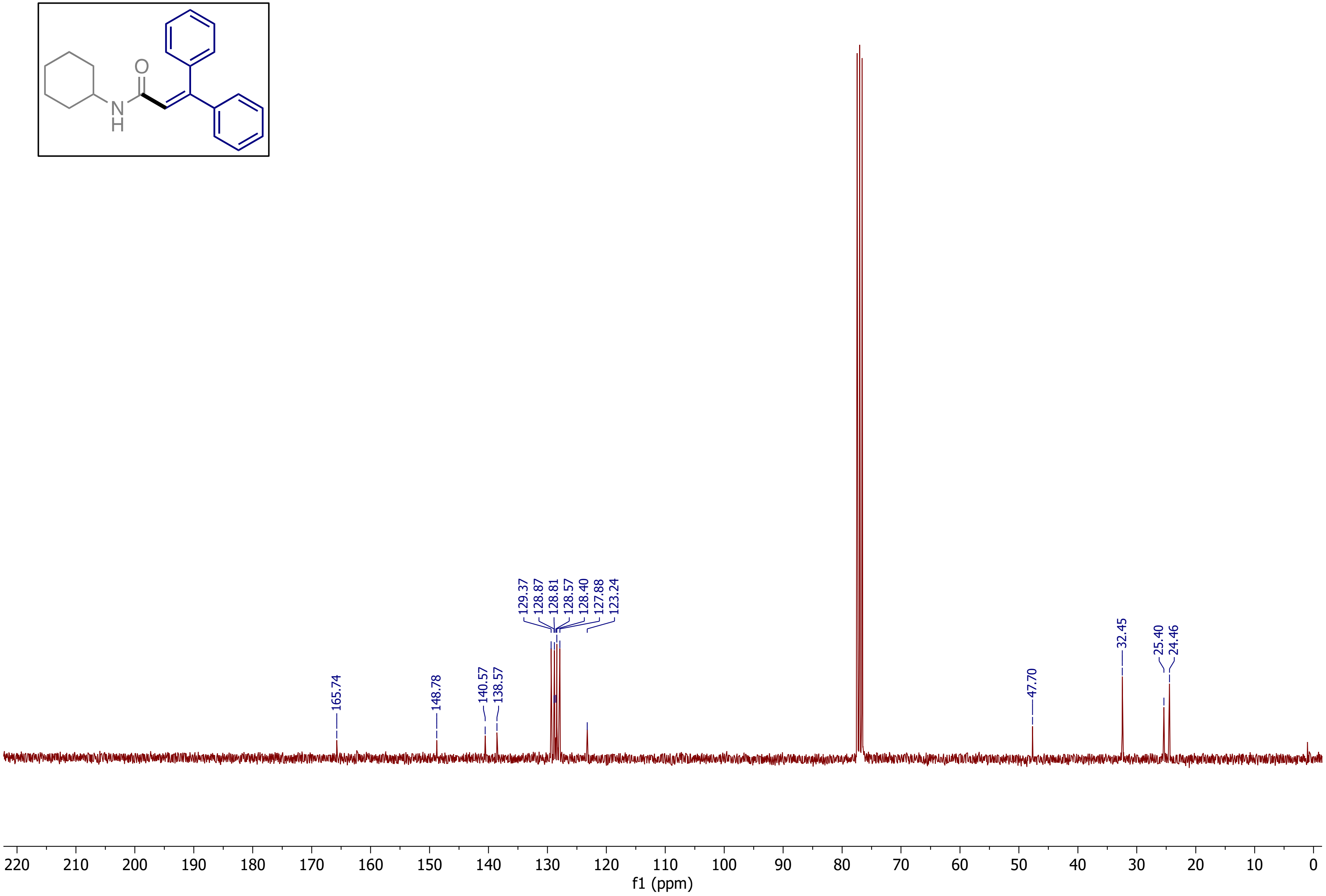
O
N
H
165.74
148.78
140.57
138.57
129.37
128.87
128.81
128.57
128.40
127.88
123.24
47.70
32.45
25.40
24.46
220 210 200 190 180 170 160 150 140 130 120 110 100 90 80 70 60 50 40 30 20 10 0
f1 (ppm)

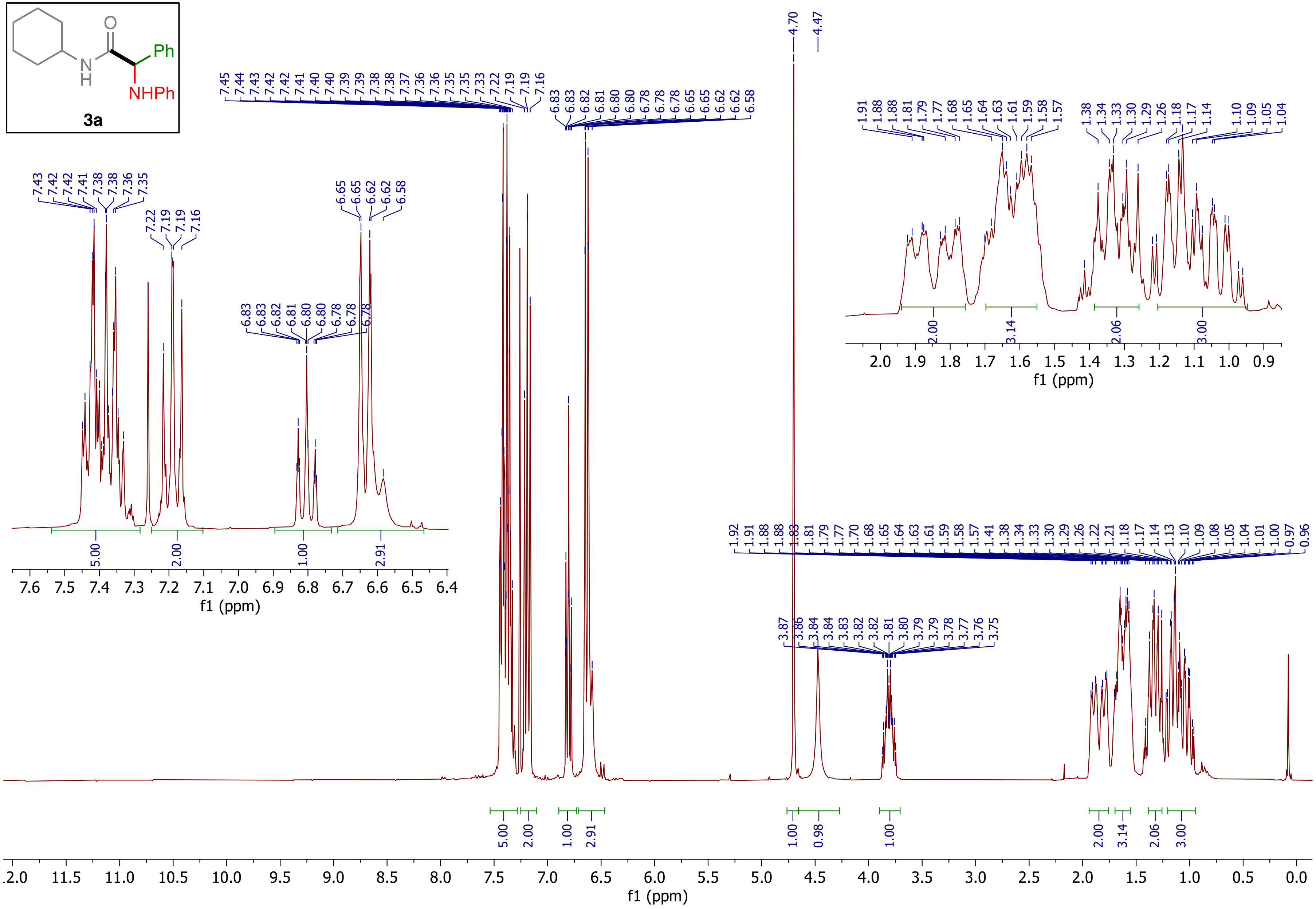

NHPh
Ph
3a
f1 (ppm)

ME
13(

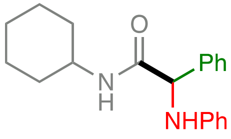
O
N
H
Ph
NHPh

3a

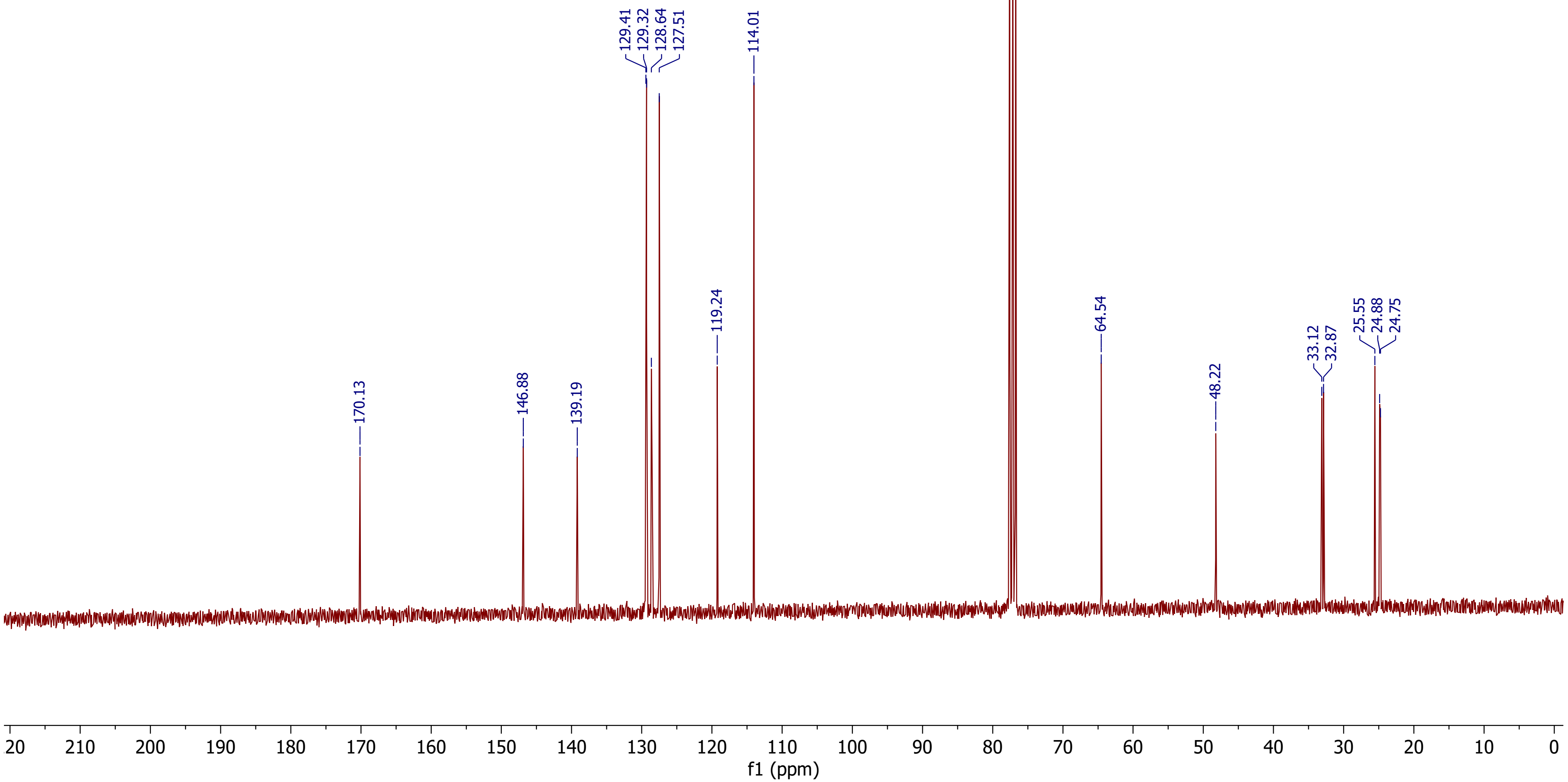
170.13
146.88
139.19
129.41
129.32
128.64
127.51
119.24
114.01
64.54
48.22
33.12
32.87
25.55
24.88
24.75
220 210 200 190 180 170 160 150 140 130 120 110 100 90 80 70 60 50 40 30 20 10 0
f1 (ppm)

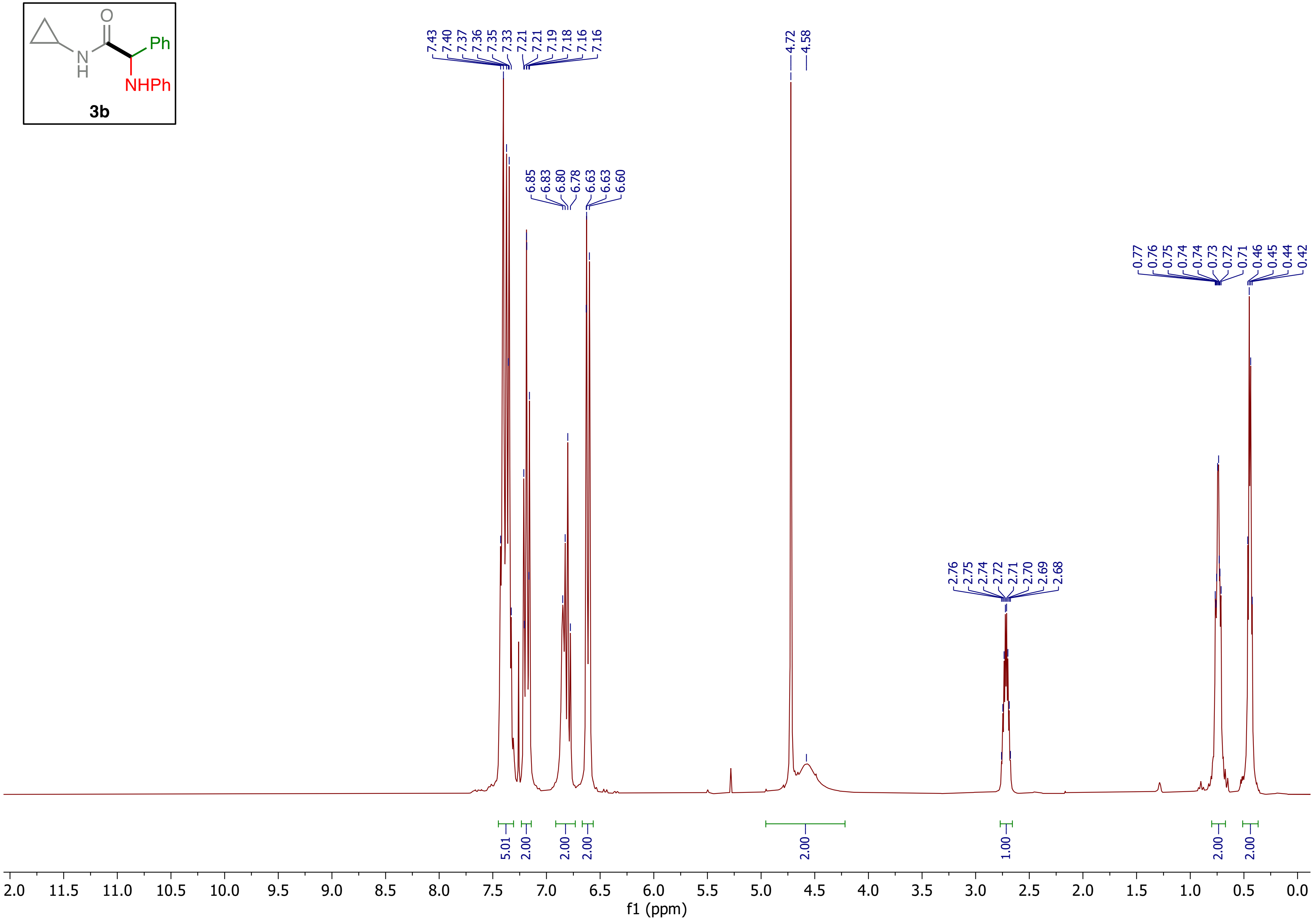

3b
Ph
NHPh
7.43 7.40 7.37 7.36 7.35 7.33 7.21 7.21 7.19 7.18 7.16 7.16
4.72 4.58
6.85 6.83 6.80 6.78 6.63 6.63 6.60
0.77 0.76 0.75 0.74 0.74 0.73 0.72 0.71 0.46 0.45 0.44 0.42
2.76 2.75 2.74 2.72 2.71 2.70 2.69 2.68
5.01 2.00 2.00 2.00 2.00 1.00 2.00 2.00
f1 (ppm)

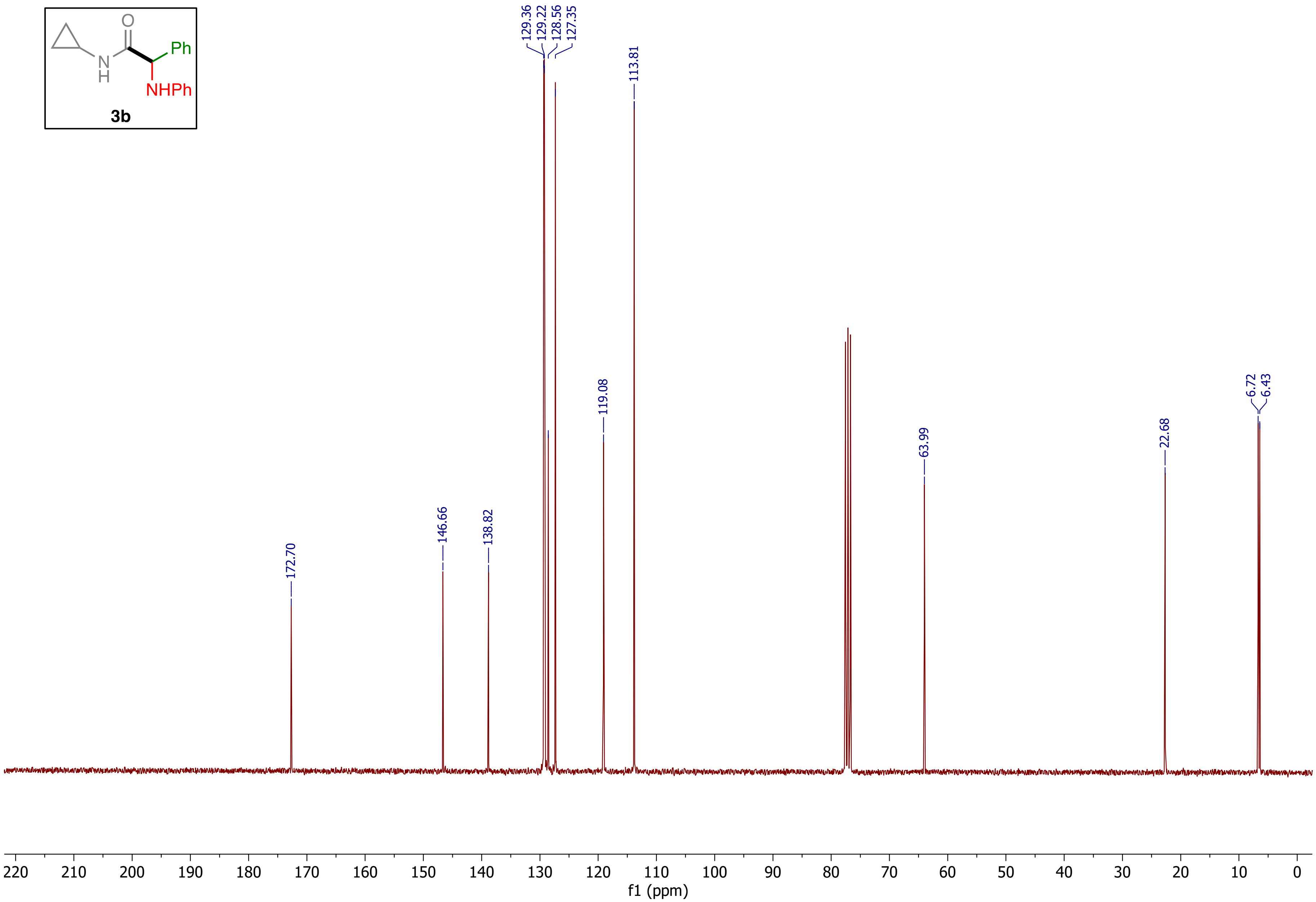
O
N
H
Ph
NHPh
3b
129.36
129.22
128.56
127.35
113.81
172.70
146.66
138.82
119.08
63.99
22.68
6.72
6.43
220 210 200 190 180 170 160 150 140 130 120 110 100 90 80 70 60 50 40 30 20 10 0
f1 (ppm)

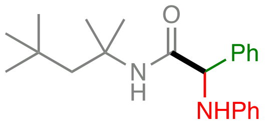

O
N
H
Ph
NHPh

3c

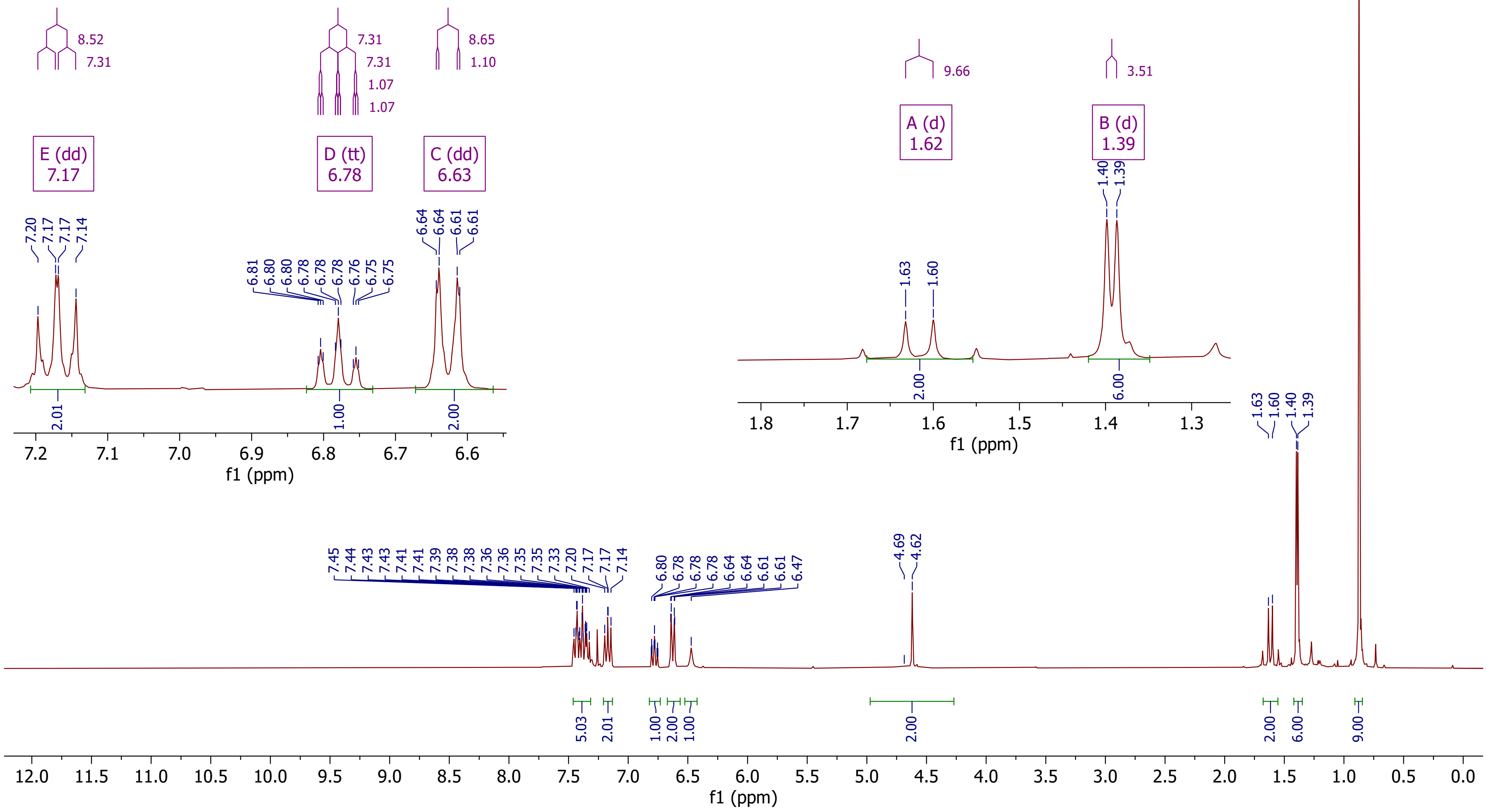

E (dd) 7.17
D (tt) 6.78
C (dd) 6.63
A (d) 1.62
B (d) 1.39
8.52
7.31
7.31
7.31
1.07
1.07
8.65
1.10
9.66
3.51
0.87
f1 (ppm)

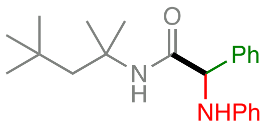
O
N
H
Ph
NHPh

3c

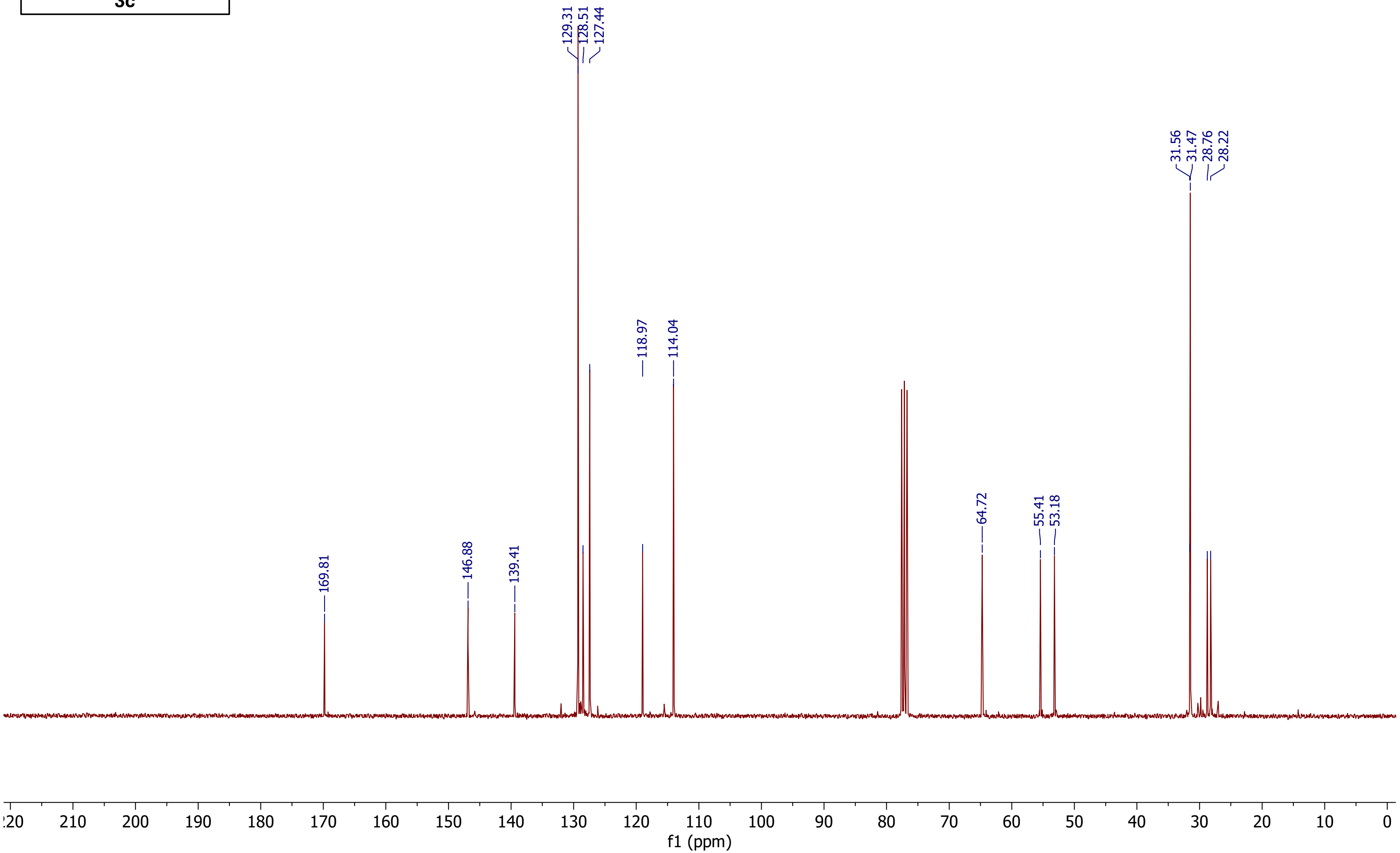
169.81
146.88
139.41
129.31
128.51
127.44
118.97
114.04
64.72
55.41
53.18
31.56
31.47
28.76
28.22
220
210
200
190
180
170
160
150
140
130
120
110
100
90
80
70
60
50
40
30
20
10
0
f1 (ppm)

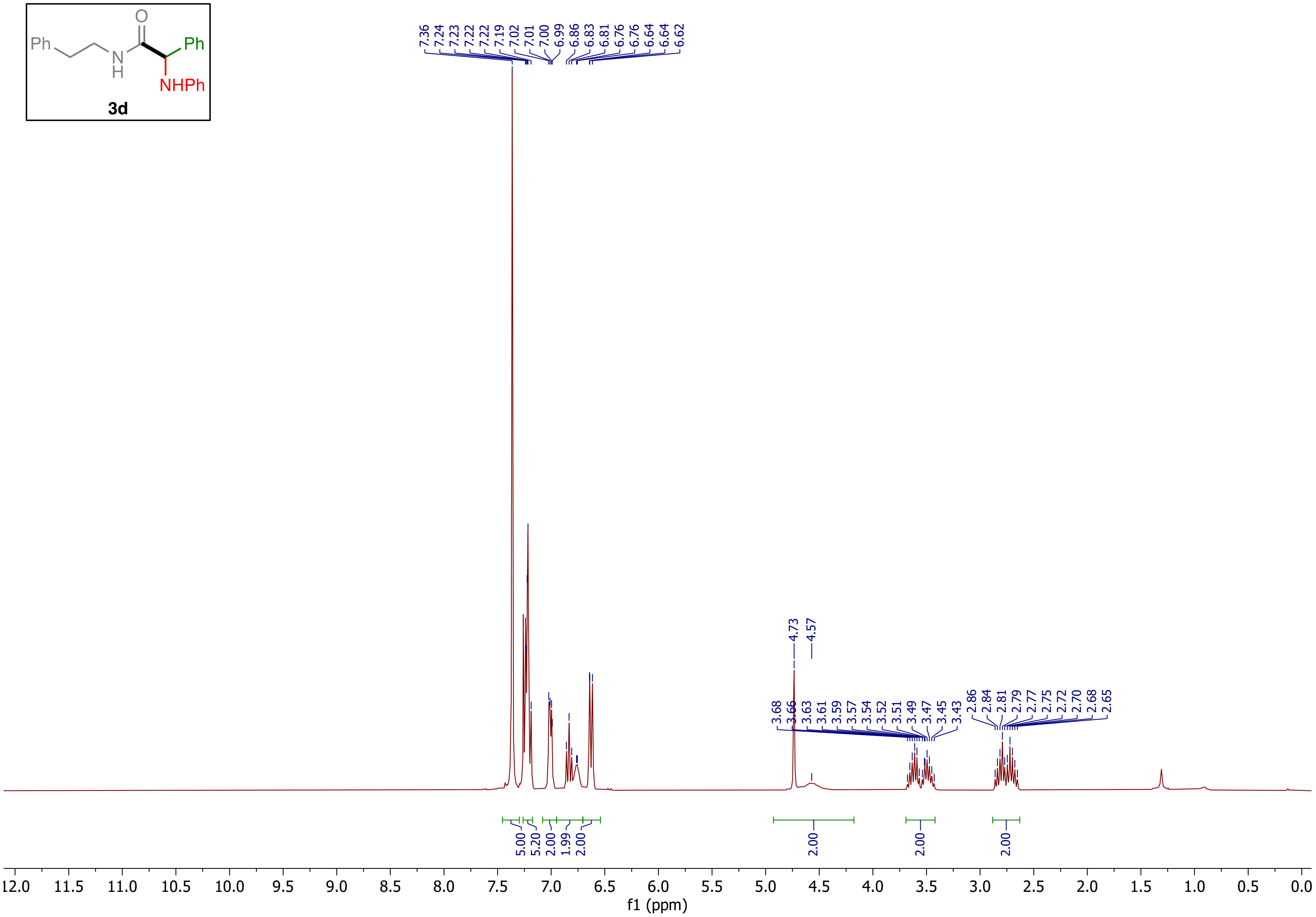

Ph
O
N
H
Ph
NHPh
3d
7.36 7.24 7.23 7.22 7.22 7.19 7.02 7.01 7.00 6.99 6.86 6.83 6.81 6.76 6.76 6.64 6.64 6.62
4.73
4.57
3.68 3.66 3.63 3.61 3.59 3.57 3.54 3.52 3.51 3.49 3.47 3.45 3.43
2.86 2.84 2.81 2.79 2.77 2.75 2.72 2.70 2.68 2.65
5.00 5.20 2.00 1.99 2.00
2.00
2.00
2.00
12.0 11.5 11.0 10.5 10.0 9.5 9.0 8.5 8.0 7.5 7.0 6.5 6.0 5.5 5.0 4.5 4.0 3.5 3.0 2.5 2.0 1.5 1.0 0.5 0.0
f1 (ppm)

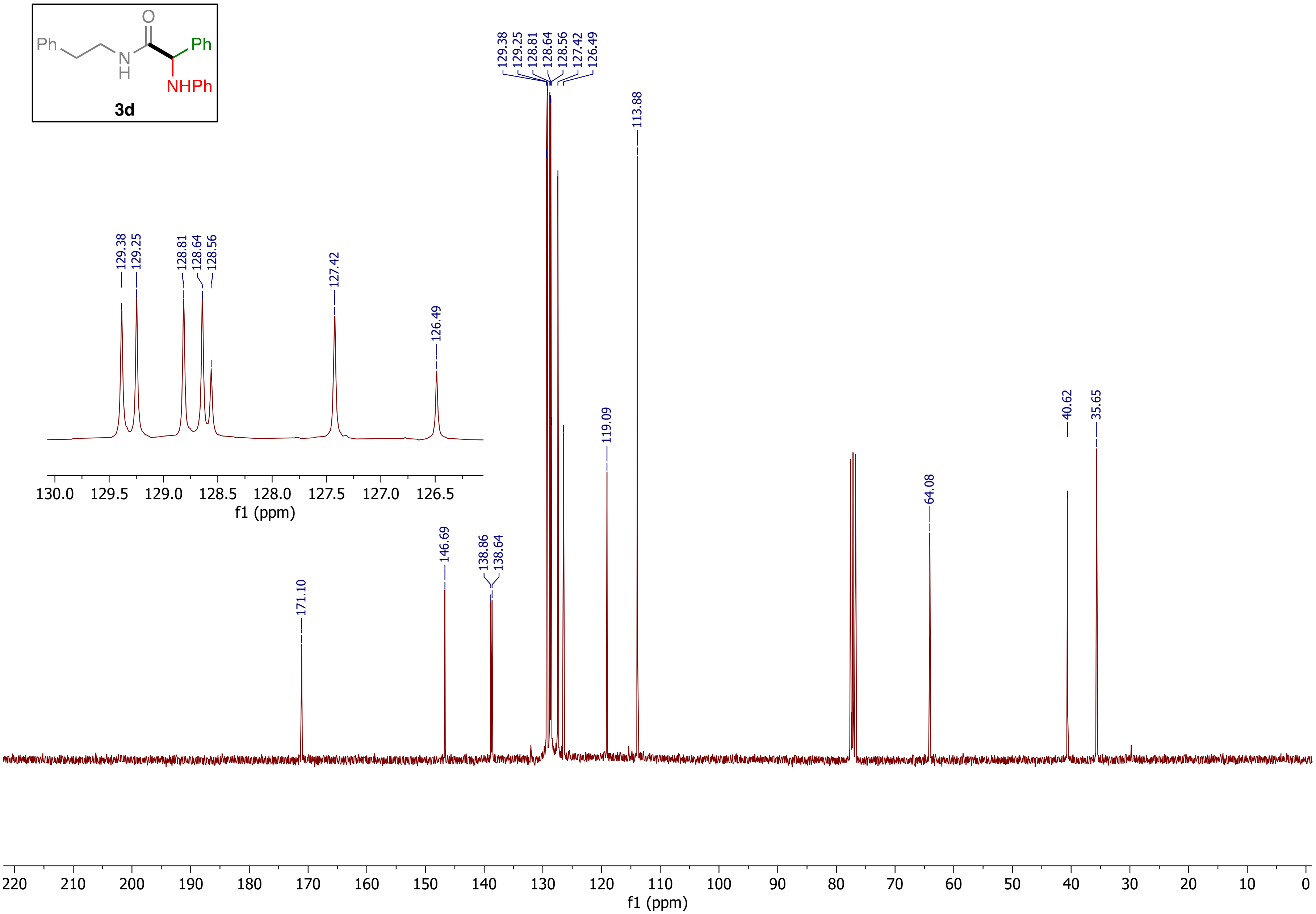

Ph
O
N
H
Ph
NHPh
3d
129.38
129.25
128.81
128.64
128.56
127.42
126.49
113.88
119.09
171.10
146.69
138.86
138.64
64.08
40.62
35.65
130.0 129.5 129.0 128.5 128.0 127.5 127.0 126.5
f1 (ppm)
220 210 200 190 180 170 160 150 140 130 120 110 100 90 80 70 60 50 40 30 20 10 0
f1 (ppm)

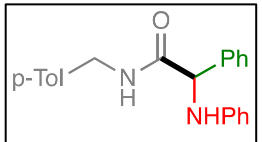
O
p-Tol
N
H
Ph
NHPh

3e

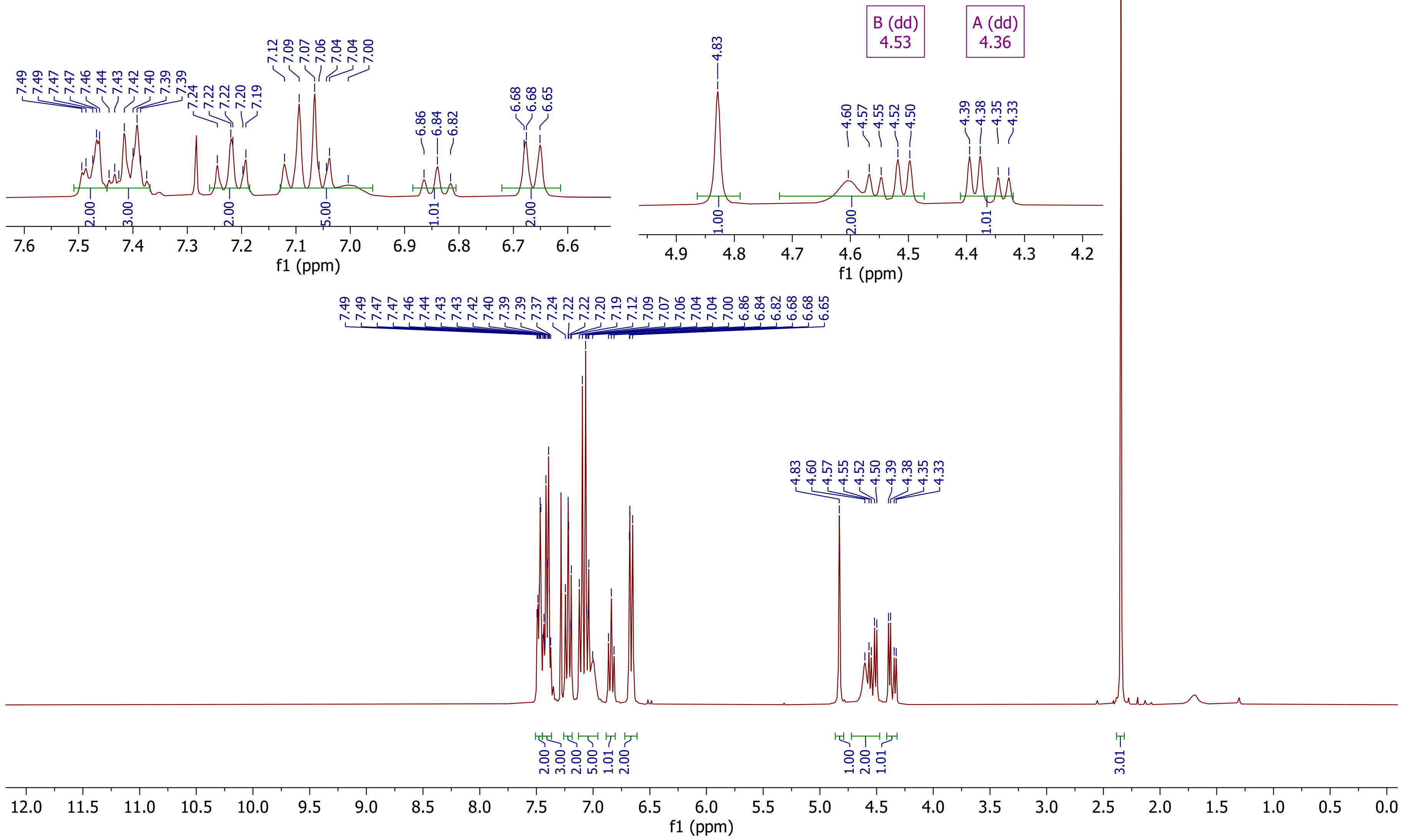
2.34
7.49 7.49 7.47 7.47 7.46 7.44 7.43 7.43 7.42 7.40 7.39 7.39 7.37 7.24 7.22 7.22 7.20 7.19 7.12 7.09 7.07 7.06 7.04 7.04 7.00 6.86 6.84 6.82 6.68 6.68 6.65
4.83 4.60 4.57 4.55 4.52 4.50 4.39 4.38 4.35 4.33
B (dd)
4.53
14.79
6.18
A (dd)
4.36
14.79
5.49
2.00
3.00
2.00
5.00
1.01
2.00
1.00
2.00
1.01
3.01
f1 (ppm)

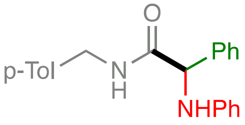
O
p-Tol
N
H
Ph
NHPh
3e

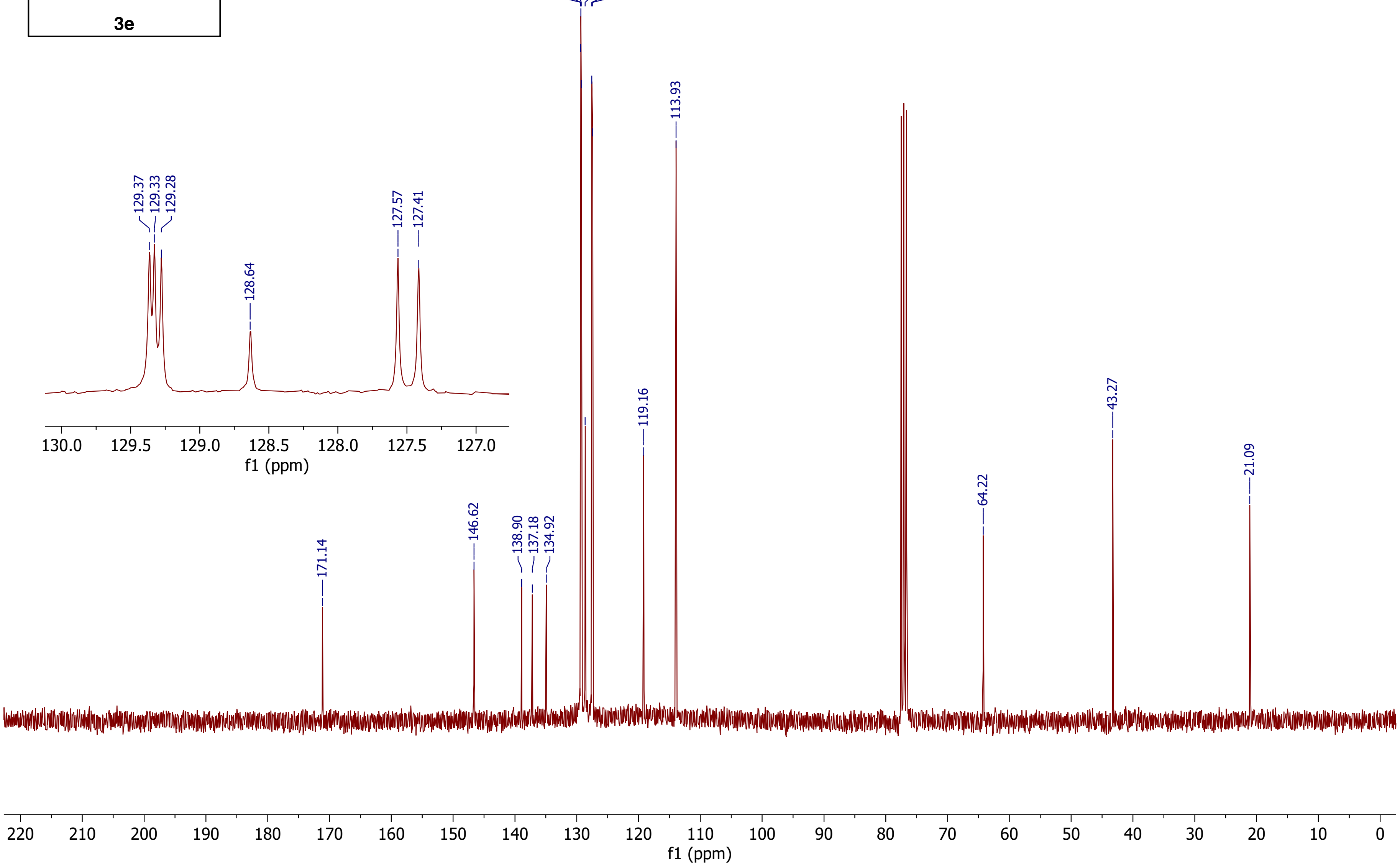
129.37
129.33
129.28
128.64
127.57
127.41
113.93
119.16
171.14
146.62
138.90
137.18
134.92
64.22
43.27
21.09
130.0
129.5
129.0
128.5
128.0
127.5
127.0
f1 (ppm)
220
210
200
190
180
170
160
150
140
130
120
110
100
90
80
70
60
50
40
30
20
10
0
f1 (ppm)

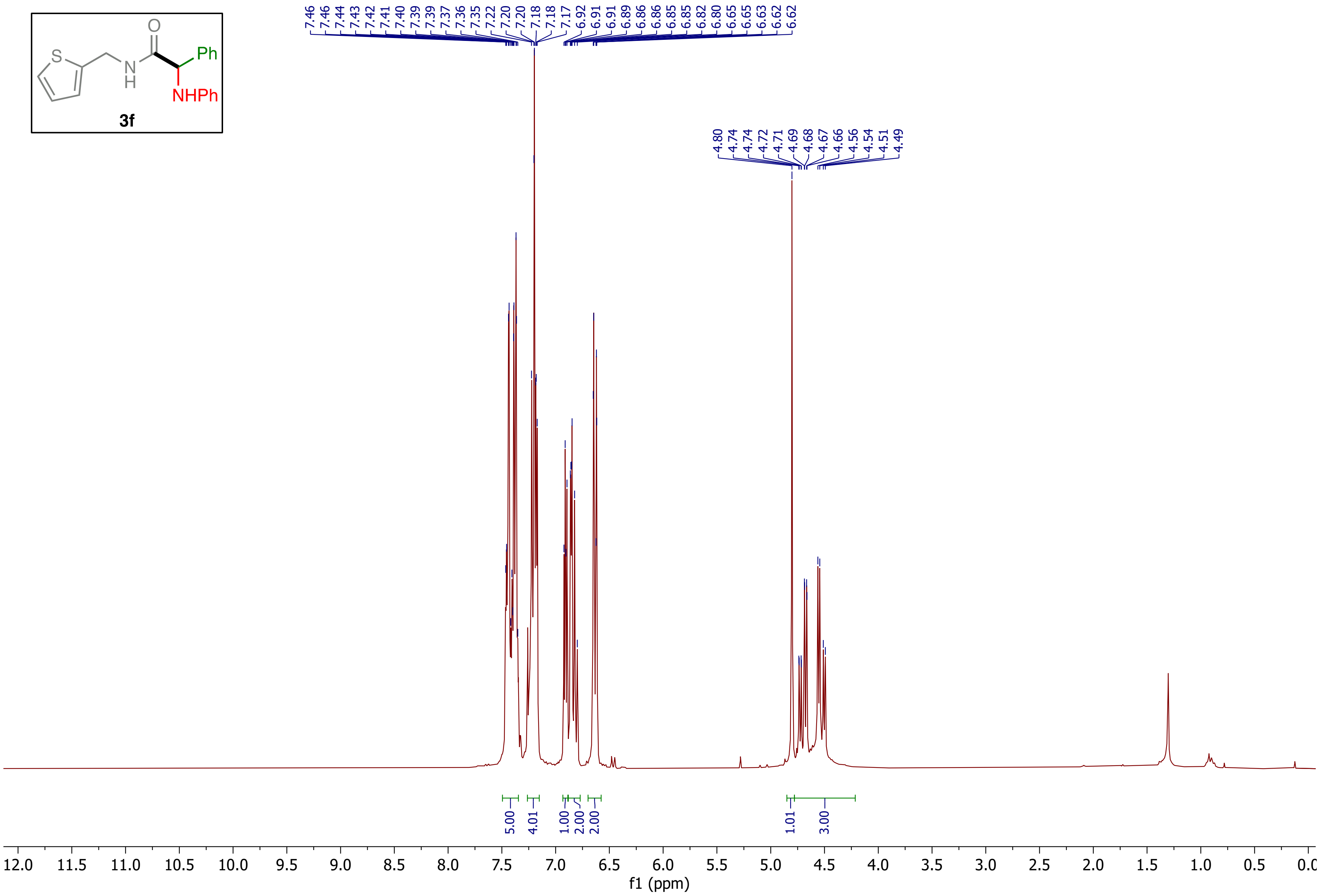

S
N
H
O
Ph
NHPh
3f
7.46 7.46 7.44 7.43 7.42 7.41 7.40 7.39 7.39 7.37 7.36 7.35 7.22 7.20 7.20 7.18 7.18 7.17 6.92 6.91 6.89 6.86 6.86 6.85 6.85 6.82 6.80 6.65 6.65 6.63 6.62 6.62
4.80 4.74 4.74 4.72 4.71 4.69 4.68 4.67 4.66 4.56 4.54 4.51 4.49
5.00 4.01 1.00 2.00 2.00 1.01 3.00
12.0 11.5 11.0 10.5 10.0 9.5 9.0 8.5 8.0 7.5 7.0 6.5 6.0 5.5 5.0 4.5 4.0 3.5 3.0 2.5 2.0 1.5 1.0 0.5 0.0
f1 (ppm)

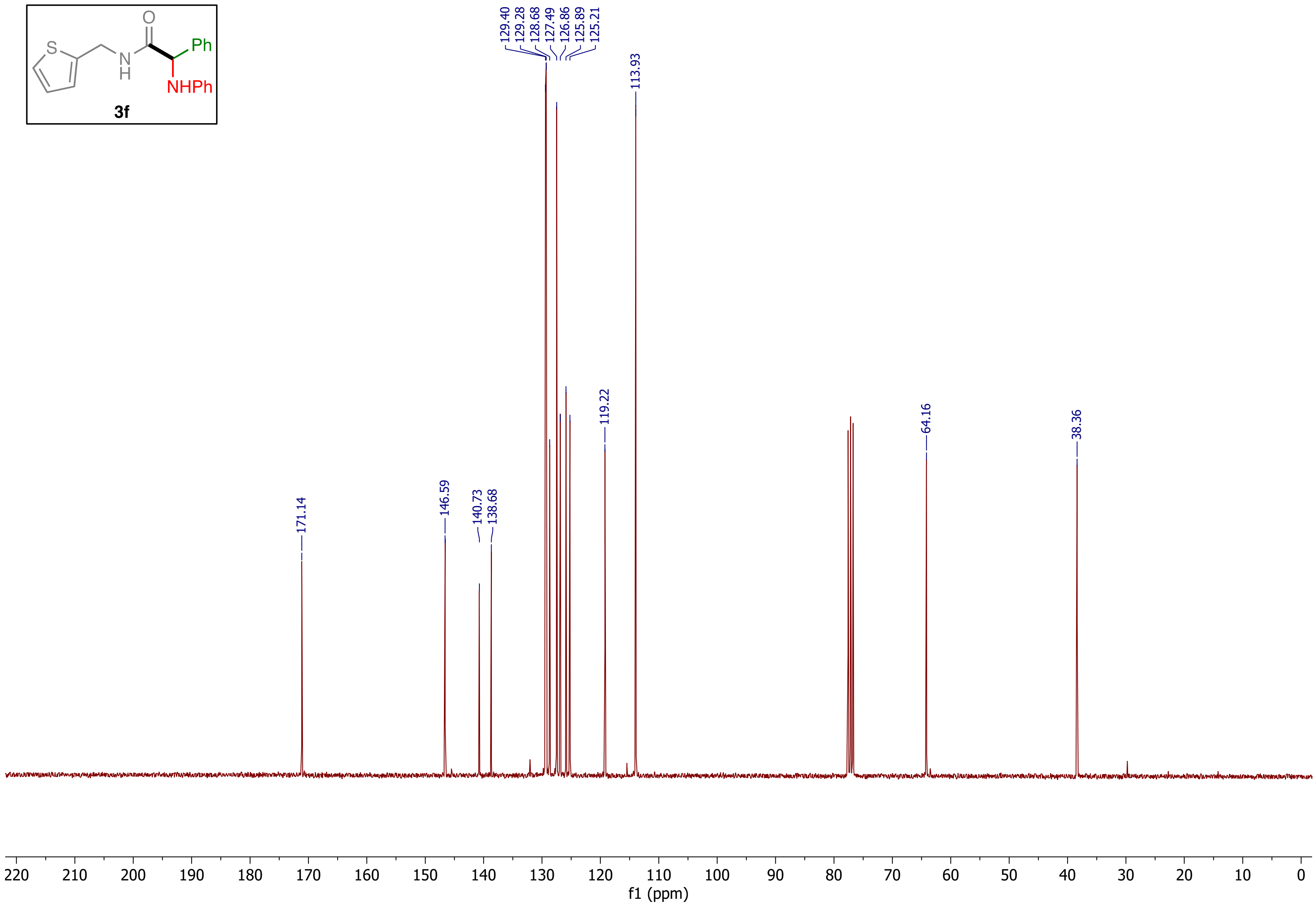
O
S
N
H
Ph
NHPh
3f
129.40
129.28
128.68
127.49
126.86
125.89
125.21
113.93
171.14
146.59
140.73
138.68
119.22
64.16
38.36
220 210 200 190 180 170 160 150 140 130 120 110 100 90 80 70 60 50 40 30 20 10 0
f1 (ppm)

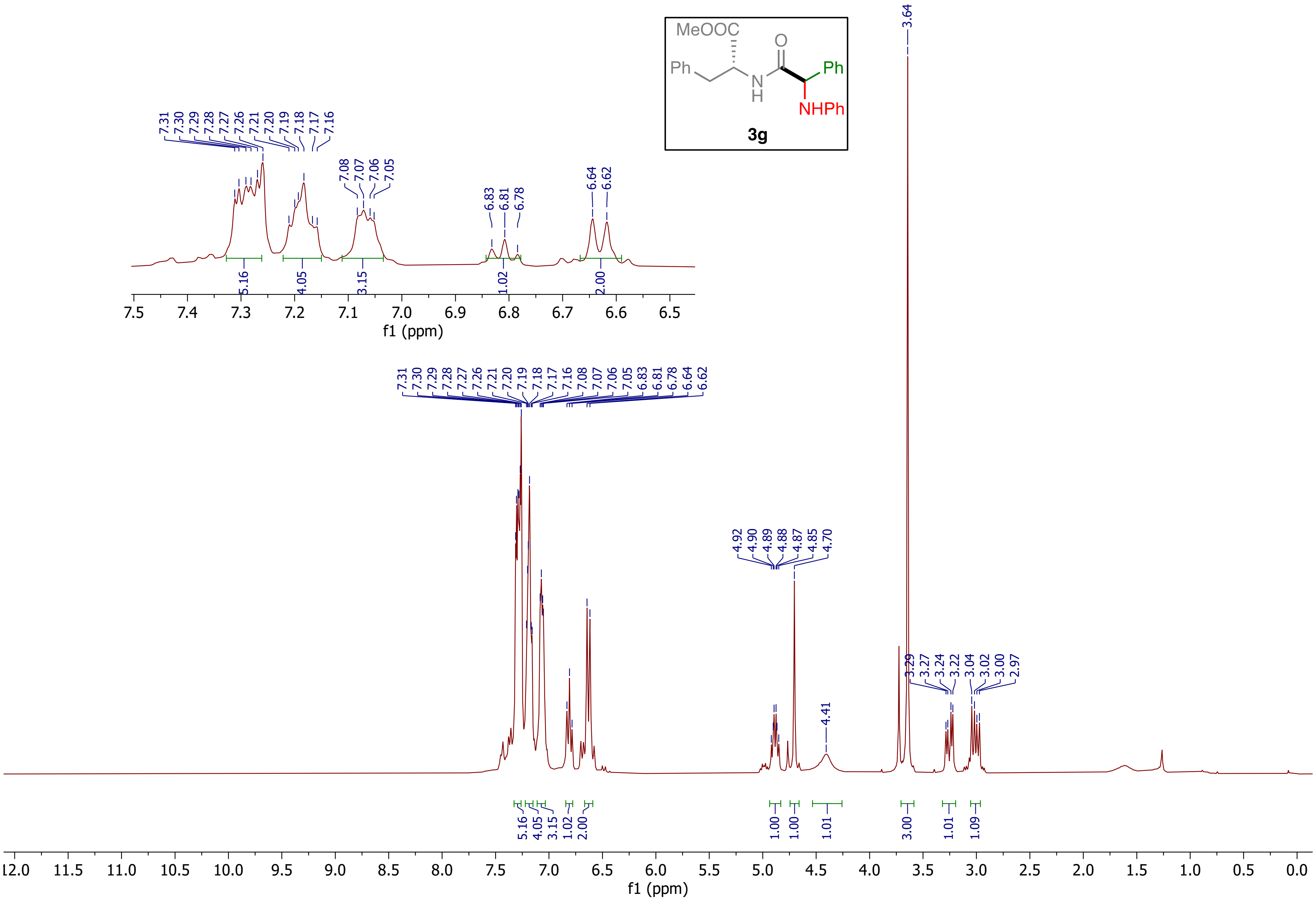
MeOOC
O
Ph
Ph
N
H
NHPh
3g
f1 (ppm)
f1 (ppm)

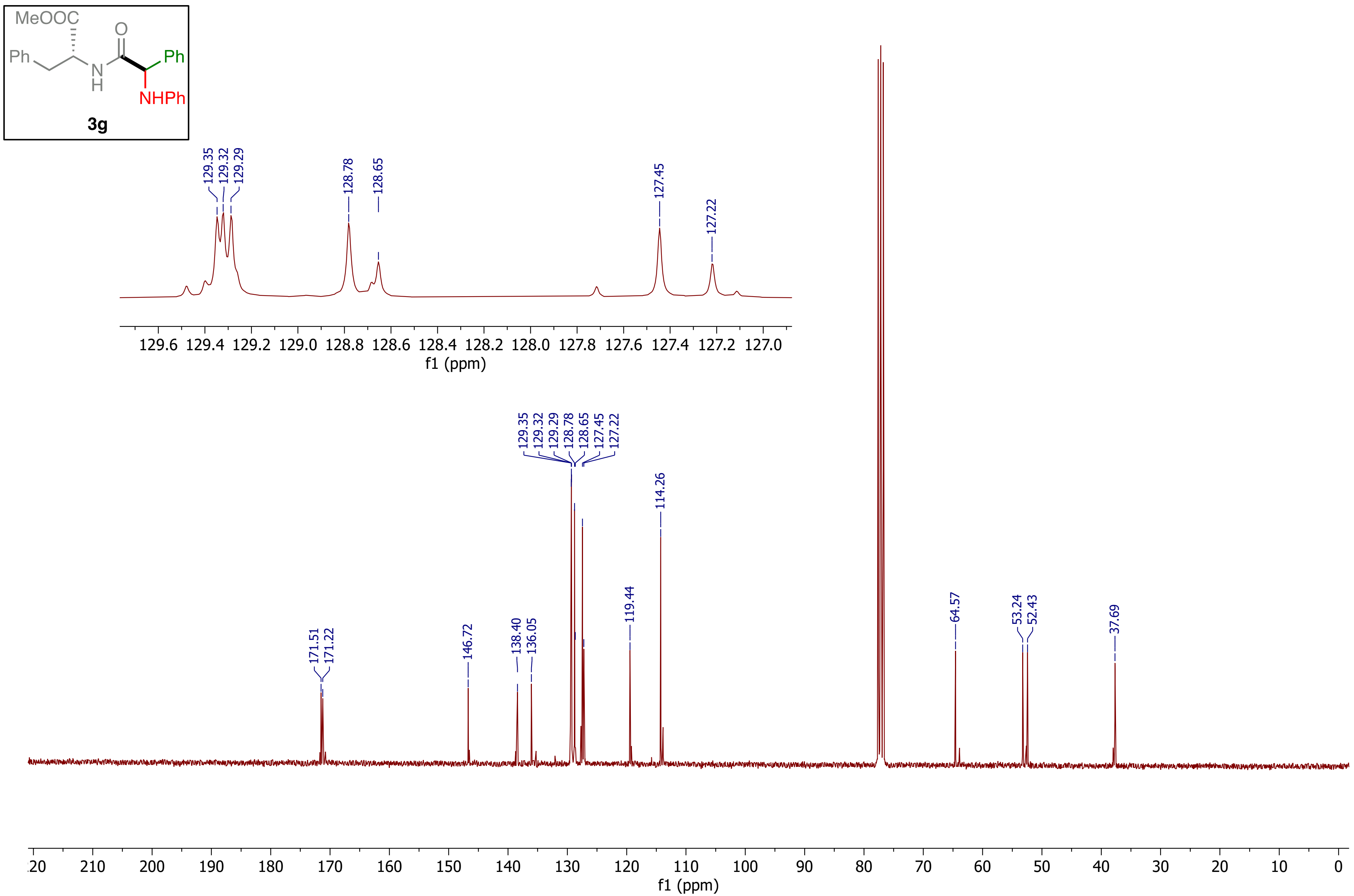
MeOOC
O
Ph
N
H
Ph
NHPh
3g
129.35
129.32
129.29
128.78
128.65
127.45
127.22
129.6 129.4 129.2 129.0 128.8 128.6 128.4 128.2 128.0 127.8 127.6 127.4 127.2 127.0
f1 (ppm)
171.51
171.22
146.72
138.40
136.05
119.44
114.26
64.57
53.24
52.43
37.69
220 210 200 190 180 170 160 150 140 130 120 110 100 90 80 70 60 50 40 30 20 10 0
f1 (ppm)

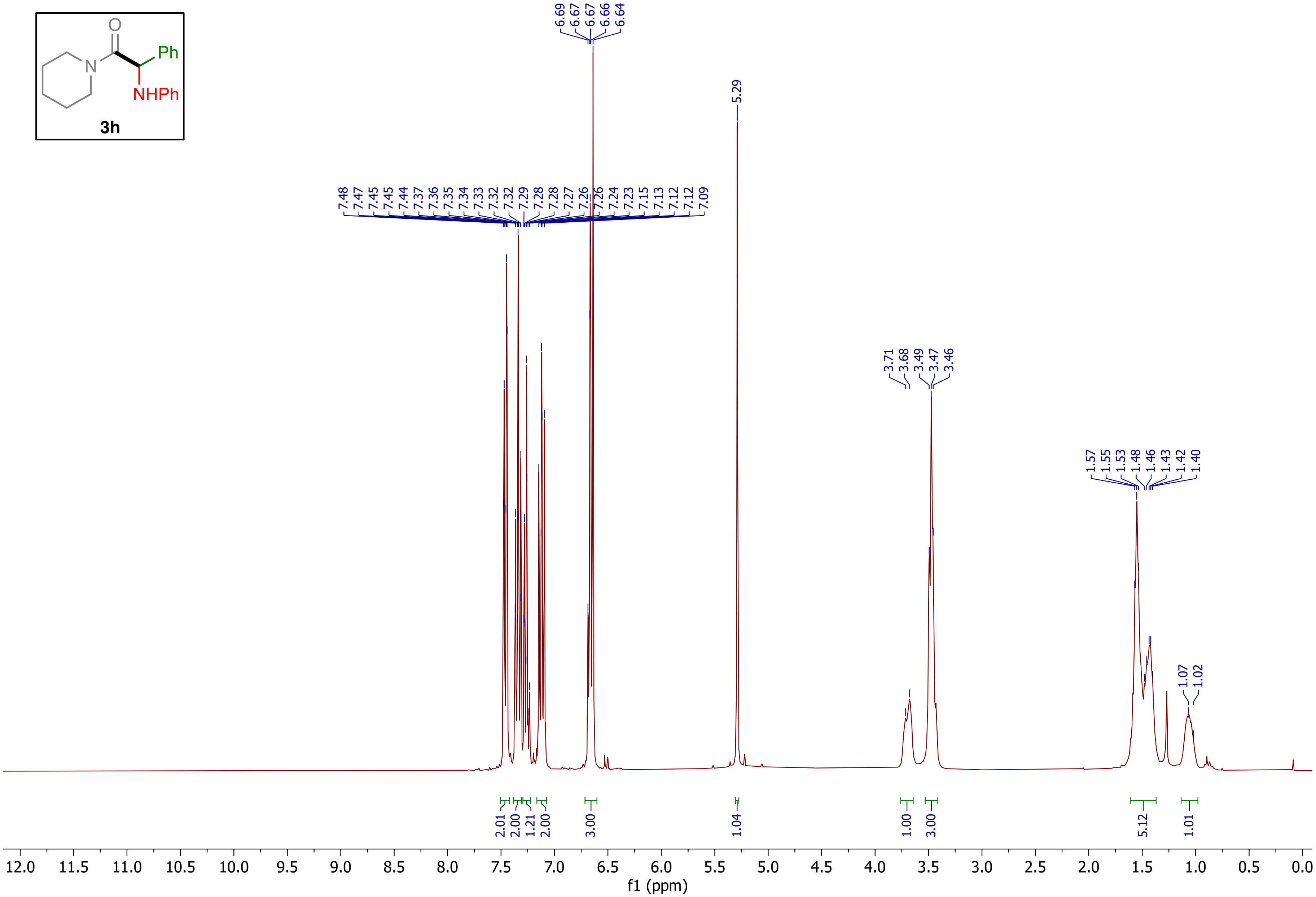

3h
NHPh
Ph
N
O
f1 (ppm)

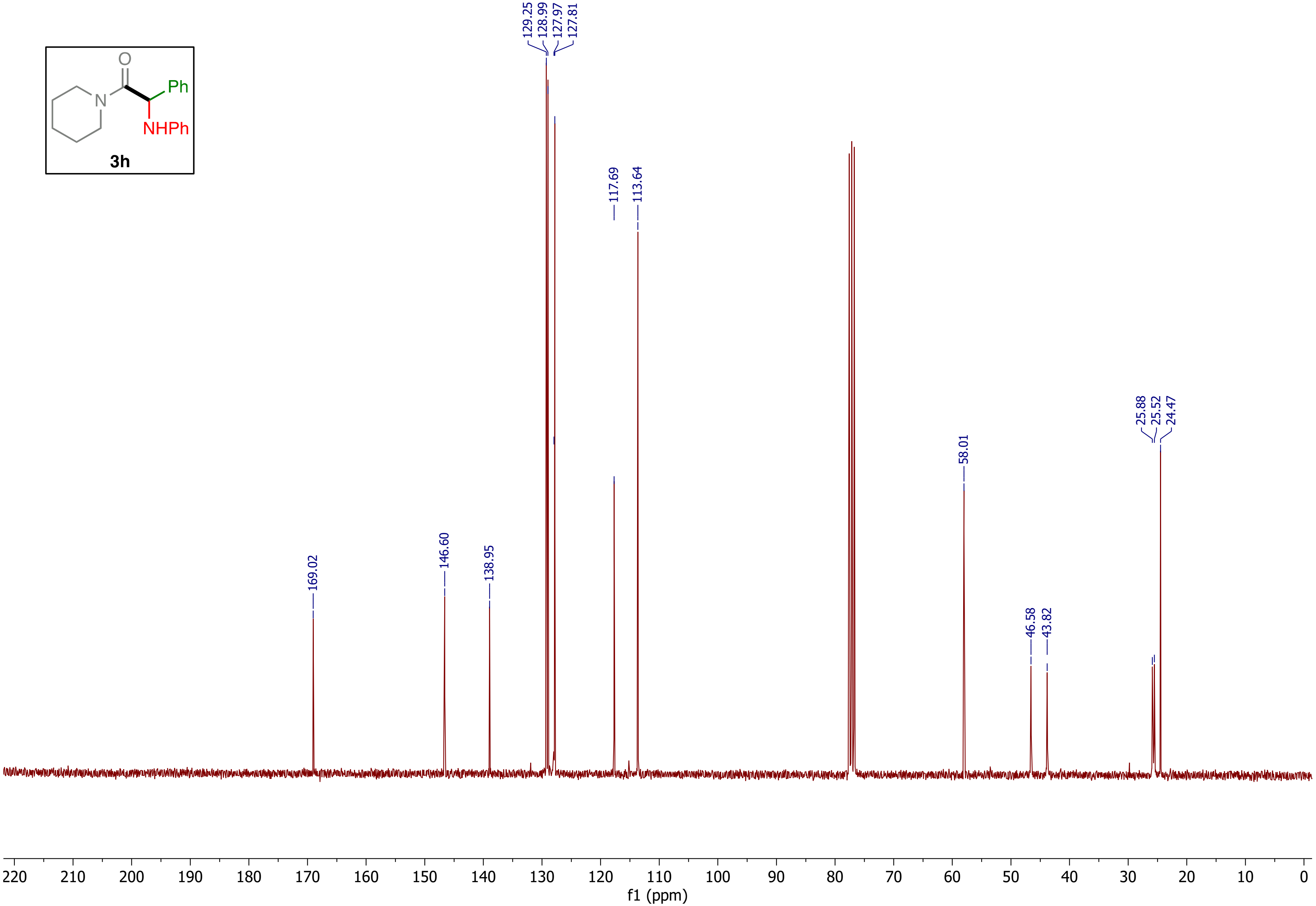

O
N
Ph
NHPh
3h
129.25
128.99
127.97
127.81
117.69
113.64
169.02
146.60
138.95
58.01
46.58
43.82
25.88
25.52
24.47
220 210 200 190 180 170 160 150 140 130 120 110 100 90 80 70 60 50 40 30 20 10 0
f1 (ppm)

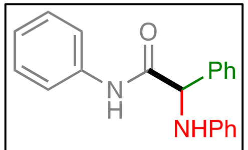
O
N
H
Ph
NHPh

3i

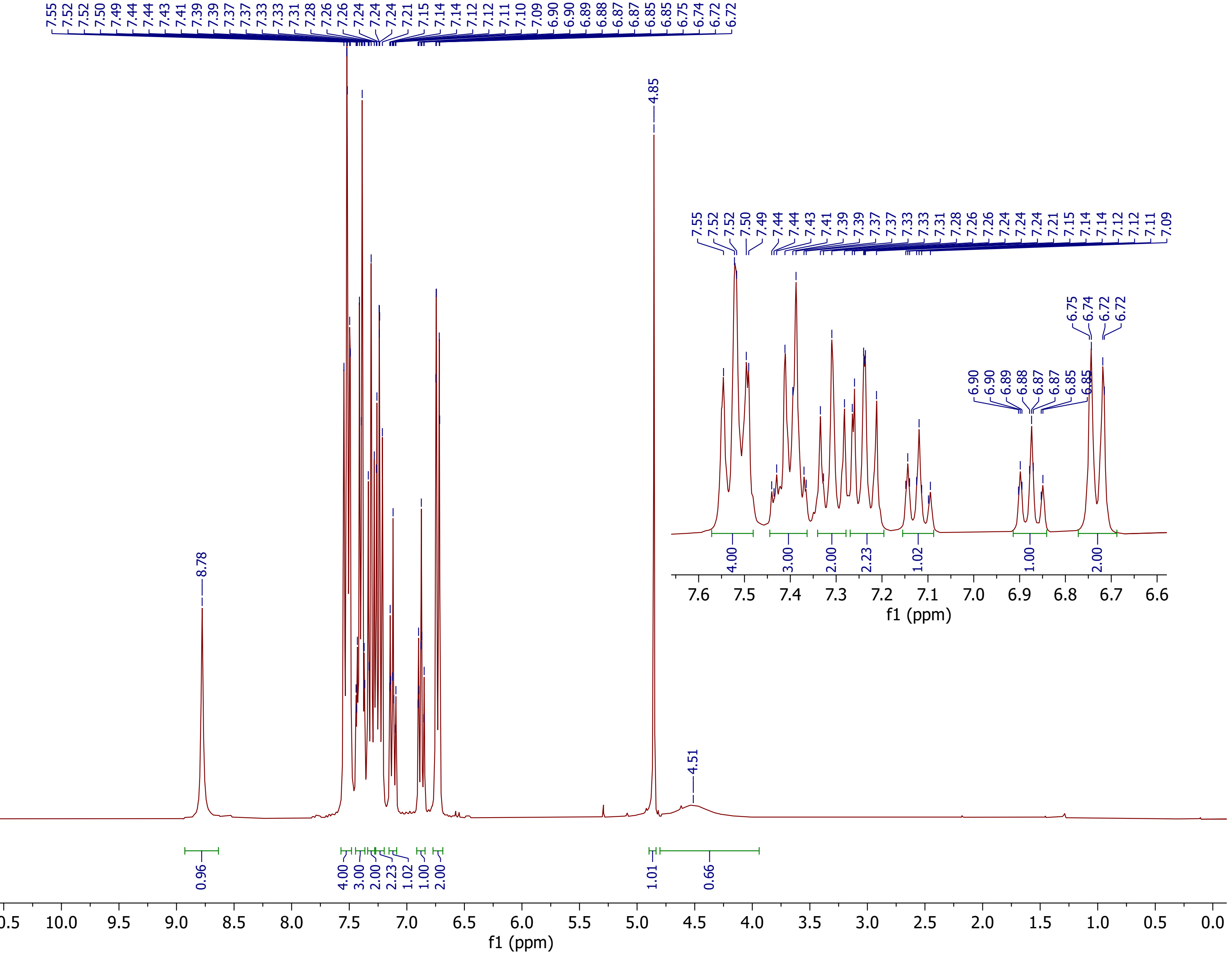
8.78
4.85
4.51
0.96
4.00
3.00
2.00
2.23
1.02
1.00
2.00
1.01
0.66
f1 (ppm)

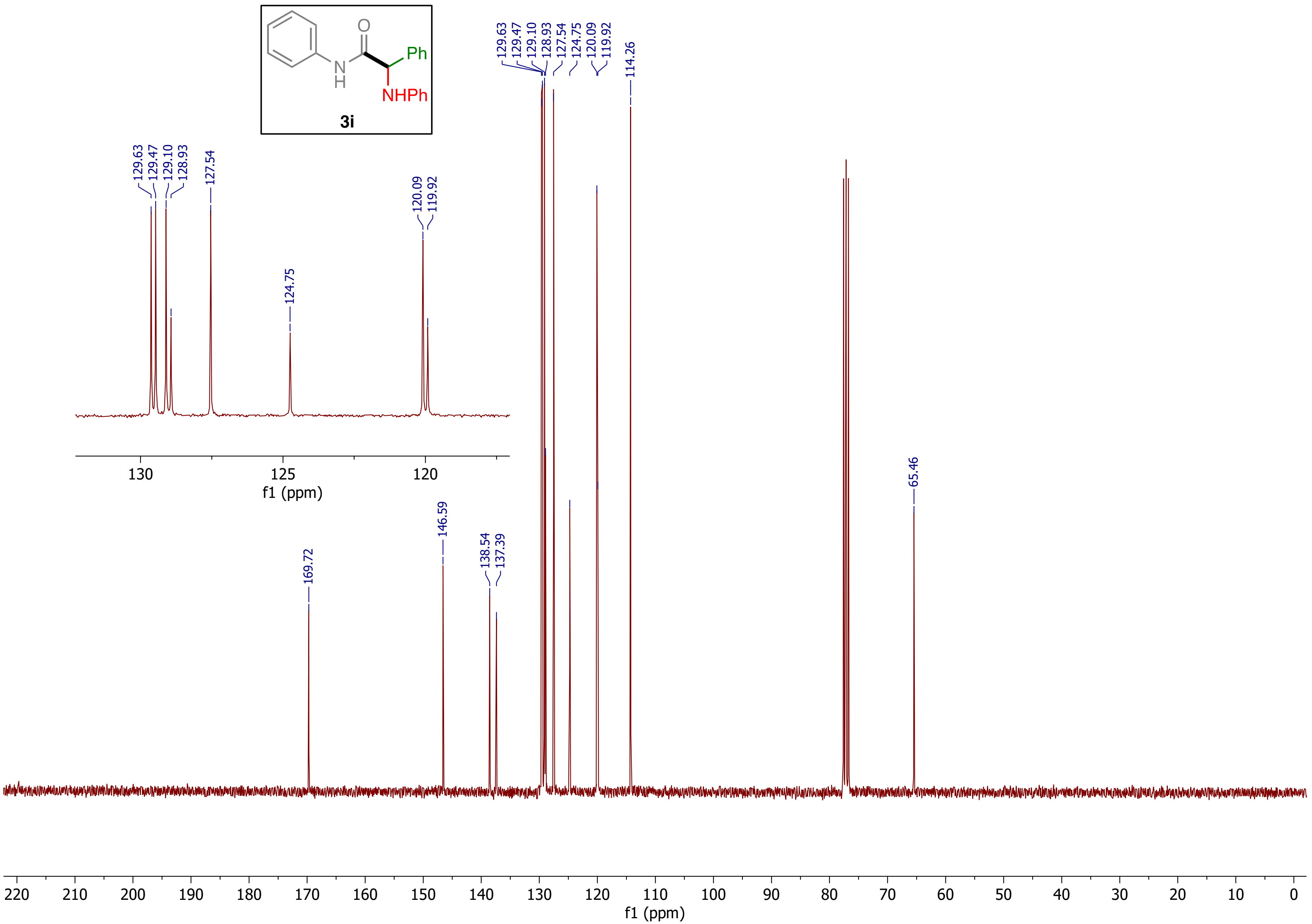

O
N
H
Ph
NHPh
3i
129.63
129.47
129.10
128.93
127.54
124.75
120.09
119.92
114.26
169.72
146.59
138.54
137.39
65.46
f1 (ppm)

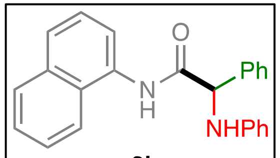
O
N
H
Ph
NHPh

3j

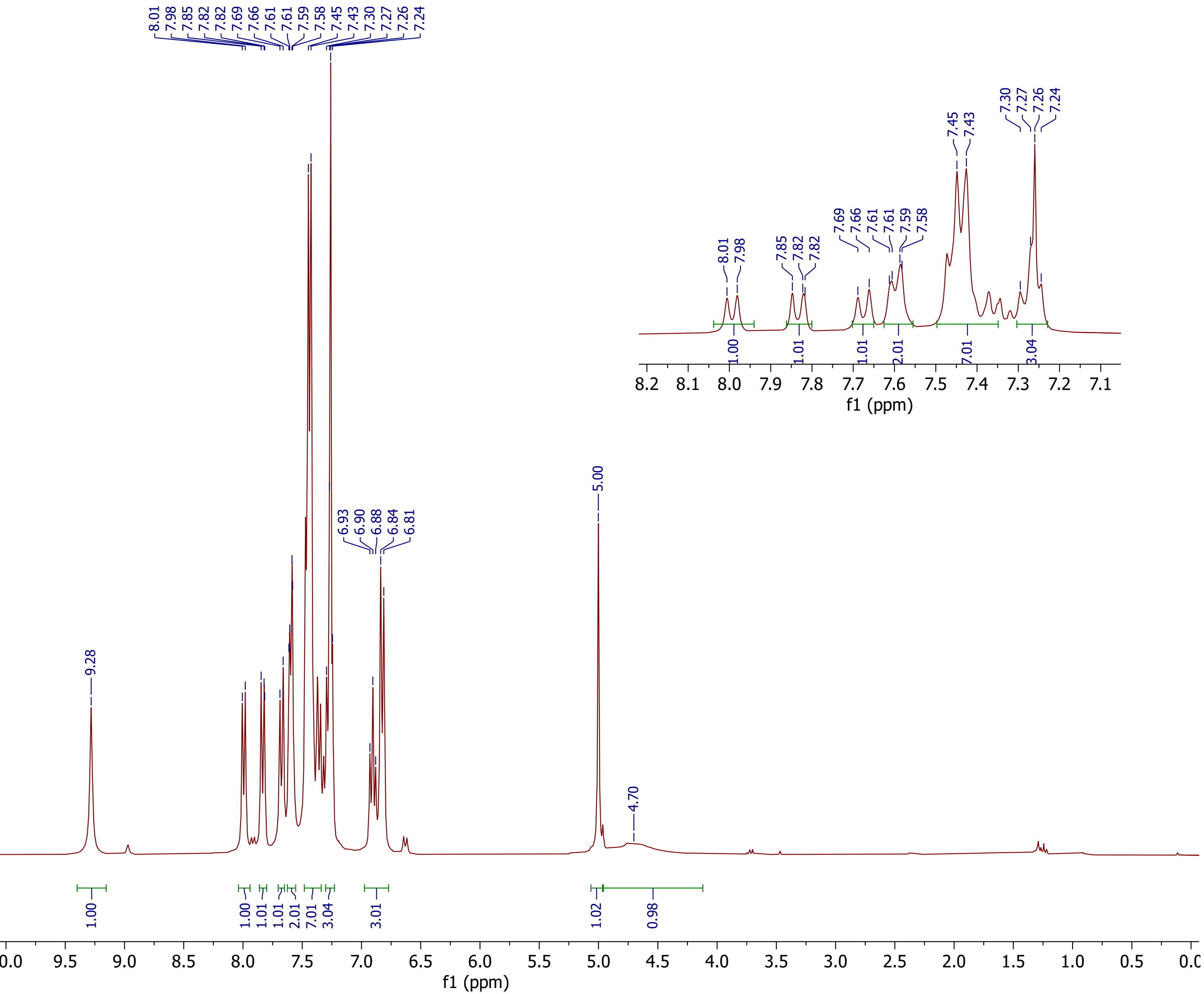
8.01
7.98
7.85
7.82
7.82
7.69
7.66
7.61
7.61
7.59
7.58
7.45
7.43
7.30
7.27
7.26
7.24
9.28
6.93
6.90
6.88
6.84
6.81
5.00
4.70
1.00
1.01
1.01
2.01
7.01
3.04
3.01
1.02
0.98
f1 (ppm)

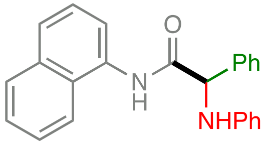
O
N
H
Ph
NHPh

3j

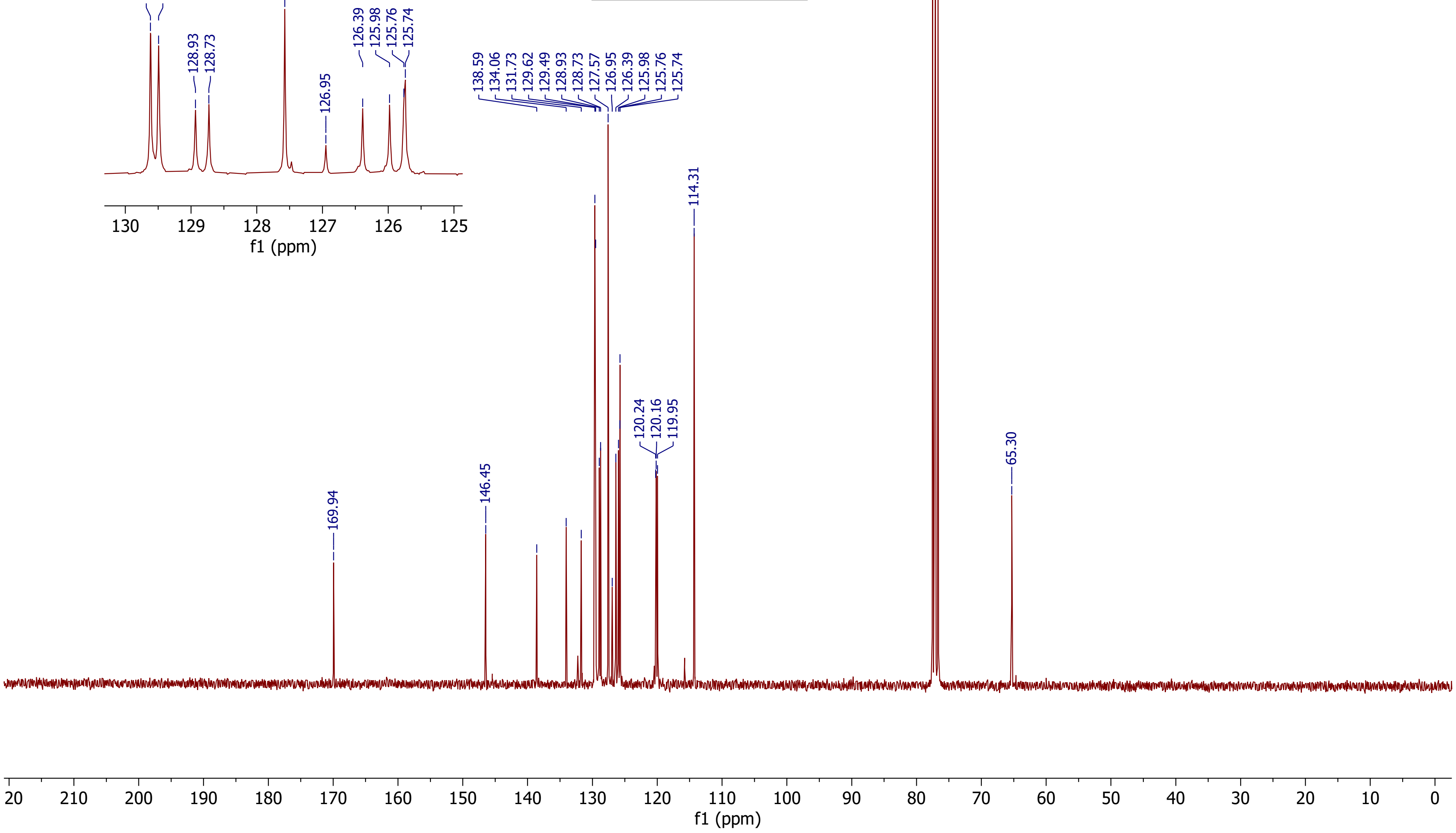
129.62
129.49
128.93
128.73
127.57
126.95
126.39
125.98
125.76
125.74
130
129
128
127
126
125
f1 (ppm)
138.59
134.06
131.73
129.62
129.49
128.93
128.73
127.57
126.95
126.39
125.98
125.76
125.74
114.31
120.24
120.16
119.95
169.94
146.45
65.30
220
210
200
190
180
170
160
150
140
130
120
110
100
90
80
70
60
50
40
30
20
10
0
f1 (ppm)

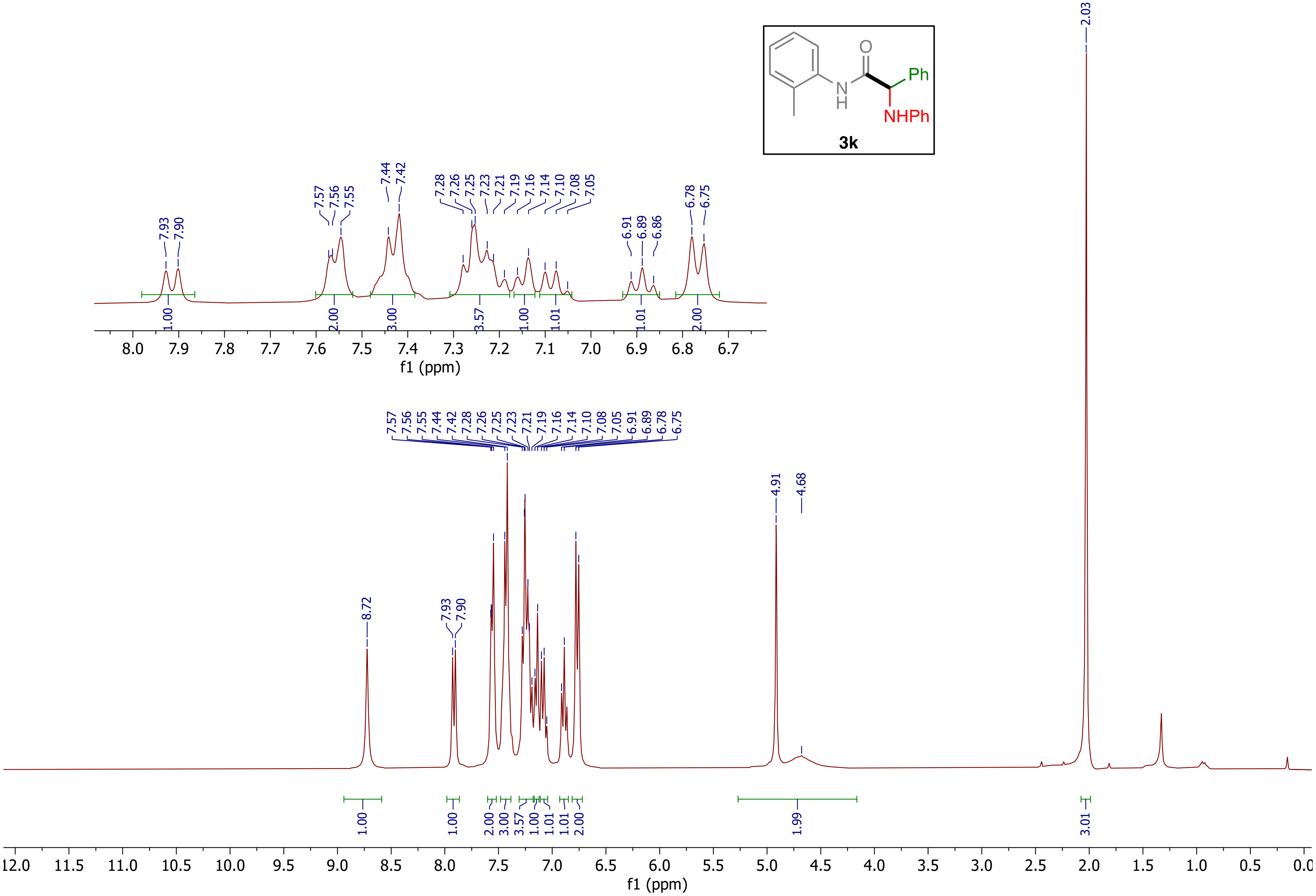
O
N
H
Ph
NHPh
3k
f1 (ppm)
f1 (ppm)

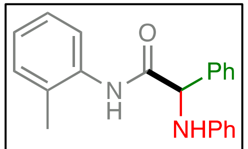
O
N
H
Ph
NHPh

3k

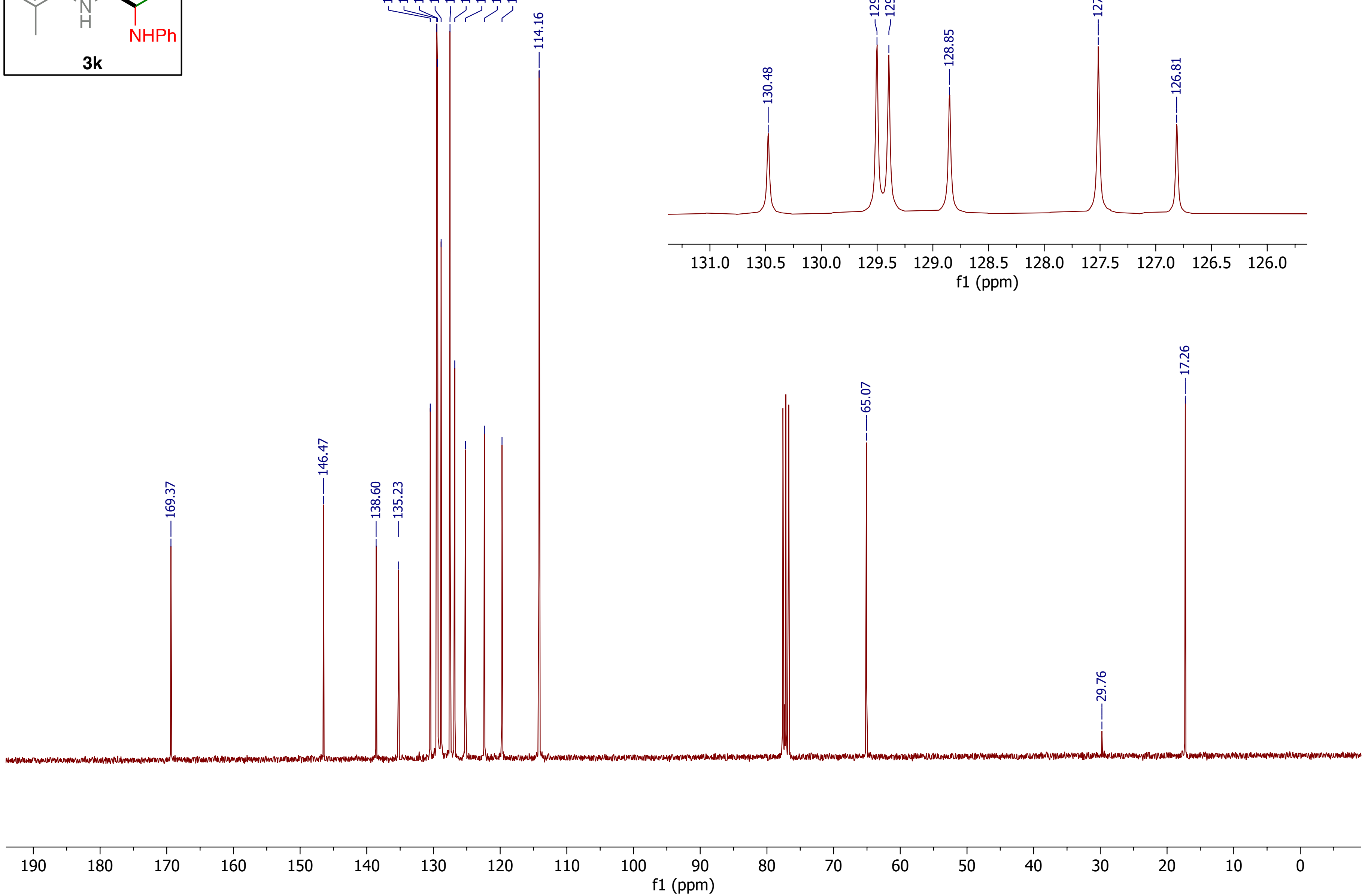
130.48
129.50
129.39
128.85
127.52
126.81
125.19
122.37
119.72
114.16
169.37
146.47
138.60
135.23
65.07
29.76
17.26
190 180 170 160 150 140 130 120 110 100 90 80 70 60 50 40 30 20 10 0
f1 (ppm)
131.0 130.5 130.0 129.5 129.0 128.5 128.0 127.5 127.0 126.5 126.0
f1 (ppm)

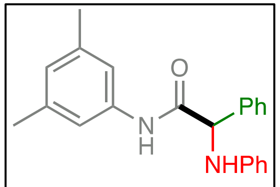

3l

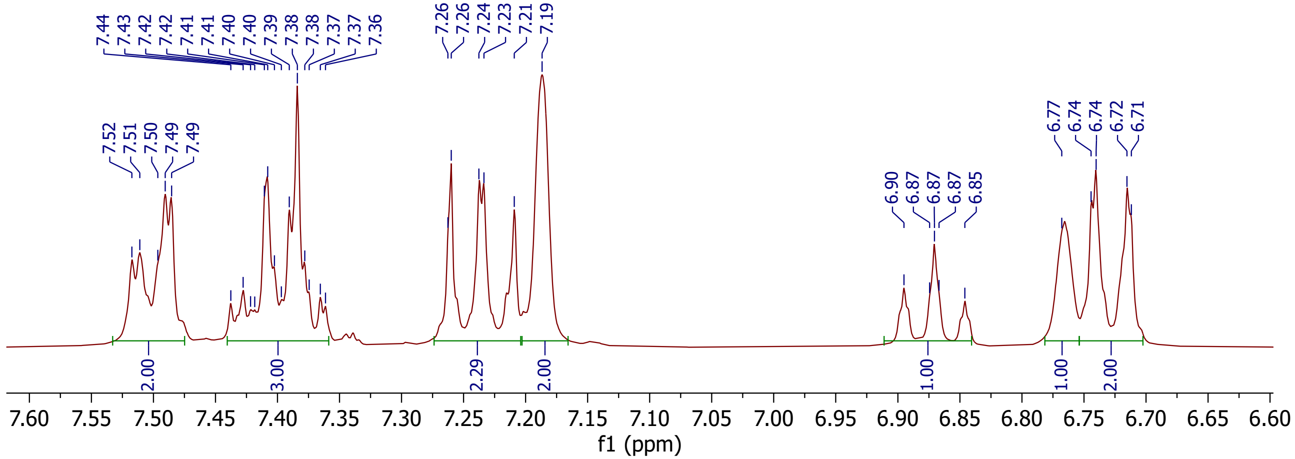

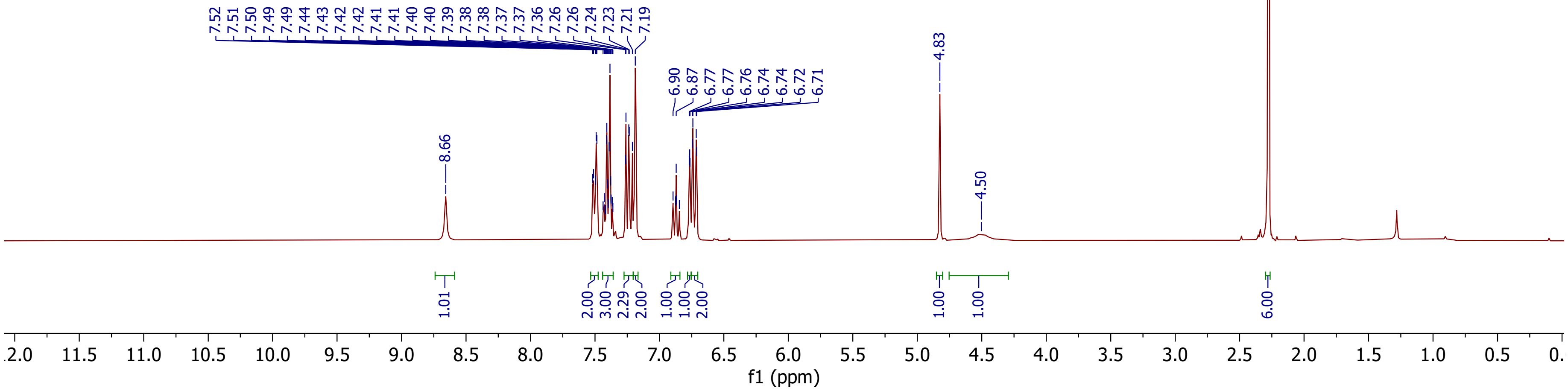

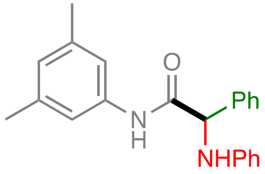
O
N
H
Ph
NHPh

3l

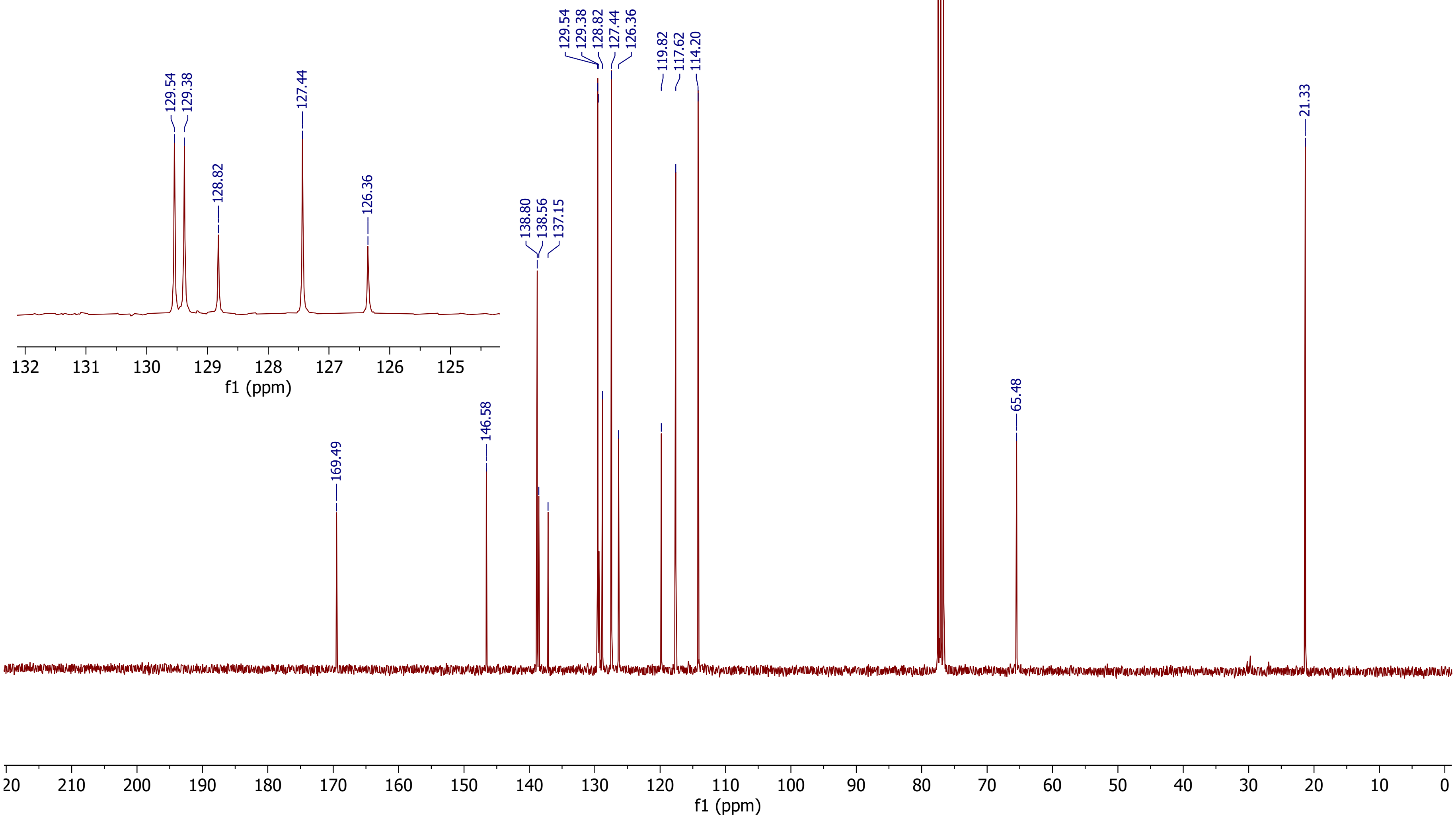
129.54
129.38
128.82
127.44
126.36
119.82
117.62
114.20
21.33
138.80
138.56
137.15
146.58
169.49
65.48
f1 (ppm)
f1 (ppm)

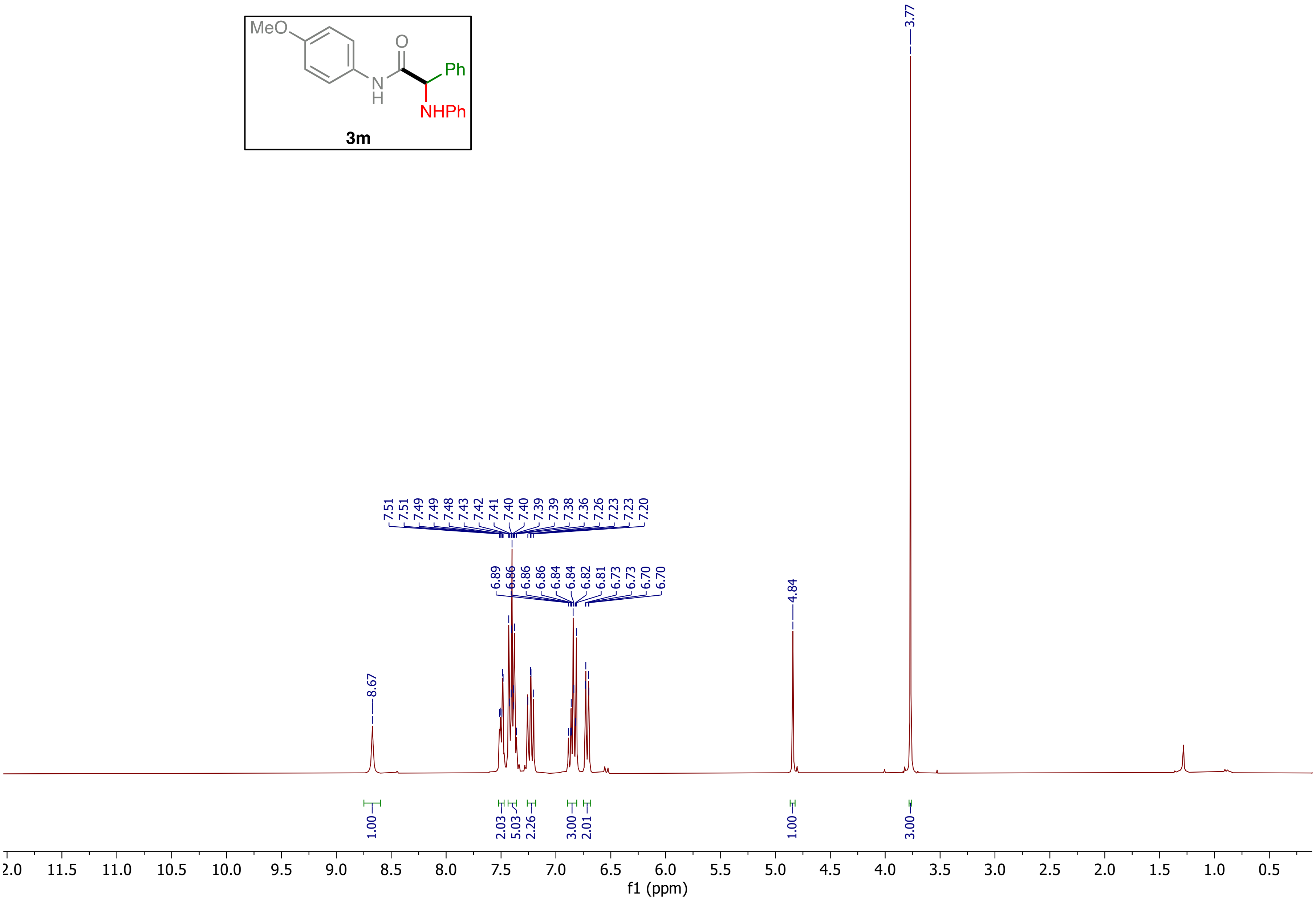
MeO
O
N
H
Ph
NHPh
3m
7.51 7.51 7.49 7.49 7.48 7.43 7.42 7.41 7.40 7.40 7.39 7.39 7.38 7.36 7.26 7.23 7.23 7.20
6.89 6.86 6.86 6.86 6.84 6.84 6.82 6.81 6.73 6.73 6.70 6.70
8.67
4.84
3.77
1.00
2.03
5.03
2.26
3.00
2.01
1.00
3.00
2.0 11.5 11.0 10.5 10.0 9.5 9.0 8.5 8.0 7.5 7.0 6.5 6.0 5.5 5.0 4.5 4.0 3.5 3.0 2.5 2.0 1.5 1.0 0.5
f1 (ppm)

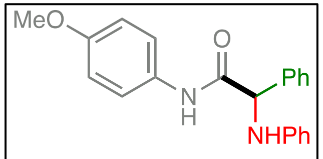
MeO
O
Ph
N
H
NHPh

3m

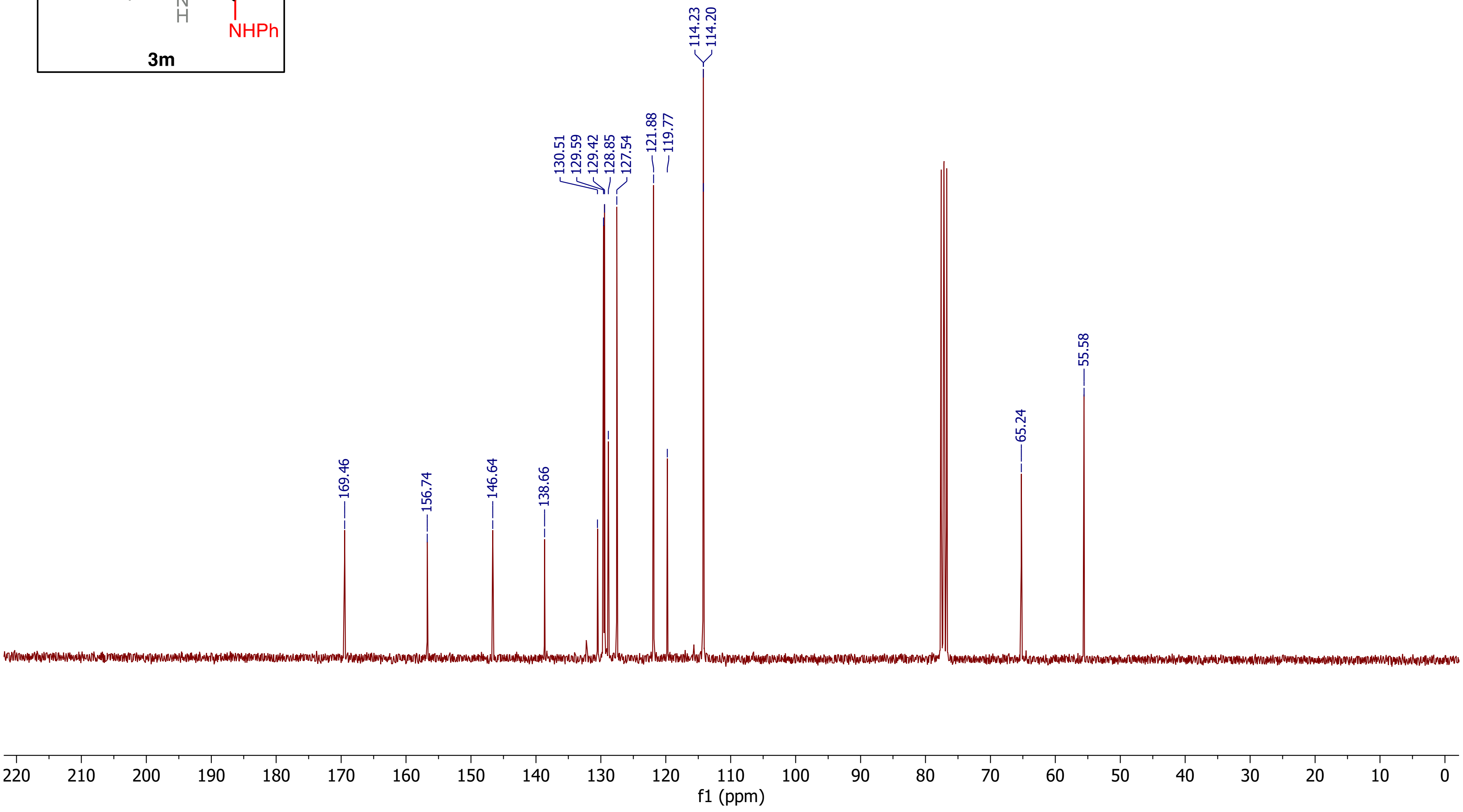
169.46
156.74
146.64
138.66
130.51
129.59
129.42
128.85
127.54
121.88
119.77
114.23
114.20
65.24
55.58
220
210
200
190
180
170
160
150
140
130
120
110
100
90
80
70
60
50
40
30
20
10
0
f1 (ppm)

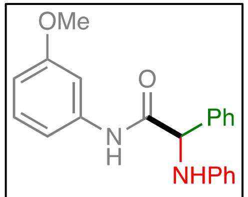

3n

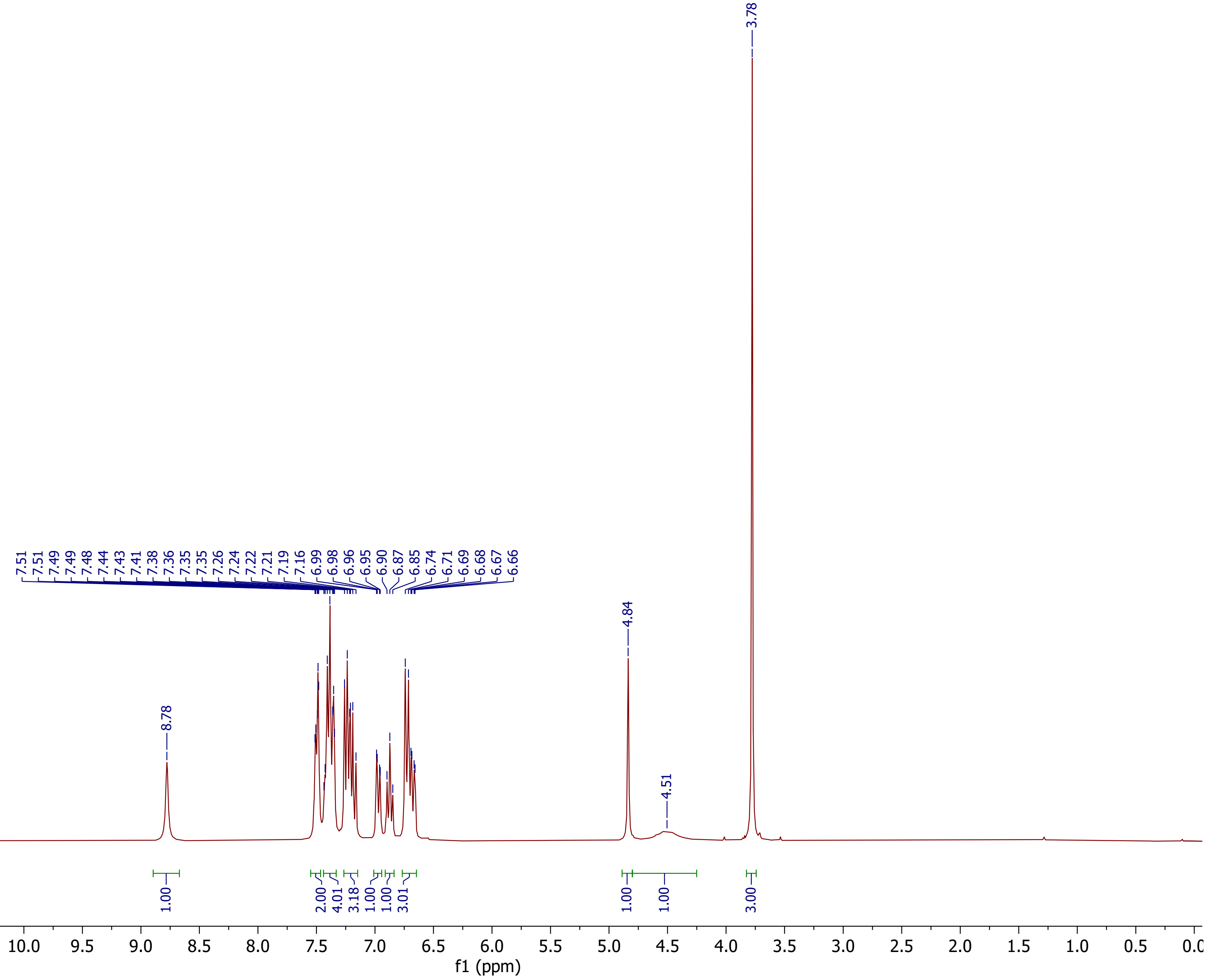

7.51
7.51
7.49
7.49
7.48
7.44
7.43
7.41
7.38
7.36
7.35
7.35
7.26
7.24
7.22
7.21
7.19
7.16
6.99
6.98
6.96
6.95
6.90
6.87
6.85
6.74
6.71
6.69
6.68
6.67
6.66
8.78
4.84
4.51
3.78
1.00
2.00
4.01
3.18
1.00
1.00
3.01
1.00
1.00
3.00
12.0 11.5 11.0 10.5 10.0 9.5 9.0 8.5 8.0 7.5 7.0 6.5 6.0 5.5 5.0 4.5 4.0 3.5 3.0 2.5 2.0 1.5 1.0 0.5 0.0
f1 (ppm)

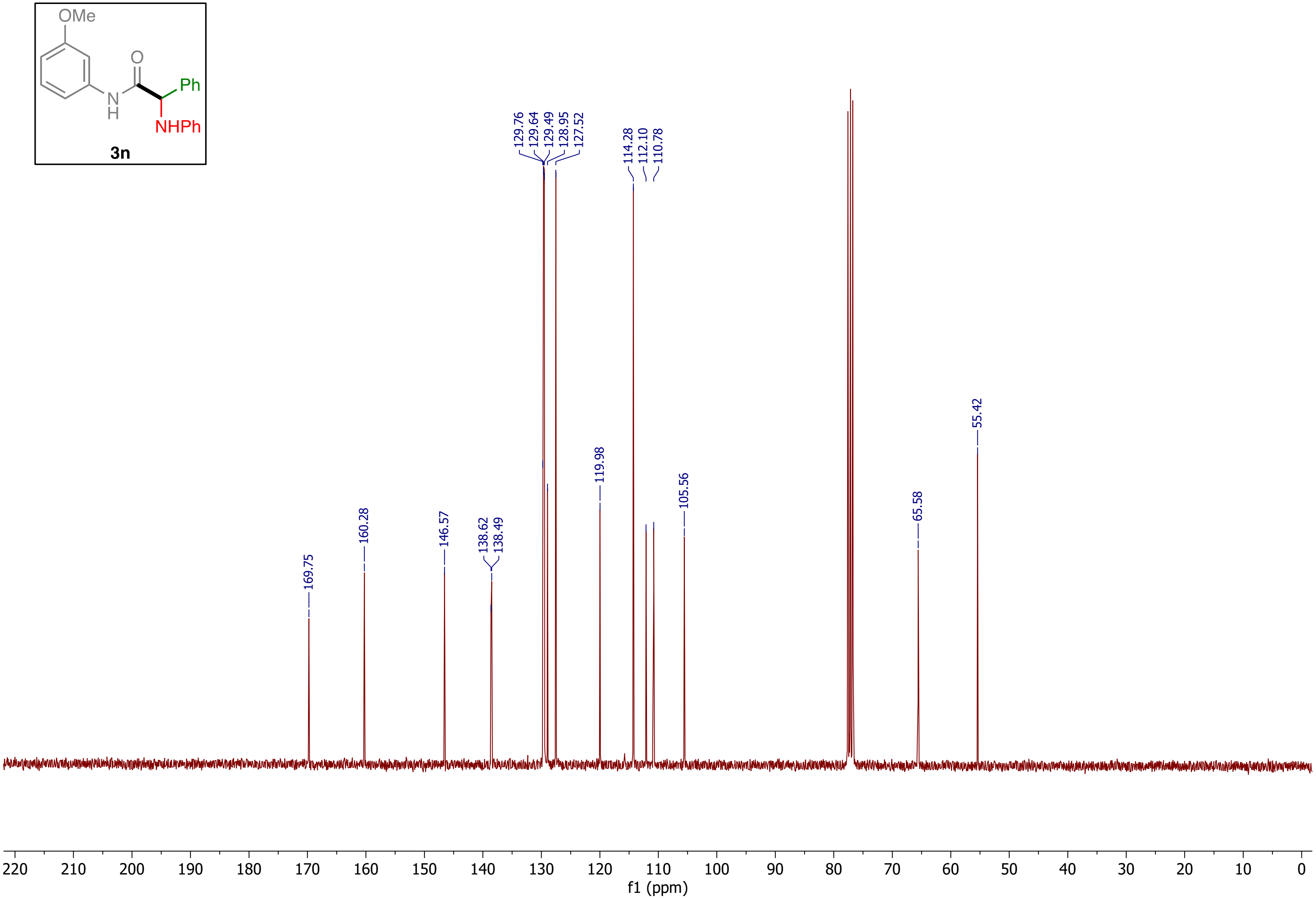
OMe
O
N
H
Ph
NHPh
3n
129.76
129.64
129.49
128.95
127.52
114.28
112.10
110.78
169.75
160.28
146.57
138.62
138.49
119.98
105.56
65.58
55.42
220 210 200 190 180 170 160 150 140 130 120 110 100 90 80 70 60 50 40 30 20 10 0
f1 (ppm)

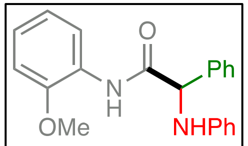

O
N
H
Ph
OMe
NHPh

3o

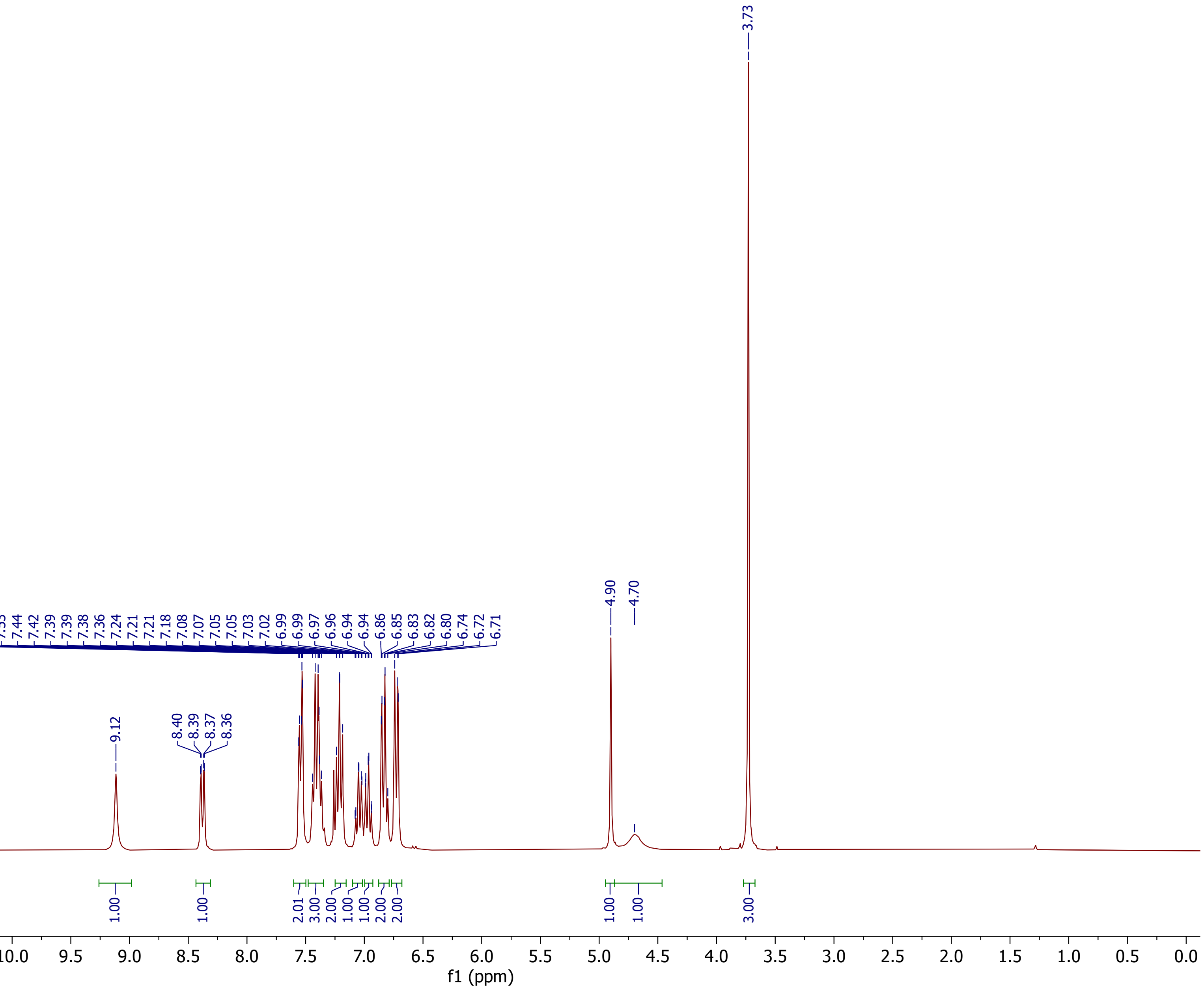

3.73
4.90
4.70
9.12
8.40
8.39
8.37
8.36
7.56
7.55
7.54
7.53
7.53
7.44
7.42
7.39
7.39
7.38
7.36
7.24
7.21
7.21
7.18
7.08
7.07
7.05
7.05
7.03
7.02
6.99
6.99
6.97
6.96
6.94
6.94
6.86
6.85
6.83
6.82
6.80
6.74
6.72
6.71
1.00
1.00
2.01
3.00
2.00
1.00
1.00
2.00
2.00
1.00
1.00
3.00
12.0
11.5
11.0
10.5
10.0
9.5
9.0
8.5
8.0
7.5
7.0
6.5
6.0
5.5
5.0
4.5
4.0
3.5
3.0
2.5
2.0
1.5
1.0
0.5
0.0
f1 (ppm)

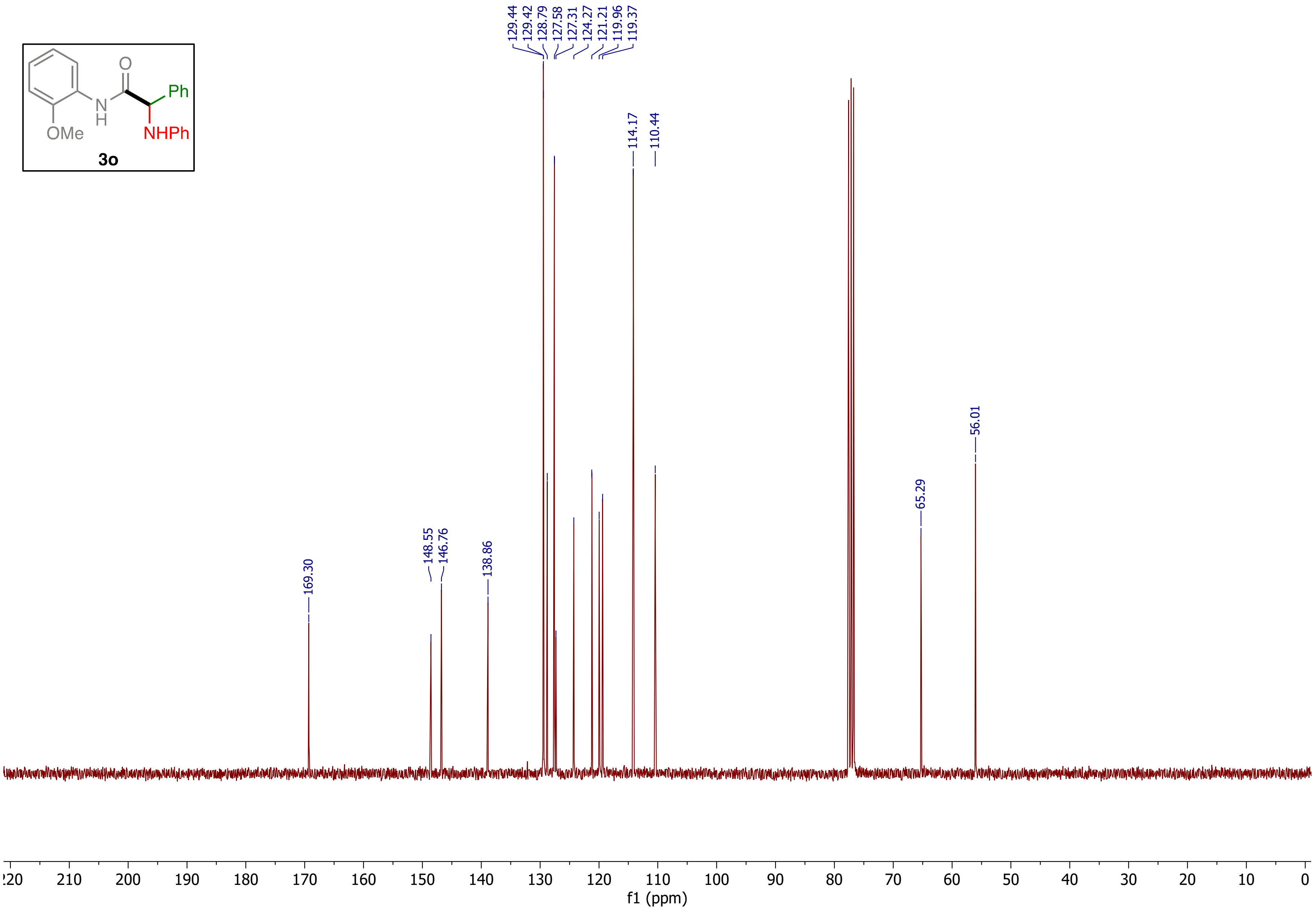

O
N
H
Ph
NHPh
OMe
3o
129.44
129.42
128.79
127.58
127.31
124.27
121.21
119.96
119.37
114.17
110.44
169.30
148.55
146.76
138.86
65.29
56.01
220
210
200
190
180
170
160
150
140
130
120
110
100
90
80
70
60
50
40
30
20
10
0
f1 (ppm)

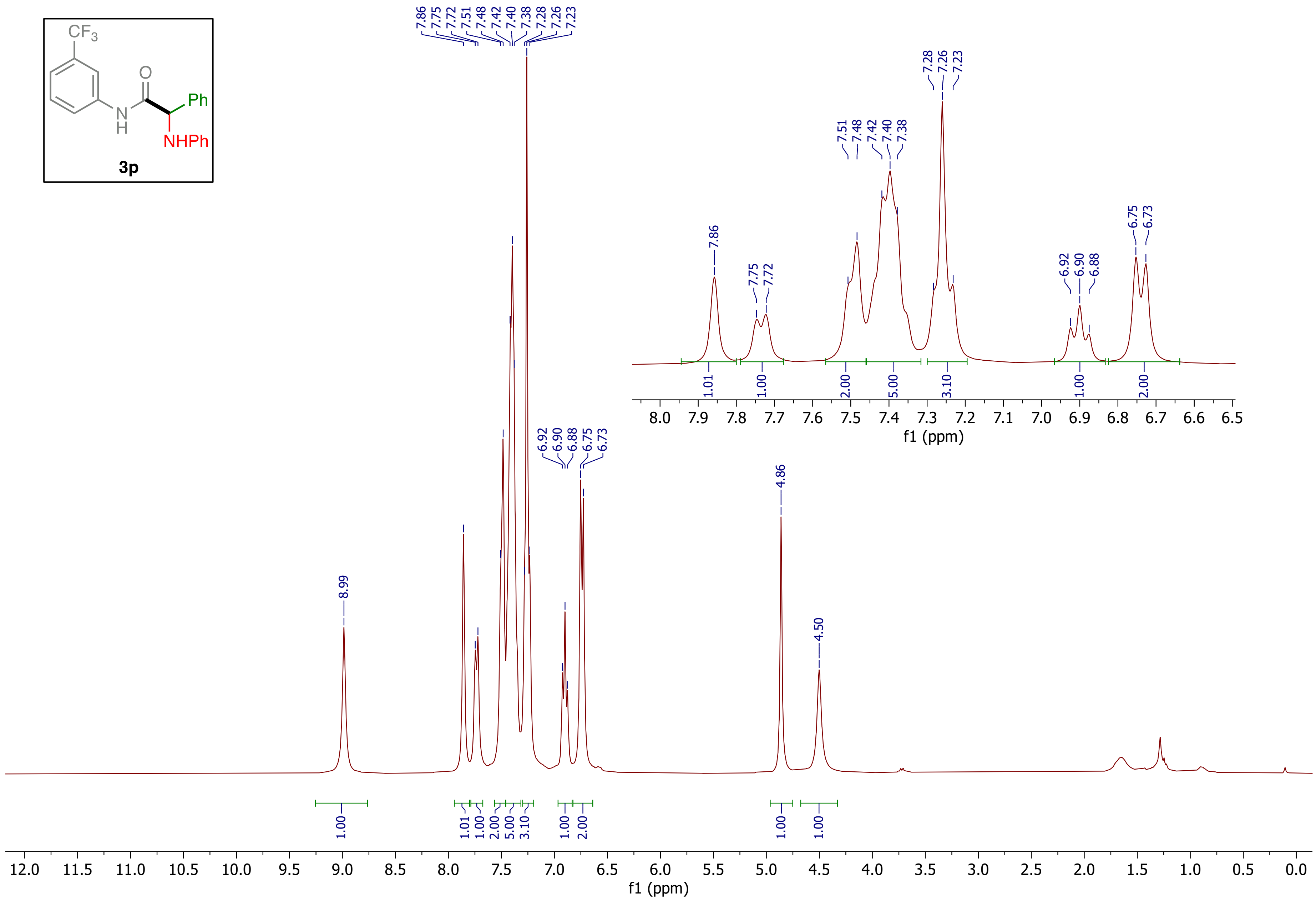

CF3
O
Ph
N
H
NHPh
3p
7.86
7.75
7.72
7.51
7.48
7.42
7.40
7.38
7.28
7.26
7.23
6.92
6.90
6.88
6.75
6.73
8.99
4.86
4.50
1.01
1.00
2.00
5.00
3.10
f1 (ppm)

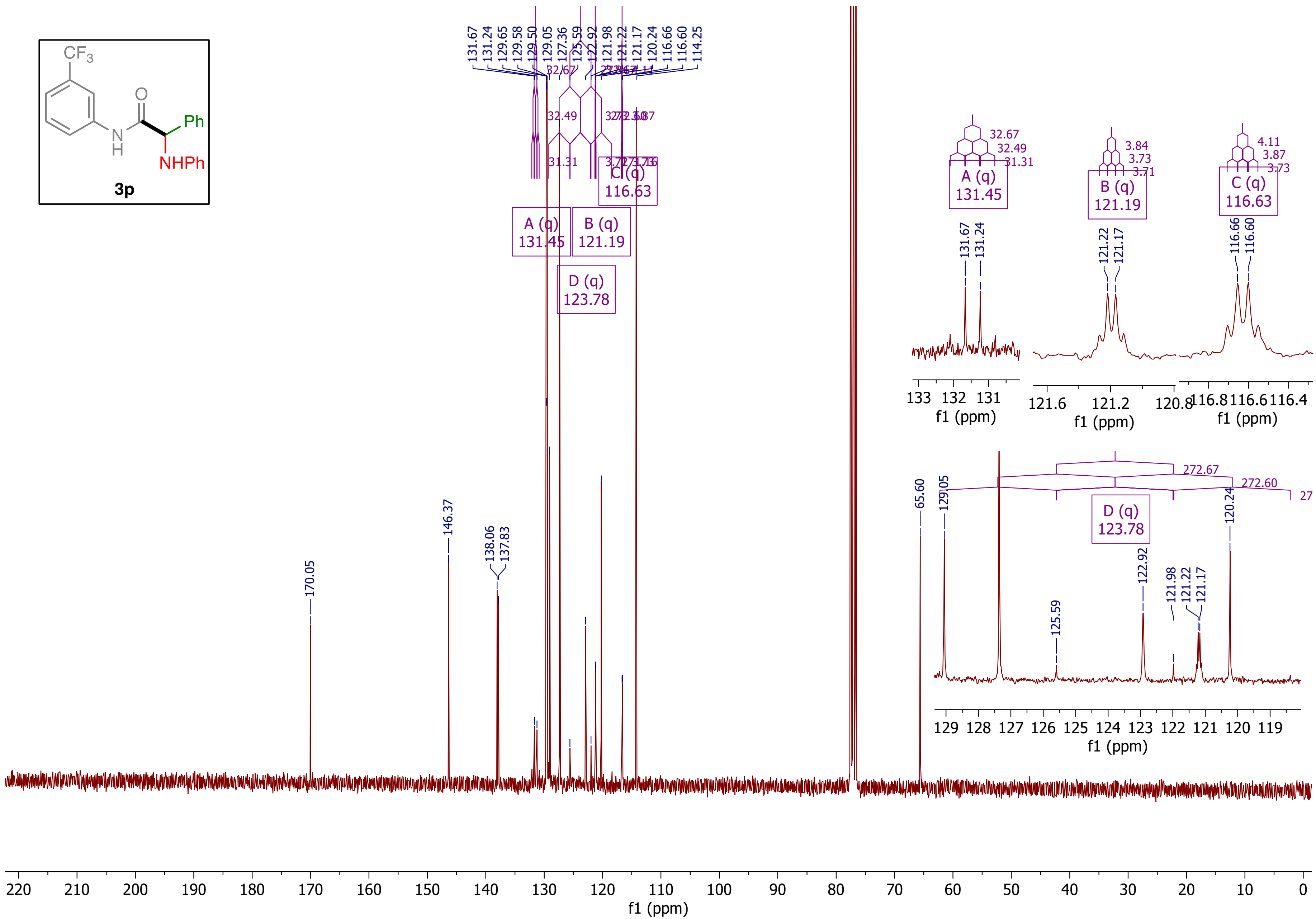

3p
A (q) 131.45
B (q) 121.19
C (q) 116.63
D (q) 123.78
170.05
146.37
138.06
137.83
65.60
f1 (ppm)

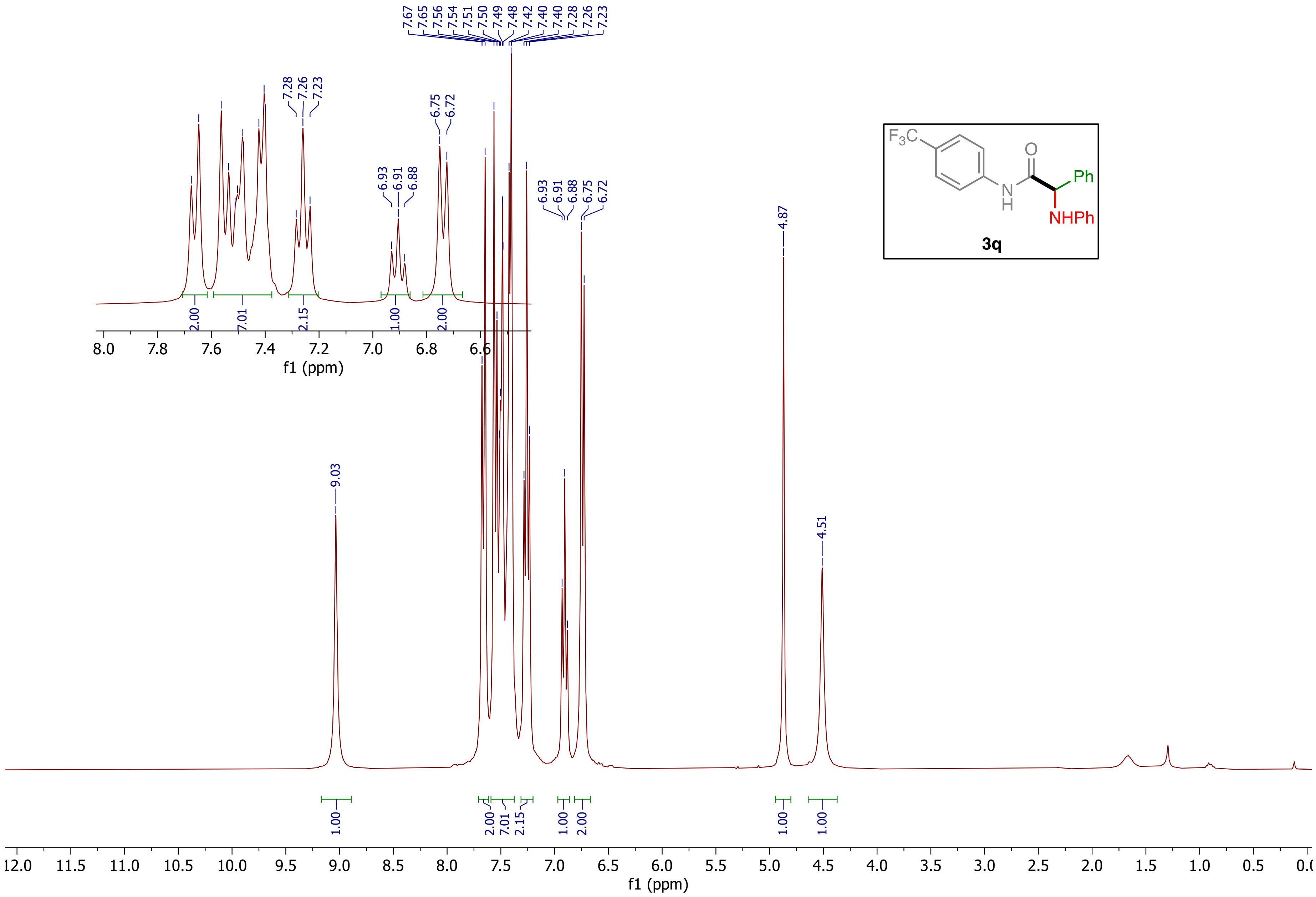

7.67 7.65 7.56 7.54 7.51 7.50 7.49 7.48 7.42 7.40 7.40 7.28 7.26 7.23
6.93 6.91 6.88 6.75 6.72
9.03
4.87
4.51
1.00 2.00 7.01 2.15 1.00 2.00 1.00 1.00
f1 (ppm)
F3C
O
N
H
Ph
NHPh
3q

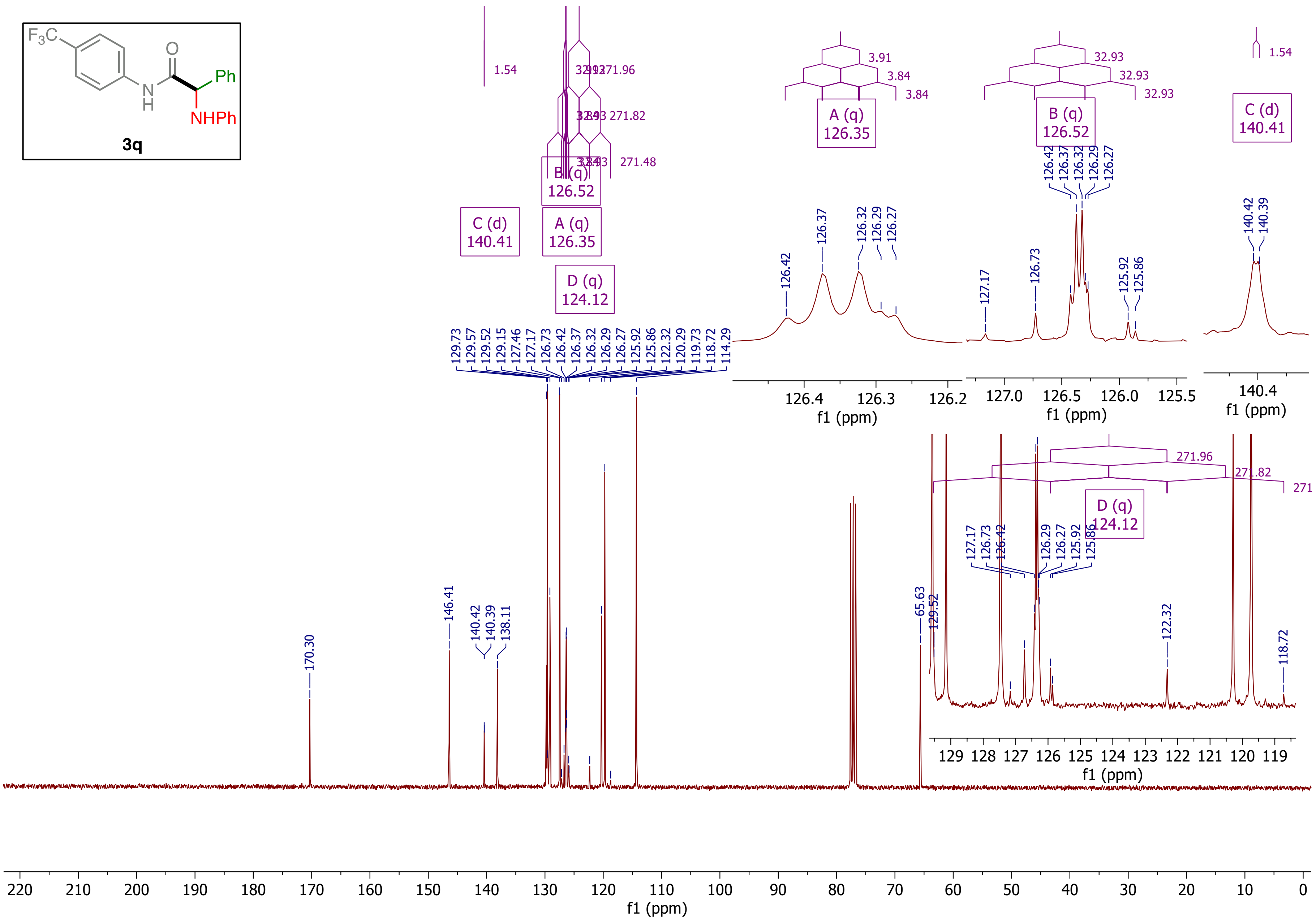

3q
F3C
Ph
NHPh
170.30
146.41
140.42
140.39
138.11
65.63
A (q)
126.35
B (q)
126.52
C (d)
140.41
D (q)
124.12
f1 (ppm)

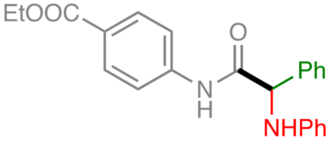

3r

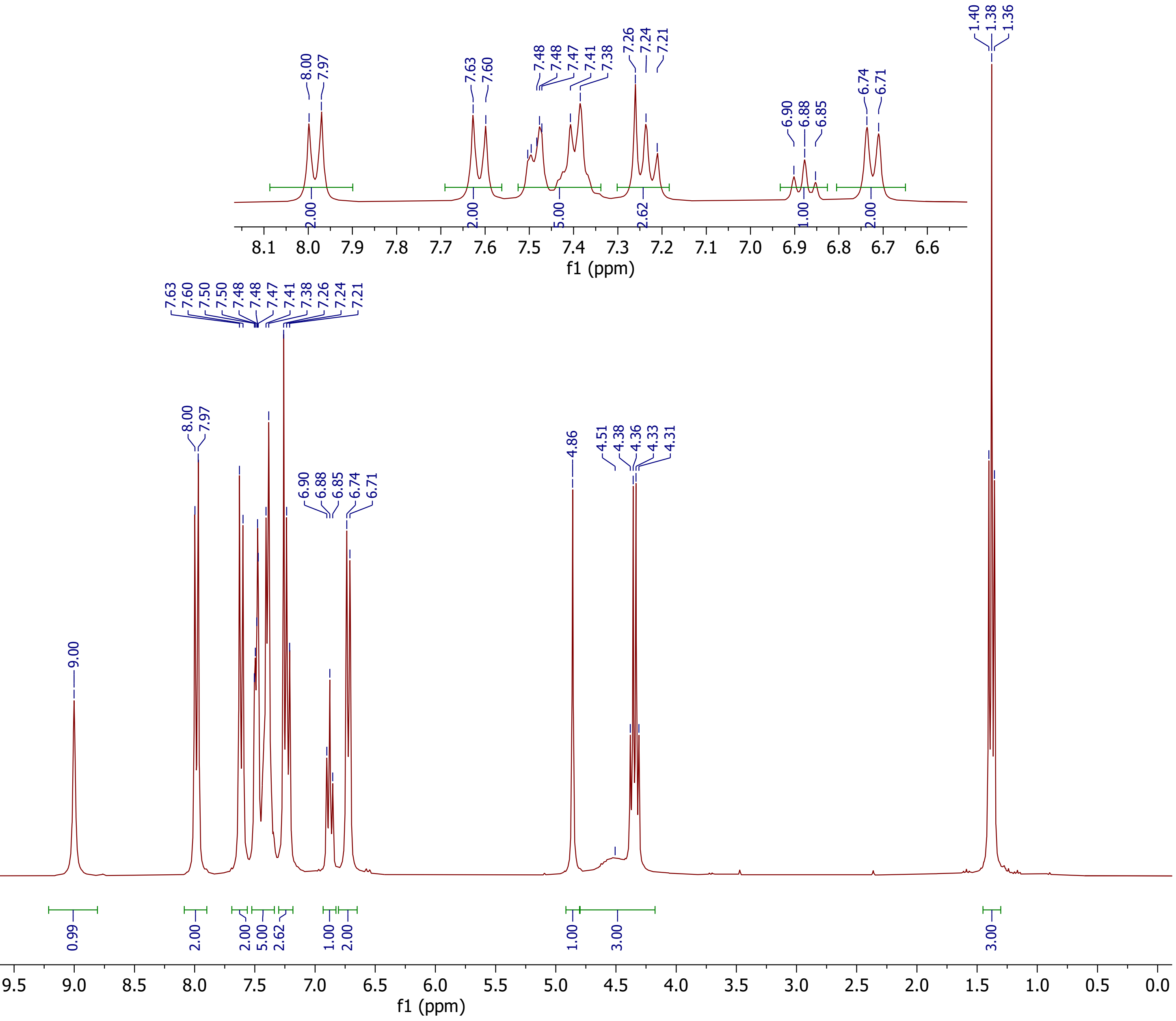

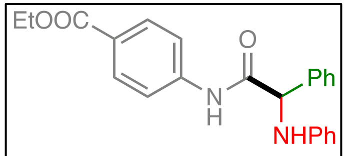
EtOOC
O
N
H
Ph
NHPh

3r

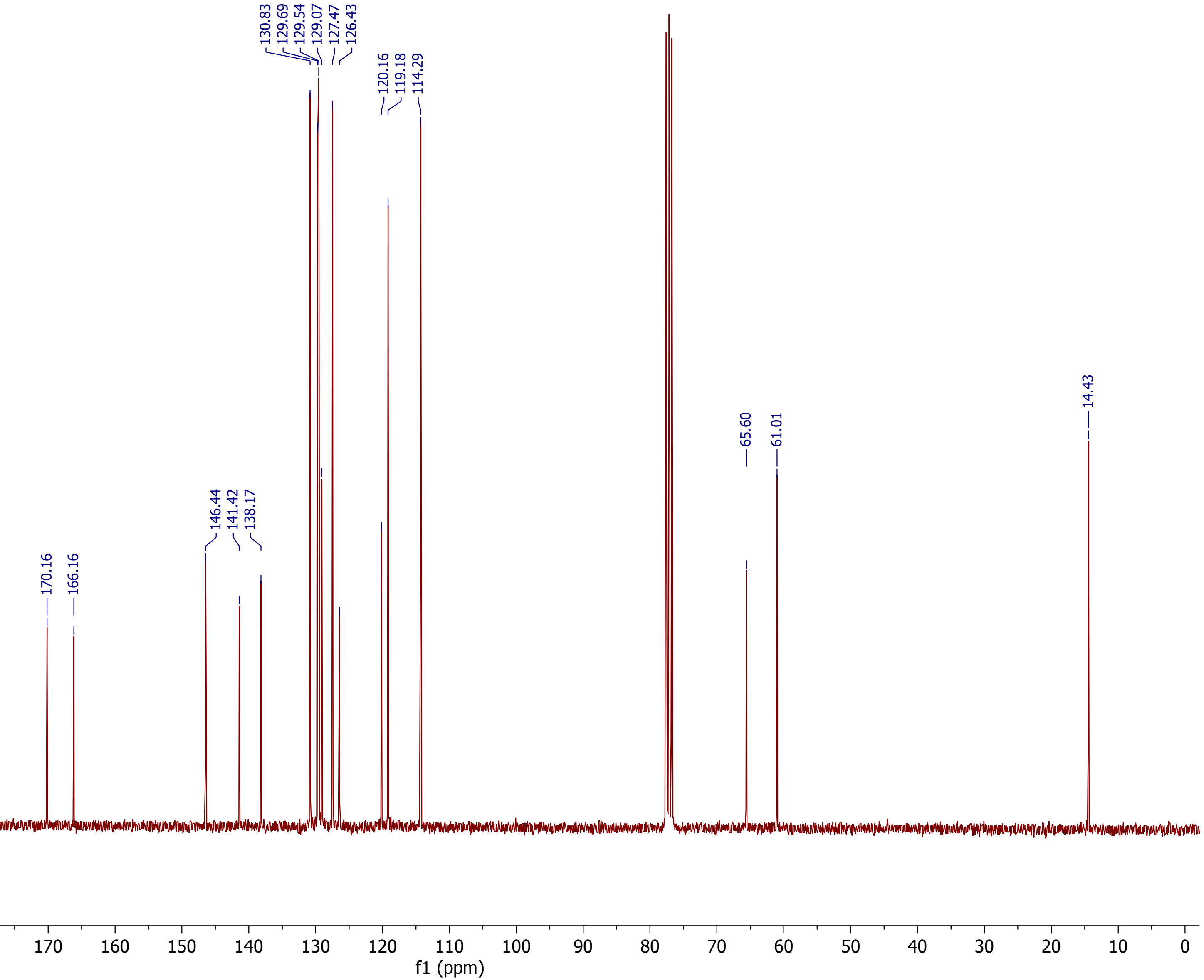
130.83
129.69
129.54
129.07
127.47
126.43
120.16
119.18
114.29
170.16
166.16
146.44
141.42
138.17
65.60
61.01
14.43
220 210 200 190 180 170 160 150 140 130 120 110 100 90 80 70 60 50 40 30 20 10 0
f1 (ppm)

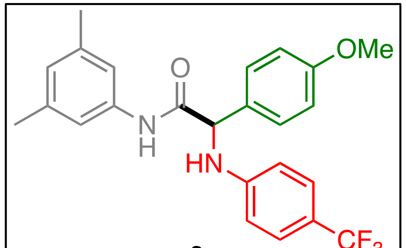

OMe
O
N
H
HN
CF3

3s

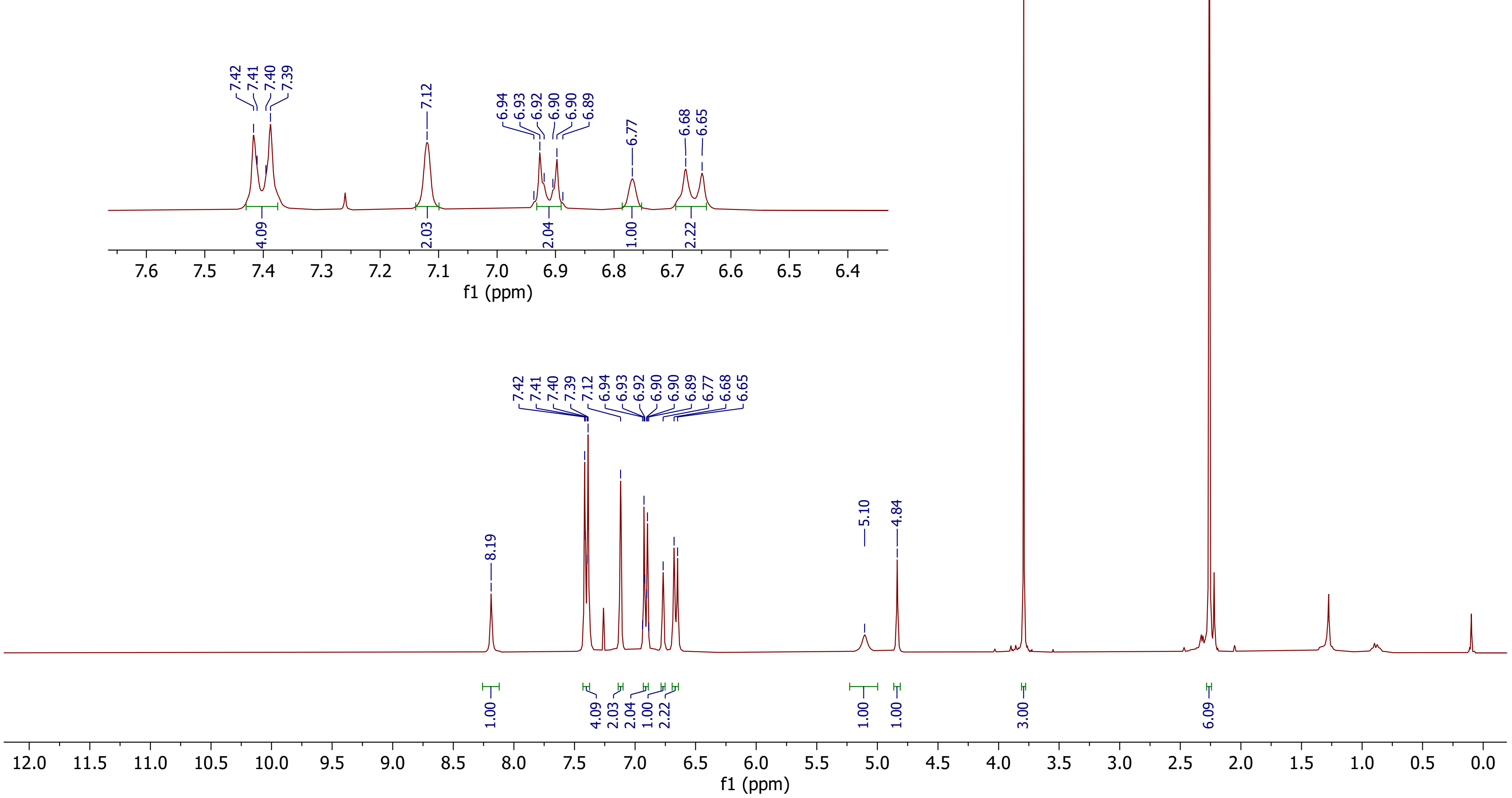

7.42
7.41
7.40
7.39
7.12
6.94
6.93
6.92
6.90
6.90
6.89
6.77
6.68
6.65
4.09
2.03
2.04
1.00
2.22
7.6
7.5
7.4
7.3
7.2
7.1
7.0
6.9
6.8
6.7
6.6
6.5
6.4
f1 (ppm)
8.19
5.10
4.84
3.79
2.26
1.00
1.00
1.00
3.00
6.09
12.0
11.5
11.0
10.5
10.0
9.5
9.0
8.5
8.0
7.5
7.0
6.5
6.0
5.5
5.0
4.5
4.0
3.5
3.0
2.5
2.0
1.5
1.0
0.5
0.0
f1 (ppm)

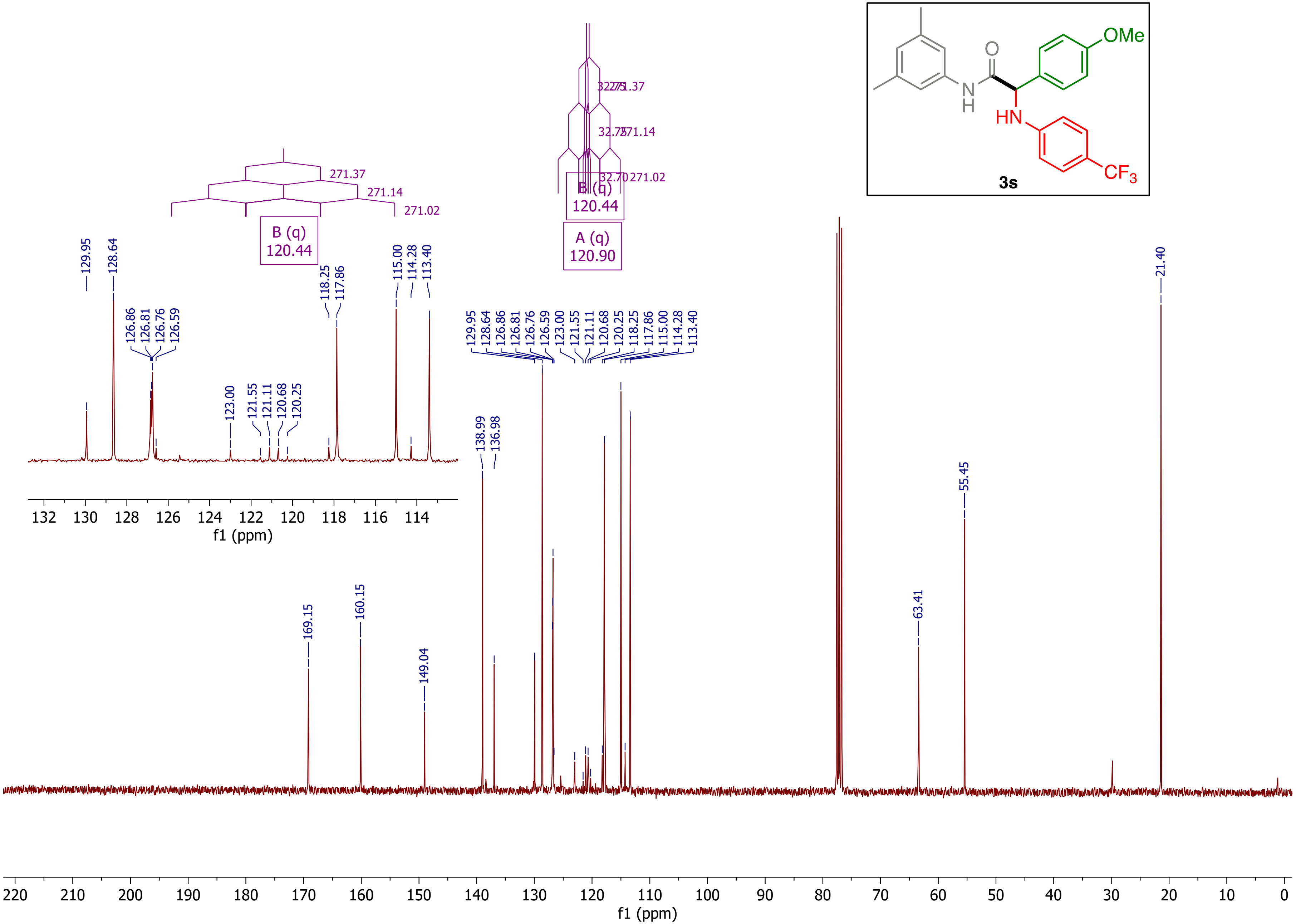

OMe
O
N
H
HN
CF3
3s
129.95
128.64
126.86
126.81
126.76
126.59
123.00
121.55
121.11
120.68
120.25
118.25
117.86
115.00
114.28
113.40
B (q)
120.44
A (q)
120.90
271.37
271.14
271.02
138.99
136.98
169.15
160.15
149.04
63.41
55.45
21.40
f1 (ppm)

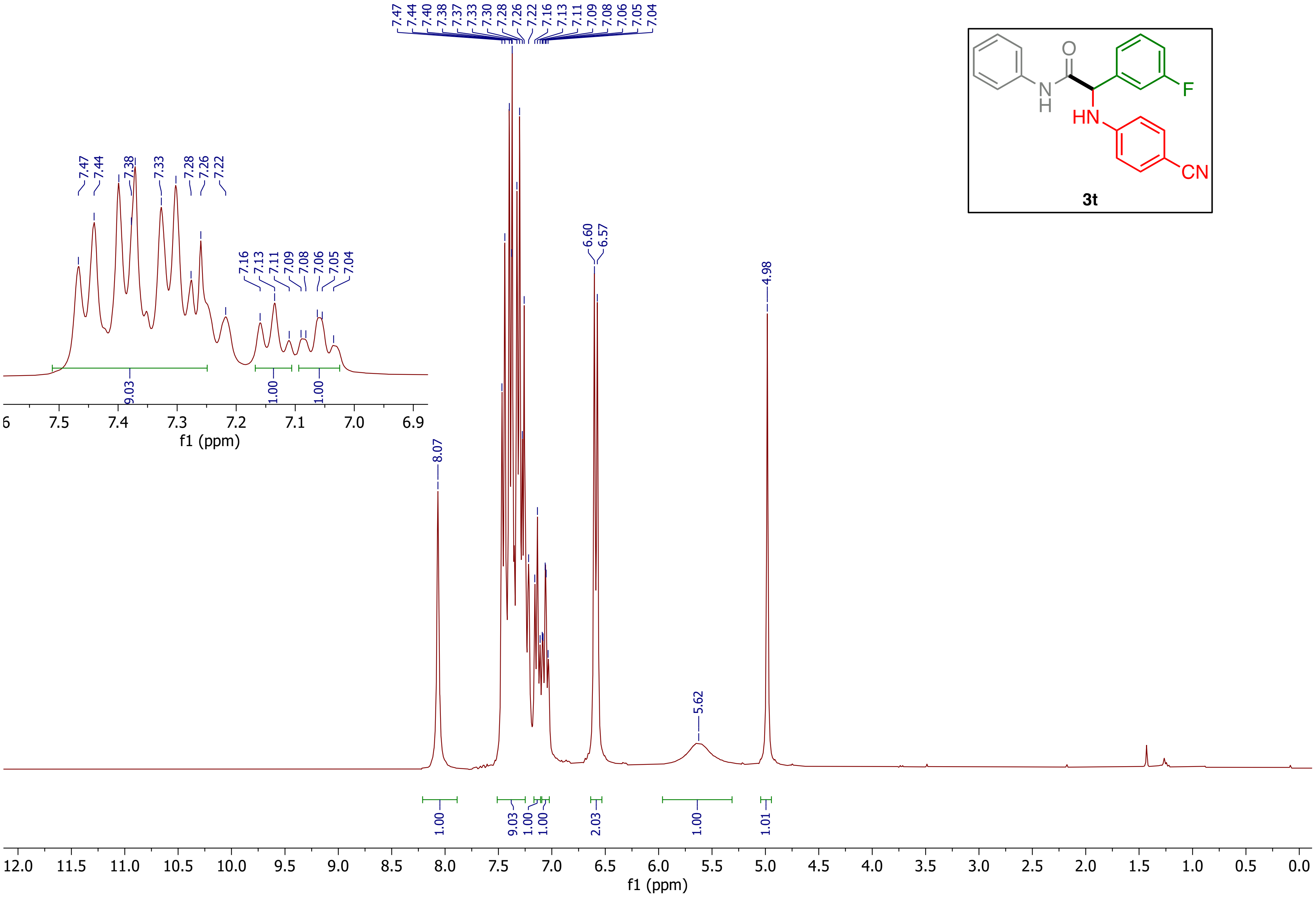

7.47
7.44
7.40
7.38
7.37
7.33
7.30
7.28
7.26
7.22
7.16
7.13
7.11
7.09
7.08
7.06
7.05
7.04
6.60
6.57
4.98
8.07
5.62
9.03
1.00
1.00
1.00
2.03
1.01
f1 (ppm)
O
N
H
F
HN
CN
3t

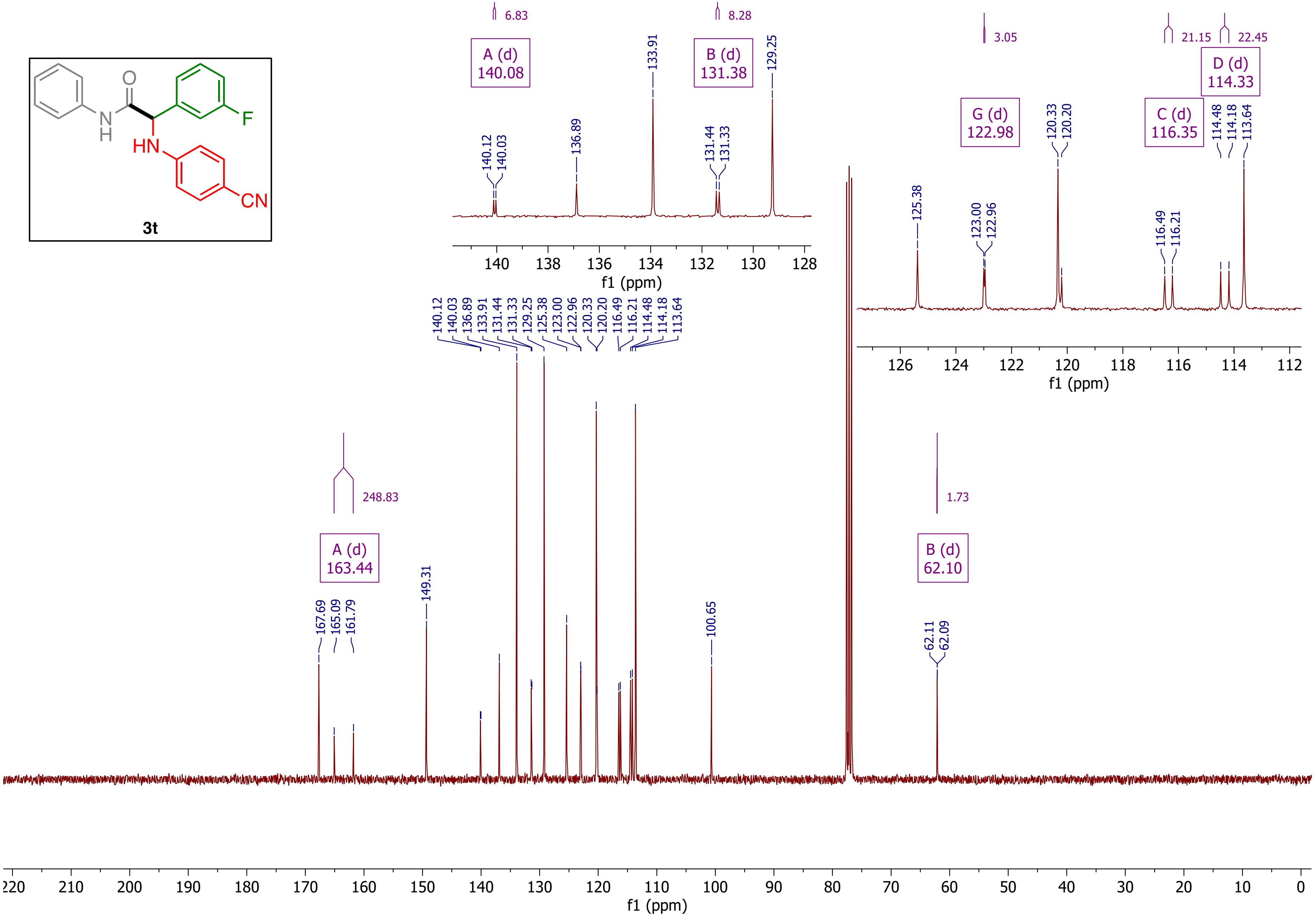

3t
CN
F
HN
A (d)
140.08
B (d)
131.38
G (d)
122.98
C (d)
116.35
D (d)
114.33
A (d)
163.44
B (d)
62.10
6.83
8.28
3.05
21.15
22.45
248.83
1.73
140.12
140.03
136.89
133.91
131.44
131.33
129.25
125.38
123.00
122.96
120.33
120.20
116.49
116.21
114.48
114.18
113.64
167.69
165.09
161.79
149.31
100.65
62.11
62.09
f1 (ppm)

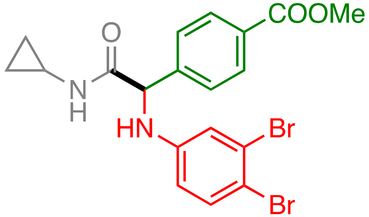

3u

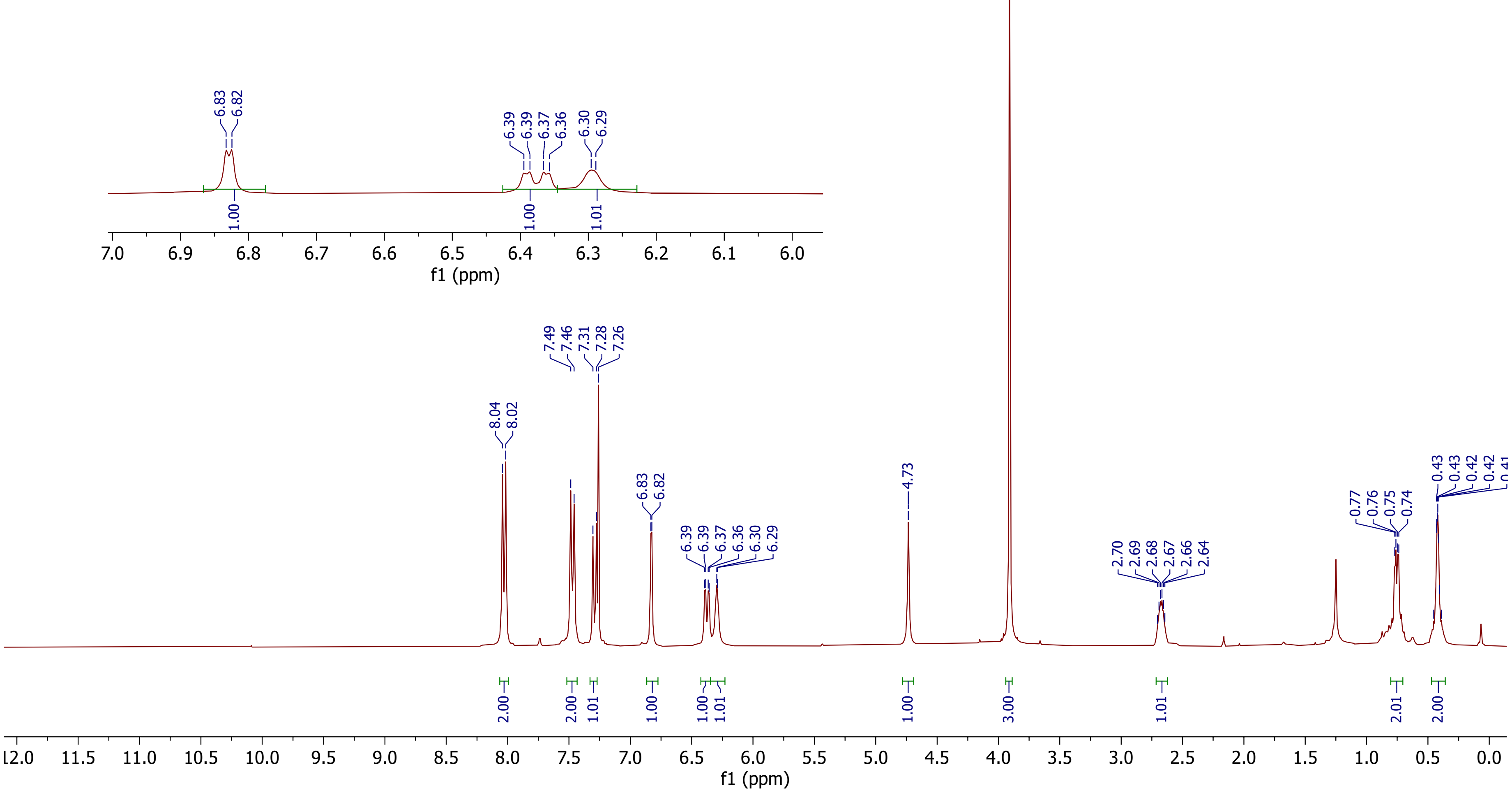

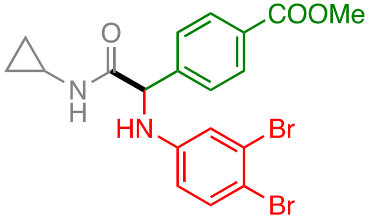

COOMe
O
N
H
HN
Br
Br

3u

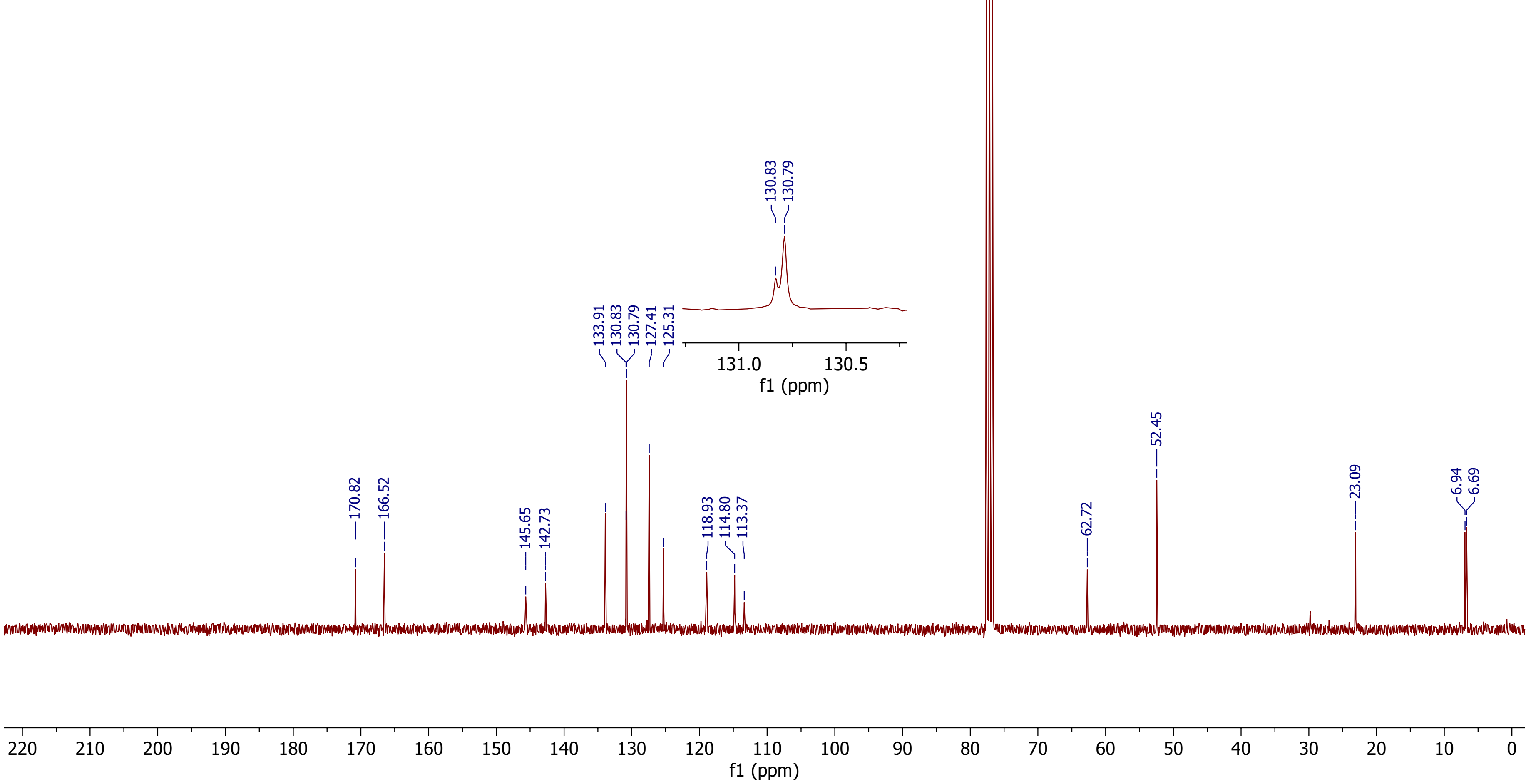

170.82
166.52
145.65
142.73
133.91
130.83
130.79
127.41
125.31
118.93
114.80
113.37
62.72
52.45
23.09
6.94
6.69
131.0
130.5
f1 (ppm)
220
210
200
190
180
170
160
150
140
130
120
110
100
90
80
70
60
50
40
30
20
10
0
f1 (ppm)

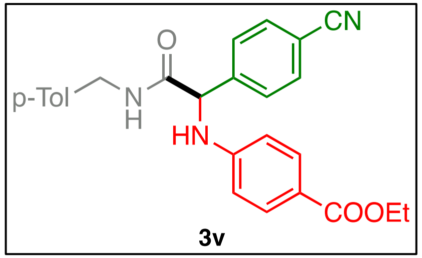

O
CN
p-Tol
N
H
HN
COOEt
3v

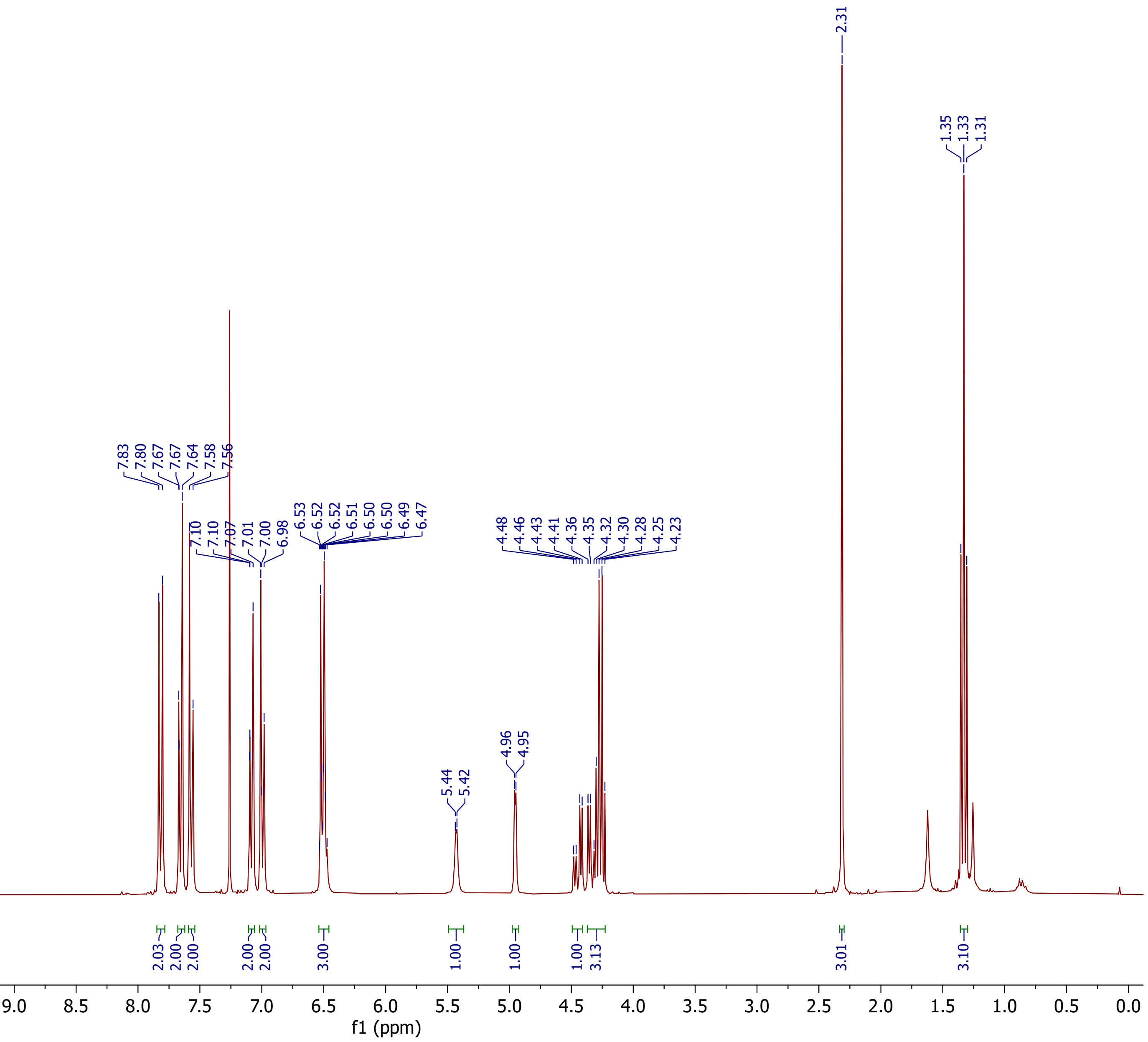

2.31
1.35
1.33
1.31
7.83
7.80
7.67
7.67
7.64
7.58
7.56
7.10
7.10
7.07
7.01
7.00
6.98
6.53
6.52
6.52
6.51
6.50
6.50
6.49
6.47
4.48
4.46
4.43
4.41
4.36
4.35
4.32
4.30
4.28
4.25
4.23
4.96
4.95
5.44
5.42
2.03
2.00
2.00
2.00
2.00
3.00
1.00
1.00
1.00
3.13
3.01
3.10
f1 (ppm)

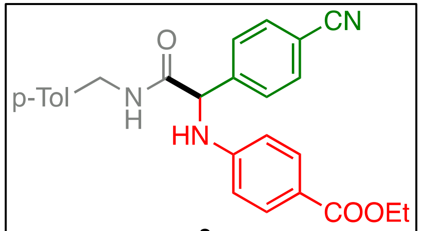

CN
O
p-Tol
N
H
HN
COOEt

3v

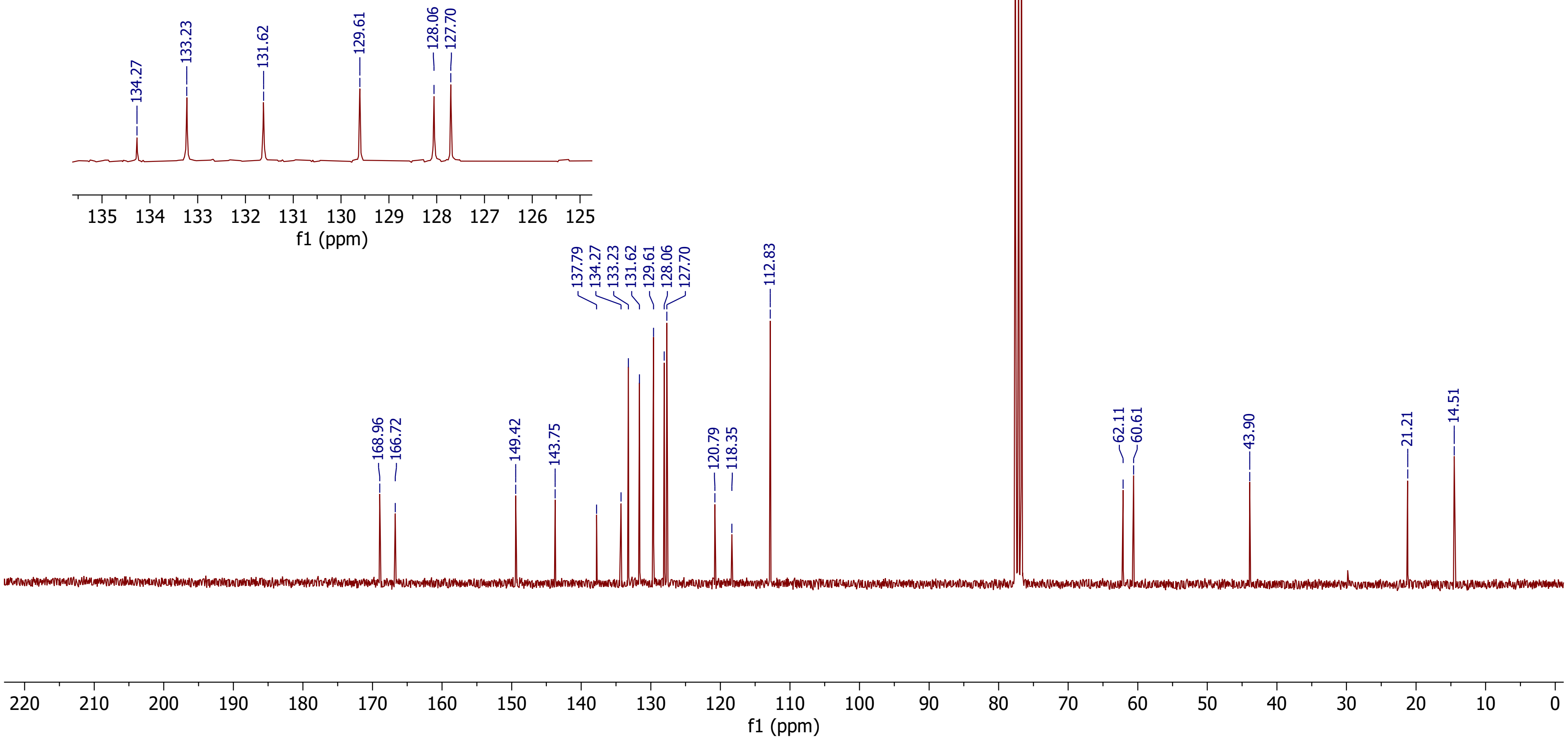

134.27
133.23
131.62
129.61
128.06
127.70
135 134 133 132 131 130 129 128 127 126 125
f1 (ppm)
168.96
166.72
149.42
143.75
137.79
134.27
133.23
131.62
129.61
128.06
127.70
120.79
118.35
112.83
62.11
60.61
43.90
21.21
14.51
220 210 200 190 180 170 160 150 140 130 120 110 100 90 80 70 60 50 40 30 20 10 0
f1 (ppm)

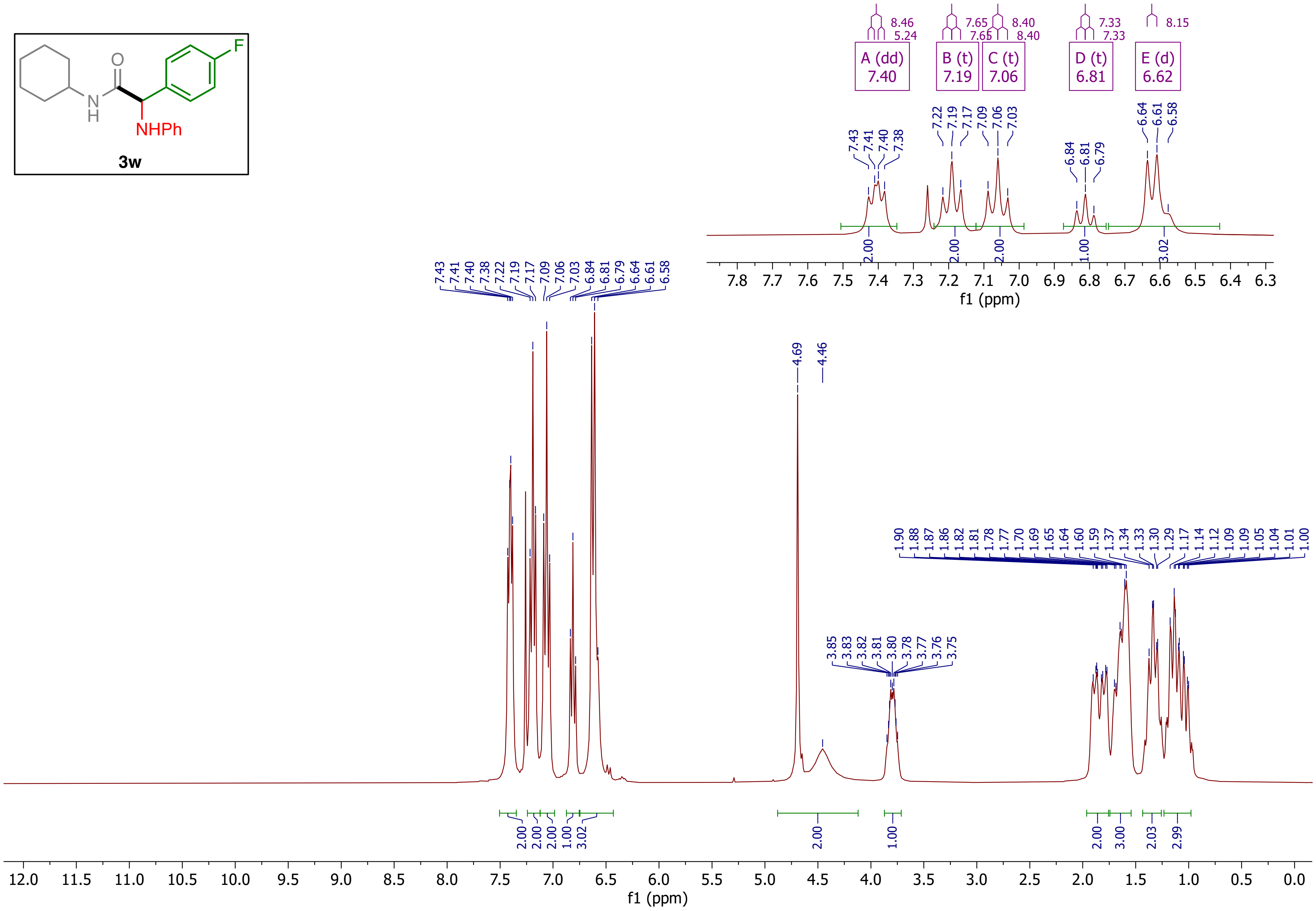
NHPh
3w
A (dd)
7.40
B (t)
7.19
C (t)
7.06
D (t)
6.81
E (d)
6.62
f1 (ppm)

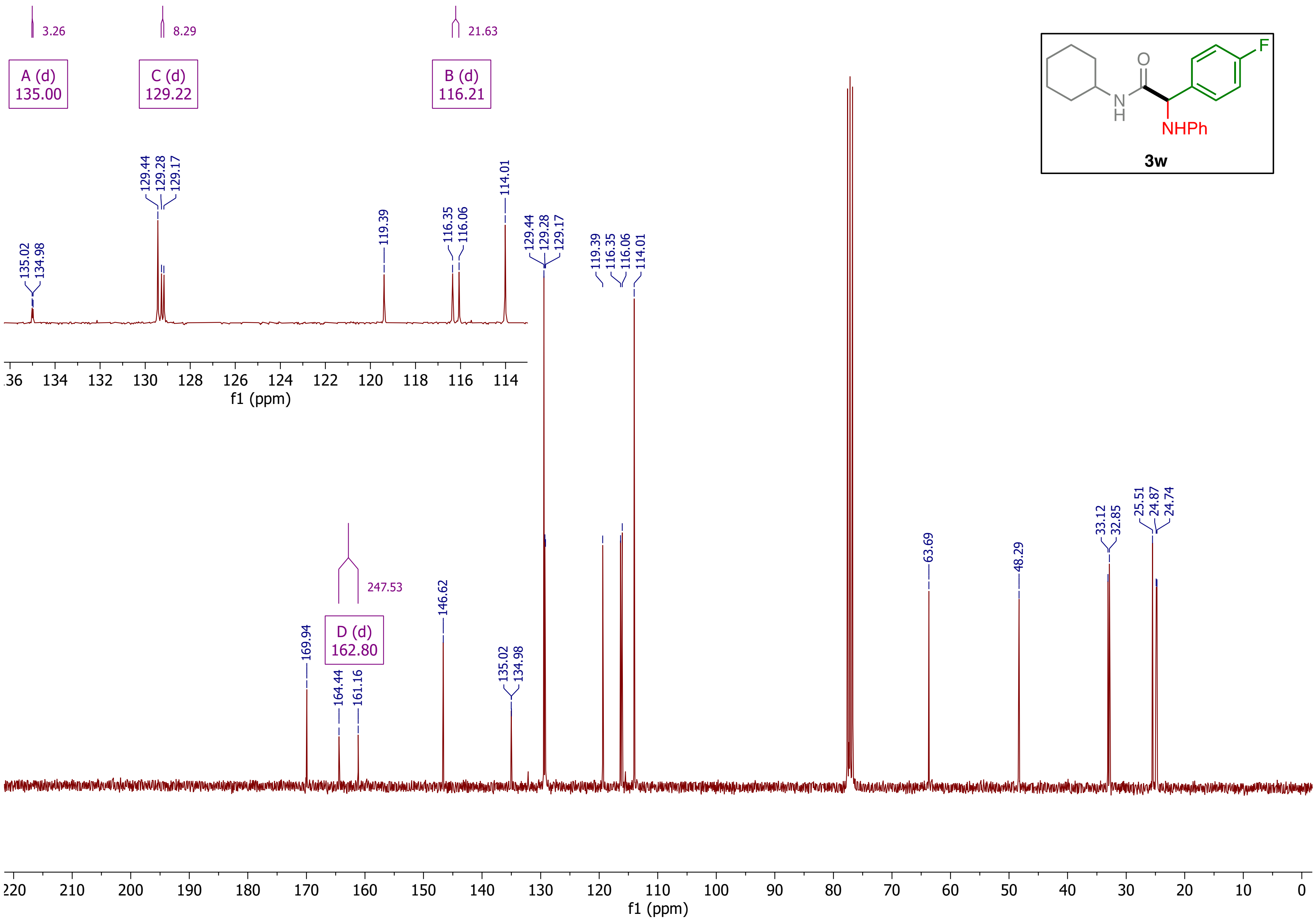
3.26
8.29
21.63
A (d)
135.00
C (d)
129.22
B (d)
116.21
135.02
134.98
129.44
129.28
129.17
119.39
116.35
116.06
114.01
f1 (ppm)
247.53
D (d)
162.80
169.94
164.44
161.16
146.62
63.69
48.29
33.12
32.85
25.51
24.87
24.74
O
F
N
H
NHPh
3w

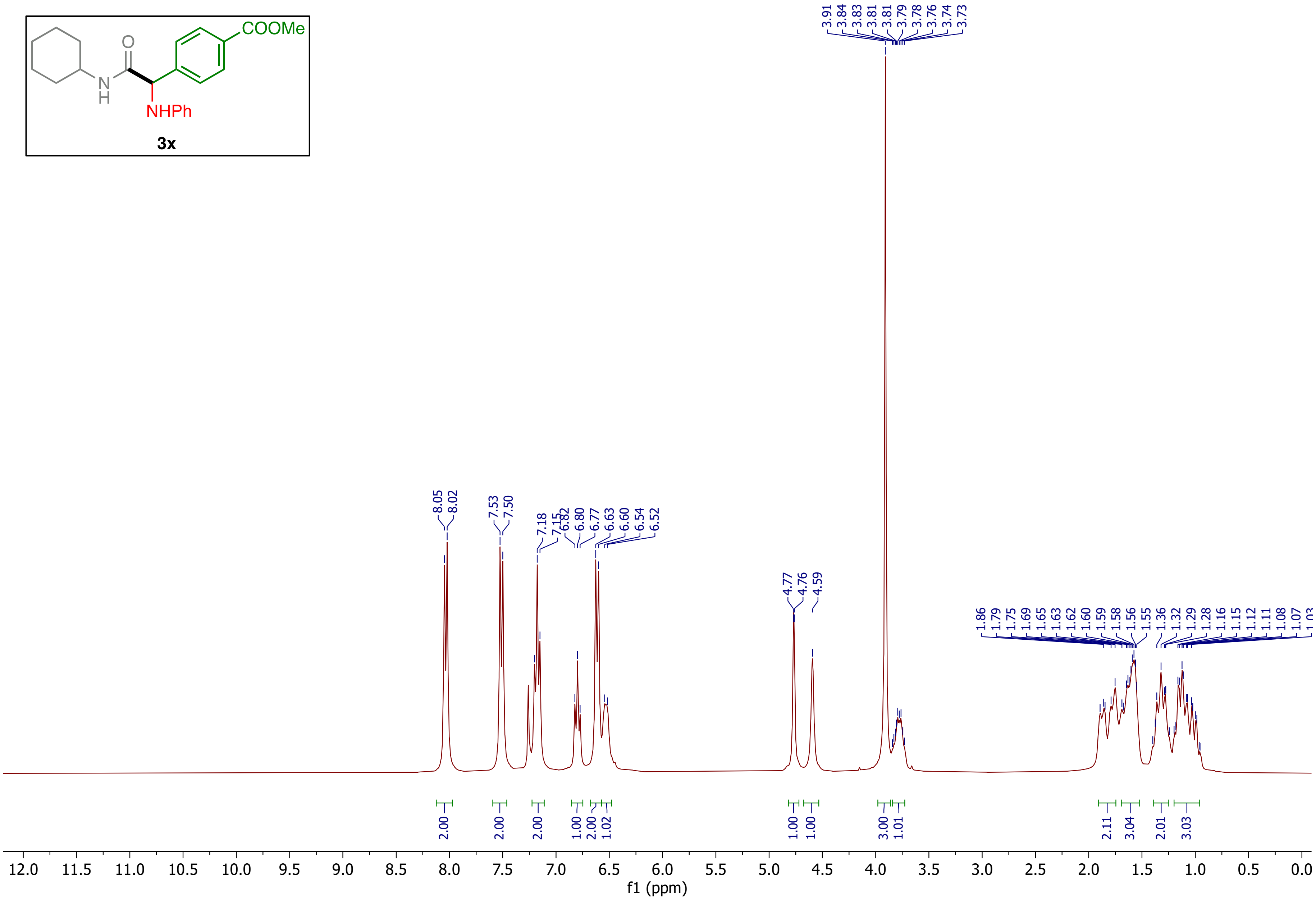
COOMe
O
N
H
NHPh
3x
f1 (ppm)

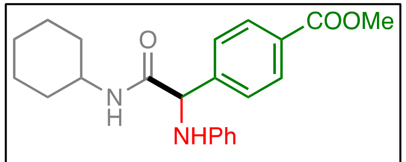
COOMe
O
N
H
NHPh

3x

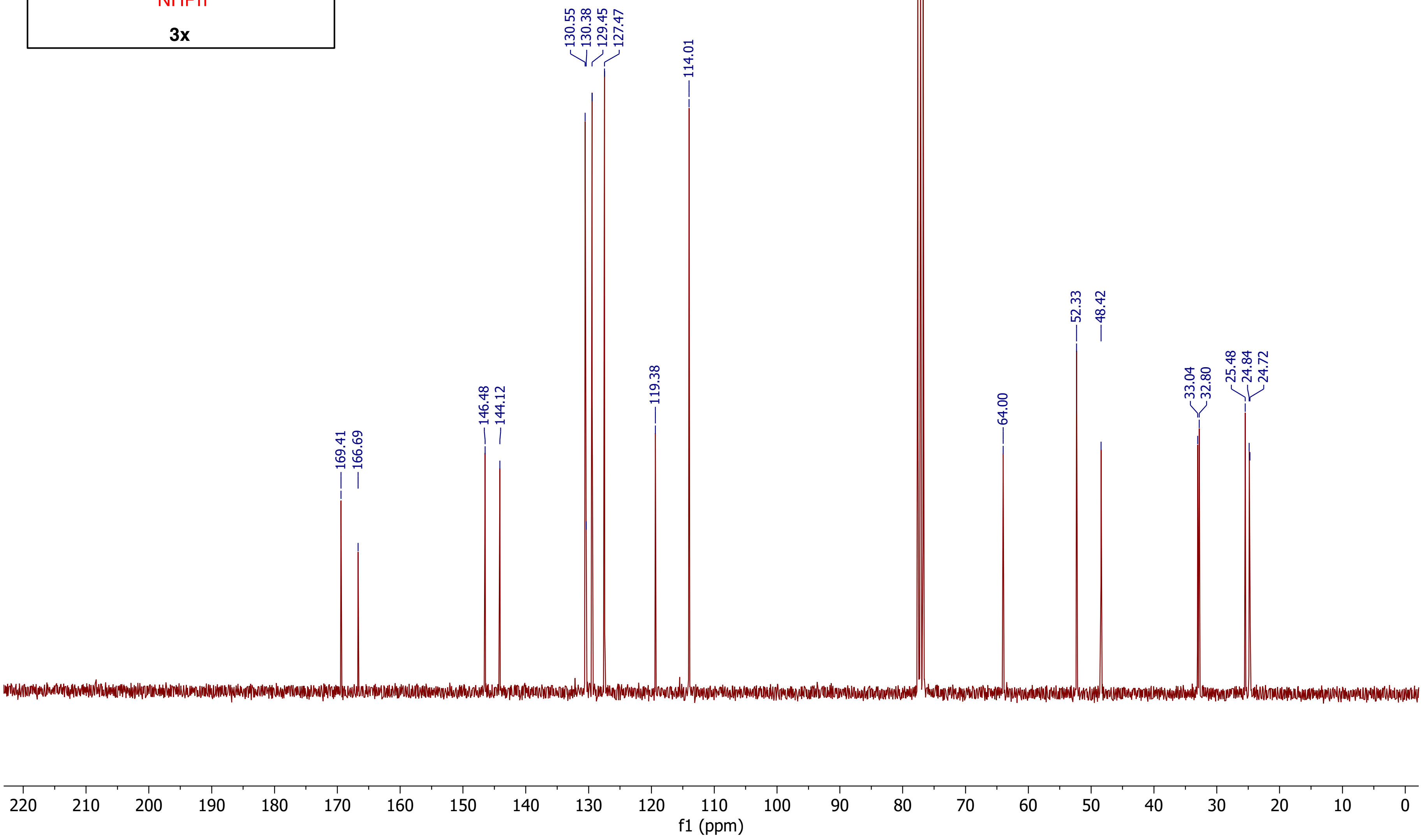
130.55
130.38
129.45
127.47
114.01
169.41
166.69
146.48
144.12
119.38
64.00
52.33
48.42
33.04
32.80
25.48
24.84
24.72
220 210 200 190 180 170 160 150 140 130 120 110 100 90 80 70 60 50 40 30 20 10 0
f1 (ppm)

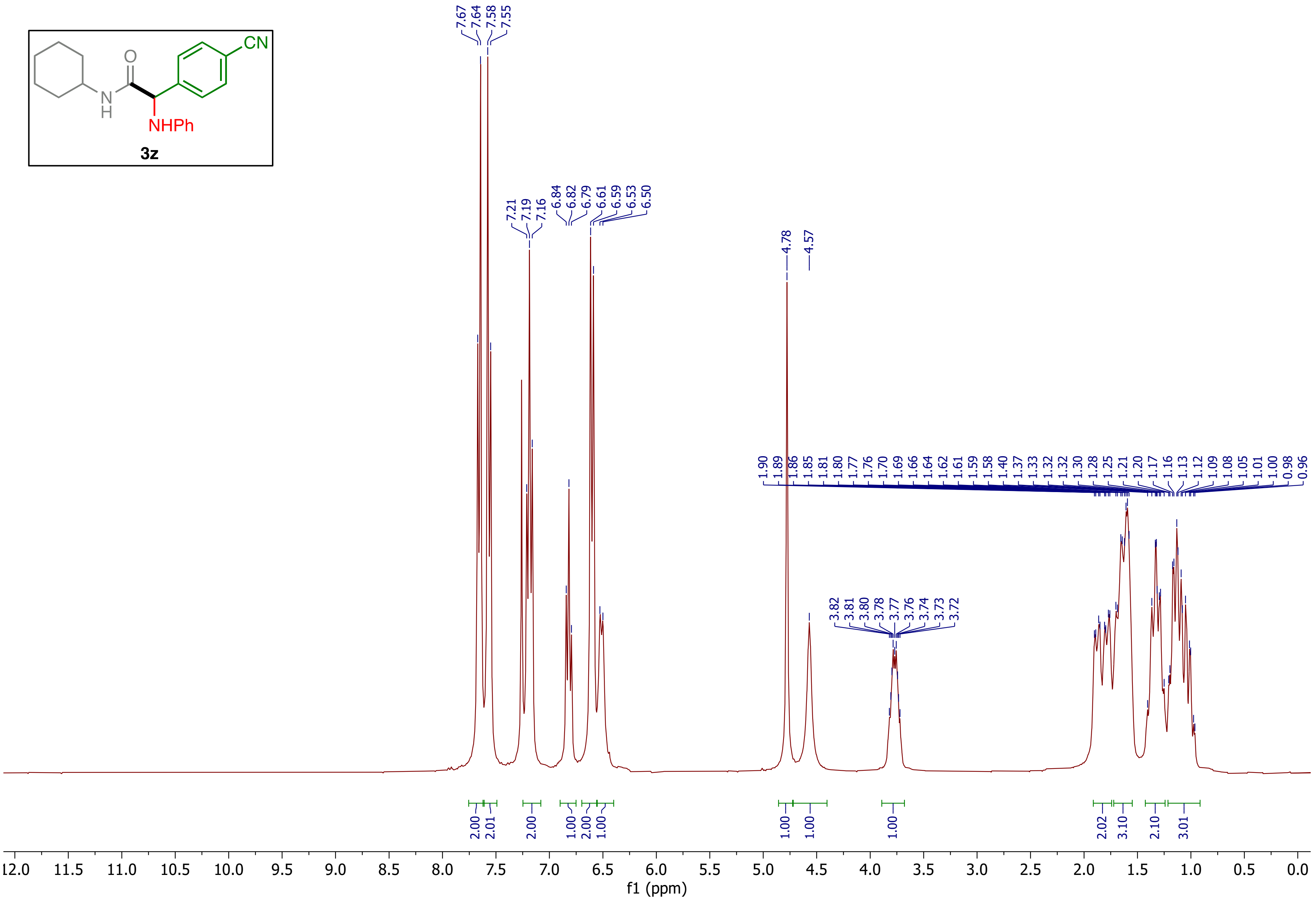

CN
O
N
H
NHPh
3z
7.67
7.64
7.58
7.55
7.21
7.19
7.16
6.84
6.82
6.79
6.61
6.59
6.53
6.50
4.78
4.57
3.82
3.81
3.80
3.78
3.77
3.76
3.74
3.73
3.72
1.90
1.89
1.86
1.85
1.81
1.80
1.77
1.76
1.70
1.69
1.66
1.64
1.62
1.61
1.59
1.58
1.40
1.37
1.33
1.32
1.32
1.30
1.28
1.25
1.21
1.20
1.17
1.16
1.13
1.12
1.09
1.08
1.05
1.01
1.00
0.98
0.96
2.00
2.01
2.00
1.00
2.00
1.00
1.00
1.00
1.00
2.02
3.10
2.10
3.01
f1 (ppm)

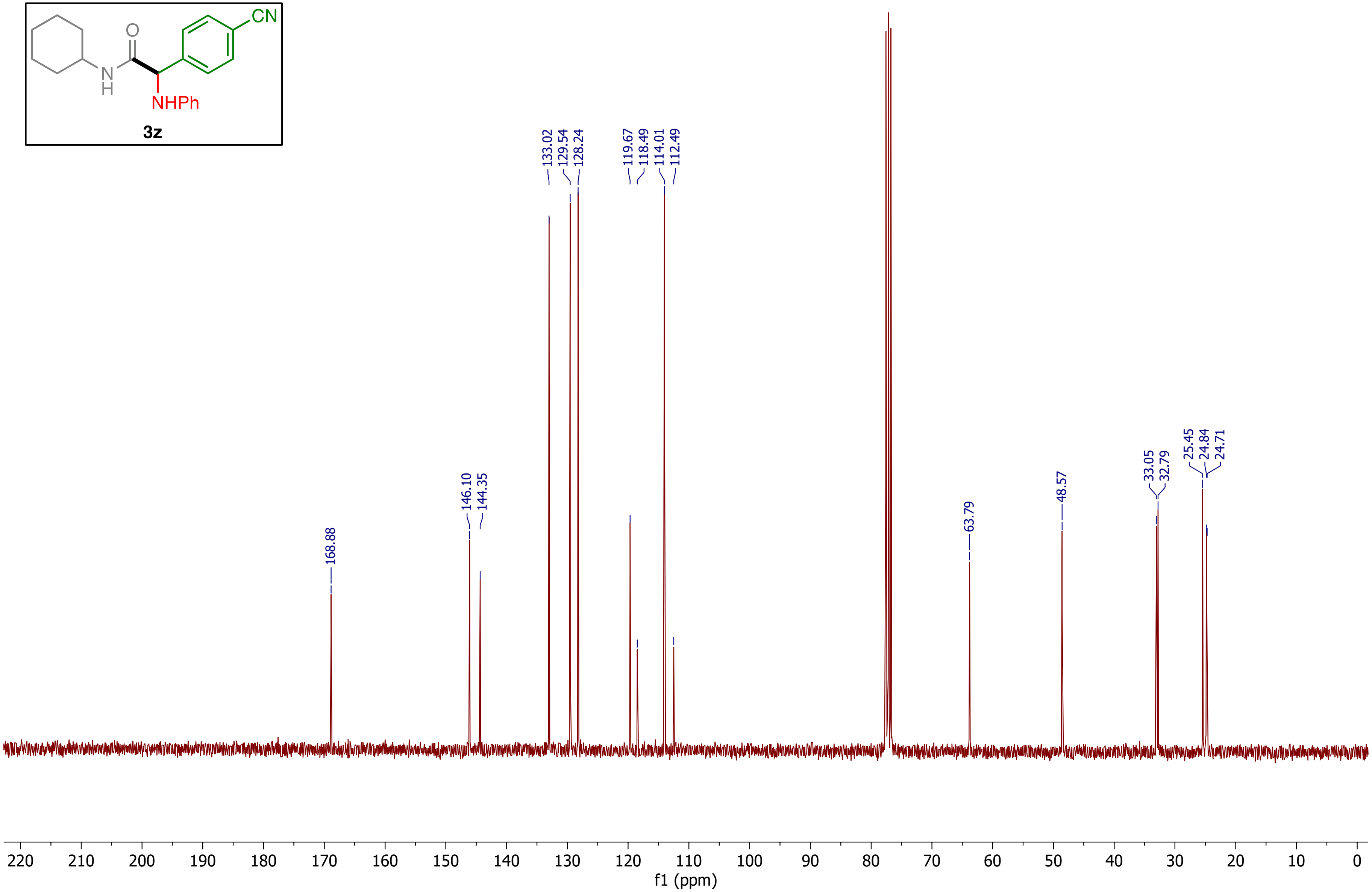

CN
O
N
H
NHPh
3z
133.02
129.54
128.24
119.67
118.49
114.01
112.49
168.88
146.10
144.35
63.79
48.57
33.05
32.79
25.45
24.84
24.71
220 210 200 190 180 170 160 150 140 130 120 110 100 90 80 70 60 50 40 30 20 10 0
f1 (ppm)

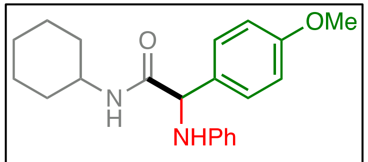

3aa

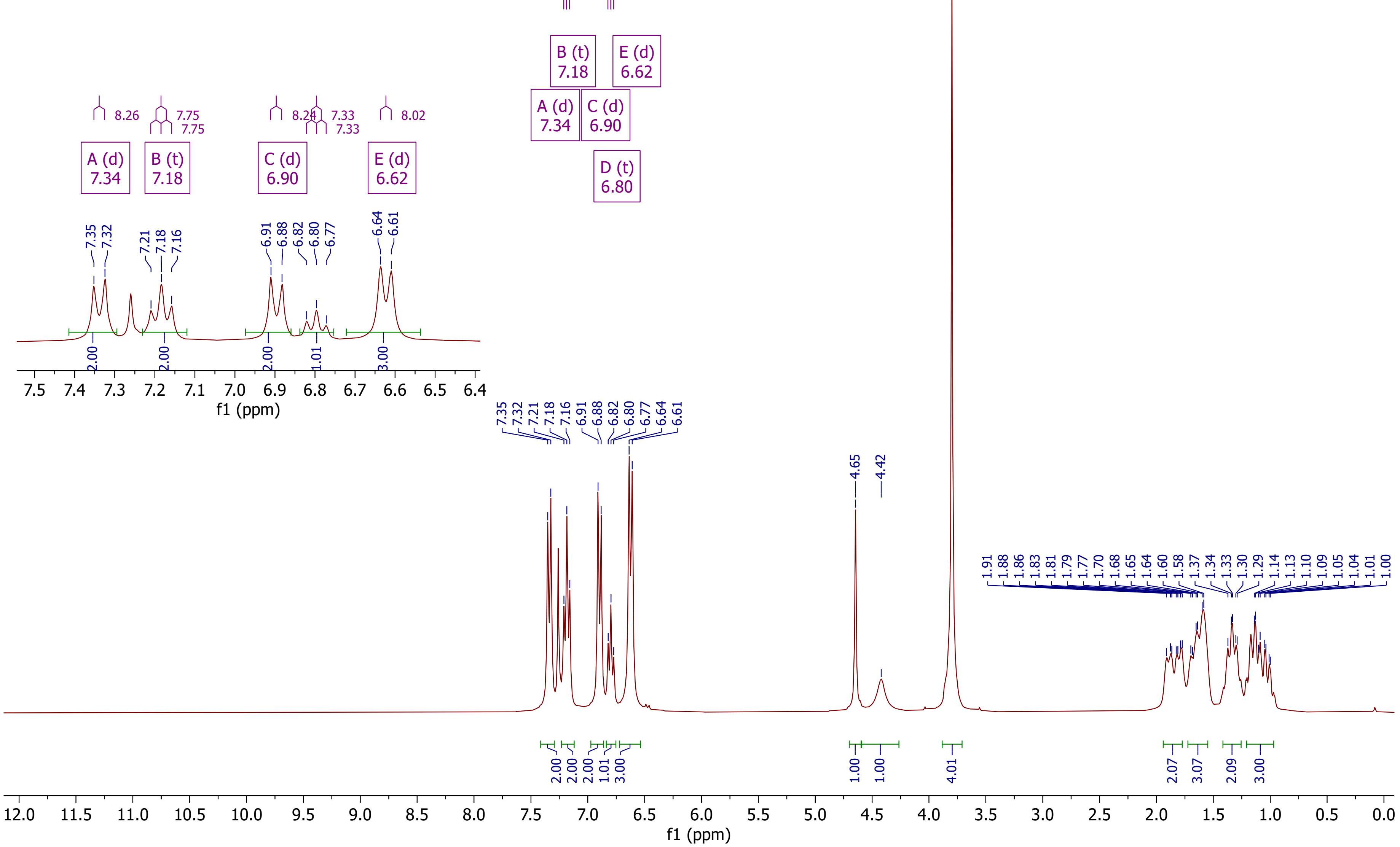

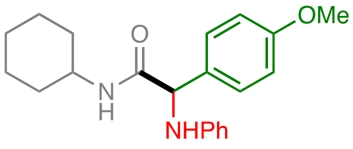
OMe
O
N
H
NHPh

3aa

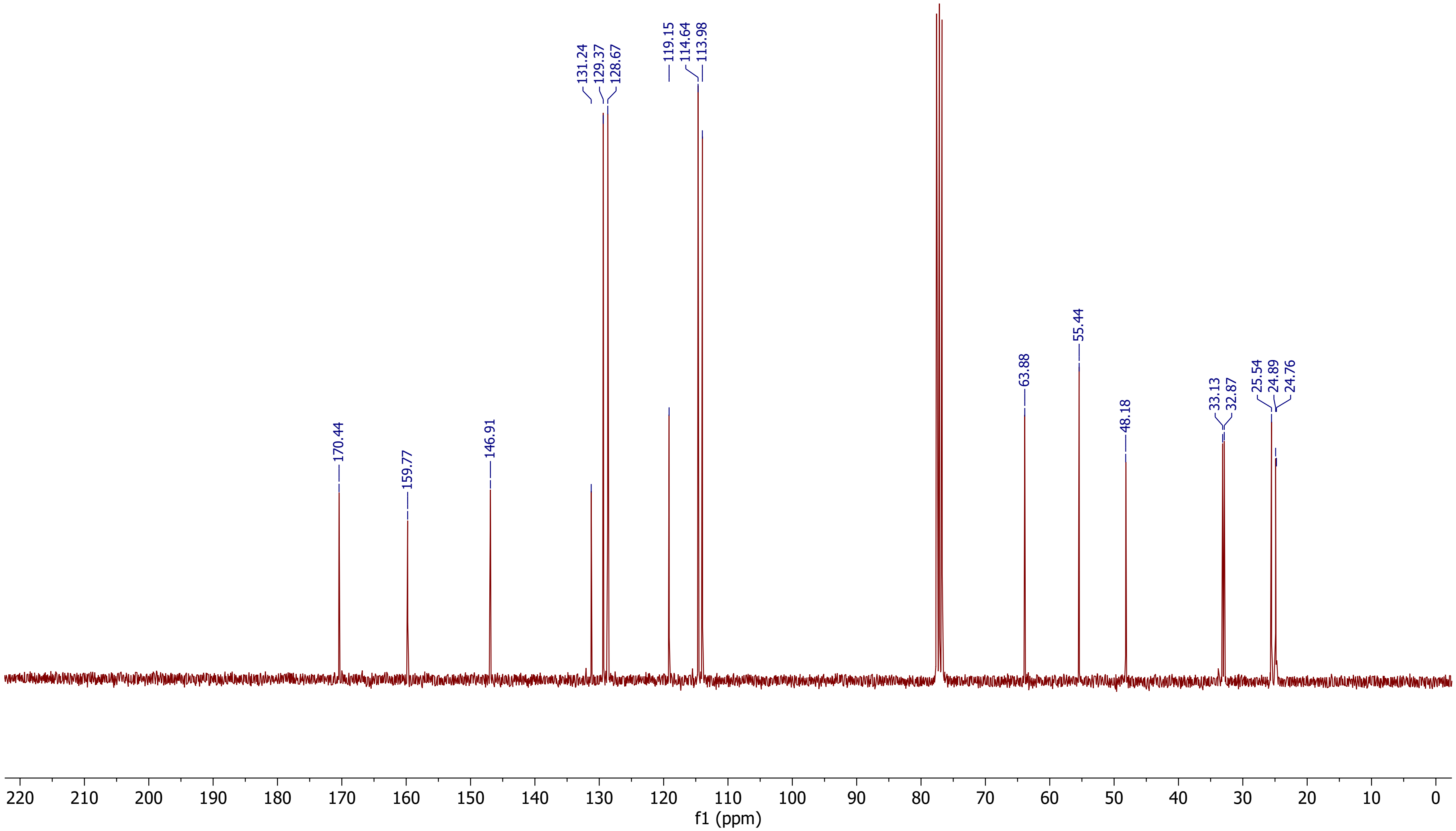
170.44
159.77
146.91
131.24
129.37
128.67
119.15
114.64
113.98
63.88
55.44
48.18
33.13
32.87
25.54
24.89
24.76
220
210
200
190
180
170
160
150
140
130
120
110
100
90
80
70
60
50
40
30
20
10
0
f1 (ppm)

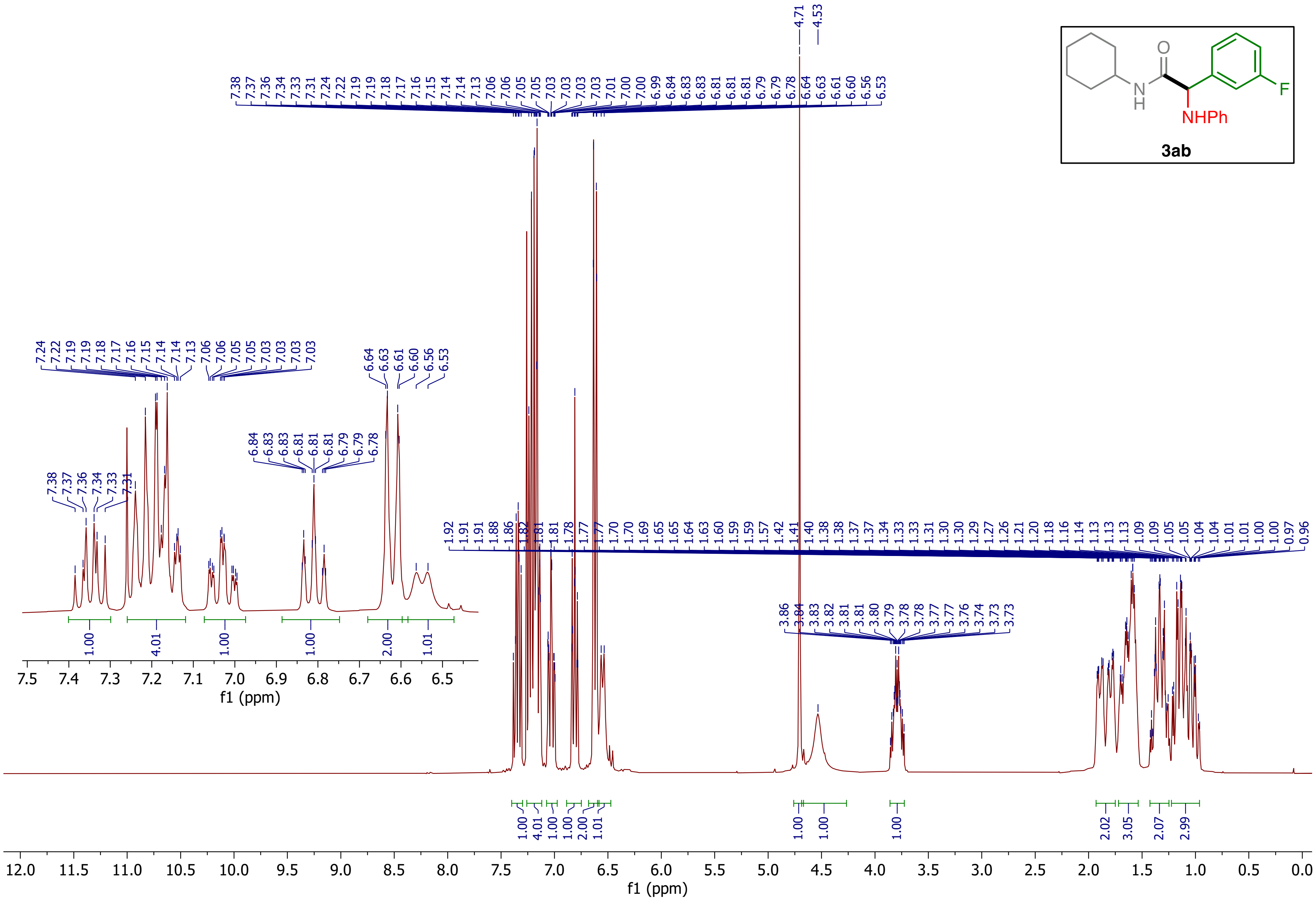
O
N
H
F
NHPh
3ab
f1 (ppm)

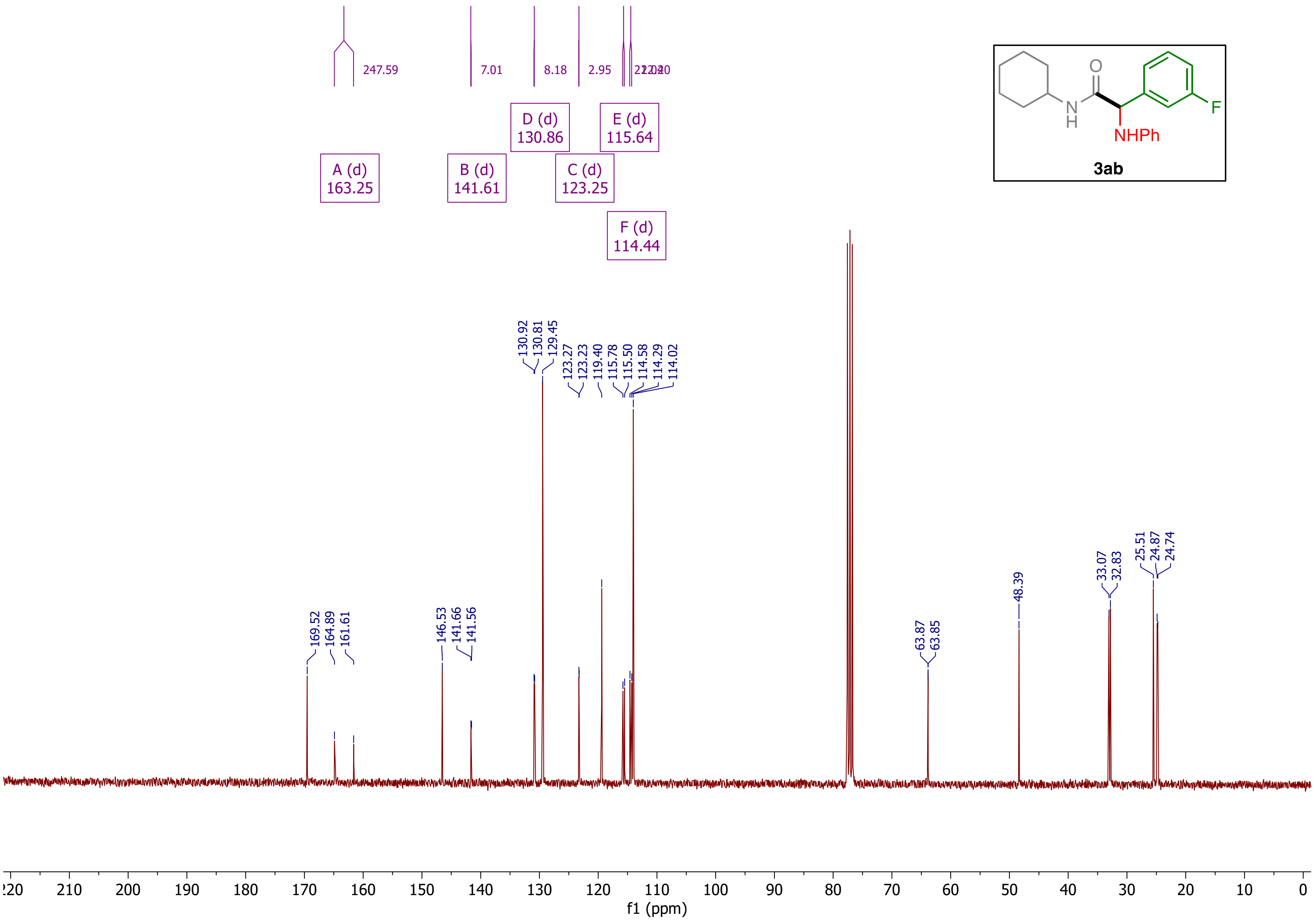

247.59
7.01
8.18
2.95
[illegible]
A (d) 163.25
B (d) 141.61
C (d) 123.25
D (d) 130.86
E (d) 115.64
F (d) 114.44
169.52
164.89
161.61
146.53
141.66
141.56
130.92
130.81
129.45
123.27
123.23
119.40
115.78
115.50
114.58
114.29
114.02
63.87
63.85
48.39
33.07
32.83
25.51
24.87
24.74
O
N
H
NHPh
F
3ab
f1 (ppm)

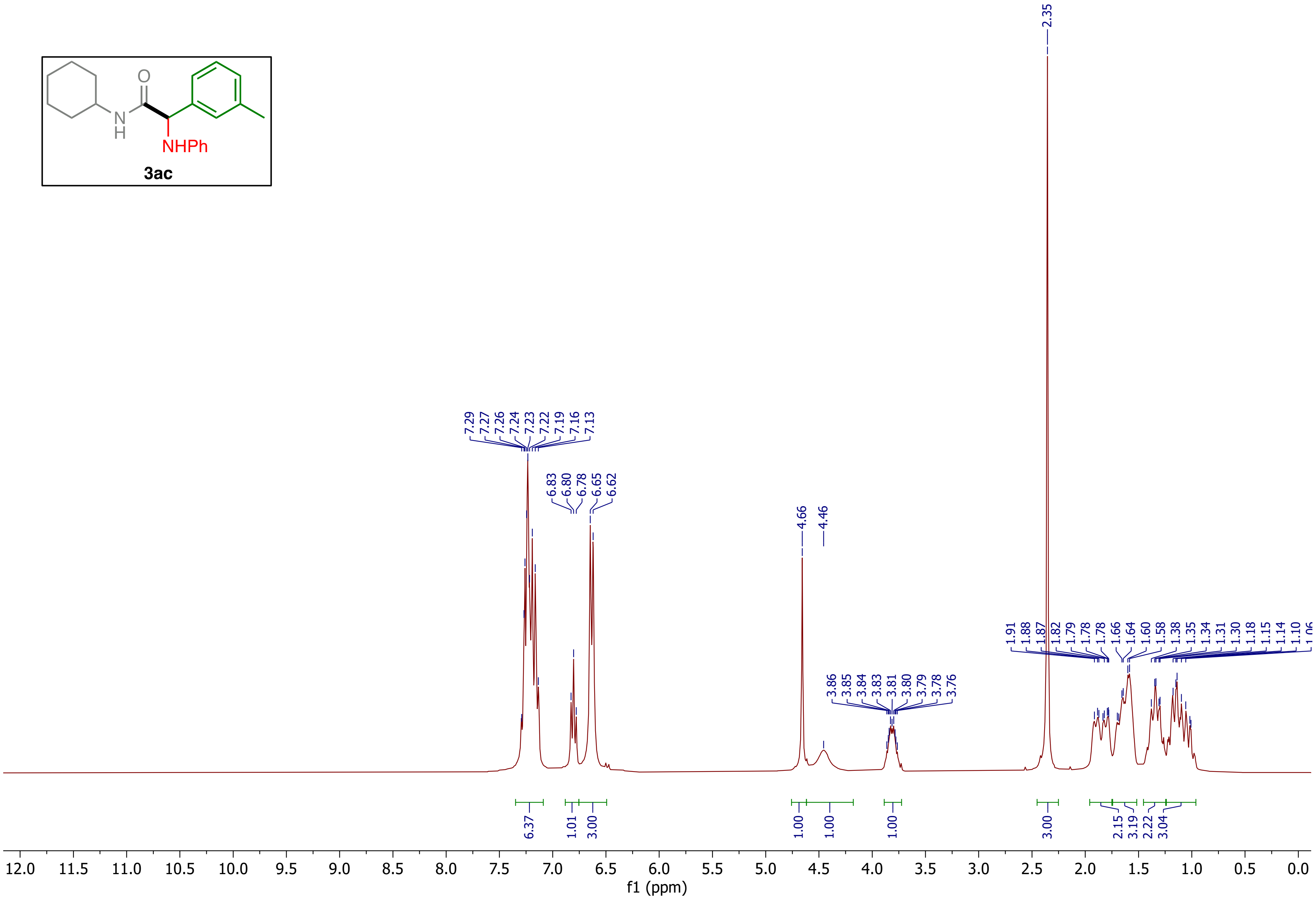
O
N
H
NHPh

3ac

7.29 7.27 7.26 7.24 7.23 7.22 7.19 7.16 7.13
6.83 6.80 6.78 6.65 6.62
4.66
4.46
3.86 3.85 3.84 3.83 3.81 3.80 3.79 3.78 3.76
2.35
1.91 1.88 1.87 1.82 1.79 1.78 1.66 1.64 1.60 1.58 1.38 1.35 1.34 1.31 1.30 1.18 1.15 1.14 1.10
6.37
1.01
3.00
1.00
1.00
1.00
3.00
2.15
3.19
2.22
3.04
12.0 11.5 11.0 10.5 10.0 9.5 9.0 8.5 8.0 7.5 7.0 6.5 6.0 5.5 5.0 4.5 4.0 3.5 3.0 2.5 2.0 1.5 1.0 0.5 0.0
f1 (ppm)

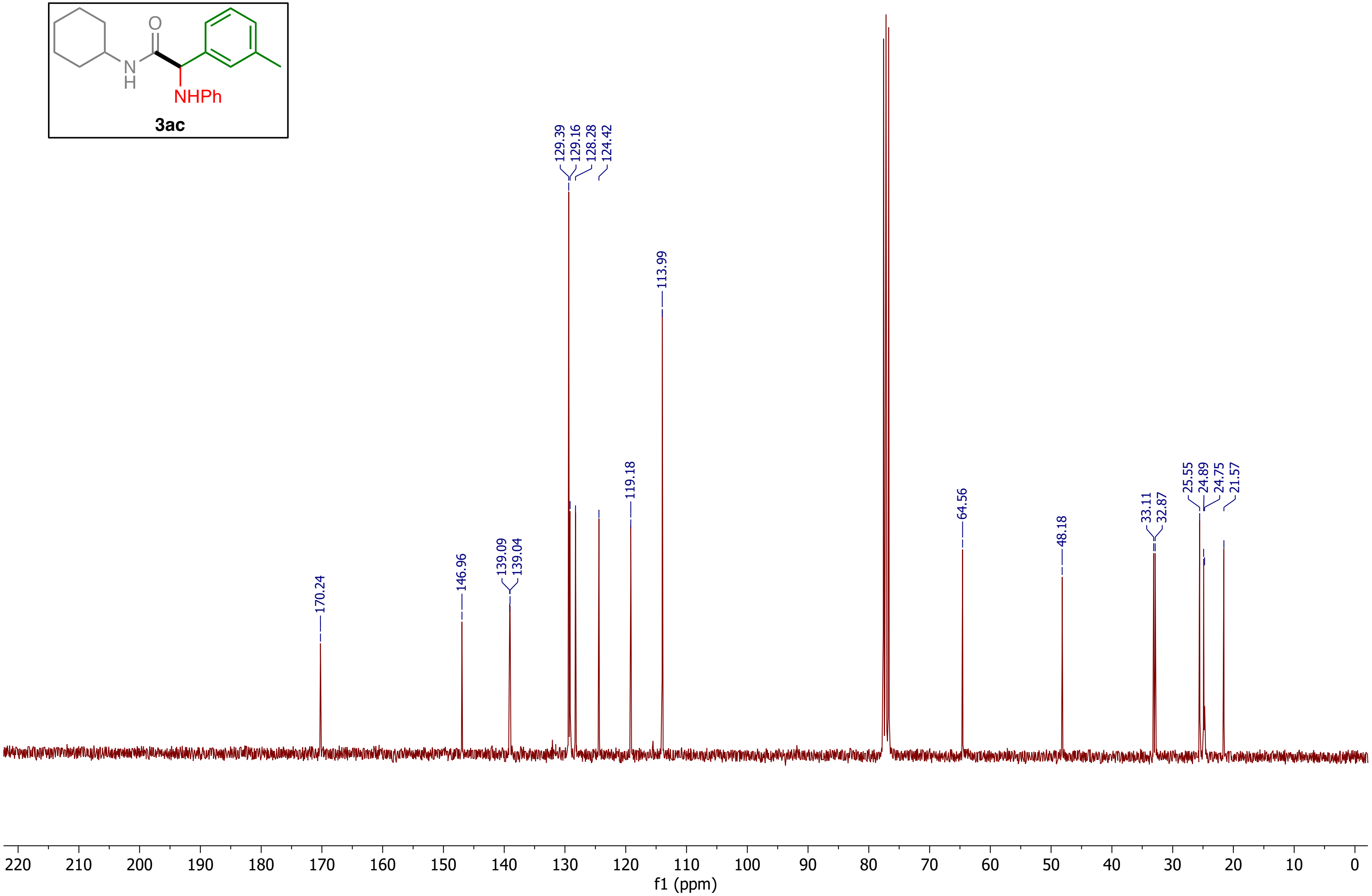

O
N
H
NHPh
3ac
170.24
146.96
139.09
139.04
129.39
129.16
128.28
124.42
119.18
113.99
64.56
48.18
33.11
32.87
25.55
24.89
24.75
21.57
220
210
200
190
180
170
160
150
140
130
120
110
100
90
80
70
60
50
40
30
20
10
0
f1 (ppm)

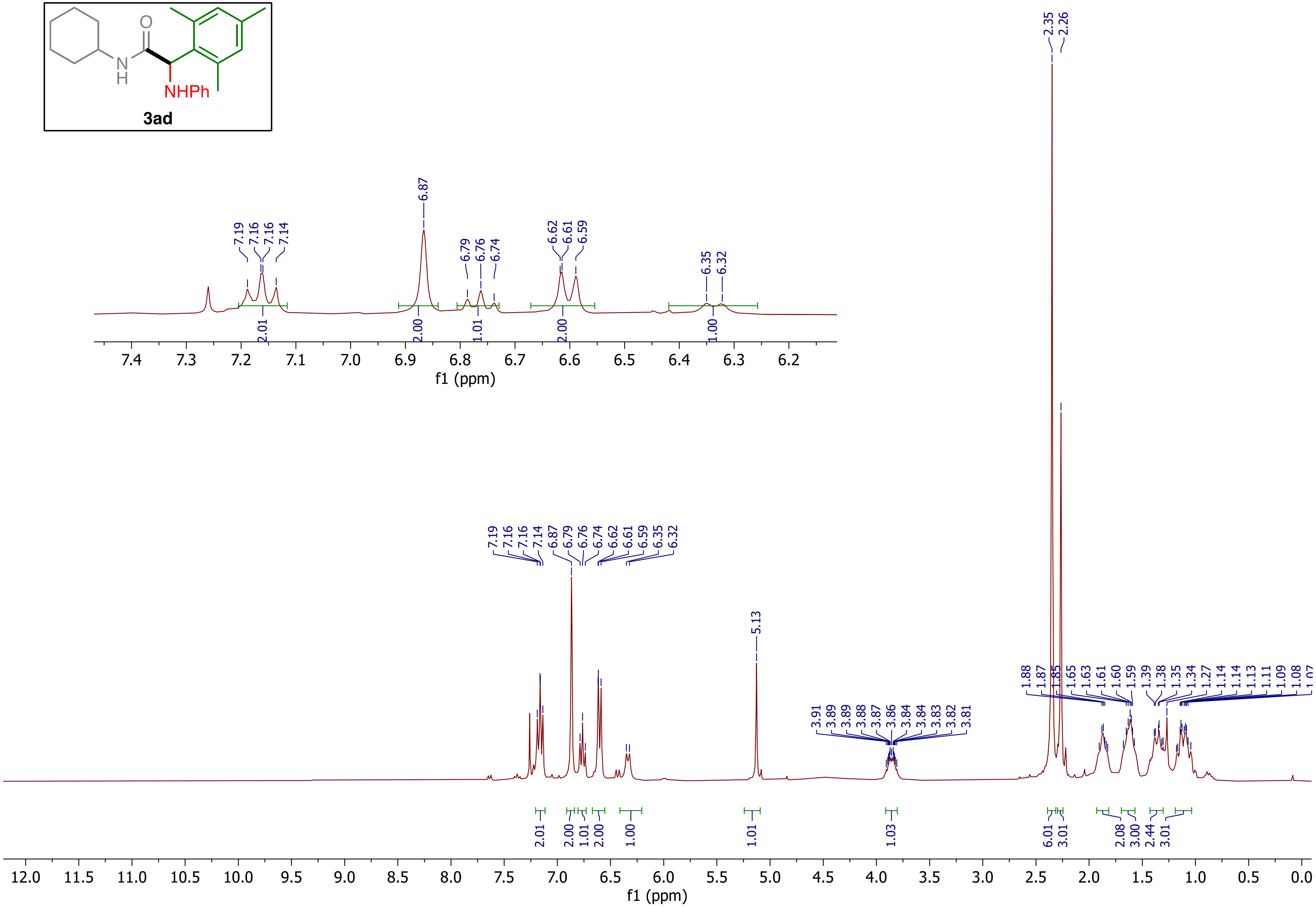

NHPh
3ad
f1 (ppm)
f1 (ppm)

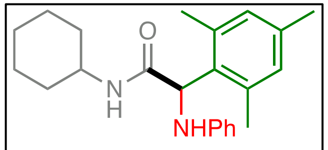
O
N
H
NHPh

3ad

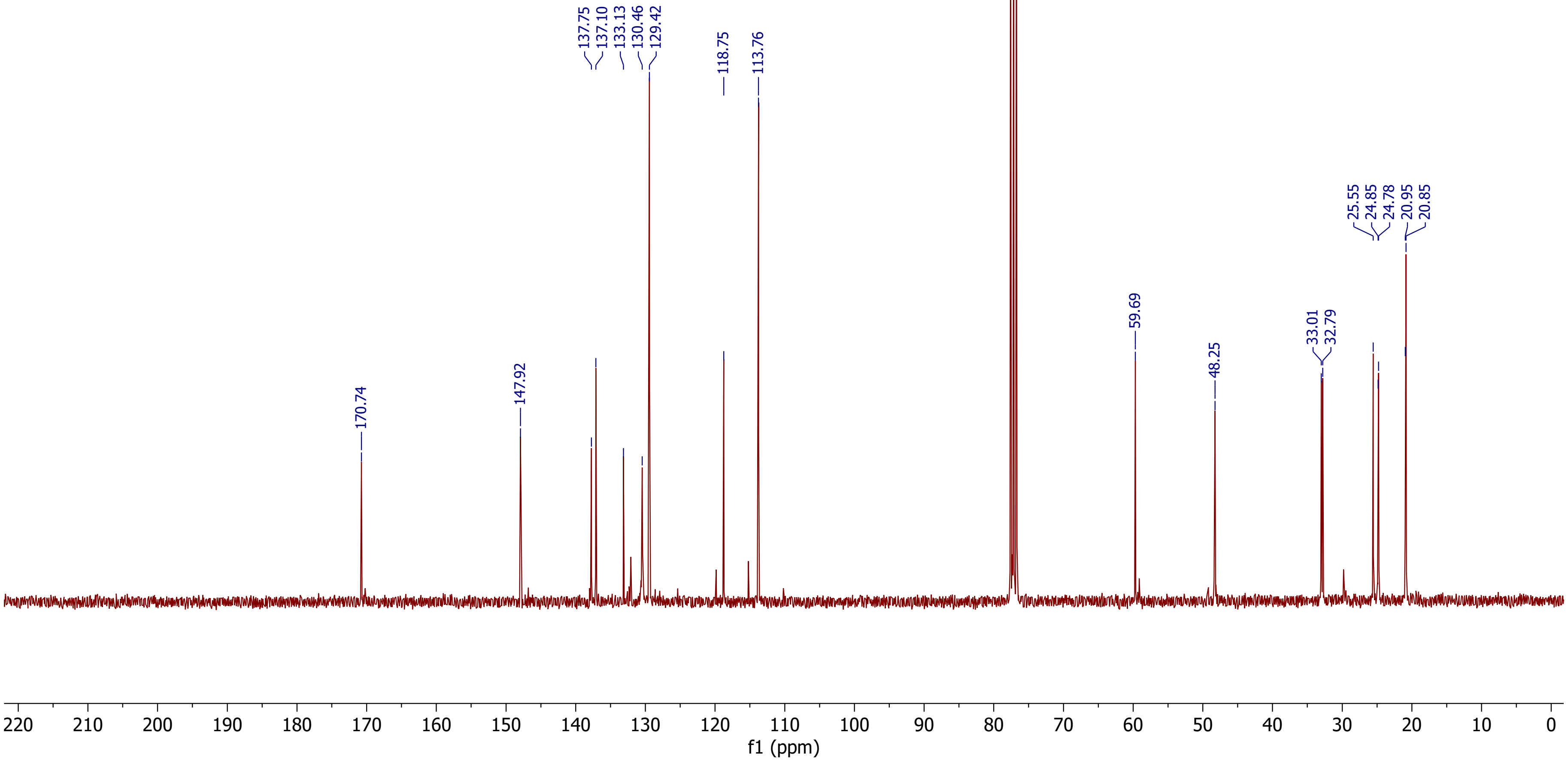
137.75
137.10
133.13
130.46
129.42
118.75
113.76
170.74
147.92
59.69
48.25
33.01
32.79
25.55
24.85
24.78
20.95
20.85
220 210 200 190 180 170 160 150 140 130 120 110 100 90 80 70 60 50 40 30 20 10 0
f1 (ppm)

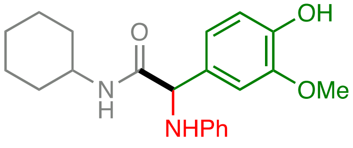
OH
OMe
O
N
H
NHPh

3ae

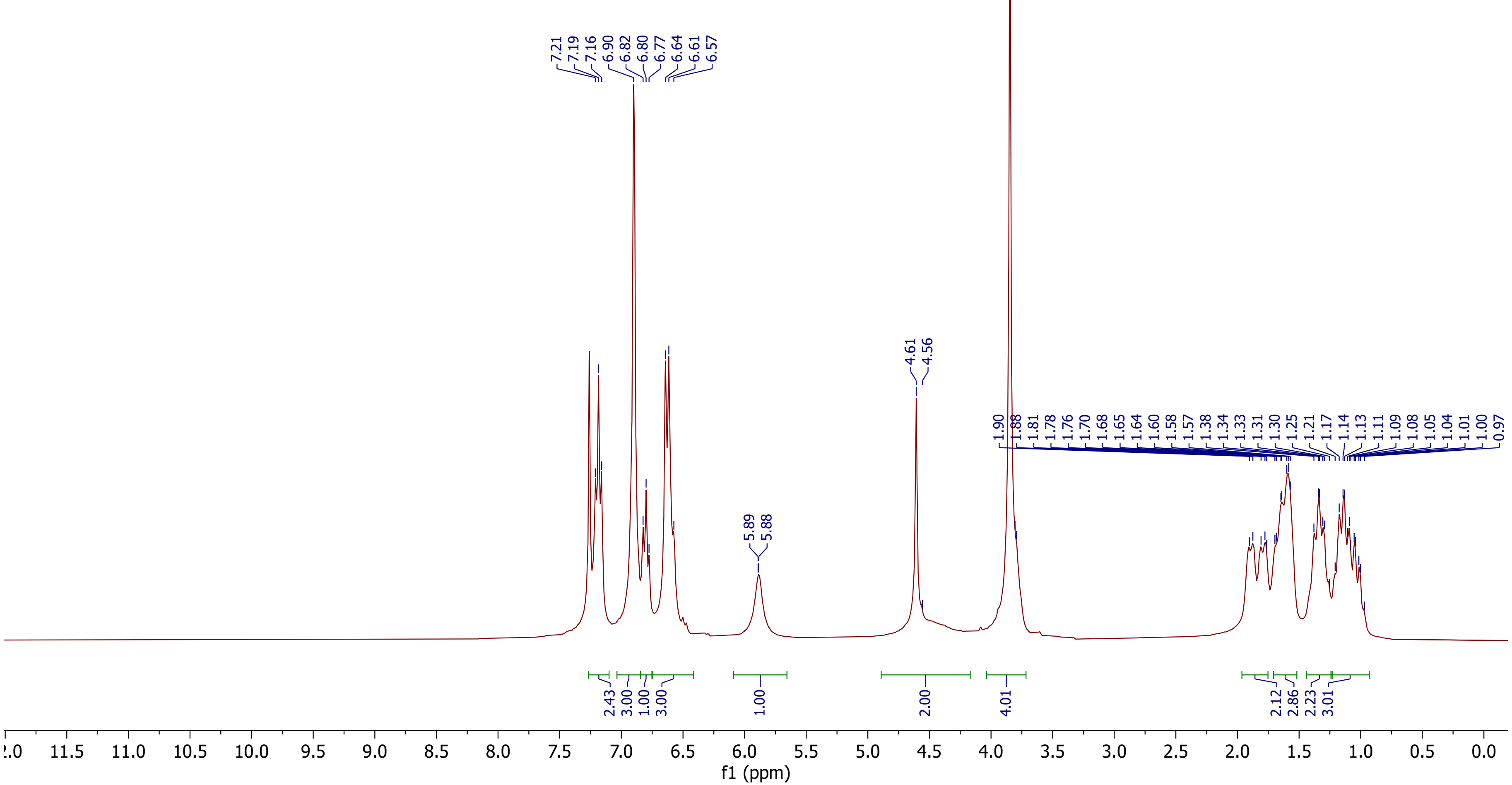
3.85
3.80
3.79
7.21
7.19
7.16
6.90
6.82
6.80
6.77
6.64
6.61
6.57
4.61
4.56
5.89
5.88
1.90
1.88
1.81
1.78
1.76
1.70
1.68
1.65
1.64
1.60
1.58
1.57
1.38
1.34
1.33
1.31
1.30
1.25
1.21
1.17
1.14
1.13
1.11
1.09
1.08
1.05
1.04
1.01
1.00
0.97
2.43
3.00
1.00
3.00
1.00
2.00
4.01
2.12
2.86
2.23
3.01
11.5
11.0
10.5
10.0
9.5
9.0
8.5
8.0
7.5
7.0
6.5
6.0
5.5
5.0
4.5
4.0
3.5
3.0
2.5
2.0
1.5
1.0
0.5
0.0
f1 (ppm)

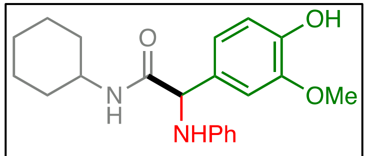
OH
O
N
H
OMe
NHPh

3ae

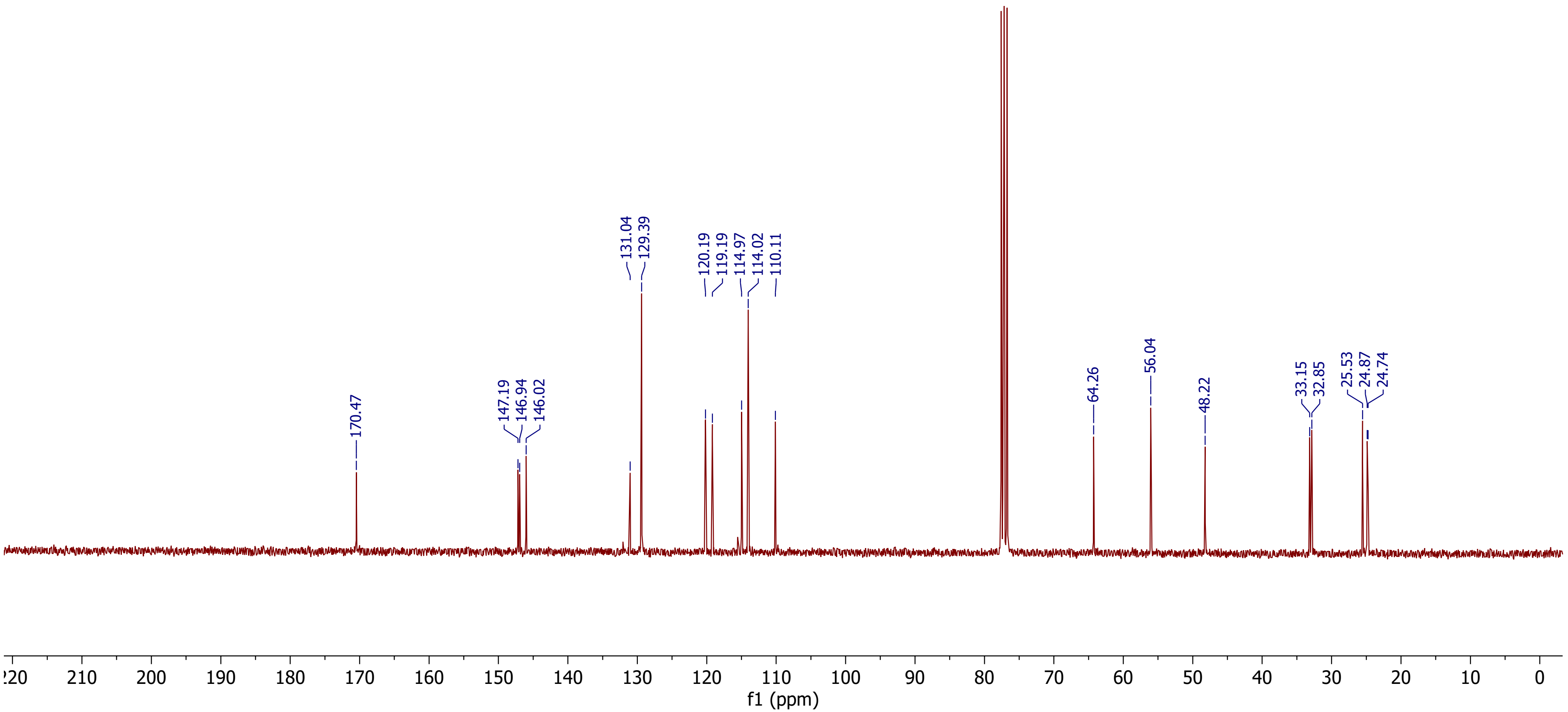
170.47
147.19
146.94
146.02
131.04
129.39
120.19
119.19
114.97
114.02
110.11
64.26
56.04
48.22
33.15
32.85
25.53
24.87
24.74
220
210
200
190
180
170
160
150
140
130
120
110
100
90
80
70
60
50
40
30
20
10
0
f1 (ppm)

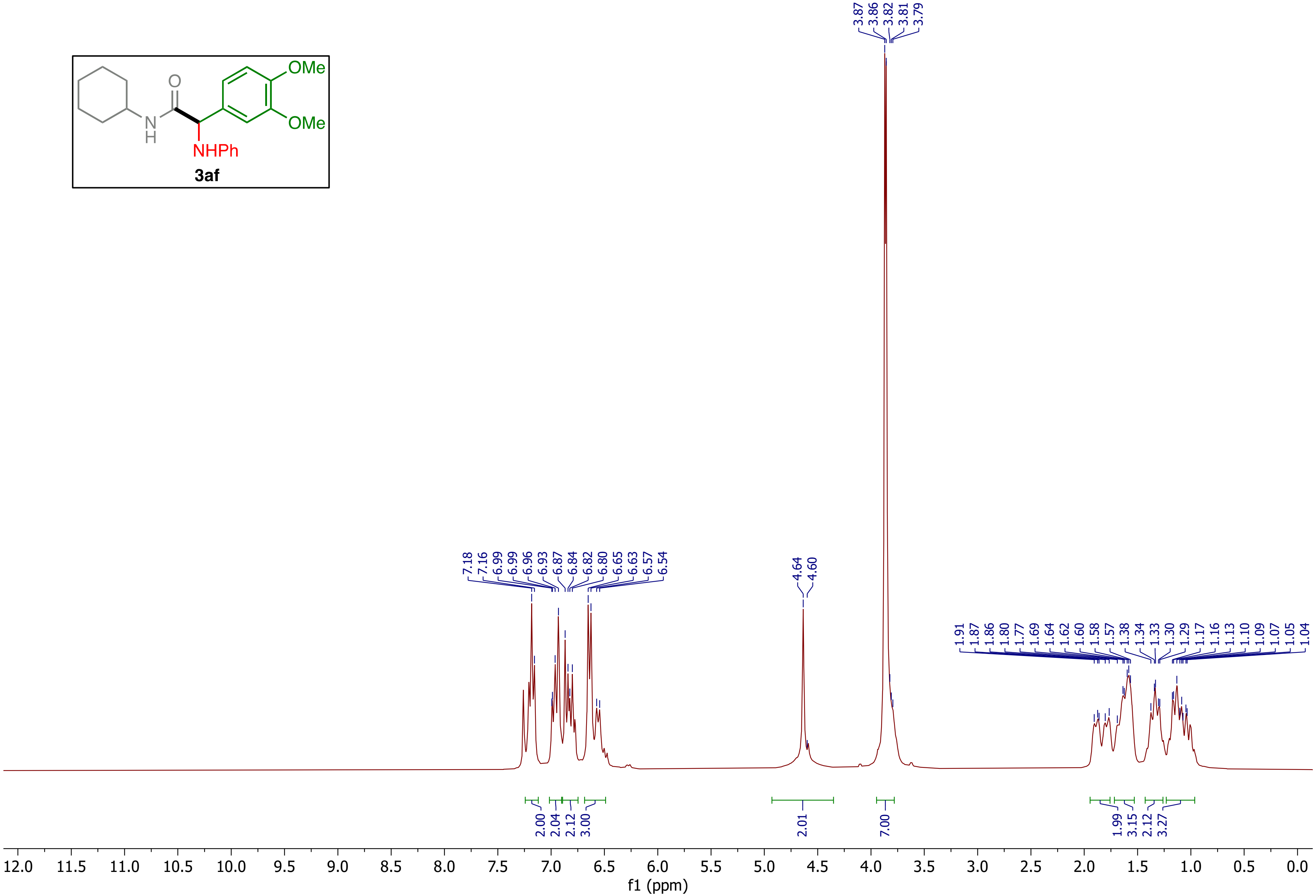
OMe
O
OMe
N
H
NHPh
3af
3.87
3.86
3.82
3.81
3.79
7.18
7.16
6.99
6.99
6.96
6.93
6.87
6.84
6.82
6.80
6.65
6.63
6.57
6.54
4.64
4.60
1.91
1.87
1.86
1.80
1.77
1.69
1.64
1.62
1.60
1.58
1.57
1.38
1.34
1.33
1.30
1.29
1.17
1.16
1.13
1.10
1.09
1.07
1.05
1.04
2.00
2.04
2.12
3.00
2.01
7.00
1.99
3.15
2.12
3.27
12.0 11.5 11.0 10.5 10.0 9.5 9.0 8.5 8.0 7.5 7.0 6.5 6.0 5.5 5.0 4.5 4.0 3.5 3.0 2.5 2.0 1.5 1.0 0.5 0.0
f1 (ppm)

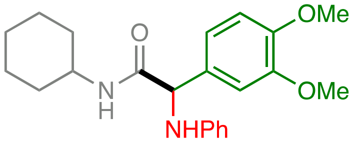
OMe
OMe
O
N
H
NHPh

3af

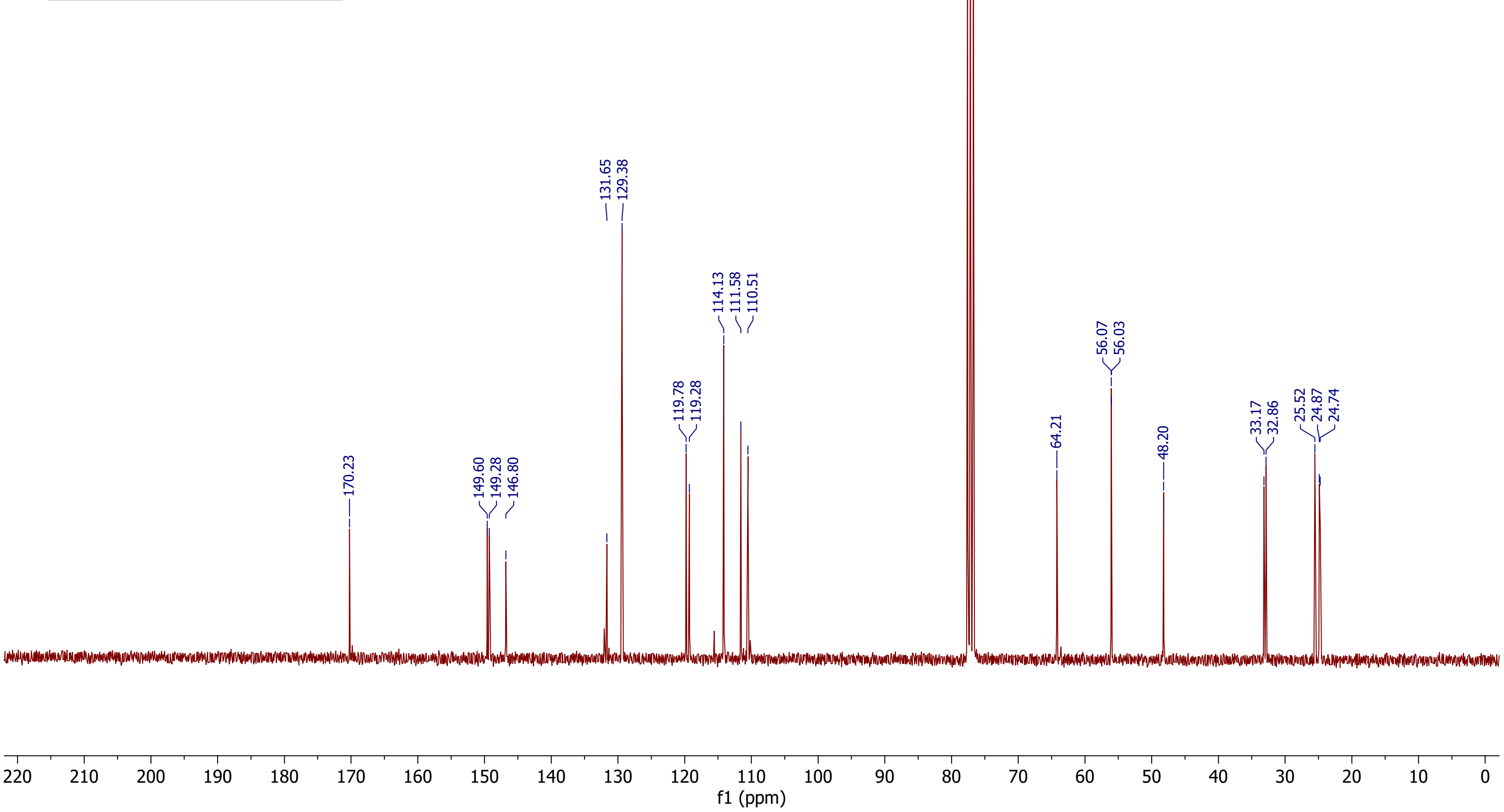
170.23
149.60
149.28
146.80
131.65
129.38
119.78
119.28
114.13
111.58
110.51
64.21
56.07
56.03
48.20
33.17
32.86
25.52
24.87
24.74
220 210 200 190 180 170 160 150 140 130 120 110 100 90 80 70 60 50 40 30 20 10 0
f1 (ppm)

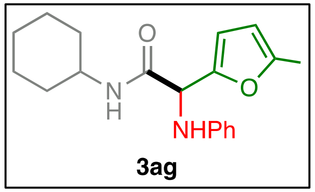

NHPh
3ag

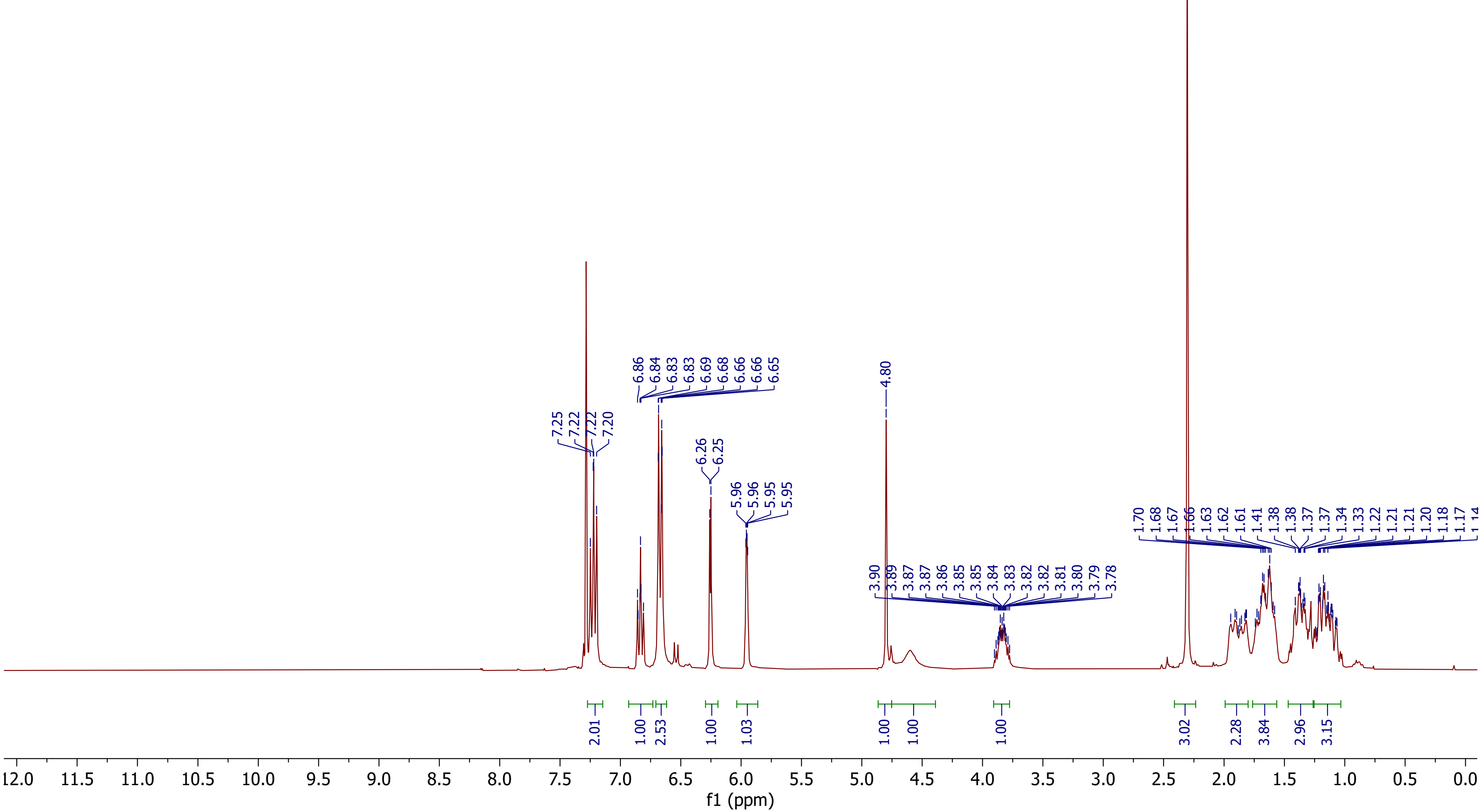

7.25
7.22
7.22
7.20
6.86
6.84
6.83
6.83
6.69
6.68
6.66
6.66
6.65
6.26
6.25
5.96
5.96
5.95
5.95
4.80
3.90
3.89
3.87
3.87
3.86
3.85
3.84
3.83
3.82
3.81
3.80
3.79
3.78
2.31
2.30
1.70
1.68
1.67
1.66
1.63
1.62
1.61
1.41
1.38
1.38
1.37
1.37
1.34
1.33
1.22
1.21
1.21
1.20
1.18
1.17
1.14
2.01
1.00
2.53
1.00
1.03
1.00
1.00
1.00
3.02
2.28
3.84
2.96
3.15
12.0
11.5
11.0
10.5
10.0
9.5
9.0
8.5
8.0
7.5
7.0
6.5
6.0
5.5
5.0
4.5
4.0
3.5
3.0
2.5
2.0
1.5
1.0
0.5
0.0
f1 (ppm)

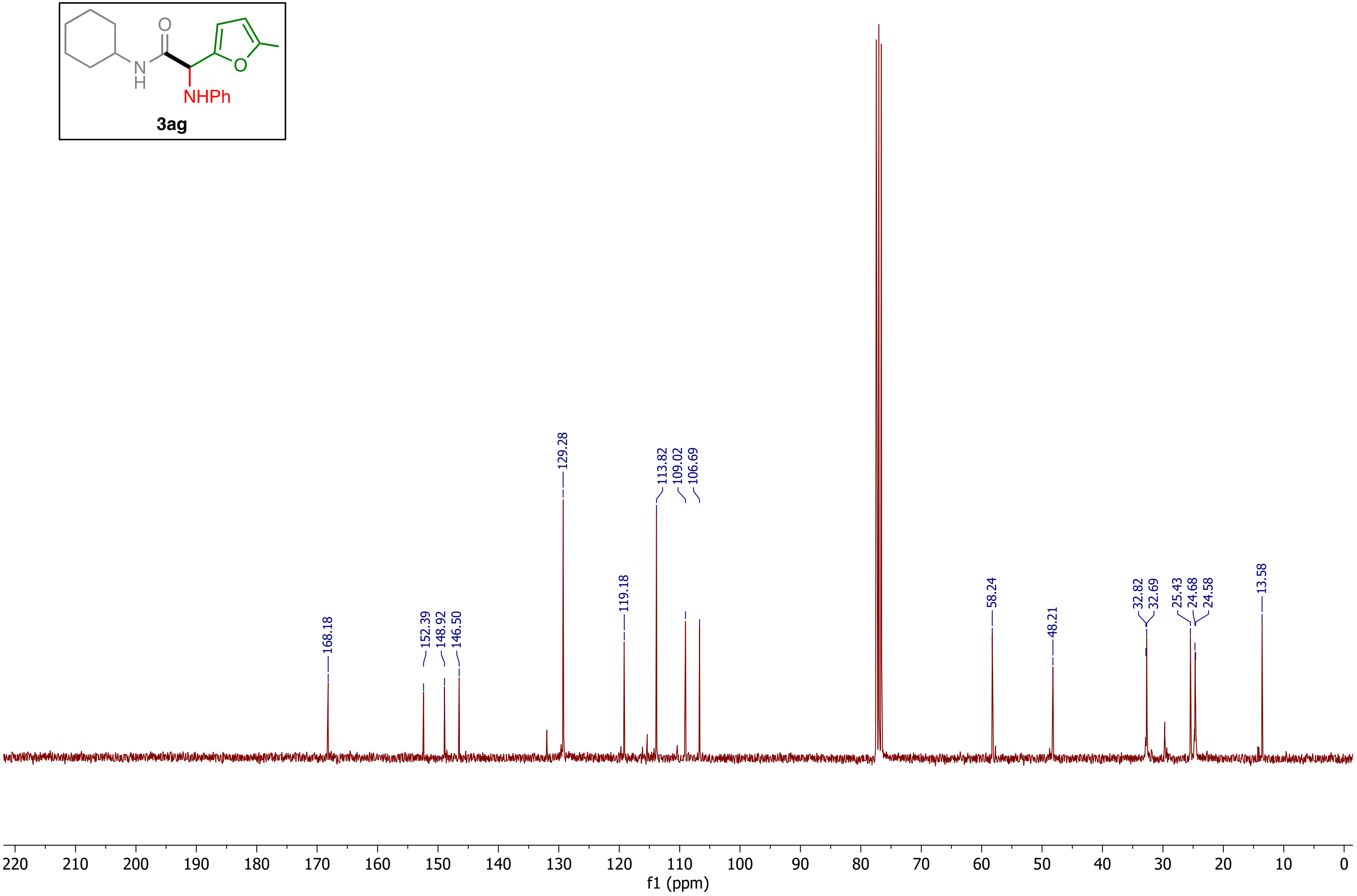

O
N
H
NHPh
O
3ag
168.18
152.39
148.92
146.50
129.28
119.18
113.82
109.02
106.69
58.24
48.21
32.82
32.69
25.43
24.68
24.58
13.58
220 210 200 190 180 170 160 150 140 130 120 110 100 90 80 70 60 50 40 30 20 10 0
f1 (ppm)

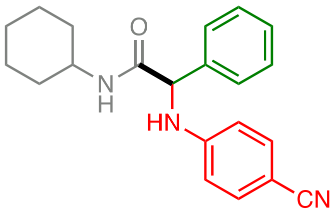

O
N
H
HN
CN

3ah

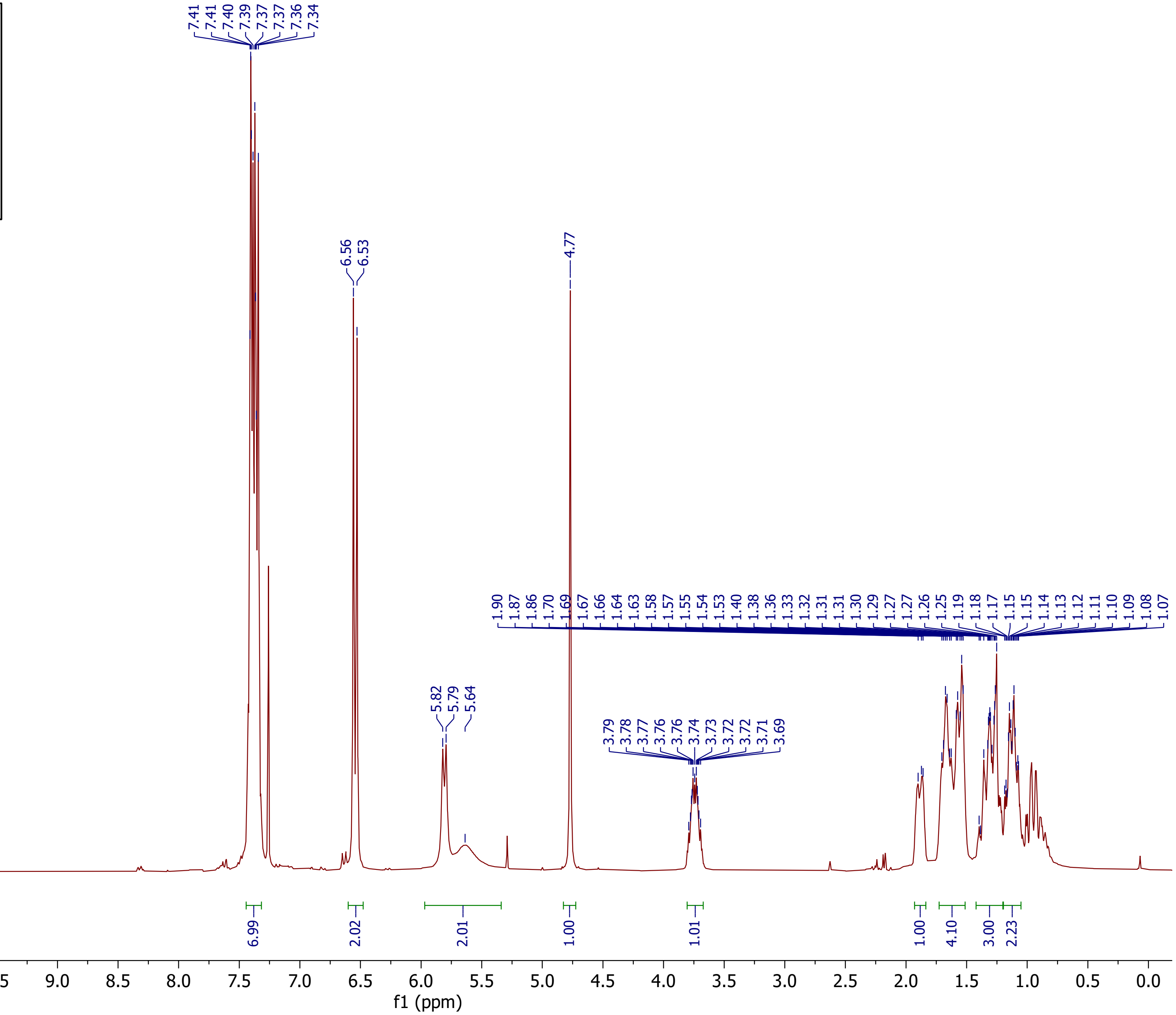

7.41
7.41
7.40
7.39
7.37
7.37
7.36
7.34
6.56
6.53
4.77
5.82
5.79
5.64
3.79
3.78
3.77
3.76
3.76
3.74
3.73
3.72
3.72
3.71
3.69
1.90
1.87
1.86
1.70
1.69
1.67
1.66
1.64
1.63
1.58
1.57
1.55
1.54
1.53
1.40
1.38
1.36
1.33
1.32
1.31
1.31
1.30
1.29
1.27
1.27
1.26
1.25
1.19
1.18
1.17
1.15
1.15
1.14
1.13
1.12
1.11
1.10
1.09
1.08
1.07
6.99
2.02
2.01
1.00
1.01
1.00
4.10
3.00
2.23
f1 (ppm)

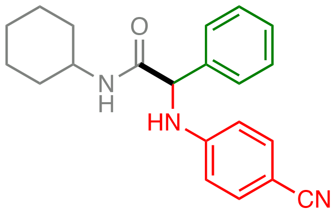
O
N
H
HN
CN

3ah

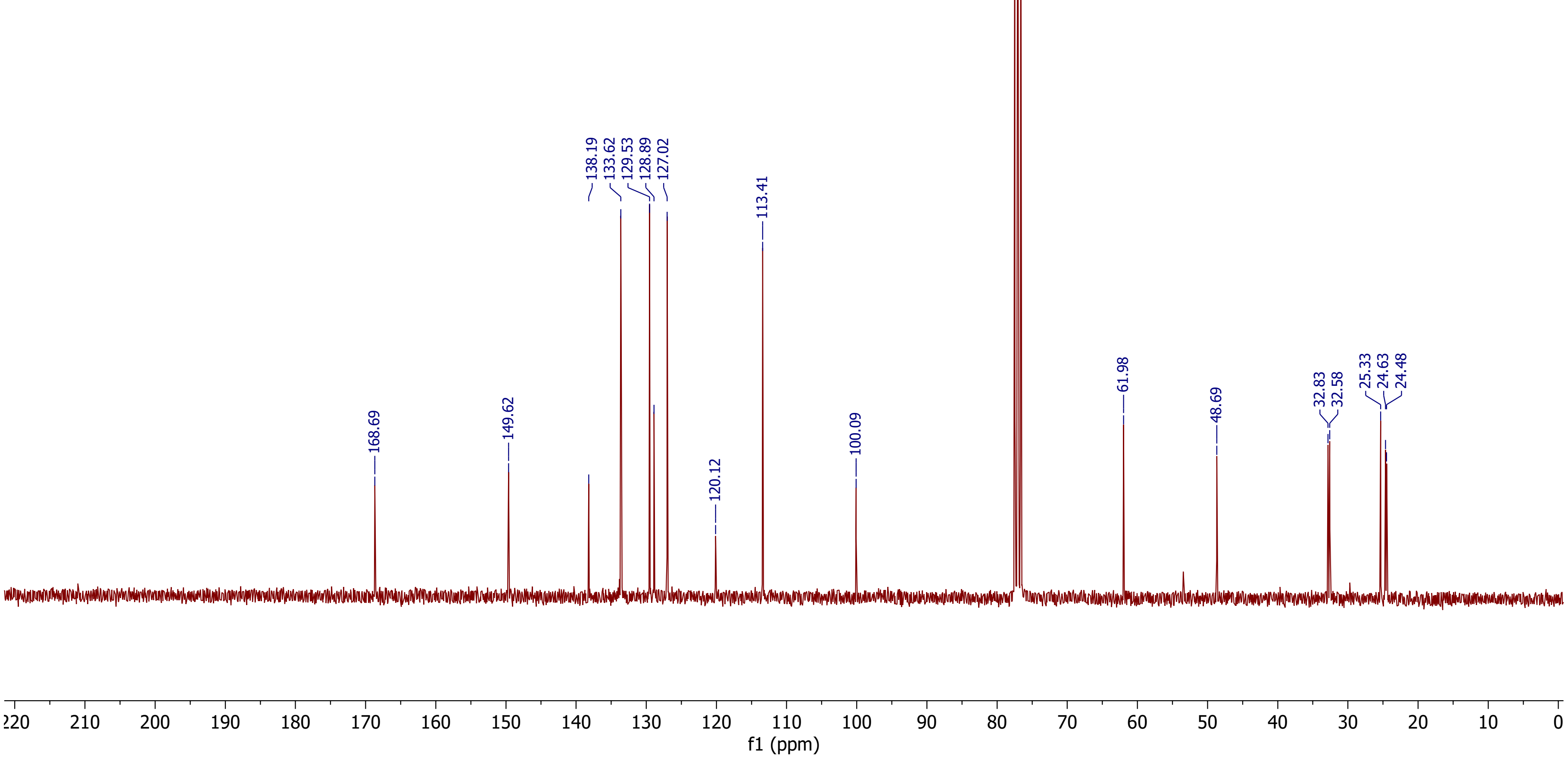
168.69
149.62
138.19
133.62
129.53
128.89
127.02
120.12
113.41
100.09
61.98
48.69
32.83
32.58
25.33
24.63
24.48
220 210 200 190 180 170 160 150 140 130 120 110 100 90 80 70 60 50 40 30 20 10 0
f1 (ppm)

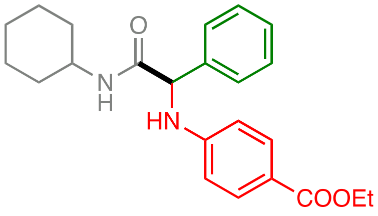

HN
N
H
O
COOEt

3ai

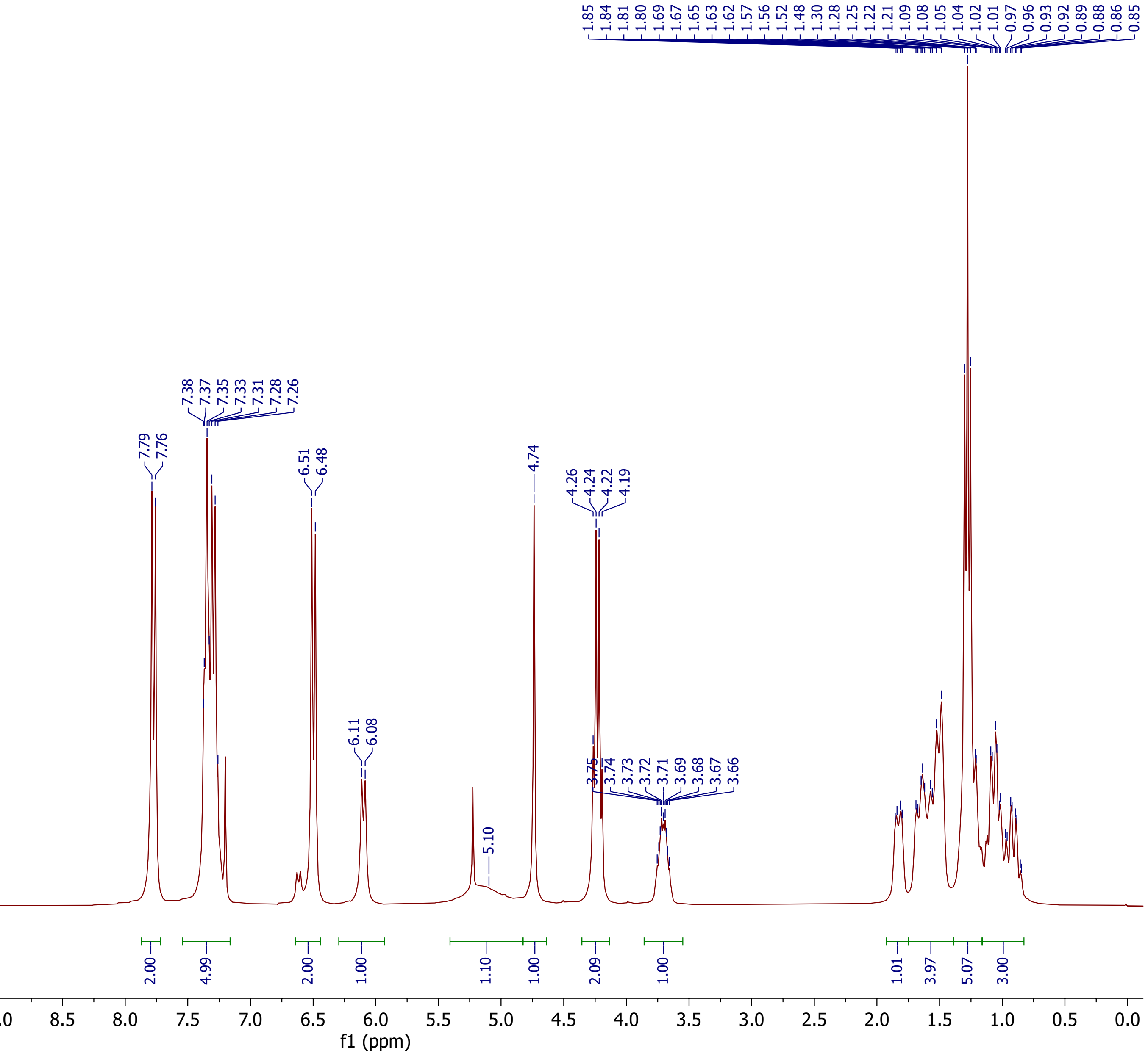

1.85 1.84 1.81 1.80 1.69 1.67 1.65 1.63 1.62 1.57 1.56 1.52 1.48 1.30 1.28 1.25 1.22 1.21 1.09 1.08 1.05 1.04 1.02 1.01 0.97 0.96 0.93 0.92 0.89 0.88 0.86 0.85
7.38 7.37 7.35 7.33 7.31 7.28 7.26
7.79 7.76
6.51 6.48
4.74
4.26 4.24 4.22 4.19
6.11 6.08
3.75 3.74 3.73 3.72 3.71 3.69 3.68 3.67 3.66
5.10
2.00 4.99 2.00 1.00 1.10 1.00 2.09 1.00 1.01 3.97 5.07 3.00
12.0 11.5 11.0 10.5 10.0 9.5 9.0 8.5 8.0 7.5 7.0 6.5 6.0 5.5 5.0 4.5 4.0 3.5 3.0 2.5 2.0 1.5 1.0 0.5 0.0
f1 (ppm)

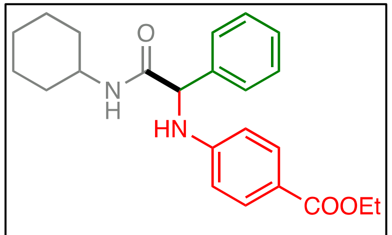
O
N
H
HN
COOEt

3ai

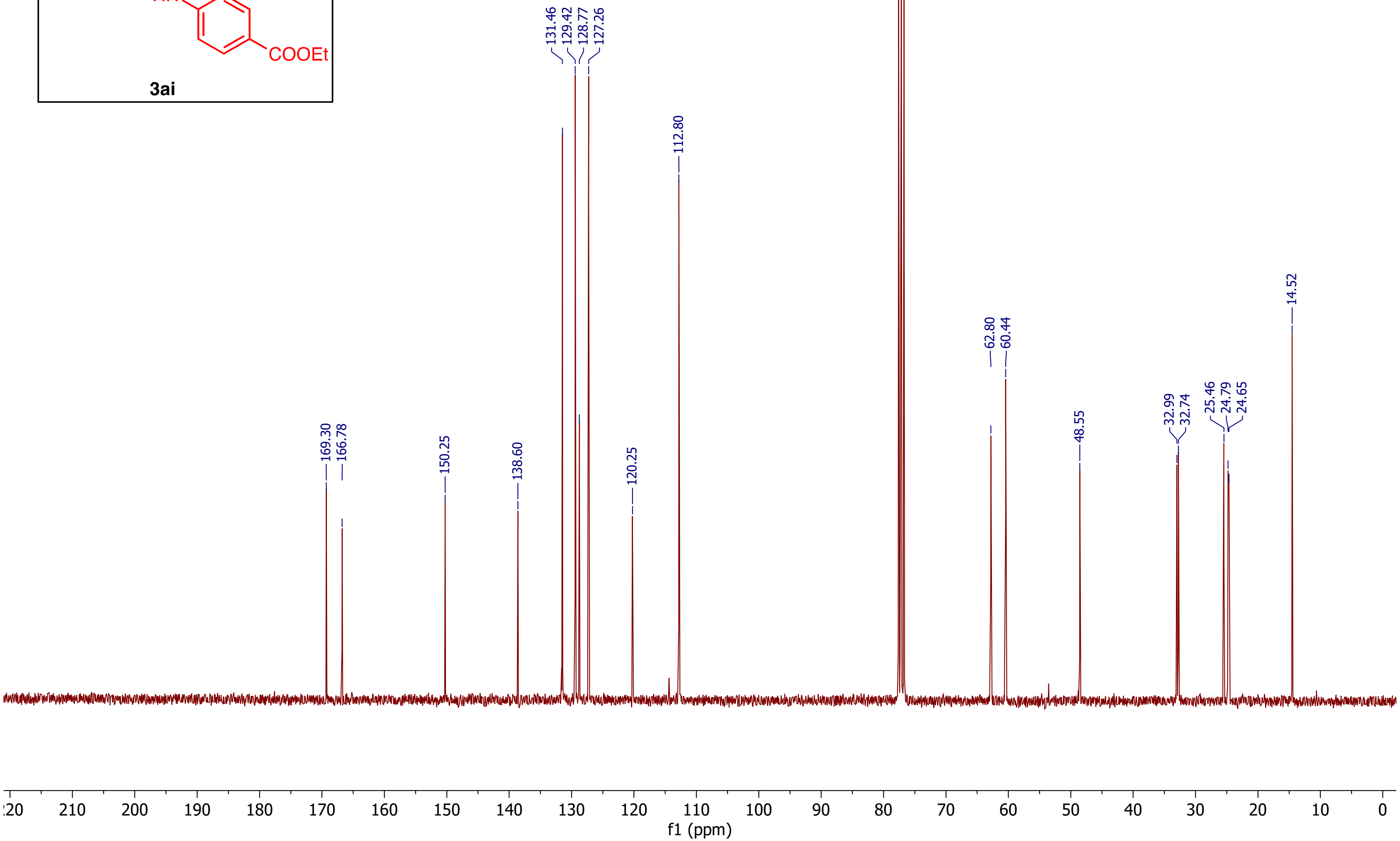
169.30
166.78
150.25
138.60
131.46
129.42
128.77
127.26
120.25
112.80
62.80
60.44
48.55
32.99
32.74
25.46
24.79
24.65
14.52
220
210
200
190
180
170
160
150
140
130
120
110
100
90
80
70
60
50
40
30
20
10
0
f1 (ppm)

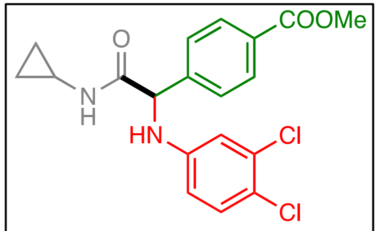
COOMe
O
N
H
HN
Cl
Cl

3aj

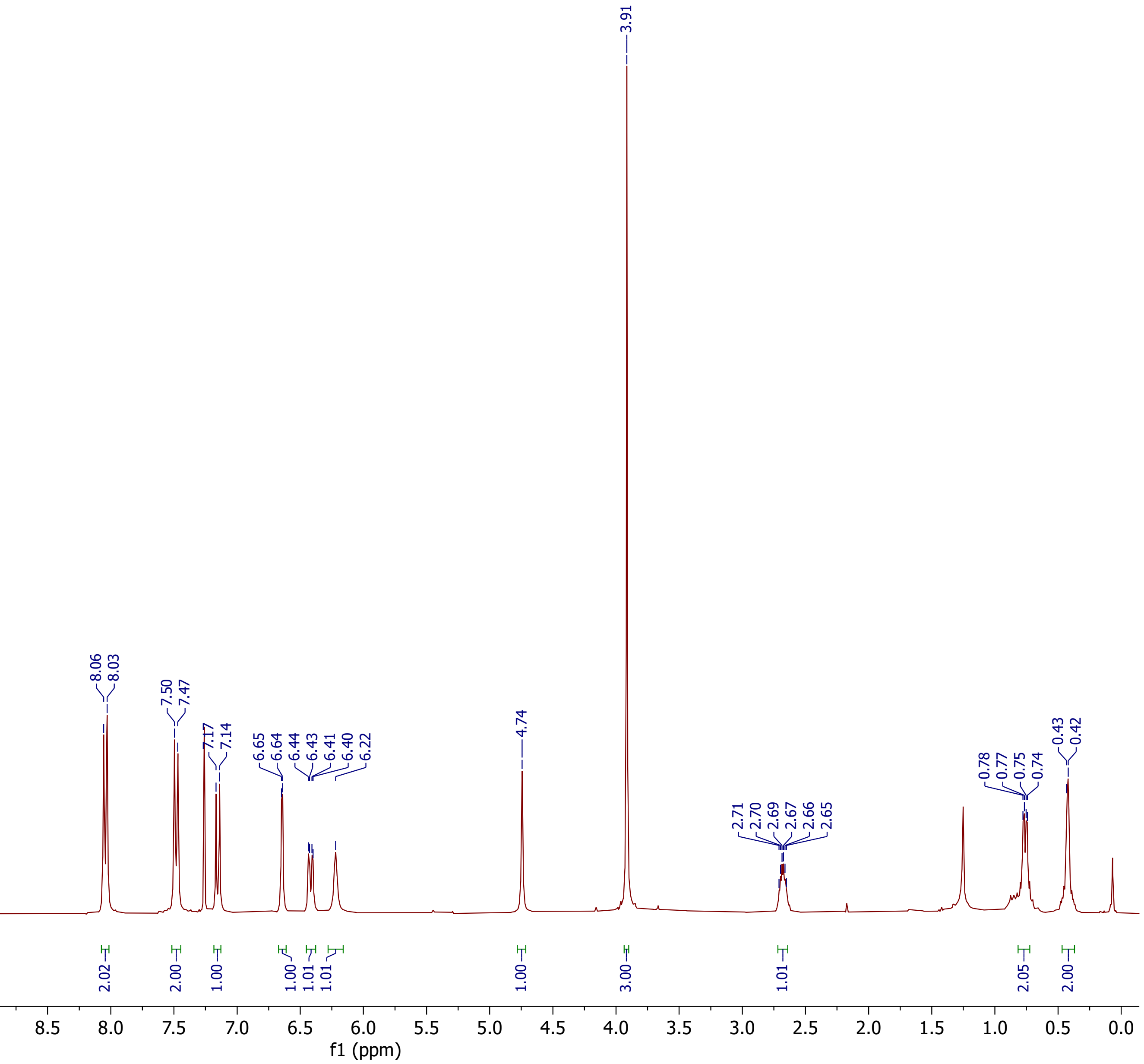
8.06
8.03
7.50
7.47
7.17
7.14
6.65
6.64
6.44
6.43
6.41
6.40
6.22
4.74
3.91
2.71
2.70
2.69
2.67
2.66
2.65
0.78
0.77
0.75
0.74
0.43
0.42
2.02
2.00
1.00
1.00
1.01
1.01
1.00
3.00
1.01
2.05
2.00
12.0 11.5 11.0 10.5 10.0 9.5 9.0 8.5 8.0 7.5 7.0 6.5 6.0 5.5 5.0 4.5 4.0 3.5 3.0 2.5 2.0 1.5 1.0 0.5 0.0
f1 (ppm)

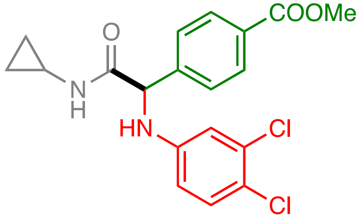
COOMe
O
N
H
HN
Cl
Cl

3aj

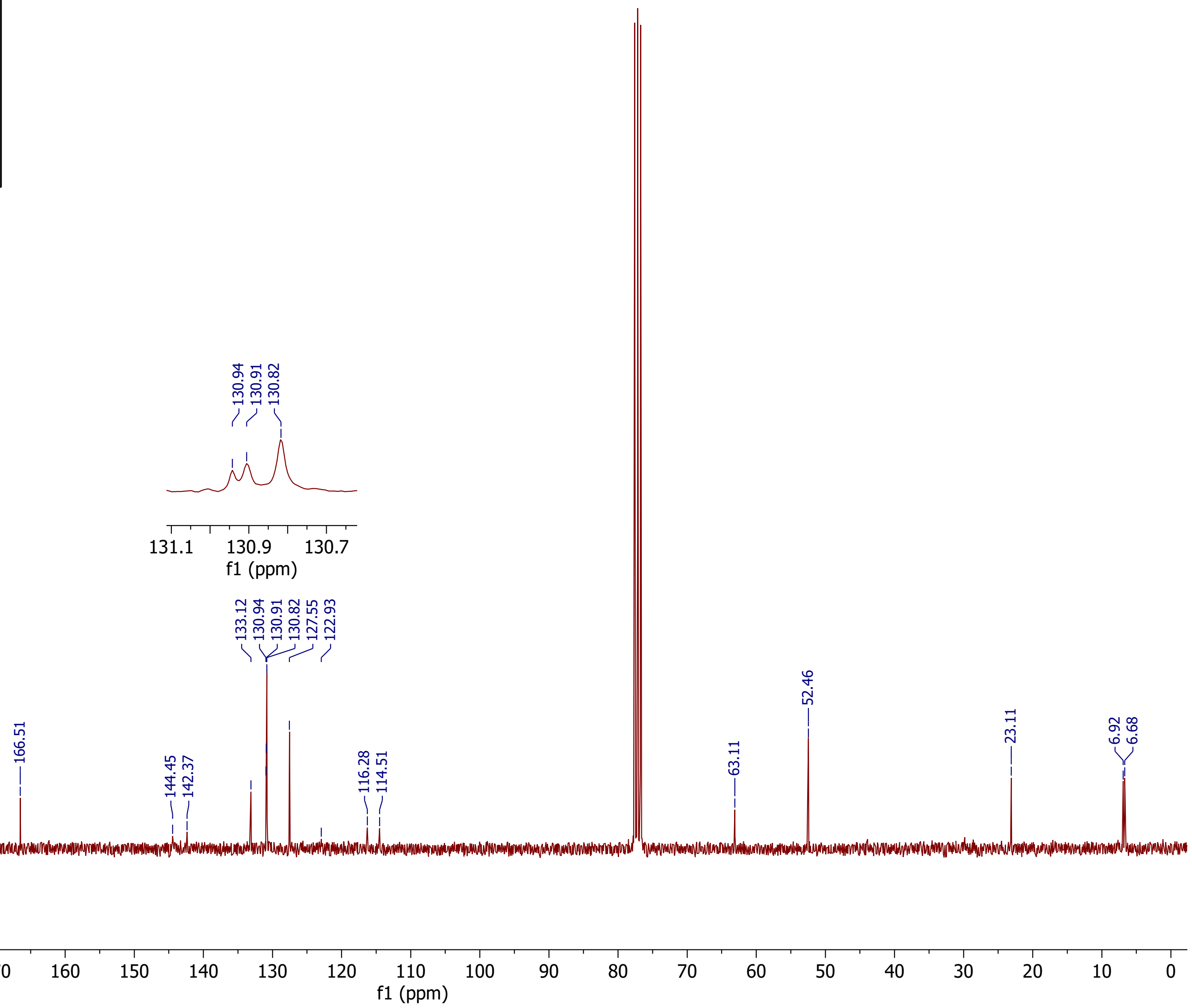
130.94
130.91
130.82
131.1
130.9
130.7
f1 (ppm)
133.12
127.55
122.93
170.65
166.51
144.45
142.37
116.28
114.51
63.11
52.46
23.11
6.92
6.68
220
210
200
190
180
170
160
150
140
130
120
110
100
90
80
70
60
50
40
30
20
10
0
f1 (ppm)

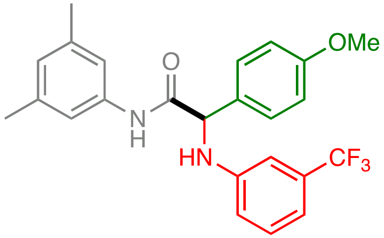
OMe
O
N
H
HN
CF3

3ak

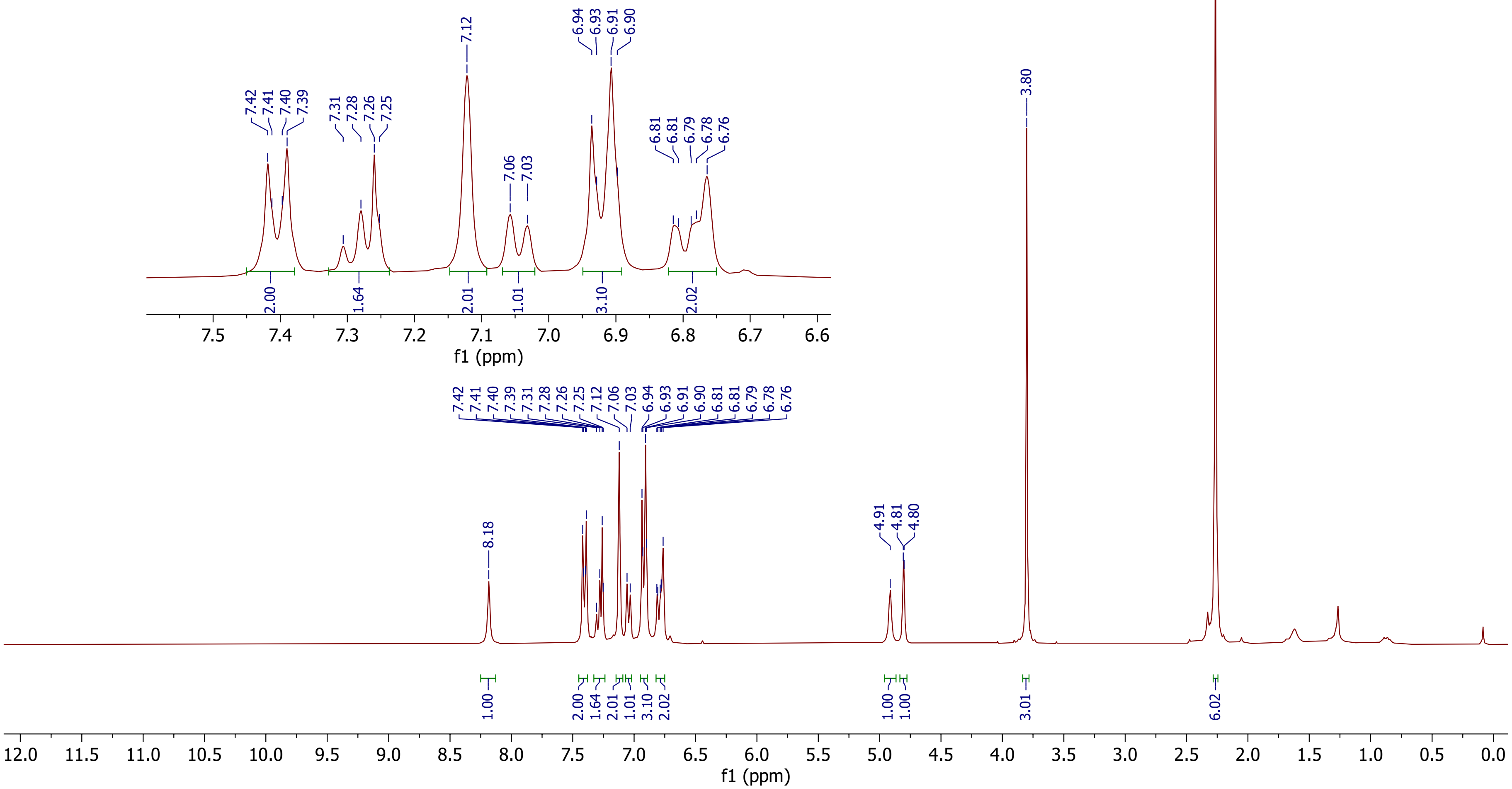
7.42
7.41
7.40
7.39
7.31
7.28
7.26
7.25
7.12
7.06
7.03
6.94
6.93
6.91
6.90
6.81
6.81
6.79
6.78
6.76
2.00
1.64
2.01
1.01
3.10
2.02
7.5 7.4 7.3 7.2 7.1 7.0 6.9 6.8 6.7 6.6
f1 (ppm)
8.18
4.91
4.81
4.80
3.80
2.27
1.00
2.00
1.64
2.01
1.01
3.10
2.02
1.00
1.00
3.01
6.02
12.0 11.5 11.0 10.5 10.0 9.5 9.0 8.5 8.0 7.5 7.0 6.5 6.0 5.5 5.0 4.5 4.0 3.5 3.0 2.5 2.0 1.5 1.0 0.5 0.0
f1 (ppm)

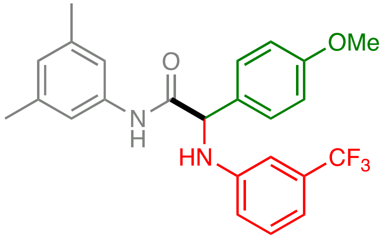
OMe
O
N
H
HN
CF3

3ak

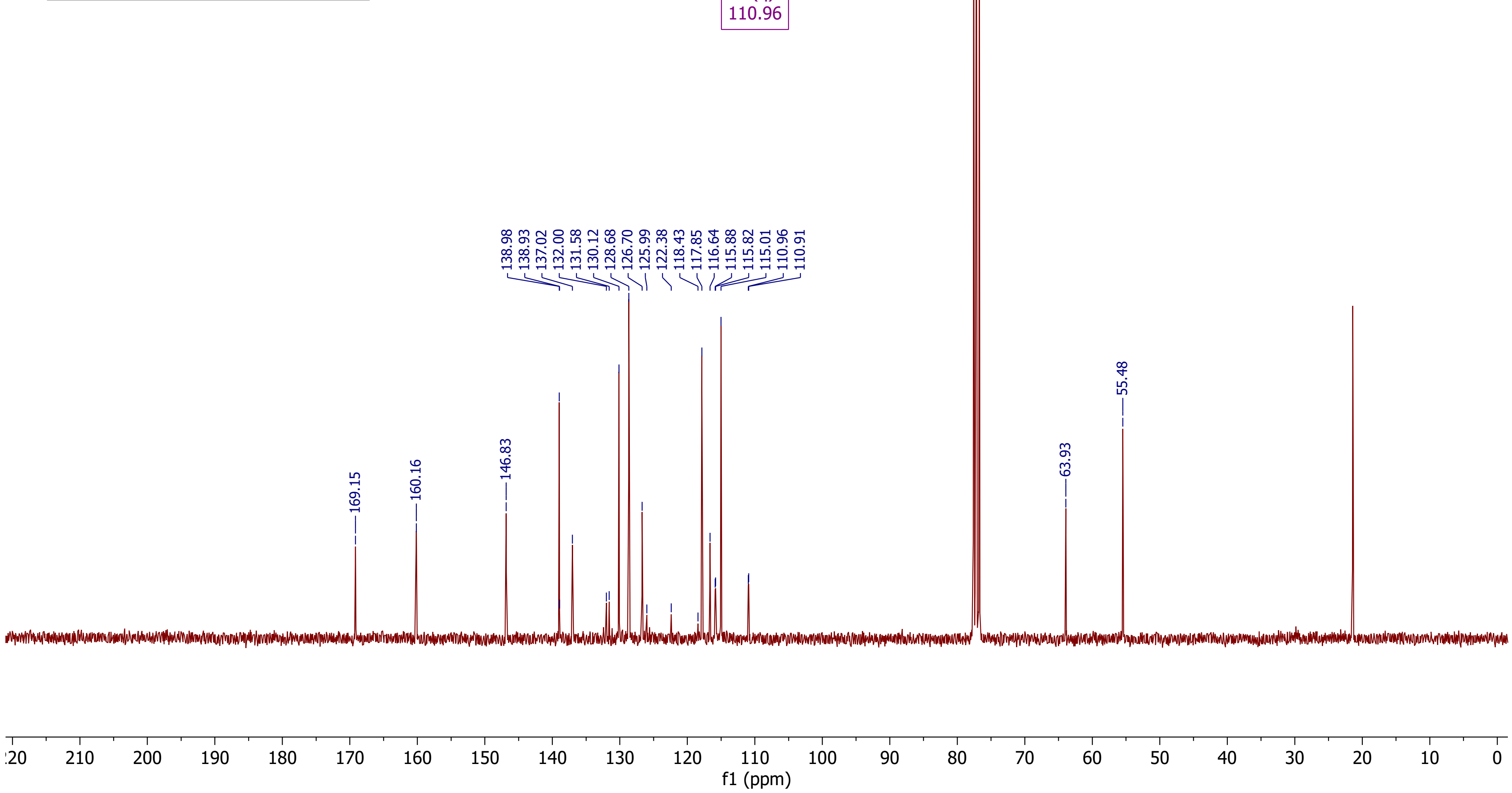
4.37 3.71
4.19 3.65
3.57 3.50
B (q)
115.85
A (q)
110.96
138.98
138.93
137.02
132.00
131.58
130.12
128.68
126.70
125.99
122.38
118.43
117.85
116.64
115.88
115.82
115.01
110.96
110.91
169.15
160.16
146.83
63.93
55.48
f1 (ppm)
